# Supplementary material for: Healthy Parent Carers programme: mixed methods process evaluation and refinement of a health promotion intervention
Source: BMJ Open. 2021 Aug 24;11(8):e045570. doi: 10.1136/bmjopen-2020-045570 (PMC8388296; doi:10.1136/bmjopen-2020-045570)
Supplement: Supplementary data [file bmjopen-2020-045570supp001.pdf]

Supplementary Document 1a. Healthy Parent Carers intervention details

The details of the intervention are also reported in the intervention development paper (Borek et al., 2018) and the feasibility study protocol (Bjornstad et al., 2019). The results of the feasibility study are reported separately. The intervention details below are reported using the checklist for reporting of group-based interventions (Borek et al., 2015).

**INTERVENTION DESIGN**	
1. Intervention source or development methods	Developed based on an intervention mapping approach (Bartholomew et al., 2016), in partnership with parent carers. Details are described in Borek et al., 2018.
2. General setting	Community setting (with 6 sites/venues)
3. Venue characteristics	6 sites/venues, with different characteristics (2 special schools, 1 children's hospice, 1 Parent Carer Forum premises, 1 adult learning community venue, and 1 hotel regularly used for parent carer meetings).<br><br>All rooms set up to enable interaction between participants (i.e. facing each other).
4. Total number of group sessions	6 or 12 sessions
5. Length of group sessions	6 daytime sessions of 4 hours (2 modules per session)<br>or 12 evening sessions of 2 hours (1 module per session)
6. Frequency of group sessions	Weekly
7. Duration of the intervention	6 or 12 weeks
**INTERVENTION CONTENT**	
8. Change mechanisms or theories of change	The intervention logic model and intended mechanisms of change are reported in Borek et al., 2018. In brief, the intervention aimed to increase knowledge of health-related behaviours, skills in making health-related changes, improve attitudes towards change, increase self-efficacy, and provide social support to make changes, leading to behaviour change (based on CLANGERS) and psychological change (increased empowerment and resilience), leading to improved health and wellbeing.
9. Change techniques	• Provide/exchange information about behaviour-health link<br>• Provide/exchange instructions on practical strategies<br>• Prompt barrier identification and problem solving<br>• Prompt practice<br>• Prompt specific goal setting (for behaviours)<br>• Prompt use of prompts and rewards<br>• Prompt self-monitoring<br>• Prompt goal/progress review (behaviours)<br>• Provide general support and encouragement<br>• Provide opportunities for social comparisons
10. Session content	12 modules:<br>1. Introduction to health and wellbeing<br>2. Introduction to CLANGERS<br>3. Connect<br>4. Learn<br>5. be Active<br>6. take Notice<br>7. Give<br>8. Eat well

	9. Relax<br>10. Sleep well<br>11. Managing stress<br>12. Keeping healthy
11. Sequencing of sessions	Yes – sessions delivered sequentially (as above)
12. Participants' materials	In the sessions: goal setting and CLANGERS diary sheets given to participants, and other materials as parts of group activities.<br><br>Online Healthy Parent Carers materials (written information for each module, links to videos watched in the sessions)
13. Activities during the sessions	Each session started with a 'warm-up' activity and a review of the past week. This was followed by discussions focused on the topic of each module (e.g. 'what does it mean to you to relax? How do you relax? Why is relaxing important? What might stop you from, or help you, relax?). For each module there was a practical activity to help illustrate the key messages or the CLANGERS (e.g., colouring, 'a day in life of a parent carer', a mindful walk). For details, see Borek et al., 2018.
14. Methods for checking fidelity of delivery	Checklists were completed by the facilitators after each session; a sample of audio-recordings of group sessions were checked using the checklist independently (double-checking) by two researchers. Details are reported separately.
**PARTICIPANTS**	
15. Group composition	Parent carers, all female, except for two groups one of which had one male carer, the other of which had two. Ages ranged from 26-71 years (mean 42.5 years)
16. Methods for group allocation	Participants were recruited locally for each site (then randomised to the group intervention or control).
17. Continuity of participants' group membership	The same participants remained in the same group for the duration of the intervention.
18. Group size	7-10 participants were assigned per group. The attendance varied between sessions and groups (lowest attendance being 2 participants over 6 sessions). Details of attendance are reported separately.
**FACILITATORS**	
19. Number of facilitators	2 facilitators per group: one lead facilitator (LF) and one assistant facilitator (AF).<br><br>Overall 2 LF and 6 AF delivered the programme
20. Continuity of facilitators' group assignment	The same pairs of LF and AF facilitated the same groups.
21. Facilitators' professional background	LF: experienced facilitators of training for parent carers delivered through the Council for Disabled Children (e.g., the Expert Parent Programme).<br><br>AF: three had some experience of facilitating parent carer groups and/or training.
22. Facilitators' personal characteristics	All facilitators were parent carers. Both LF were female and one of the six AFs was male.
23. Facilitators' training in intervention delivery	LF received 4 (2 x 2) days of training in delivering the programme; one block of 2 days of these were delivered

	together for lead and assistant facilitators, LFs and AFs also received one day of refresher training.
24. Facilitators' training in group facilitation	LF were trained and experienced in group facilitation as part of their facilitator/trainer roles for the Council of Disabled Children.<br><br>All facilitators received an overview of facilitating groups and managing group dynamics as part of the training in delivering the intervention.
25. Facilitators' materials	Facilitators delivered the sessions using a manual with instructions outlining all session activities.
26. Intended facilitation style	Participant-centred, interactive, and discussion-based (not didactic).

Supplementary Document 1b. TIDieR checklist for HPC intervention

TIDieR Template for Intervention Description and Replication

The TIDieR (Template for Intervention Description and Replication) Checklist*:

Information to include when describing an intervention and the location of the information

Item number	Item	Where located ** Primary paper (page or appendix number)	Other † (details)
	**BRIEF NAME**		
**1.**	Provide the name or a phrase that describes the intervention.	__p4_________	_______________
	**WHY**		
**2.**	Describe any rationale, theory, or goal of the elements essential to the intervention.	__p4_________	______________
	**WHAT**		
**3.**	Materials: Describe any physical or informational materials used in the intervention, including those provided to participants or used in intervention delivery or in training of intervention providers. Provide information on where the materials can be accessed (e.g. online appendix, URL).	__p5________	Box 1_______
**4.**	Procedures: Describe each of the procedures, activities, and/or processes used in the intervention, including any enabling or support activities.	__p6_________	Supplementary document 1a parts 8-13
	**WHO PROVIDED**		
**5.**	For each category of intervention provider (e.g. psychologist, nursing assistant), describe their expertise, background and any specific training given.	__p5_________	Box 2, Supplementary document 1a parts 21-25
	**HOW**		
**6.**	Describe the modes of delivery (e.g. face-to-face or by some other mechanism, such as internet or telephone) of the intervention and whether it was provided individually or in a group.	__p5_________	Box 1________

	**WHERE**		
**7.**	Describe the type(s) of location(s) where the intervention occurred, including any necessary infrastructure or relevant features.	___p5________	Supplementary document 1a, parts 2-3
	**WHEN and HOW MUCH**		
**8.**	Describe the number of times the intervention was delivered and over what period of time including the number of sessions, their schedule, and their duration, intensity or dose.	___p5________	Supplementary document 1a, parts 4-7
	**TAILORING**		
**9.**	If the intervention was planned to be personalised, titrated or adapted, then describe what, why, when, and how.	___N/A_______	_____________
	**MODIFICATIONS**		
**10.‡**	If the intervention was modified during the course of the study, describe the changes (what, why, when, and how).	___N/A_______	_____________
	**HOW WELL**		
**11.**	Planned: If intervention adherence or fidelity was assessed, describe how and by whom, and if any strategies were used to maintain or improve fidelity, describe them.	___p7 _______	Supplementary document 4; Bjornstad et al. (2021)
**12.‡**	Actual: If intervention adherence or fidelity was assessed, describe the extent to which the intervention was delivered as planned.	___p7________	_____________

** **Authors** - use N/A if an item is not applicable for the intervention being described. **Reviewers** – use '?' if information about the element is not reported/not sufficiently reported.

† If the information is not provided in the primary paper, give details of where this information is available. This may include locations such as a published protocol or other published papers (provide citation details) or a website (provide the URL).

‡ If completing the TIDieR checklist for a protocol, these items are not relevant to the protocol and cannot be described until the study is complete.

* We strongly recommend using this checklist in conjunction with the TIDieR guide (see *BMJ* 2014;348:g1687) which contains an explanation and elaboration for each item.

* The focus of TIDieR is on reporting details of the intervention elements (and where relevant, comparison elements) of a study. Other elements and methodological features of studies are covered by other reporting statements and checklists and have not been duplicated as part of the TIDieR checklist. When a **randomised trial** is being reported, the TIDieR checklist should be used in conjunction with the CONSORT statement (see www.consort-statement.org) as an extension of **Item 5 of the CONSORT 2010 Statement.** When a **clinical trial protocol** is being reported, the TIDieR checklist should be used in conjunction with the SPIRIT statement as an extension of **Item 11 of the SPIRIT 2013 Statement** (see www.spirit-statement.org). For alternate study designs, TIDieR can be used in conjunction with the appropriate checklist for that study design (see www.equator-network.org).

References:

Bartholomew Eldredge LK, Parcel GS, Kok G, Gottlieb NH, Fernandez ME. *Planning health promotion programs: an intervention mapping approach*. 4th ed. San Francisco: Jossey-Bass; 2016.

Bjornstad G, Wilkinson K, Cuffe-Fuller B, Fitzpatrick K, Borek A, Ukoumunne OC, Hawton A, Tarrant M, Berry V, Lloyd J, McDonald A. Healthy Parent Carers peer-led group-based health promotion intervention for parent carers of disabled children: protocol for a feasibility study using a parallel group randomised controlled trial design. *Pilot and Feasibility Studies*. 2019;5(1):1-3.

Bjornstad G, Cuffe-Fuller B, Ukoumunne OC, et al. Healthy Parent Carers: feasibility randomised controlled trial of a peer-led group-based health promotion intervention for parent carers of disabled children. *Pilot Feasibility Stud*. 2021;7(1):144.

Borek AJ, Abraham C, Smith JR, Greaves CJ, Tarrant M. A checklist to improve reporting of group-based behaviour-change interventions. *BMC Public Health*. 2015;15(1):963.

Borek AJ, McDonald B, Fredlund M, Bjornstad G, Logan S, Morris C. Healthy Parent Carers programme: development and feasibility of a novel group-based health-promotion intervention. *BMC Public Health*. 2018;18(1):270.
