# Supplementary material for: Healthy Parent Carers programme: mixed methods process evaluation and refinement of a health promotion intervention
Source: BMJ Open. 2021 Aug 24;11(8):e045570. doi: 10.1136/bmjopen-2020-045570 (PMC8388296; doi:10.1136/bmjopen-2020-045570)
Supplement: Supplementary data [file bmjopen-2020-045570supp003.pdf]

Facilitator training feedback form

During this phase of the research we are still developing and finalising the facilitator training. Your feedback will help us to ensure that any future training delivered is as comprehensive and useful as possible.

Your feedback is anonymous and you will not be identified in any research reports. This information will only be viewed by the research team.

1. Please rate, on a scale of 1-5, your knowledge and understanding, and skills and confidence in the following areas (with 1 being very little and 5 being a lot).

**Knowledge and understanding of:**	**1**	**2**	**3**	**4**	**5**
The intervention's theoretical framework (CLANGERS); the session activities and associated learning outcomes.					
Group facilitation techniques and how to create a positive group atmosphere.					
Particular challenges faced by parent carers and how this may impact on health and wellbeing.					
The role of the HPC Lead/Assistant Facilitator.					
The study design.					
Safeguarding procedures.					

**Skills and confidence**	**1**	**2**	**3**	**4**	**5**
Ability to confidently present programme information, lead activities and facilitate discussions.					
Ability to create a positive group atmosphere and inspire collective desire to make lifestyle changes to improve health and wellbeing.					
Ability to manage time effectively.					
Ability to manage difficult/sensitive issues that may arise.					

2. The time allotted for the training was sufficient (***Please tick one)***

**Strong Agree**	**Agree**	**Neutral**	**Disagree**	**Strongly Disagree**

3. Are there any aspects of the training that could be improved (e.g. content, time provided to cover the learning objectives and/or delivery)?

4. Any other comments?

Many thanks for taking the time to give your feedback.
