# Supplementary material for: Healthy Parent Carers programme: mixed methods process evaluation and refinement of a health promotion intervention
Source: BMJ Open. 2021 Aug 24;11(8):e045570. doi: 10.1136/bmjopen-2020-045570 (PMC8388296; doi:10.1136/bmjopen-2020-045570)
Supplement: Supplementary data [file bmjopen-2020-045570supp004.pdf]

Healthy
PARENT CARERS

CHECKLIST FOR MODULE 1 DELIVERY

Instructions to the Facilitators and Assistant Facilitators:
Please complete this checklist at the end of each session to provide us with feedback on what was covered and how the session went. This feedback will help us assess if the sessions can be delivered as planned and identify potential challenges and improvements.

Date & time of the session: ____

Session number: ________________________

Venue: ____

Facilitators' names: ____

Session duration (as delivered): _______________________

Number of participants attending: _____________________

**Activities**	**Delivered** (Please tick if delivered)	**Comment** (If partly or not delivered, please explain why)
1. Introductions / icebreaker activity		
2. Hopes and fears		
3. Ground rules		
4. What are health and wellbeing?		
5. What influences health and wellbeing?		
6. The resilience game		
7. Conclusion		
Time for informal interaction (e.g. tea break)		

Lloyd J, *et al. BMJ Open* 2022; 11:e045570. doi: 10.1136/bmjopen-2020-045570

Reflections on the session

Please indicate which picture best describes your perception of how well connected, in general, the attendees of today's session appeared to be (tick the box on the right of the picture).

For example, the first picture would suggest that there was very little connection between individuals – that they did not come together as a group at all. Whereas the final picture would suggest that individuals were very closely connected – they very much came together as a group.

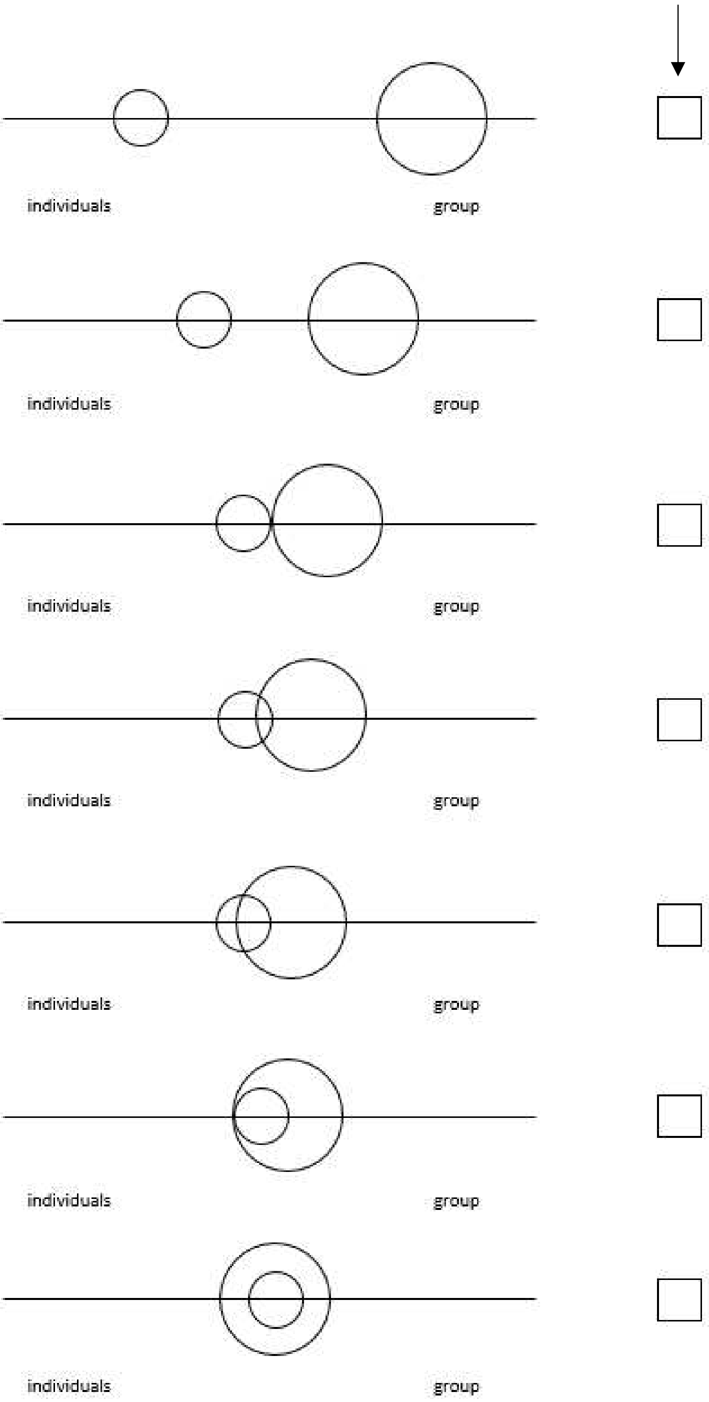

Lloyd J, *et al. BMJ Open* 2022; 11:e045570. doi: 10.1136/bmjopen-2020-045570

Reflections on the session cont.

Please circle one

In your opinion, how well did today's session go?

Not well at all				Very well
1	2	3	4	5

How engaged were the participants in today's session?

Not well at all				Very well
1	2	3	4	5

Do you have any comments or reflections?

(For example, related to how the group worked together, participants' engagement, session content or activities)

Lloyd J, *et al. BMJ Open* 2022; 11:e045570. doi: 10.1136/bmjopen-2020-045570
