# Supplementary material for: Healthy Parent Carers programme: mixed methods process evaluation and refinement of a health promotion intervention
Source: BMJ Open. 2021 Aug 24;11(8):e045570. doi: 10.1136/bmjopen-2020-045570 (PMC8388296; doi:10.1136/bmjopen-2020-045570)
Supplement: Supplementary data [file bmjopen-2020-045570supp005.pdf]

Healthy
PARENT CARERS

Draft topic guide for a focus group with HPC Facilitators

Topics and example questions for the focus group with HPC Facilitators and Assistant Facilitators:

- Motivation for / expectations of delivering the group programme, e.g.:
    - Why did you decide to get involved in this study?
    - What did you expect from taking part in the study / delivering the programme?
- Views about training / preparation to deliver the programme, e.g.:
    - How did you find the training to deliver the programme? (Prompt about whether it was sufficient, anything that would be helpful)
    - How prepared did you feel to deliver the programme? (Prompt about their background and previous experience, or any new skills developed by delivering HPC)
- Experience of delivering the programme / group facilitation, e.g.:
    - Having delivered the programme now, what do you think about it?
    - Generally, how did you find delivering the programme? (Prompt about following the Facilitator Manual, any adaptations made, challenges, how feasible it was to deliver)
    - How engaged was the group / the participants?
    - How did you find facilitating / managing the group? (Prompt about any challenges and how they managed those, how engaged they were in delivering it)
- What worked best in the programme? Why? (Prompt about activities, content, group)
- What didn’t work well? What were the challenges or difficulties? Why?
- Do you have any suggestions for improvements? (e.g. related to the programme delivery, content, Facilitator Manual, training)
- Is there anything else that we haven’t talked about and that you’d like to discuss?
