# Supplementary material for: Healthy Parent Carers programme: mixed methods process evaluation and refinement of a health promotion intervention
Source: BMJ Open. 2021 Aug 24;11(8):e045570. doi: 10.1136/bmjopen-2020-045570 (PMC8388296; doi:10.1136/bmjopen-2020-045570)
Supplement: Supplementary data [file bmjopen-2020-045570supp006.pdf]

**HEALTHY PARENT CARERS**
**WEEKLY PARTICIPANT FEEDBACK FORM FOR GROUP INTERVENTION**

**GROUP:** ______________________________________________

**SESSION NUMBER:** ______________________________________

We would like to know what you think about this session and would be grateful for your feedback. The feedback is anonymous and it will help us refine and improve the programme in future.

*Please circle one*

**1. Overall, how satisfied are you with today's session? (please circle one)**

Not satisfied at all — Very satisfied
1 2 3 4 5

**2. Do you feel that you were able to take an active part in the session? (please circle one)**

Not at all — Very much
1 2 3 4 5

**3. Please tell us about the best things** in today's session (e.g. most enjoyed or most helpful):

**4. Please tell us about the things that could be improved in today's session:**

**5. Which picture best describes how well connected, in general, you felt to the rest of the group today?**

For example, the first picture would suggest that you felt very little connection to the rest of the group, whereas the final picture would suggest that you felt very connected to the group.

**Please tick the box on the right hand side of the picture that best describes how you felt.**

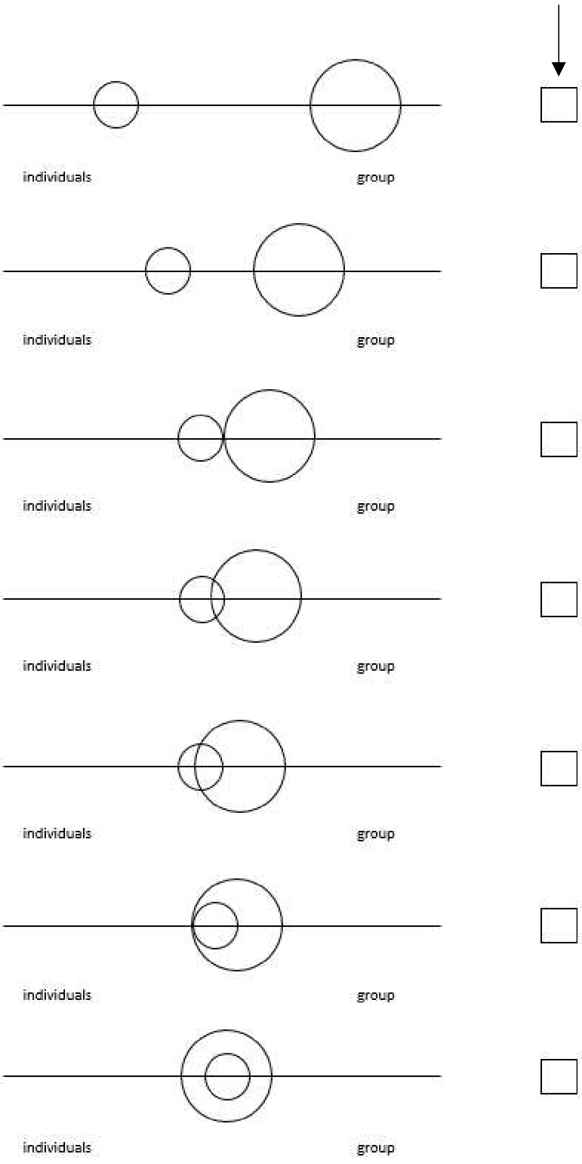


**6. How much did you identify with other members of this group? (please circle one)**

| Not at all | | | | Very much |
|---|---|---|---|---|
| 1 | 2 | 3 | 4 | 5 |
