# Supplementary material for: Healthy Parent Carers programme: mixed methods process evaluation and refinement of a health promotion intervention
Source: BMJ Open. 2021 Aug 24;11(8):e045570. doi: 10.1136/bmjopen-2020-045570 (PMC8388296; doi:10.1136/bmjopen-2020-045570)
Supplement: Supplementary data [file bmjopen-2020-045570supp007.pdf]

HEALTHY PARENT CARERS
Feedback FORM

We would like to know what you think about the Healthy Parent Carers programme and the research study and would be grateful for your feedback. The feedback is anonymous and it will help us refine and improve the programme and our research in future.

1. Overall, how satisfied are you with taking part in the Healthy Parent Carers programme?

Not satisfied at all				Very satisfied
1	2	3	4	5

2. How useful has the programme been in helping you improve your health and wellbeing?

Not at all				Very much
1	2	3	4	5

Was there anything in particular that helped you?

3. Have you made any changes as a result of taking part in the programme? No / Yes

If yes, what?

If no, why?

4. Approximately how many of the online materials have you accessed?

None	25%	50%	More than 50%

5. Did you attend group sessions for the programme? Yes / No

If yes, reveal questions 6-10, then continue from question 12.

If no, reveal question 11, then continue from question 12.

6. How would you rate the length of the individual sessions?

Too short	About right	Too short

7. Did you miss any of the group sessions? Yes / No

If yes, why?

8. Overall, how much did you feel included and part of the group?

Not at all				Very much
1	2	3	4	5

9. How satisfied were you with the way the sessions were facilitated and delivered?

Not satisfied at all				Very satisfied
1	2	3	4	5

10. Did you discuss the programme with anyone who had access to the online materials only (i.e. they did not attend the group sessions)? Yes / No

11. Did you discuss the programme with anyone who attended the group sessions? Yes / No

12. Do you have any suggestions about how the programme or materials could be improved?

13. Would you recommend this programme to other parent carers?

Not at all				Very much
1	2	3	4	5

14. Do you have any other comments, reflections or suggestions?
