# Supplementary material for: Healthy Parent Carers programme: mixed methods process evaluation and refinement of a health promotion intervention
Source: BMJ Open. 2021 Aug 24;11(8):e045570. doi: 10.1136/bmjopen-2020-045570 (PMC8388296; doi:10.1136/bmjopen-2020-045570)
Supplement: Supplementary data [file bmjopen-2020-045570supp008.pdf]

Appendix 2: Data collection materials

Follow-up questionnaire
Version 1, 11 June 2019

HEALTHY PARENT CARERS
Follow-up questionnaire

1. Have you continued to access the online resources in the past 6 months?

Yes / No

2. Approximately how many of the online materials have you accessed in total?

None	25%	50%	More than 50%

3. Did you attend group sessions for the programme? Yes / No

If yes, reveal question 4.

If no, end survey.

4. Since the Healthy Parent Carers group ended, how many members of your group have you been in touch with (including members that you knew prior to starting)?

(number up to 10)

5. Out of these, how many were people that you did not know before the programme?

(number up to 10)

6. Since the group ended, have you been in touch with any members the group that you didn't know before in any of the following ways?

(select all that apply)

Phone

Text

Email

WhatsApp

Other Social media (e.g. Facebook, Twitter)

Meeting in person

Other (please specify – please do not include anyone's name) (free text box needed if ticked)

Appendix 2: Data collection materials

Follow-up questionnaire
Version 1, 11 June 2019

I have not been in touch with any of the group members that I didn't know before

7. Approximately how long ago was the last time you had contact with any members of the group that you didn't know before it started (please do not include anyone's name)?

(free text)

8. Can you please describe how often you have been in touch with any members of the group that you didn't know before (please do not include anyone's name)?

(free text)

9. How many of these new contacts would you now say are your friends?

(number up to 10)

Lloyd J, *et al. BMJ Open* 2022; 11:e045570. doi: 10.1136/bmjopen-2020-045570
