# Supplementary material for: Healthy Parent Carers programme: mixed methods process evaluation and refinement of a health promotion intervention
Source: BMJ Open. 2021 Aug 24;11(8):e045570. doi: 10.1136/bmjopen-2020-045570 (PMC8388296; doi:10.1136/bmjopen-2020-045570)
Supplement: Supplementary data [file bmjopen-2020-045570supp009.pdf]

Draft interview topic guide: HPC Participants (intervention arm)

- Introduce myself (PenCRU childhood disability research unit)
- The aim of the interview is to get their feedback on the HPC programme and research study
- Confirm how much time interviewee has for interview. Confirm that meant to take about 30 mins but can vary depending on length of discussion – let me know if you have somewhere you need to be and we can manage accordingly
- Remind participant that taking part is voluntary – if I ask about something you don't want to talk about, please just say you'd rather not go in to that
- Remind participants that the interview will be recorded and transcribed and analysed to help us understand how the programme and study worked. Interview transcripts will be anonymous.
- Check participant is comfortable (has drink of water etc.) and has given consent to continue

Record before/after the interview:

- Participant ID:
- Participant's group ID & facilitators' IDs:
- Gender:
- Age:

Topics and example questions for participants in the intervention arm:

- Motivation to participate & expectations, e.g.:
  - Why did you decide to take part in the study / programme?
  - Was there anything that helped you to decide to take part?
  - What did you expect from taking part in the study?
- Understanding of, and views on, the study design, e.g.:
  - How sufficient did you find the information about the study & the programme and what they involve?
  - What did you think when you found out that you were assigned to the group programme, rather than only being given access to online materials?
  - Would you change any aspect of how it was explained to you, or what information was available, about the programme or the research? (probe suggestions from them)
- Experience of the group programme (activities, group):
  - What do you think about the content of the programme? (prompt about relevance "relevant to you as a parent carer", helpfulness of the content e.g. CLANGERS)
- Views on length & frequency of sessions; any missed sessions & reasons for missing sessions
  - What do you think about the length and frequency of the sessions? Did you need to miss any sessions? (prompt about reasons for missing sessions)
- Views on & engagement with the group:
  - Tell me about your group. Did you feel included and a part of it?
- Views about facilitators and delivery style, e.g.:

  - What do you think about the facilitators and the way they delivered the programme? How well did they do in delivering the programme and facilitating the group? (prompt about facilitator engagement)
- Usage of, and views on, the online programme materials
  - Did you access the programme materials online? (prompt about how they used them/how often, or about reasons for not using them) If yes, what prompted you to look at them?
- Impact of the programme & any changes made, e.g.:
  - How helpful did you find the programme? Did you make any changes in result of the programme? (prompt for examples) Was there anything that got in the way of you being able to make those changes? Was there anything that supported you to make those changes?
- Contextual influences, e.g.:
  - Did anything affect how much you got out of participating in the programme? Were there any barriers to you engaging with or benefiting from the programme?
- Best & worst things, suggestions for improvements, e.g.:
  - What did you like most / found most helpful (if anything) about the programme?
  - What did you not like / did not find helpful (if anything)?
  - Do you have any suggestions for how the programme could be improved? (prompts: delivery, content, group, timing / length of programme)
- Acceptability of data collection and measures, e.g.:
  - How did you find having to fill in all the questionnaires at the beginning and at the end of the study? (prompt about time, clarity, relevance)
  - The sessions were audio recorded. How would you have felt if the sessions had been video recorded instead?
- Is there anything else that we haven't talked about and that you'd like to mention?
