# Supplementary material for: Healthy Parent Carers programme: mixed methods process evaluation and refinement of a health promotion intervention
Source: BMJ Open. 2021 Aug 24;11(8):e045570. doi: 10.1136/bmjopen-2020-045570 (PMC8388296; doi:10.1136/bmjopen-2020-045570)
Supplement: Supplementary data [file bmjopen-2020-045570supp010.pdf]

Healthy PARENT CARERS

Draft interview topic guide: HPC Participants (control arm)

- Introduce myself (PenCRU childhood disability research unit)
- The aim of the interview is to get their feedback on the HPC programme and research study
- Confirm how much time interviewee has for interview. Confirm that meant to take about 30 mins but can vary depending on length of discussion – let me know if you have somewhere you need to be and we can manage accordingly
- Remind participant that taking part is voluntary – if I ask about something you don't want to talk about, please just say you'd rather not go in to that
- Remind participants that the interview will be recorded and transcribed and analysed to help us understand how the programme and study worked. Interview transcripts will be anonymous.
- Check participant is comfortable (has drink of water etc.) and has given consent to continue

Record before/after the interview:

- Participant ID:
- Gender:
- Age:

Topics and example questions for participants in the control arm:

- Motivation / expectations to participate, e.g.:
    - Why did you decide to take part in the study / programme?
    - Was there anything that helped you to decide to take part?
    - What did you expect from taking part in the study?
- Understanding of, and views on, the randomised controlled design and control group, e.g.:
    - How sufficient did you find the information about the study and the programme, and what they involve?
    - What did you think when you found out that you were assigned to the group with access to online materials only and not the group programme?
    - Would you change any aspect of how it was explained to you, or what information was available, about the programme or the research?
- Contamination with the intervention arm, e.g.:
    - Do you know anyone who also took part in the study and attended a group? (If yes, prompt about how much they discussed what happened in the group and how much that might have influenced their view on the programme materials or changes made).
- Usage of, and views on, the online programme materials, e.g.:
    - Did you access the programme materials online? (prompt about how they used them/how often, or about reasons for not using them)
    - What do you think about them?
- Impact of the programme, any changes made, e.g.:

  - How helpful did you find the materials? Did you make any changes in result? (examples) Was there anything that got in the way of you being able to make those changes? Was there anything that supported you to make those changes?
- Views on the data collection and measures
  - How did you find having to fill in all the questionnaires at the beginning and at the end of the study? (prompt about time, clarity, relevance)
- Best & worst things, suggestions for improvements, e.g.:
  - What did you like most / found most helpful (if anything) about the programme?
  - What did you not like / did not find helpful (if anything)?
  - Do you have any suggestions for how the materials or the research elements could be improved?

Is there anything else that we haven't talked about and that you'd like to mention?
