# Supplementary material for: Healthy Parent Carers programme: mixed methods process evaluation and refinement of a health promotion intervention
Source: BMJ Open. 2021 Aug 24;11(8):e045570. doi: 10.1136/bmjopen-2020-045570 (PMC8388296; doi:10.1136/bmjopen-2020-045570)
Supplement: Supplementary data [file bmjopen-2020-045570supp011.pdf]

**Supplementary Document 11. Additional quotes and comments**

# 1. Facilitators' experiences

## *1.1. Motivations and expectations*

New experiences, new skills & being involved in the research project. (Facilitator pre-training questionnaire)

Recognised the need for healthy wellbeing of parent carers through myself and friends. Facilitate a small support group locally. Ready for a new challenge. (Facilitator pre-training questionnaire)

To apply my training delivery skills and experience to a programme which is unique in its focus on parent carer health, something myself as a parent carer feel I could directly benefit from. (Facilitator pre-training questionnaire)

CLANGERS has changed my life and I feel it is really important to help others by spreading the message. It's also a research project and that excites me as well! (Facilitator pre-training questionnaire)

I always believed in this course because it had such a positive impact on me and I just wanted to give back to others. (AF, focus group)

When we did the [training], I do remember thinking some of this is really resonating with me and my own challenges and thinking actually, I can see where this is going, and it has been really, really good. (LF, focus group)

## *1.2. Training, preparation and support*

It became clear to me that there was actually quite a distinctive style that was expected as well… we would all have liked some more concrete direction in terms of what to do and how. (AF, focus group)

It's something actually a little bit more fundamental about that. It's about why are you writing this stuff down? Because there's nothing in the trainer notes that explained to the trainer what the purpose of having this stuff written down is. I always find it difficult delivering training that's been written by somebody else… I had to keep saying in my head, 'do it as it's written, don't go off and do your own thing'. (LF, focus group)

I would [practise] 'Connect' because it would give you the opportunity to identify some of the issues that are going to come up, because 'connect' can be really difficult for parent carers. So you could use it as, well this is an example of somebody who struggles, how would you do it in this situation, how would you cope with this? So it can be quite an emotional one and it's also the first one that we do in the programme… The group were also very nervous and quiet in that one, so that makes it harder. (AF, focus group)

LF: …trainers probably need to undergo the training in the first instance because I think you need to experience the programme, both to have a better understanding but you would get a lot of ideas and facilitation through that.

AF: I felt I was at an advantage because I had done it… because I knew the course I was able to listen and scribe, knew what was coming up because I'd done it before, but I was also able to input in my way and from my experiences… which I felt helped. So it wasn't new information to me, I'd already gone through that quite hard process of thinking and I had already adjusted and adapted and was aware of it and everything else. And you could see it on the faces of the participants that actually it was hard at times and I had been there, so I understood. (Focus group)

More time for assistants to get a clearer understanding of breath of exercises / module content. (Assistant training, feedback form)

It's all very hurried. More time practicing our role. (Refresher training, feedback form)

### *1.3. Programme content and group delivery*

It's tricky to manage but more difficult with some groups than others, definitely… but I think for the most part parents actually said it was really nice to focus on themselves. (LF, focus group)

LF: I would be very tempted to remove SMART from there and just replace it with achievable.
AF: The most important thing about CLANGERS is that you take ownership of it and you become aware that that's what you're doing. So it's important to have those goals but it's also important to recognise when you're not achieving those goals, where did it fall down. (…) so actually you might not have achieved that goal but you've actually done bits of CLANGER here instead. So it's just raising that awareness and your own understanding and building up that way. (Focus group)

The fact that we had such small groups was really disappointing for me… I think it changed the nature of the course, being such small groups, it was far more intense, I believe, for the training team, than it would have been if we'd had lots of participants there who were able to share their views and other things. So yes, altogether a highly enjoyable and positive experience but a few things that, perhaps, my expectations were slightly different. (LF, focus group)

I think the key of the leaders being parents of children with disabilities just really, really helps everybody to bond and understand. For me, I'm like wow, you've been through it, you're this much further on the same journey that I'm on and look at you; you're out here and you're doing this and you're working and, oh, your life is okay… That's reassuring. (AF, focus group)

All participants were very engaged. Unfortunately, one has a lot of problems currently so found it difficult to discuss resilience without being extremely emotional. Other participants very supportive. (Facilitators session checklist)

Group extremely connected. Tearful at the end of evening. Have made arrangements for continued contact as a group. Great sharing + reflection of course. (Facilitator checklist at the end of the final session)

## 2. Participants' experiences and views

### *2.1. Motivation to participate and expectations*

*Wanting to improve health and wellbeing:*

I feel that being a parent-carer is really draining on your mental health and also I wanted to try and get healthy anyway, especially with it being January. Yes, I just thought it would be a good thing to try out and see if it does help me. (…) a bit of knowledge maybe about the way I feel and maybe a bit less guilt, the fact that I feel that I need to take time out for myself. (P13, control)

I was aware that my own health, fitness, mental health. All of those things really have taken quite a hit over the years, so it was a bit of a chance to actually focus on that for probably the first time in a long time really. So that was my main motivation. (…) It just seemed to fall into my lap at the right moment, so I, yes, sort of grabbed the opportunity with both hands really. (P3, intervention)

We decided to take part in the study because we wanted to be proactive as we are on most things. We knew that we needed to help ourselves as carers because we've become carers abruptly and inadvertently. (…) We've been using all the materials together [with his wife]. And we wanted to take part because we aren't going to sit on our arses and wait for something to happen because what we've learnt is the public sector will do nothing for you unless you poke it very hard. (P14, control)

I just thought it could be useful because a lot of the time I find as a parent carer all the focus is on the children which is obviously the main thing but the parents need to be healthy and happy to give the children the best. (P8, intervention)

*Expecting advice, resources and signposting:*

I was hoping just to find some resources to help me turn the attention off my daughter and more onto myself, just learn how to look after myself in amongst the very challenging times that we have looking after my daughter. (P15, control)

It was sort of different to what I expected because, like I say, I was expecting more sort of direct, "Right, this is who you can go to," rather than sort of what seemed like giving people information that they probably already knew. (P7, intervention)

*Good timing of the programme:*

If you wanted me to do the course at the beginning of last year I wouldn't have been able to do it because my daughter was very ill, but now she's in a good place at the moment so I could do it. (…) I've got to that point in my life that I have to say right, [my daughter] will always come first, she has to, and my husband and the rest of the children and grandchildren. But sometimes the work you do has to change, it has to go in a different direction (…) I've just always got on with it, but I think now I've got to that point that I think right, things have to change. (P11, intervention)

I just thought that it just fitted with the times in my life that I wanted to try and get a bit healthier and a bit less stressed. (P13, control)

Probably the fact that it was on Monday night. I've got very few timeslots that are regularly available and that one happens to be free, so. I think that probably was the biggest thing that made me go, yes let's just do it. (P2, intervention)

*Wanting to contribute to research:*

I thought it would be really helpful for me and I just felt it is quite important research, because I can't say I've looked up if there is research in this area, but I don't think there is a lot of research and I don't think unless you are in the situation, no one really gets the impact on your life and your health and everything else, it just takes over. So it just felt important for me to do for myself and also part of a bigger picture really because I think they are many many people in this situation and I don't think there's much light shed on it. I think it's getting better. But it just felt like an important thing to be part of really. (P3, intervention)

Well I thought it would help me with day-to-day stuff, but it's also an area that I have looked for help with in the past and found that there isn't any specifically, mental and physical health specific to parent carers, so I wanted to contribute to it because I want it to work [laughs], I want it to be there for when people need it. (P6, intervention)

*Preference for group programme:*

I was just excited to be involved in something and potentially going to be around other people… (…) I was hoping to meet other people who are in a similar situation to me and I think that you can learn a lot that way and also it's a support, meeting other people. Particularly for me, I'm very isolated and I'm assuming that there are other parent carers that are the same, that you can become very isolated and also quite often friends and family don't really understand, they say they do but you know from their actions and the things that they say sometimes that they don't really understand. So you can feel quite lonely. So having the opportunity to know that I'm going to be with other people who are in a similar situation, I was really pleased about. (P4, intervention)

I just thought that was going to be a lot more likely to be, well, something I would see through because there's a kind of commitment to turning up each week and I'm dreadful for just dropping out of things and going, oh whatever, I'm too busy… (P2, intervention)

I was a bit gutted really because I know that I always struggle with things on computers. It's not because I struggle with computers, but I always put it off to the next day and the next day, whereas if it was a group, I would just go to it as if it was an appointment. (P13, control)

I would have liked to have been part of a group to be able to discuss it with as a parent going to do similar things. (…) If there was to be a Healthy Parent workshop here in [town], I would have done it, I would have been able to find a way and I think I probably would have got more out of it from just sharing experiences and getting to know other people just for networking and support for each other. (P15, control)

*Preference for, or acceptance of, online-only programme:*

I wouldn't have been able to attend the groups anyway. I'm working 12-hour days, so it would have been a real rush. If you'd assigned me to the group, I probably would have asked to either drop out or go online anyway. So it worked out well in the end. (P14, control)

I was disappointed [when found out about being assigned to the group programme] because I was quite looking forward to doing the online material where I didn't have to go out, see people and talk to people, but again that is just my personal preference. It is perfect because it pushed me out of my comfort zone. I was quite apprehensive on the first day. (P9, intervention)

*Feeling nervous before the first session:*

It took a lot of self-will to take myself to the first session. It was one of those where when it came to it I was really excited and getting ready for it, then when it actually came to it I suddenly was trying to find excuses. (…) It was the sheer fact that just as it was about to start, everything around me became 10 times busier. (…) I think that was my main excuse for why I was allowing apprehension to take over. I think it is always that self-doubt factor of, is it going to be any use to me? Am I going to have to talk to loads of people and I don't really want to? It was a combination of those factors I think. (P9, intervention)

### 2.2. Experiences of the programme and its impact (and mechanisms)

*Positive experiences:*

It was fantastic. I think it was really well done. (…) I think it's changed my life. I really do. I haven't stopped talking about it since. Yes. It just makes complete sense. (…) I think the best thing was going to the group, meeting everybody in the group, just meeting like-minded people, having a break from life and being in a different place for a while, reflecting on aspects of my life that I didn't really think about or had put away, and I learnt more about myself. (P5, intervention)

I thought it was brilliant. It really has opened my eyes on an awful lot of things and I have changed an awful lot in my life. (P10, intervention)

The impact of a course like that, if that keeps me going so that I can look after my daughter, if it helps me figure out, work out our stresses in our family, keeps my relationship together, that has not just impact for our family obviously but wider in terms of taxpayers' money. So for me, I think it's really important. (P4, intervention)

I suppose they made you feel uncomfortable, which once you have got over the feeling uncomfortable about the fact that you have analysed something and thought oh I don't really like that and you can kind of box it but you think well actually no I need to address that. But that's just more about taking you out of your comfort zone really. But that's not really a bad thing, that's just an uncomfortable thing. (P1, intervention)

Lots of things! Meeting people who understand and 'get it ' reduced my isolation. Having parent carers as facilitators helped a lot. Having 12 structured sessions made me commit to it and focus on my own health. Having structured fun informative sessions helped a lot - I definitely went through a process of change. The use of humour was really important to me - and learning to be more resilient. (End-of-programme feedback)

Learning how useful and how important it is to keep myself healthy, not only for myself but for my child family and friends. Learning how to reflect and realising the amount I already do for myself and others that I am now proud of achieving a good sense of self-worth. Using Smart goals. (End-of-programme feedback)

I just think it was excellent and totally life changing!! Every parent carer in the UK and worldwide should have access to this course!! Thank you so so so much!! (End-of-programme feedback)

*The group:*

Being part of a group feeling like my challenges were understood and acknowledged. We laughed a lot cried sometimes and developed a lovely supportive friendship. I felt that the course was a really good balance of talking to off-load whilst also being 'solution focussed'. This was very empowering. Whilst my situation remains very challenging, I feel I have the skills and knowledge to help myself whilst also now having a wonderful new group of supportive friends. I am so grateful for this experience. Thank you. (End-of-programme feedback)

Our group worked really well actually and it really evolved, which was lovely because there were people who were thinking they weren't going to make it through to the end and they did. (…) We had quite an empathetic group and there was sort of, now that I'm thinking about it, possibly an unwritten code. (…) There was a lot of respect in the group. Obviously, there's some people whose kids have mental health issues and having major issues. There was a lot of well let's just sit and make sure we listen. There was a lot of support and everybody injected into the study, nobody sort of sat back, or if they were uncomfortable they said they were. As a group, it did, it evolved and it moulded and it's a really nice group. (P1, intervention)

I found it all helpful. I think the… and again, because it was a group, and that's why I would say I'm really glad that I was on the group side of it, the interaction and people's knowledge and people's just experiences and you kind of… but that sort of camaraderie type of feeling really, that's probably what I got more… probably, and then, yes, you put that down and the connecting that we did really. There's lots and lots of connecting really and… Yes, there was lots and lots of all of it actually, thinking about it, but just people putting through ideas of, "Well I do this, I don't know if that would work for you". There was a lot of that and it was, yes, that's probably what I got mostly from it. Yes. (P1, intervention)

It was a really nice group of people, everyone was very… I'd say everyone was very open and honest, people were able to talk about quite difficult circumstances and situations and it felt like a supportive environment and people were able to get upset and feel supported I think and also we did have a really good laugh, which was brilliant. I think we were really lucky. We are planning to keep in touch. Typical carers, no one can find the time when we are all available. It just sums it up really, doesn't it? But it was such a positive experience. After the first night I came out on a bit of a high because it's just always so… because friends and family and they care, but people just don't get it in the same way as when you are with a group of people who just know how bleak it can be, yes. (P3, intervention)

What I loved about the course was that it was very solution focused and I think quite often you can go to groups and things that it's just about talking, and as helpful as that is, you still come away with it going, well I'm not really sure… It was nice, but I don't really know how to make things better still, so to go to a group that's like you have the opportunity to talk and to gain help from other people and be listened to, great, but let's put that to one side, what are the barriers and how can we overcome them and let's work together to help each other overcome our barriers to having a more healthy and greater wellbeing in our lives. So I think if I'd have known that beforehand, I would have been even more excited. So maybe to have known that that was what it was trying to achieve. (P4, intervention)

I liked being around other people who were in a similar position to me. I liked the fact that we could listen to each other and offer support and help and useful advice. There was a lot of that that went on between us. Thinking creatively about how… from everybody's own experiences, what knowledge do I have that can help you? So I liked the supportiveness of the group. It wasn't all serious either, we laughed a lot, and that was lovely to have those moments where we shared silly jokes. It really was a case of we laughed together and we cried together, do you know what I mean, and we helped each other. So it was a really lovely group to be in and I really liked that. (P4, intervention)

We all got to share our stories, we all got to know each other, we all gave each other presents at the end and had a little party. It was really good. I think we all really became different people throughout the course. (…) I think the best thing was going to the group, meeting everybody in the group, just meeting like-minded people, having a break from life and being in a different place for a while, reflecting on aspects of my life that I didn't really think about or had put away, and I learnt more about myself. (P5, intervention)

When somebody was missing it kind of really felt like somebody was missing because we all had very different perspectives and different situations and it was really interesting. The biggest part of the thing for me was not what came out of the structure but out of the conversations that we had between us. Everybody interacted equally with everybody else across the six weeks, so there wasn't like two people who got on really well and had lots more in common and went off and talked between themselves, it was like completely among the group and it wasn't… In some weeks one of us might be quiet for our own reasons and that was fine. Everybody was very… I don't know, sort of accepting of other people's contribution, whether they were contributing a lot or not very much that week. (P6, intervention)

Everybody was listening and it was nice… near the end of the… the latter sessions, we were very much… if you understand what I mean, as a group, we were able to have little jokes together and bring up things that we discussed before. It was very nice. It was very much a camaraderie if you know what I mean. It was really nice and everybody was nice to everybody. There was not any anger or frustration. You could see people were struggling with certain things. We were able to advise them 'Actually, you are better than what you think' so it was really nice. (P12, intervention)

*Group size:*
It was smaller than I expected. I was expecting a group twice the size. And in some ways that might have been better because it might have been easier to find maybe subgroups within that that I could get… because there were definitely individuals in that group I could definitely get along with better but instead because it was very small it felt quite kind of intense which then meant that, yes, each person ends up trying to make it large and that… yes, and in a way that almost makes it harder to share. Yes, I don't know. I think I probably would have preferred a slightly larger group actually, but not massive. (P2, intervention)

There was only three of us, which was kind of a shame there wasn't more but, at the same time, we couldn't have got as much out of it as we did because we all got to share our stories, we all got to know each other, we all gave each other presents at the end and had a little party. (P5, intervention)

I think if the group was a little bit bigger, maybe not massively bigger but maybe, say, nine to ten people, I think you would get more input and there would be more discussion because even though

we were able to discuss things, I think a bit more of an open, broader amount of different people with different lives would make it a lot more interesting. (P12, intervention)

*Peer facilitators:*

We were sometimes a bit naughty on time when we talked too much, but I think that just happens when you are in that group. But yes, we managed to cover everything. There was ample time. They made sure everyone was included and had a say at every step really. I don't think there was anyone who didn't feel they could contribute or felt left out. (P3, intervention)

[Facilitators were] Brilliant, brilliant. And they were bouncing off each other. It was not just coming from one person. Because sometimes, you know when they do these things and you are only getting it from one perspective and [name] was able to challenge that perspective and then we were all able to challenge it and it was nice to be able to share rather than actually going, 'We are learning off a book that they were using to get their information' but actually, we were having to break it up even more on the sheets in front of us and where they were writing it all down, we were able to take notes and everything, so it was really good. (P12, intervention)

They really kept things going. They both had quite different approaches which I think complemented each other. Yes, it just seemed to work really well as a complete group including the facilitators really. (P3, intervention)

I think it makes a massive difference that they were both parent carers because they obviously have that innate understanding. I think it would have been different if they hadn't been, if the facilitators hadn't experienced some of those issues, I think it would have been very different. So I think that was a massive bonus. Because it's almost like a short hand, isn't it, there's things you don't even have to explain or go into because you just know that there's an understanding there already. So I think that was really helpful. (P3, intervention)

I think that's really helpful and important because it's not… Both of them are parent carers themselves and have been through very difficult situations and were open about sharing some of their challenges and their experiences and I think that that helps you then feel that it's okay to express what you are going through and that it's understood, because I think in those situations where it's being delivered by somebody who is reading the material without having had any of those challenges themselves, makes it less believable, less… like going to a dieting class that's being led by someone who has never had any problems, challenges with their weight, do you know what I mean? (…) It makes it less believable and somebody preaching to you but coming from a place that they have no experience of but they are preaching to you about how you can make changes in your life. And I think for me then I go I'm not sure if I can… It makes me not want to… It's less genuine, I guess. So for me having people facilitate the programme who have both had those challenges and, as I said, were quite open about their own challenges, and each week when we were going through the different clangers or whatever, they were honest at times, saying, "Well I've struggled this week to do it," and so it makes you feel it's okay that I'm struggling with this as well. And we all kind of worked together to help each other and encourage each other and that was a really lovely thing. Really lovely thing. (P4, intervention)

The facilitators of the programme were excellent in that they helped to create an environment where, as I said, it felt safe to talk about these things, that you were understood, that they cared about what you were going through and that very clearly came across from both of the facilitators, that they genuinely cared about what we were all going through and our challenges and also that they were honest about their own challenges. (P4, intervention)

The facilitators were very experienced (…) [they] were really knowledgeable, made us feel very relaxed, and I think that was a great part of it as well. (P11, intervention)

[Facilitator] was great, really good and whist she was delivering a programme she was absolutely excellent at tuning into individual people's situations and responses to things without singling them out. I could see she was being very empathetic and helping different people to get through different bits of it individually but as a group, if that makes sense, which I think was a real skill. She wasn't just delivering a programme, she was also doing it as individuals and again I think being a very small group really helped that. [Facilitator 2], yes, she was lovely. I mean, she was very good, kind. (P6, intervention)

They were lovely. Both of them were fantastic. I cried at times and they were caring, they were welcoming when you got there, they understood and remembered things. You felt like you got to know them and they got to know you and they'd talk about their experiences as well. I couldn't fault them at all, either of them, they were both fantastic. (P8, intervention)

This was doable because the facilitators weren't just 'professionals' preaching at us. (End-of-programme feedback)

The 2 tutors delivering the course were absolutely amazing! Being parent carers themselves really made them part of the group and not just 'teachers'. They were both friendly, welcoming, caring and informative. They made the course something to look forward to each week. (End-of-programme feedback)

Having the course delivered by another parent carer helped you to feel more at ease with disclosing information about your life and to someone who could totally relate and understand you circumstances. (End-of-programme feedback)

*Content and activities:*
The content of the course was good, it was really helpful and I know that it's going to help me going forward and I know that I am going to keep referring back to that to help me. (P4, intervention)

I think on every feedback form after every week it was like what was good about it and I was like discussion and sharing [laughs] which didn't come directly from the content of the programme but it made that happen. So that for me was the biggest thing because those are the things that stick, someone else's experience or if somebody else is maybe struggling with something, you'd support each other and you would kind of remember those things, you remember the personal stuff, human interaction that you have with people, whichever way, more than stuff you have been told. So I thought that was great. (…) It just seemed like a very natural way with that structure and the structure almost kind of… kind of like disappeared a bit into the background. I mean, sometimes to keep things moving, [facilitator] would have to bring us back to it and move on and stop one thing and start another thing, but it was almost like that became like a supporting thing, so the structure supported what happened in the session, it didn't drive what happened in the session, although it gave it structure but the session didn't hang on the content. (P6, intervention)

I think it would be relevant to everybody actually. I think everyone could probably do with doing that course. I think as parent carers we have additional challenges, obviously certainly lots of the stress and the anxiety associated with your children who are struggling, but I thought that the content

really helped you to focus on how do I keep myself healthy and look after my wellbeing, because if I fall down then I can't support my children. And I think that's really really important because I think so often families don't get support until it's too late. (P4, intervention)

I think it's relevant to all human beings [laughs], but I think putting it in the context of being a parent carer, yes I did find it very helpful and yes they were all the things. I don't think there was anything in there that was kind of superfluous and I don't think there was anything in there that was, I don't know. Yes, just when you blend them, when you blend the CLANGERS, they cover everything in life really. So parent carers, I didn't feel there was anything there, they were all things that were accessible. So there weren't things in there like for a lot of parent carers would feel like a non-starter, overwhelming, unachievable. They were all sort of day-to-day things that we all need to look at. Yes, I think they absolutely covered the typical things. Not to say that other people don't struggle with them but that parent carers particularly are more likely to struggle with more than the average person. (P6, intervention)

Learning about the CLANGERS and realistic ways to apply them - small steps. Having a laugh. (Weekly feedback, module 2)

Everything today I could relate to, enjoyed the videos, very informative and has made me look at how I can change to help improve my life. (Weekly feedback, module 3)

Looking at achievability of fitting in more exercise / active stuff. Looking for opportunities rather than barriers. (Weekly feedback, module 5)

Really enjoyed doing a 'top tips' exercise. It was a nice reminder of the CLANGERS. Revisiting the 'wheel' to see how far I have come. Very positive for me. (Weekly feedback, module 12)

Having 12 structured sessions made me commit to it and focus on my own health. Having structured, fun, informative, sessions helped a lot - I definitely went through a process of change. (End-of-the-programme feedback)

Loved the CLANGERS definitely made me think about the areas of my own wellbeing I need to work on. (End-of-the-programme feedback)

Reading the information made me think more about things I can do to help myself feel better. I even completed a cross stitch for the learning something new part. The CLANGERS acted as prompts to get me thinking about things I needed to do to give myself some time for myself. (End-of-the-programme feedback)

Being able to reflect on what's working and what isn't by going through the resources and questionnaire in a structured way. (End-of-the-programme feedback)

*Being reminded and prompted:*
I just am aware of… perhaps more aware of what's wrong, but I don't know that I've made, yes, any great changes to anything. I mean, I went away and said I'd do various things, and some of them I think were okay. I suppose it gives me more of a picture of what doing well might look like. (…) I'd have made them before if I could. I wasn't unaware… (P2, intervention)

It gave me a big kick up the bum to do something, I think that's been helpful. (P13, control)

A lot of it is very common sense stuff… you don't think about or focus on things enough. The CLANGERS, I've discussed that with quite a few people, because it's so simple, isn't it, but it really did help me to focus on yes just all of those things really. (…) It's like a revelation without it being a revelation, if you know what I mean, because you know all that stuff, but you don't take the time to think about it and it was just very much about focusing on us and improving things for ourselves before we can do it for everyone else really. (P3, intervention)

It was all about trying to make sure we reminded ourselves what we already knew but take the time to try and make sure we can sustain ourselves because sometimes you just get really tired, physically and emotionally. (P14, control)

It's not like a six-week programme where you go in not knowing something at the beginning and you come out knowing something at the end or being able to do something at the end that you didn't already know in your heart. It's a six-week programme that concentrates on reminding you of things that you can do and having had that reminder in a concentrated way it's with me all the time. (P6, intervention)

The CLANGERS acted as prompts to get me thinking about things I needed to do to give myself some time for myself. Being able to reflect on what's working and what isn't by going through the resources and questionnaire in a structured way. (End-of-programme feedback)

*Reflecting and setting goals:*
I think acceptance is a big thing, so accepting what you can't change. And we talked a lot about that on the course, looking at what are our challenges. (…) So it's kind of separating what you can and can't change I think helps you make steps forward, so going, okay, well this is the way it is, I can't do anything about that, so I'm just going to accept it, and now okay what can I change, what do I have control over and then breaking that down again into what are the specific barriers to me making those changes and working on those. (P4, intervention)

I think what causes the stress and anxiety and things like that for me and kind of the downward spiral that you can get into is that feeling that you don't have control, that everything is happening to you and you can't take control of it (…) So you can end up feeling like you don't have control over anything in your life anymore and that's when things start to spiral. So to stop for a minute and go, okay, well let's just think about my health and wellbeing and to break it down in a way that you can go, okay, this is what I know I need to do to be healthy and for my wellbeing for me to carry on doing this, but often everything can feel so overwhelming that you don't know where to start. So to be able to break it down like that and then go, right, I know I need to do this for my wellbeing, what are my barriers, what's stopping me from doing it, and then to analyse that and go, okay, well how can I realistically overcome that to make a positive change gives you… makes you feel like you have more control again. It brings back some control into being able to help yourself, which I think is really important, is having the tools and the skills to be able to help yourself, which is what I felt that course gave me. (P4, intervention)

It taught me how to break down those things into manageable chunks and to know that even a small change is good, that it doesn't… because I think so often we get caught up in the fact that it's all or nothing, right? You have to make that change and you have to do it all the time otherwise it's not worth it, and I think it's recognising that even small changes can make a big difference and being

able to work out how to make those changes, as I said, by breaking it down and looking at what my barriers are. (P4, intervention)

The message was you don't have to make massive changes to improve things. So I'm just trying to, for me and the kids really, tweak things a little bit to try and improve things a bit so it doesn't feel like an onerous task and I don't have to go to a class, I can sort of fit in activity as and when. That's quite reassuring really. So it's about making little changes that you can achieve rather than setting massive goals that you are just going to fail and then feel like a failure. (P3, intervention)

When you do the diary, ticking those and understanding what each planner meant it was suddenly occurring to me that I didn't eat very well, I didn't relax, I didn't sleep, so I needed to focus more on that side of my life. That really helped – the tick chart and setting goals. (…) What might stop you was literally time, so I know I needed to make adjustments. (P10, intervention)

*Focusing on oneself:*

You know all that stuff, but you don't take the time to think about it and it was just very much about focusing on us and improving things for ourselves before we can do it for everyone else really. (…) it has made me focus much more on my health and wellbeing and thinking I've just got to give myself that time. There's loads of things that I need to do, but at the minute I just need to get better. (P3, intervention

To be honest, it is all basic stuff, isn't it? It is just bringing it to the forefront, that actually, you are a worthy person and it is important that you do something for you, as well as your family and everybody else. (P18, control)

It is about making the carer feel that they are just as important as the caree. Because if we do not focus on ourselves, which is something we learnt, then you cannot work well for the person that you are caring for. (P12, intervention)

I think because of the way it was designed as well, we found out more about the people ourselves. We were ourselves in that. Even though we were carers and we were there because we were carers, we were talking about how we were. (P1, intervention)

*Less positive experiences, limited impact:*

The thing is, I found it helpful and I know I will continue to find it helpful. I can't say anything has changed as a direct result. Well, maybe some things have changed as a direct result of the programme but because I had an injury and because I'm just tired all the time, it's sort of like well that hasn't really changed [laughs] and it's not going to change. I think it gave me more hope that I will use… It's not like a six-week programme where you go in not knowing something at the beginning and you come out knowing something at the end or being able to do something at the end that you didn't already know in your heart. It's a six-week programme that concentrates on reminding you of things that you can do and having had that reminder in a concentrated way it's with me all the time. So as an example, I'm in a fairly poor state at the moment, not in life, nothing terrible but I'm really tired and struggling with this damn finger and feeling a bit like I'll never get things back to normal, but I know I will and I know what things I'm going to do to do that and the CLANGERS thing just… yes, it just stuck with me and I'm not speaking for other people but I could say that it did for the group, the other participants. It didn't make a massive short-term difference in my life. It did make a massive difference in my

thinking and my awareness and hope that [laughs]… I think it's hope. It gives you hope that you can get back on track no matter what, I'd say. (P6, intervention)

It was interesting, it showed me lots of aspects that I could look at. It wasn't just one thing to change, there were lots of things and one of the videos stuck in my mind. I keep thinking of that, but I haven't actually motivated myself to do anything about that just half an hour of your day. There's like 24 hours in the day and it's just like all we are asking for is half an hour to actually go and do some exercise. So that was the most beneficial that you could do to try and lift your wellbeing and your health and everything, I thought yeah, yeah. I'd got it in my head, but I still hadn't done anything which is a bit… I feel a bit annoyed with myself that I haven't. I thought that in my head all the time why don't I just do that half hour? (P16, control)

I think I probably would have got more if there had been more time for chatting because that was pretty much banned and the time was very limited because of the research element of it, whereas I think in a group like that people would chat normally, so probably the loo break was the best bit in terms of chatting, which sounds weird but it's a bit unnatural to ban chatting. (…) Some of the topics were much heavier than others and bigger than others and that wasn't reflected in the time allocation, so some of it felt like a bit of time filling and all the kind of introductory and end sessions felt a bit time filling when the actual topics really could have done with a lot more depth in some cases. (P2, intervention)

Right at the start was when we did the connecting. Well, it was a big one… I know CLANGERS starts with a C so you start with connect, but connecting is actually a really big one and actually can cause… I know we were talking about connecting with people outside, you are walking the dog, you chat with people you see and say hi, but you can actually come away feeling more lonely than you did when you went out and connected with them. (P1, intervention)

The one I disliked most of all was… It might have been relax. It was a lot to do with managing stress and it was all related to work type stress and very unrelated to the experiences that you have as a parent carer. Yes. Mmm. Once upon a time I had a job and that just pissed me off even more because actually I miss having a job enormously and it's just like [sighs] right, yes, great, yes, people get stressed at work, mmm, that would be nice [laughs]. Just a bit sort of… that one missed the mark quite badly for me but not necessarily for everybody else because it was hitting a sore spot really. (P2, intervention)

A lot of the videos were based on how to eat properly and how the brain works when you have exercise and I think that most people sort of understand these things and how beneficial they are but not everyone has access to them and not everyone can achieve what is put across in the videos and I was sort of more expecting help on… The thing is I think what you can't get away with being a parent carer is it's supposed to be for you as a parent carer but everything that you think about and you do, you think about your children, so you can't really get away from that aspect of it. So maybe if it was done in a way that it was for a parent carer and their children and what they can achieve then it would be different. (P7, intervention)

I must admit when I was watching the 'How to make a box' video, I was thinking, 'This is quite random.' For me, that was right up my street because I love making things like that, but I was thinking, 'If this was my husband watching it, he would probably be watching it thinking "Why am I watching this video?"' (P18, control)

### 2.3. Views on online materials

Mixed views:

I probably accessed about, well, 7 or 8 of them. Nearly all of them. I don't think I looked at the last one. But it just reinforced what we did in… I didn't do them… Of course, they only came up after, didn't they? I was going to say I didn't do them before because I didn't come up before, that's why. Yes, so I only accessed them after and then I think there was one occasion where the video didn't work or something… there was some reason why we couldn't see it and then I accessed it online. So it was great to have that as a backup if other things didn't quite work out in the session and to reinforce. (P1, intervention)

There's almost always something that I need to get done and if I've got access to a computer I'd rather be doing something more pleasant or relaxing or social than going through an online course on something that isn't central to my interests I guess. So yes, I think that goes back to why I was pleased that I got the real life group because it's like yes I actually will turn up and listen to that, whereas I'm not sure I will actually get round to doing an online course, I'd probably just go yes I just dropped out. (P2, intervention)

Initially I was curious and then I think it dwindled off because I felt like the course was covering everything so thoroughly that would it add anything to what I had already had? So I think that's probably why I didn't bother so much was because, yes, I felt that everything had been covered well during the session. (P4, intervention)

I missed most of [one] session. But then I went online and did it from online. Yes. It was really easy to get into and everything was on there, but I didn't take it in as well as I did on all the other days. It was the Connect and Learn were the two CLANGERS I wasn't there for. All of the other ones I think I got more out of than that one because even though I wrote stuff down from the internet onto the pages she gave me, it didn't sink in as well discussing it. (P5, intervention)

The week that I missed, I went through and used the online materials for that. So yes, a little bit, but I didn't do it systematically alongside the group each week… Once I'm out of the room, group, I've got a hundred million other things that take priority over that. (P6, intervention)

I thought they were really good, the little videos and things like that, I thought it was really helpful information and I think had I have been accessing it every week I would have found it helpful. I had a quick flick through the weeks to just have a quick nosey what it was about at the start. But yes, I do think it was helpful information, but I just didn't follow it through. (P13, control)

I really appreciated the reminders and I really appreciated the way it was laid out in saying what commitments are you going to make today to yourself, to anybody to do these things? But some of the online stuff was very good. (…) Some of the other stuff was just lowbrow and moved on, to be honest. It didn't keep my attention. Making a box, well, get real, come on. (…) So yes, there's some very good video content and yes it fitted together quite well, it's just that there was some demeaning stuff that I didn't do anything with, I just sort of saw it and thought that's not for me, moved it to one side and moved on. (P14, control)

What was there was absolutely fantastic and I think the videos were a good way of doing it because I, for one, am better at learning through watching the videos and being able to go back to them rather than just having loads and loads of information to read because obviously, you do get jaded and you do get tired, so… yeah, I thought it was done really well. (P18, control)

I found some of them were a bit patronising, a bit sort of childish [laughs] and very Americanised. There were like a few which I found more helpful or were quite pleasant to do, but some of the videos I thought were a bit, yeah, a little bit childish. (P17, control)

*Lack of groups as impeding the learning:*

I always put it off to the next day and the next day, whereas if it was a group I would just go to it as if it was an appointment, and I forget about things all the time otherwise. (P13, control)

I think although it was very useful to have it online, I did like that, but I wish there had been an option to maybe meet up once or twice in the group. I'm the sort of person that when I'm (recording inaudible 0:15:05.2) myself I do like getting out and about and meeting other people who have got similar things and I think it would have been… If you'd have had this group locally, I would have loved to have gone every week and I think that if I'd have gone every week I probably would have finished it no matter what and I think that personally I would have maybe got even more out of it by engaging with other people. (…) Maybe just gaining new friendships, to be able to have that contact with people after the group had finished. Yes, just sharing experiences, sharing tips. Lots of people have different ways of dealing with things and you get ideas from other people or you find out about things to support your children or your family or yourself that maybe you have not heard about. (…) I think it's really useful to be able to have that physical contact with other people really. (…) if you are in a group and you went back the next week and you said, "How did you get on?" it would just encourage you to discuss what you'd done that week and, as I said, maybe learn from other people and just taking it forward, being able to just be perhaps more motivated, I guess. If you are in a group and you see lots of people benefitting it just encourages you. (P15, control)

I was a bit disappointed because I'm in [town] and I thought oh I could do this every week, it will get me out of the house, because that would have given me the incentive to go somewhere and get it and it would have been a walk and some exercise to get there. I was thinking all that in my head, but actually even getting there would be part of it, part of the looking after myself. So I thought online it wasn't going to be as useful. (…) it took a discipline to sit down and actually make that time to look at the second, go back to it. (…) I did forget about it because you've got lots of things to do, whether you had a little reminder or something where you could check in or just say what you… you know what I mean? How did you find doing that part of it or maybe a bit of homework or something [laughs]. You could say, "Oh I didn't manage to do it," but it might be that incentive just to do something, whereas if no one is checking in on you just to say, "How did you find that?" or "Did you do anything?" then you are more likely to not and to put it lower down your list, aren't you? So if I had someone who I thought they are going to ask me what I've done, even like an email, they are going to ask me what I've done, I'll feel like I've got to do it, to try it, yes, but because there was nobody doing that, I didn't push myself. (P16, control)

I found doing the online course quite isolating. Personally, I would have… I think it would have made more changes in a quicker timespan if I had been in the group one. For me, it is not so much… like I said earlier, it is having that group session, it is being with people who are going through very similar things and I think if you have got the pressure, for want of a better word, of a group session, you are more likely to make those changes. Do you know where I am coming from? It is very difficult to put into words. But for me, I have done it online and I have ticked the box, if that makes sense. I have ticked the box. Yeah, I have done it, but it is… I think it is going to be more of a… it is going to be more difficult for me to be able to actually put those things into practice having done it online than if I was going to a course and they said, 'Right. Okay, what have you done for yourself this week?' (…) I think

that whilst it is brilliant to have had access to the course online, I do think it is something that possibly needs to be more personable, more face-to-face rather than a computer screen. Because that does isolate you in yourself. I am taking myself away from my husband and my family to go up and do it, which is great because it is time for me. (…) I do contradict myself, I know, because in one sense, it is easier to do it online because of the timings involved and things like that with work and all the balls that you are juggling, but on the other side, it is harder. It has been harder to do. (…) There was one video that was really quite long and I will not lie, I cannot even remember what it was about now, but it was probably the day I watched it, I had a really difficult emotional day and when I was watching this really long video, I think it was about eleven minutes, and I did find that I was very tired and I did find myself… my mind wandered because you are looking at a computer screen and you are thinking around you, 'I have got to do the washing. I have got to do this.' Because I was in my own environment, I found myself thinking about everything else that I should be doing, whereas if I was in… if I had gone to the group and they were watching that video, you have got nothing else to do, you are switched off. You are switched on to be watching that video, whereas for me, I was there thinking, 'Yeah. Okay, I have got to do this, so I will sit down and do this… and then my mind… like I say, I was… at one point, I was so tired that I was thinking, 'Oh gosh, I have got to go and make lunch, something for [name] and I to have for lunch tomorrow because I have got work, I have got issues at work.' (P18, control)

I found being in the online group really hard as well I could never find time to look at the resources. If I'd have been in the face to face group I'd have had allocated time to get out and access the sessions. (End-of-the-programme feedback)

I wasn't able to attend the groups so looked at the online resources and I don't tend to follow up on things from online. (End-of-the-programme feedback)

### *2.4. Factors influencing ability to engage with and benefit from the programme*

*Commitments and inadvertent events:*

Just giving myself time to do it really. I didn't prioritise it highly enough; I was just putting other things before it. I knew it was all at the back of my mind and I put a note up for me to look at it, but because there was nobody telling me, I did prioritise other things really. (P16, control)

There were certain things that came up in my life that I hadn't planned, an interview one day and stuff, that it was kind of a big commitment for that sort of six weeks to be able to sort of… if something came up and I was like, oh no, really, and I didn't want to let people down obviously, but there was a few of us that had to take days off here, there and everywhere and maybe it was a bit too long. In hindsight, it probably would have been easier to have done the online stuff. (P7, intervention)

I've had this chest infection, which I missed the last session unfortunately because I was really quite ill and I didn't want to pass it on to other people who are looking after vulnerable people particularly. (P3, intervention)

I think the people who are kind of at the not coping end of the spectrum are likely to not make a lot of the sessions because you have to drop everything all the time. (P2, intervention)

I put it in a sheet, put it in Excel, added a few columns, put in words and scoring and, yes, I'm quite happy for you to see it. It just explains on some days you are impacted by inadvertent events and there is no relaxation, there is five hours' sleep. That's just the way it is. (P14, control)

At the end of a very long day when you are exhausted, you have been to work, you have had a meltdown from an unstable children, you are then getting them to bed, cooking them tea, bla bla bla and then you sit down, you can read something online, but it does not necessarily go in, so it was good to print some bits off, so that I have got that for future. (P18, control)

*External factors:*
When you have got too much in the way of other things that you are not getting what you need, you are not going to be able to look at it. So yes, I don't know that it felt massively relevant to me because I have got bigger fish to fry. (…) I have some really quite big social care needs and health care needs of my own that are not being met and so it is much more difficult to sit there going, "Ah well, have I eaten properly today or have I…?" That kind of stuff isn't the kind of… But those things don't exist so it's better than nothing. (…) But I think it's working really well for some people. Yes, I can see that other members of the group were getting masses more out of it than I was. So I think if it's the right thing for you then it's clearly way more beneficial. (P2, intervention)

To be honest, the timing of this course could not have been worse [laughter] because my colleague has just been diagnosed with lymphoma, so he has been taken away and he is undergoing really aggressive chemo at the moment, so that means we are a man short in the office and I am thinking, 'Oh my goodness, I have got all this to contend with at the minute,' but that is a parameter that you cannot… as a group, that is not your fault. That is just one of those things. That is not something that you can control. (…) Ironically, I do not know if you have got access to my before and my after, but I actually think my after ones are possibly the answers that are not quite so good in terms of my mental health than the beginning ones but like I say, that is circumstances out of your control because it is nothing to do with reflecting on the course; it is just purely that we have had so much negativity and bad things happening in the last six weeks, that that is possibly reflected in that, but like I say, there is not an awful lot you can do because that is outside of your control. (P18, control)

Unfortunately I have experienced many personal stressful situations since starting the programme which has not helped me to focus on it as much as I had hoped if things had been stable. (End-of-programme feedback)

If there is no respite available and you'll see very little difference as you can't implement what you learn - care is 24-7 in some cases. (End-of-programme feedback)

My son was poorly so I was only able to access so much. (End-of-programme feedback)
