# Supplementary material for: Healthy Parent Carers programme: mixed methods process evaluation and refinement of a health promotion intervention
Source: BMJ Open. 2021 Aug 24;11(8):e045570. doi: 10.1136/bmjopen-2020-045570 (PMC8388296; doi:10.1136/bmjopen-2020-045570)
Supplement: Author's manuscript [file bmjopen-2020-045570.draft_revisions.pdf]

BMJ Open

BMJ Open is committed to open peer review. As part of this commitment we make the peer review history of every article we publish publicly available.

When an article is published we post the peer reviewers' comments and the authors' responses online. We also post the versions of the paper that were used during peer review. These are the versions that the peer review comments apply to.

The versions of the paper that follow are the versions that were submitted during the peer review process. They are not the versions of record or the final published versions. They should not be cited or distributed as the published version of this manuscript.

BMJ Open is an open access journal and the full, final, typeset and author-corrected version of record of the manuscript is available on our site with no access controls, subscription charges or pay-per-view fees (http://bmjopen.bmj.com).

If you have any questions on BMJ Open's open peer review process please email info.bmjopen@bmj.com

BMJ Open

Healthy Parent Carers feasibility randomised controlled trial: process evaluation and refinement of a health promotion intervention for parent carers of disabled children

Journal:	*BMJ Open*
Manuscript ID	bmjopen-2020-045570
Article Type:	Original research
Date Submitted by the Author:	15-Oct-2020

Complete List of Authors:	Lloyd, Jenny; University of Exeter Medical School, NIHR ARC South West Peninsula (PenARC), Institute of Health Research<br>Bjornstad, Gretchen; University of Exeter Medical School, Peninsula Childhood Disability Reseach Unit (PenCRU) and NIHR ARC South West Peninsula (PenARC), Institute of Health Research<br>Borek , Aleksandra ; Oxford University, Nuffield Department of Primary Care Health Sciences<br>Cuffe-Fuller, Beth; University of Exeter Medical School, Peninsula Childhood Disability Reseach Unit (PenCRU), Institute of Health Research<br>Fredlund, Mary; University of Exeter Medical School, Peninsula Childhood Disability Research Unit (PenCRU) Family Faculty, Institute of Health Research<br>McDonald, Annabel; University of Exeter Medical School, Peninsula Childhood Disability Research Unit (PenCRU) Family Faculty, Institute of Health Research<br>Tarrant, Mark; University of Exeter Medical School, NIHR ARC South West Peninsula (PenARC), Institute of Health Research<br>Berry, Vashti; University of Exeter Medical School, NIHR ARC South West Peninsula (PenARC), Institute of Health Research<br>Wilkinson, Kath; University of Exeter Medical School, Peninsula Childhood Disability Research Unit (PenCRU) and NIHR ARC South West Peninsula (PenARC), Institute of Health Research<br>Mitchell, Siobhan; University of Exeter Medical School, NIHR ARC South West Peninsula (PenARC), Institute of Health Research<br>Gillett, Annette; University of Exeter Medical School, Peninsula Childhood Disability Research Unit (PenCRU), Institute of Health Research<br>Logan, Stuart; University of Exeter Medical School, Peninsula Childhood Disability Research Unit (PenCRU) and NIHR ARC South West Peninsula (PenARC), Institute of Health Research<br>Morris, Christopher; University of Exeter Medical School, Peninsula Childhood Disability Research Unit (PenCRU) and NIHR ARC South West Peninsula (PenARC), Institute of Health Research

Keywords:	QUALITATIVE RESEARCH, STATISTICS & RESEARCH METHODS, PREVENTIVE MEDICINE

BMJ

I, the Submitting Author has the right to grant and does grant on behalf of all authors of the Work (as defined in the below author licence), an exclusive licence and/or a non-exclusive licence for contributions from authors who are: i) UK Crown employees; ii) where BMJ has agreed a CC-BY licence shall apply, and/or iii) in accordance with the terms applicable for US Federal Government officers or employees acting as part of their official duties; on a worldwide, perpetual, irrevocable, royalty-free basis to BMJ Publishing Group Ltd ("BMJ") its licensees and where the relevant Journal is co-owned by BMJ to the co-owners of the Journal, to publish the Work in this journal and any other BMJ products and to exploit all rights, as set out in our licence.

The Submitting Author accepts and understands that any supply made under these terms is made by BMJ to the Submitting Author unless you are acting as an employee on behalf of your employer or a postgraduate student of an affiliated institution which is paying any applicable article publishing charge ("APC") for Open Access articles. Where the Submitting Author wishes to make the Work available on an Open Access basis (and intends to pay the relevant APC), the terms of reuse of such Open Access shall be governed by a Creative Commons licence – details of these licences and which Creative Commons licence will apply to this Work are set out in our licence referred to above.

Other than as permitted in any relevant BMJ Author's Self Archiving Policies, I confirm this Work has not been accepted for publication elsewhere, is not being considered for publication elsewhere and does not duplicate material already published. I confirm all authors consent to publication of this Work and authorise the granting of this licence.

For peer review only

Healthy Parent Carers feasibility randomised controlled trial: process evaluation and refinement of a health promotion intervention for parent carers of disabled children

Lloyd J, Bjornstad G, Borek A, Cuffe-Fuller B, Fredlund M, McDonald A, Tarrant M, Berry V, Wilkinson K, Mitchell S, Gillett A, Logan S, Morris C

Jenny Lloyd 01392 722972 (Corresponding author)
J.J.Lloyd@exeter.ac.uk
NIHR ARC South West Peninsula (PenARC), Institute of Health Research, University of Exeter Medical School, University of Exeter, St Luke's Campus, Heavitree Road, Exeter, EX1 2LU

Gretchen Bjornstad
g.j.bjornstad@exeter.ac.uk
Peninsula Childhood Disability Research Unit (PenCRU) and NIHR ARC South West Peninsula (PenARC), Institute of Health Research, University of Exeter Medical School, University of Exeter, St. Luke's Campus, Heavitree Road, Exeter, EX1 2LU

Aleksandra Borek
aleksandra.borek@phc.ox.ac.uk
Nuffield Department of Primary Care Health Sciences, Medical Sciences Division, University of Oxford, Radcliffe Observatory Quarter, Woodstock Road, Oxford, OX2 6GG

Beth Cuffe-Fuller
B.Y.R.Cuffe-Fuller@exeter.ac.uk
Peninsula Childhood Disability Research Unit (PenCRU), Institute of Health Research, University of Exeter Medical School, University of Exeter, St. Luke's Campus, Heavitree Road, Exeter, EX1 2LU

Mary Fredlund
mary_fredlund@hotmail.com
Peninsula Childhood Disability Research Unit (PenCRU) Family Faculty, Institute of Health Research, University of Exeter Medical School, University of Exeter, St. Luke's Campus, Heavitree Road, Exeter, EX1 2LU

Annabel McDonald
tinkabelmcd@gmail.com
Peninsula Childhood Disability Research Unit (PenCRU) Family Faculty, Institute of Health Research, University of Exeter Medical School, University of Exeter, St. Luke's Campus, Heavitree Road, Exeter, EX1 2LU

Mark Tarrant
m.tarrant@exeter.ac.uk
NIHR ARC South West Peninsula (PenARC), Institute of Health Research, University of Exeter Medical School, University of Exeter, St Luke's Campus, Heavitree Road, Exeter, EX1 2LU

Vashti Berry
V.Berry@exeter.ac.uk
NIHR ARC South West Peninsula (PenARC), Institute of Health Research, University of Exeter Medical School, University of Exeter, St Luke's Campus, Heavitree Road, Exeter, EX1 2LU

For peer review only

For peer review only - http://bmjopen.bmj.com/site/about/guidelines.xhtml

Kath Wilkinson
K.Wilkinson2@exeter.ac.uk
Peninsula Childhood Disability Research Unit (PenCRU) and NIHR ARC South West Peninsula (PenARC), Institute of Health Research, University of Exeter Medical School, University of Exeter, St. Luke's Campus, Heavitree Road, Exeter, EX1 2LU

Siobhan Mitchell
s.b.mitchell@exeter.ac.uk
NIHR ARC South West Peninsula (PenARC), Institute of Health Research, University of Exeter Medical School, University of Exeter, St. Luke's Campus, Heavitree Road, Exeter, EX1 2LU

Annette Gillett
A.Gillett@exeter.ac.uk
Peninsula Childhood Disability Research Unit (PenCRU) and NIHR ARC South West Peninsula (PenARC), Institute of Health Research, University of Exeter Medical School, University of Exeter, St. Luke's Campus, Heavitree Road, Exeter, EX1 2LU

Stuart Logan
Stuart.Logan@exeter.ac.uk
Peninsula Childhood Disability Research Unit (PenCRU) and NIHR ARC South West Peninsula (PenARC), Institute of Health Research, University of Exeter Medical School, University of Exeter, St. Luke's Campus, Heavitree Road, Exeter, EX1 2LU

Christopher Morris
christopher.morris@exeter.ac.uk
Peninsula Childhood Disability Research Unit (PenCRU) and NIHR ARC South West Peninsula (PenARC), Institute of Health Research, University of Exeter Medical School, University of Exeter, St. Luke's Campus, Heavitree Road, Exeter, EX1 2LU

For peer review only

For peer review only - http://bmjopen.bmj.com/site/about/guidelines.xhtml

Abstract

Objectives: Parent carers of children with additional needs and disabilities are at risk of poorer mental and physical health. In response to these needs, we co-developed the 'Healthy Parent Carers' intervention (HPC). This paper presents the views and experiences of participants in HPC feasibility RCT and programme refinement.

Intervention, setting and participants: HPC is a group-based intervention (supported by online materials) for primary carers of disabled children, encouraging behaviours linked with health and wellbeing. It was delivered by two lead and six assistant peer facilitators in six community sites (one lead and one assistant per group) in the South West of England over six or twelve sessions. Control participants had online materials only. The trial involved 47 intervention and 45 control parent carers and eight facilitators.

Design: A pre-planned mixed methods process evaluation using questionnaires and checklists as well as qualitative interviews with a sub sample of 18 participants and a focus group with facilitators was conducted at the end of the feasibility trial.

Results: HPC was highly acceptable to participants and facilitators and experiences were very positive. Participants felt that the programme increased awareness of what parent carers could and could not change and their self-efficacy to engage in simple, health-promoting behaviours. The intended mechanisms of action (social identification and peer support) matched participants' expectations and experiences. Control participants found the online-only programme flexible but isolating as there were no opportunities to share ideas and problem-solve with peers, the key function of the programme. Areas for improvement were identified for programme content, facilitator training and delivery.

Conclusion: HPC was acceptable, well-received and offers considerable potential to improve the health of parent carers. Under the pandemic, the challenge going forward is how best to maintain reach and fidelity to function whilst delivering a more virtual programme.

Trial registration: ISRCTN151144652

For peer review only

Strengths and Limitations of this study

- The HPC programme has followed the key principles of intervention development
- Qualitative and quantitative data have been synthesised to refine and optimise the intervention
- The intervention refinement process is transparent, and adaptations reflect the process data collected
- Key uncertainties to be addressed in future research have been identified
- Experiences and views are not necessarily representative of parent carers from ethnic and minority groups.

Background

Parent carers of children with additional needs and disabilities are at increased risk of poorer mental [1-10] and physical health.[2, 3, 6, 7, 11-13] They often experience challenges to maintaining good health that have implications for their wellbeing and their ability to care for their children. In response to these needs, we co-developed the 'Healthy Parent Carers' (HPC) programme with considerable input from parent carers. This health promotion intervention encourages behaviours linked with health and wellbeing known by the acronym 'CLANGERS':[14] (1) Connecting with people, (2) Learning, (3) being Active, (4) taking Notice, (5) Giving, (6) Eating well, (7) Relaxing, and (8) maintaining Sleep hygiene. The programme was designed to be peer-led and group-based. It involves a range of activities to improve parent carer confidence, motivation and self-efficacy to plan, prioritise and enact strategies to improve their own health and wellbeing, whilst expanding their social network and providing peer-to-peer social support.

Intervention Mapping [15] with extensive stakeholder involvement was used to develop the programme based around a set of universal and evidence-informed wellbeing actions (supplementary document 1). The programme was piloted with one group of seven parent carers, delivered by two peer facilitators, with whom the intervention was co-created. The findings of this proof-of-concept study and details of the intervention development, logic model and content were published previously.[16] Box 1 summarises programme content, format, and delivery.

Insert Box 1

For peer review only - http://bmjopen.bmj.com/site/about/guidelines.xhtml

The Feasibility Trial

The feasibility trial aimed to assess whether the programme could be delivered in the community and evaluated the acceptability of a randomised trial design.[17] The trial ran between July 2018 and June 2020. Ninety-two participants were randomised: 47 to the HPC group programme (delivered in 6 community sites across Cornwall, Devon and Somerset) and 45 to the control group, which involved access to the HPC online resources only. The group sessions ran between January and July 2019. Outcome measures were collected at baseline (prior to randomisation), immediately post intervention and six months later (supplementary document 2).

HPC group sessions were delivered by pairs of peer Lead and Assistant Facilitators who were themselves parent carers of children with chronic health conditions. Two Lead Facilitators, experienced in delivering group training to parent carers, were recruited from the Council of Disabled Children (CDC). Nine Assistant Facilitators (including three reserves) were recruited through recommendations from our Stakeholder Advisory Group and through local network adverts. Volunteers were shortlisted and interviewed by telephone. Selection decisions were made based on a practical understanding of the challenges faced by parent carers in relation to their own health and wellbeing with reference to our person specification, and availability to deliver at one of the selected venues. Details of the training programme are in Box 2.

One of the Lead Facilitators delivered the six-week programme to two groups (two sites) and one delivered to four groups (four sites), one of which followed the 12-session format (Box 1). Each Assistant Facilitator was assigned to support delivery in one of the six groups.

Insert Box 2

Figure 1 shows the flow of participants in the trial and details of data collection.

Insert Fig 1

This paper reports on the motivations of parent carer participants and facilitators to take part, their views and experiences of the HPC programme and its subsequent refinement.

Methods

For peer review only - http://bmjopen.bmj.com/site/about/guidelines.xhtml

Data Collection

Qualitative and quantitative data were collected from participants and facilitators on programme uptake, reach, training, delivery, and experience as well as suggestions for improvement. Box 3 presents the measures, (details in supplementary documents 3-10) and sampling process.

Insert Box 3

Data Analysis

Qualitative data

All qualitative data was uploaded to NVivo (version 12). Taking both an inductive and deductive thematic approach to analysis, we developed a coding framework to categorise the data from participant interviews, facilitator focus group, facilitator support calls, and free-text data from the participant and facilitator questionnaires. Three parent interviews were coded independently by three researchers (BCF, AB, JL) initially and compared to agree the coding framework, which included combining codes and arranging them into higher-level categories to organise the data. While the detailed codes were developed inductively, the categories were more deductive and followed the key areas of interest in the process evaluation, reflecting the interview topic guide. The agreed framework was then used to code the remaining interviews, adding new inductive codes when identified. All interview transcripts (n=18) were coded by BCF, and 50% (n=9) were double-coded by AB and JL. The focus group transcript was coded by BCF and checked by JL and AB.

Quantitative data

Relevant quantitative questionnaire data in relation to participant and facilitator experiences and views were collated and presented descriptively alongside the qualitative findings.

Data Synthesis for Intervention Refinement

Data relevant to programme improvement were extracted from NVivo. These data were and sorted into three categories: facilitator training, intervention delivery, and intervention content (including online materials). For each suggestion, the source of data was noted, including when there were mixed or contradictory views. Figure 2 shows the refinement process and possible refinements were prioritised using a colour-coded system.

Insert Fig 2

For peer review only

Findings

Figure 1, shows the number of participants who provided each type of process evaluation data. Completeness of questionnaire data was high for participants (>80%) and facilitators (100%). Interviews were carried out with eighteen parent carers (12 intervention - 2 per group/site; 6 control – 1 per group/site) and the focus group involved all lead and assistant facilitators (n=8).

1. Facilitator experiences

1.1. Motivations and expectations

Five out of eight facilitators had experience of facilitating parent carer groups. Most reported being interested in delivering the programme because it fitted with their skills and interests, and offered an opportunity to further develop their professional skills and confidence. They recognised the need for, and importance of, health and wellbeing training for parent carers, believed in the value of the programme (and group support), and wanted to support other parent carers. All reported interest in contributing to the research and programme development, and five hoped to benefit from the programme themselves.

1.2. Training, preparation and support

Following block 1, 2 and 3 of training (Box 2), all Lead and Assistant Facilitators completed the facilitator training feedback questionnaire. Overall scores were out of 5. Knowledge/understanding and skills/confidence for each block were high at 4.5 and 4.6; 4.5 and 4.3; 4.6 and 4.45, respectively. The only component to decrease over the training was the facilitator's skills and confidence to manage difficult/sensitive issues. Thus, unsurprisingly, in the focus group following programme delivery, they suggested the training could focus more on the delivery style and practice, and managing difficult issues specific to parent carers likely to arise in group discussions. They suggested that future facilitators could be recruited from those completing the programme, so facilitators would possess a greater understanding of the content and process and be able to build on their own experience of the programme. They also suggested better clarifying the roles and responsibilities of Lead and Assistant Facilitators, and to be matched and trained together. They reported satisfactory support throughout programme delivery (i.e. supervision from CDC, support calls with the research team), but particularly valued the sharing and support among fellow facilitators. Nearly three quarters of facilitators believed that sufficient time had been allocated to training.

1.3. Programme content and group delivery

Facilitators perceived the following benefits of the programme for participants: the focus on parent health/wellbeing (rather than their children); the face-to-face group delivery (rather than online);

For peer review only - http://bmjopen.bmj.com/site/about/guidelines.xhtml

the consistent and predictable session structure; the weekly goal setting (but suggested simplifying 'SMART' to 'achievable' goals). They perceived both 6- and 12-week delivery as suitable.

They found the practical activities an important component (in addition to group discussions) but some activities triggered difficult feelings among some participants. The facilitators reported that some participants disclosed difficult personal issues, which were challenging to address in the group without turning into counselling sessions; thus, the facilitators stressed the importance of training in managing such situations. This was more problematic when groups were small, and the facilitators agreed that 6-8 participants was an optimal group size. The facilitators emphasised the importance of facilitators being parent carers, and how the positive experiences of co-facilitating and the programme helped them reflect on their own health and wellbeing.

Facilitators thought that each session went well, and that participant engagement was high, their average ratings across groups for each session ranging between 4.4 and 4.8 out of 5 for how well they thought the session went and 4.5-5 out of 5 for parent engagement. Facilitator judgement of how connected their groups were following each session was very high and increased over the course of the programme from 5/7 to 7/7. Box 4 presents selected quotes illustrating facilitators' experiences.

Insert Box 4

2. Participants' experiences

Below we refer to 'interviewees' to indicate the views only of those interviewed and to 'participants' to indicate data collected from the questionnaires.

2.1. Motivation to participate and expectations

All interviewees (n=18) reported participating in the programme to focus on and find ways to improve their health and wellbeing. Some wanted to do that to better support their families, and to feel less guilt for taking time to take care of themselves. A few interviewees thought the programme took place at the right time for them: when they were able, ready and available to participate and focus on their health. Most interviewees also reported wanting to contribute to research and help other parent carers, especially as they perceived little available support for parent carers.

The majority of interviewees in the group programme reported positive aspects of face-to-face delivery, such as peer support and dedicated time to share and discuss issues affecting their wellbeing. A few participants, mainly from the online-only control arm, reported that the flexibility of the online programme enabled them to engage with the programme more easily than attending face-to-face groups. Overall, group-based delivery was preferred, including those who had not

For peer review only - http://bmjopen.bmj.com/site/about/guidelines.xhtml

experienced it, as groups were perceived to instigate a stronger commitment to engage with the programme.

2.2. Experiences of the group programme and its impact

Positive experiences: Overall 84% (37/44) of participants were satisfied or very satisfied with the group programme, with 67% (28/42) finding it useful in helping them to improve their health and wellbeing. Most of the interviewees echoed this, noting the positive impact of the programme on them and their families. Two reported that it 'pushed them out of their comfort zones' by addressing some uncomfortable but important issues. Several participants commented that the programme should be rolled-out to benefit all parent carers.

Groups: All participants who responded to the post intervention questionnaire felt included and part or very much 'included and part of the group' with 85% (17/20) indicating that session length was 'about right'. All intervention interviewees described one of the main benefits of the programme as having the opportunity to discuss and share with other parent carers in a supportive, empathetic, safe, and respectful group context. They highlighted the value of peer support and discussions in motivating change. They also perceived the group programme as providing more than support groups because of the practical activities and exploring barriers and solutions in specific areas. Some were surprised by the small group size but liked it as it enabled them to participate and get to know each other and bond as a group; yet, most thought that slightly larger groups would provide more varied perspectives. Interviewees noted also that the group programme gave them time to focus on themselves and permission (to take time) to take care of themselves.

Peer facilitators: All participants who responded to the questionnaire were satisfied or very satisfied with the way the programme was delivered, and interviewees highlighted the critical importance of the facilitators being parent carers, enabling shared understanding and empathy. They valued facilitators delivering the programme together ('complementing each other' and 'bouncing off each other'), facilitating learning through group discussions (rather than 'teaching'), creating positive group context (with facilitators seen as part of the groups), and being knowledgeable, understanding and kind.

Content and activities: Interviewees generally had positive comments about programme content and preferences for different activities. The content and activities were mainly valued for providing structure and prompts to group discussions and focus on different life areas. The promoted messages were seen as 'common sense' and applicable to all people, but more challenging to parent carers. The programme reminded participants about the importance of health/wellbeing, and

For peer review only

discussing/sharing reinforced that, thus prompting them to make changes.

Reflecting and setting small achievable goals: Interviewees valued the programme's focus on simple and meaningful actions (CLANGERS) that increased their awareness of areas of their lives in which they could make positive changes (while also helping accept things that they could not change). They also valued focusing on small steps that they can take, setting achievable goals at each session/module, and then reflecting on them. This helped them feel more in control of doing something positive about their health/wellbeing. 76% (35/46) reported making changes.

Less positive experiences: Despite overwhelmingly positive experiences, nine participants did not find the programme useful, with 5 indicating ambivalence. A few interviewees reported a less positive experience and *not* making changes, mainly due to factors outside the programme (described below); however, they still valued the raised awareness and 'hope' it provided. This was reflected in the quantitative feedback with 24% (11/46) reporting not making changes. A few interviewees reported feeling apprehensive and nervous at the start of groups; finding different activities or content difficult, challenging or less appealing; feeling uncomfortable with others in the group (e.g., due to expressed views/comments); perceiving less shared experience and challenges with others in the group (e.g., due to personal or system-related contexts).

2.3. Views on online materials

Participants' experiences of, and views on, using online materials were mixed. Interviewees attending the groups reported finding the online materials unnecessary or just reinforcing the sessions. Interviewees from the control group reported some positive experiences of the online-only programme (valuing videos as an engaging way of providing information) but found some content less relevant or helpful. Overall satisfaction of control participants with the programme was good, although lower than intervention participants (66% vs 84%), with only 38% (11/29) finding the programme 'useful in helping them to improve their health and wellbeing', although 65% (20/31) reported making changes.

Interviewees in both study arms described the importance of group discussions in enhancing the learning and programme impact. Without group sessions, some found the online-only programme isolating (without opportunities to discuss, share ideas and problem-solve with other parent carers) and harder to focus on because of lacking reminders and scheduled time (with other matters taking priority).

For peer review only - http://bmjopen.bmj.com/site/about/guidelines.xhtml

2.4. Factors influencing ability to engage with and benefit from the programme

Interviewees in both study arms reported factors that affected their ability to attend the sessions or use online materials, including: other (unexpected) commitments, lack of time, inadvertent events (their or children's illness), access/transport, and childcare. A few also discussed external factors out of their control (e.g., social care needs, access to respite, work situation) that affected their wellbeing and ability to benefit from the programme, reinforcing the importance of participating in the programme at the right time in their lives. Box 5 presents selected quotes illustrating participants' experiences

Insert Box 5

Additional quotes and comments from facilitators and participants can be found in supplementary document 11.

3. Programme refinement

The process evaluation confirmed that the HPC programme was highly acceptable to both parent carers and facilitators with the vast majority expressing positive experiences; however less positive experiences were carefully considered in the refinement process (Figure 2). Several potential areas for improvement were identified and, where possible, incorporated into the refined HPC programme. Key changes are outlined below.

3.1. Optimising session and online content

- Wording of the online materials simplified to increase understanding, engagement, and usage.
- Examples of SMART goals added to online materials and delivery manual to increase understanding, and 'SMART' replaced with 'achievable'.
- Create or select videos more relevant to parent carers.

3.2. Optimising training

- Explanation of the rationale for certain activities included to increase understanding of their purpose.
- Training in managing challenges that specific content/activities may generate, and worked examples on how these may be addressed.
- Training in how best to present and deliver the videos to elicit discussion around the key 'take away' messages.
- Facilitators to complete the online modules prior to training so that that they are familiar with the CLANGERS and resources, providing more time to focus on delivery strategies.
- Group dynamics session refined so that it is more practical and interactive (scenarios and practice included in how to build, enhance and maintain group cohesion under challenging situations; how to find commonalities in shared experience despite having differing challenges/situations).

For peer review only - http://bmjopen.bmj.com/site/about/guidelines.xhtml

- Delivery process for each of the CLANGERS to be modelled in detail using ‘Connect’ (as it is the most challenging of the CLANGERS to deliver and experience) to ensure that facilitators understand the application of the programme’s theory of change.
- Trainers asked to create and disseminate a FAQ document to follow up any questions/concerns not addressed in the training due to time constraints.
- Increased focus on developing delivery skills using modelling techniques to improve confidence and quality of delivery (e.g. modelling good responses to parent questions, supporting parents who are struggling).
- Roles and responsibilities of the Lead and Assistant Facilitators clearly explained to avoid misunderstanding and enhance team working.

3.3. Optimising delivery

- Lead and Assistant Facilitators to communicate with each other prior to the first session (using the ‘preparation’ checklist) to allay any delivery fears/concerns.
- Prior to session 1, facilitator photo, with a written introduction to be sent to participants; Lead Facilitators to call participants to introduce themselves, ascertain any practical support required and take steps to provide this support where possible.
- Advice on managing difficult situations added to the delivery manual.
- Description of roles and responsibilities of the Lead and Assistant Facilitators added to the delivery manual.
- Key ‘take away’ messages from each video added to the delivery manual.
- Delivery manual adapted to accommodate the 12x2 hour format to improve coherence.
- Completion of a simple participant feedback form at the end to provide information to improve future group delivery.
- Change minimum viable group number from 4 to 6 to increase the range of perspectives and create better conditions for peer-to-peer learning and support.

Discussion

This paper presents facilitator and parent carers’ views on and experiences of the HPC programme from the feasibility RCT, which have informed the refinement of the programme. We are now exploring implementation uncertainties, funded by the Economic and Social Research Council Impact Accelerator Account Strategic Initiative Award (ES/T501906/1) in preparation for a future evaluation of programme effectiveness. Findings show that the group programme is valued for providing peer support and practical activities, where difficult and emotional conversations are facilitated and explored. This is a key function of the programme, which accounted for the high levels of satisfaction and reported impact on both the parent and the wider family. It appears that this aspect of the programme necessitates larger groups (6-12) to allow for varied perspectives and a ‘facilitation’ rather than ‘counselling’ approach. Group size, therefore, will be explored as part of intervention fidelity to function [18, 19] in the definitive trial.

As intended, participants felt that the programme increased awareness of what parent carers could and could not change and their self-efficacy to engage in health-promoting behaviours (CLANGERS).

For peer review only - http://bmjopen.bmj.com/site/about/guidelines.xhtml

Participant motivations and expectations showed that, overall, the target group was reached (i.e. those who wanted and felt ready to do something to improve their own health/wellbeing), with the intended mechanisms of action (social identification and peer support) matching the participants' expectations and experiences.

Facilitators reported that the relationship between the Leads and Assistants was important for effective delivery and that clarity on and practice of these different roles were a crucial part of training and quality delivery. Both intervention participants and facilitators thought that, to deliver the programme effectively, Leads and Assistants needed to have completed the programme themselves and that the development of a network of facilitators to share experiences of delivery using support calls and/or online meetings was important in supporting them to improve their practice.

The strength of this research is that it has systematically followed key principles of intervention development [20, 21] and refinement, using a dynamic, iterative and creative process with extensive stakeholder consultation, where the developers have been open to change based on data collected in a series of iterations.[16, 17] We examined how the intervention will be evaluated in the next phase of research and identified learning and key uncertainties to be addressed, such as blended (online and face-to-face) delivery of group sessions, commissioning and implementation. Reporting mixed methods data on participant views and experiences and the subsequent refinement process in feasibility studies is recommended.[22] Not only does it increase knowledge about the intervention refinement/optimisation process but it also allows others to link up intervention development processes and subsequent trial outcomes. However, the study has limitations. The lack of ethnic diversity in South-West England, where this study was conducted, meant that the sample was ethnically homogeneous, thus experiences and views may not represent parent carers from different cultures and contexts. Most work on ethnicity and parenting interventions has been conducted in the USA and is equivocal about whether outcomes differ by ethnicity.[23, 24] However, adapting parenting interventions for different ethnic groups poses many issues. Adaptation may neither be practical or a desirable service model for multi-ethnic European cities.[25] Presently, we do not have the available data as individual trials are not powered to test intervention effects by ethnicity. Understanding how ethnicity might influence the development of a shared group identity, recognised as a key mechanism of action for group-based programmes, will be explored in a future programme of work, to support further programme refinement.

This study has helped to refine the programme in many aspects; however, there remain a number of barriers to parent carers' capacity to engage in all group sessions and benefit from the programme.

For peer review only - http://bmjopen.bmj.com/site/about/guidelines.xhtml

We need to explore these barriers in further detail and the extent to which they can be mitigated to enhance accessibility to participate in the programme. As we write, the COVID-19 pandemic has meant social distancing and increased challenges to running groups. We are therefore keen to explore to what extent the group programme could be delivered virtually using videoconferencing, which may overcome some other barriers to participation, but we would need to evaluate the extent to which peer support and cohesive support of the group is maintained.

Conclusion

The format, content and delivery of the HPC programme was highly acceptable to participants and for facilitators to deliver. The process evaluation data enabled programme refinement to optimise impact going forward. Although the programme focuses on promoting health and wellbeing at an individual level (i.e. individual psychological and behavioural change), we acknowledge the importance of other factors at interpersonal, community and societal levels that affect parent carers' health and wellbeing, such as access to services, negative public attitudes towards disability, which in turn impact on parent carers' capacity to make and sustain changes. The programme does, however, provide support and hope for those who find it difficult, both practically and psychologically, to attend to their own wellbeing. The challenge we face going forward is delivery of the programme in the shadow of COVID-19. The team plan to explore how this might be reimagined to accommodate a new way of supporting parents, minimising risk to health, whilst delivering an accessible and inclusive package that maintains fidelity to function.

Trial findings related to recruitment and retention, fidelity of intervention delivery, the feasibility and acceptability of trial processes and outcome data will be reported separately.

(4000 word count)

Declarations

Funding

This paper presents independent research funded by the National Institute for Health Research (NIHR) under its Research for Patient Benefit (RfPB) Programme (Grant Reference Number PB-PG-0317-20044). We also acknowledge support from the National Institute for Health Research Applied Research Collaboration South West Peninsula. The views and opinions expressed in this paper are those of the authors and not necessarily those of the National Institute for Health Research or the Department of Health and Social Care. Delivery of the Healthy Parent Carers programme is funded by Public Health England, Kernow Clinical Commissioning Group, Northern, Eastern and Western Devon Clinical Commissioning Group, and a grant from the National Lottery Community Fund

For peer review only - http://bmjopen.bmj.com/site/about/guidelines.xhtml

through the Reaching Communities programme. Peer review of the study design was provided during the NIHR funding application process. The funders have had no involvement in the writing of the manuscript and will not have any role during its execution, analyses, interpretation of the data, writing of the report, or the decision to submit the report for publication.

Ethics approval

The study procedures were approved by University of Exeter Medical School Research Ethics Committee (reference 18/06/174)

Competing interests

None

Author contributions

All authors contributed. CM led the development and preliminary evaluation of the programme and leads the study. AB, AM, MF and CM designed the original programme. JL, AB, CM, VB, MT, KW and BCF designed the process evaluation and programme refinement process. BCF, KW, GB, AG and SM coordinated, collected and managed data from parent carers and facilitators. AB led the analysis and write up of the qualitative interviews, supported by JL and BCF. JL drafted the manuscript and all authors provided feedback. SL facilitated infrastructure support through National Institute for Health Research Applied Research.

Patient and Public Involvement

Parent carers have been involved all stages of developing the intervention, designing the feasibility study and refining the programme in light of the findings from this feasibility trial, through the involvement of the Peninsula Cerebra Research Unit (PenCRU) Family Faculty (www.pencru.org/getinvolved/ourfamilyfaculty). The Family Faculty are parent carers who are offered opportunities to be involved in research.

Acknowledgments

For peer review only - http://bmjopen.bmj.com/site/about/guidelines.xhtml

We are grateful for the contributions of the PenCRU Family Faculty and feedback provided by the participants in both trials arms. We also thank our colleagues within the wider PenCRU team, Katharine Fitzpatrick for organising the parent carer working group meetings and Tania Hind for administrative support.

Data availability statement

The datasets analysed during the current study are available from the corresponding author on reasonable request.

Licence statement

The Submitting Author accepts and understands that any supply made under these terms is made by BMJ to the Submitting Author unless you are acting as an employee on behalf of your employer or a postgraduate student of an affiliated institution which is paying any applicable article publishing charge ("APC") for Open Access articles. Where the Submitting Author wishes to make the Work available on an Open Access basis (and intends to pay the relevant APC), the terms of reuse of such Open Access shall be governed by a Creative Commons licence.

For peer review only

List of Boxes

Box 1. Healthy Parent Carer's Programme and online materials

Box 2. Facilitator training programme

Box 3. Process evaluation measures

Box 4. Selected quotes illustrating facilitators' experiences

Box 5. Selected quotes illustrating participant' experiences

List of Figures

Figure 1. Flow of participants and measures

Figure 2. Programme refinement process

List of Supplementary Documents

Supplementary document 1 - HPC intervention details

Supplementary document 2 - Study protocol publication

Supplementary document 3 - Facilitator training feedback questionnaire

Supplementary document 4 - Facilitator delivery checklist (Module 1)

Supplementary document 5 - Facilitator focus group topic guide

Supplementary document 6 - Participant end of session questionnaire

Supplementary document 7 - Participant end of programme questionnaire

Supplementary document 8 - Participant 6 month follow up questionnaire

Supplementary document 9 - Participant interview topic guide (intervention)

Supplementary document 10 - Participant interview topic guide (control)

Supplementary document 11 - Additional quotes and comments from facilitators and participants.

For peer review only

For peer review only - http://bmjopen.bmj.com/site/about/guidelines.xhtml

Boxes

Box 1. Summary of the Healthy Parent Carer's Programme (including the online materials)

Face-to-face sessions

Content - Activities based on CLANGERS, an extension of the '5 ways to wellbeing'.

- Connect, Learn, be Active, Notice, Give, Eat well, Relax and Sleep.

Format – 12 modules over 24 hours

Setting – Community sites (2 special schools, 1 children's hospice, 1 Parent Carer Forum premises, 1 adult learning community venue, and 1 hotel regularly used for parent carer meetings).

Delivery – Six four-hour daytime sessions (comprising 2 modules per session) or 12 two-hour evening sessions (one module per session) delivered to groups of 4-12 parent carers.

Personnel - One Lead and one Assistant Facilitator per group.

Online materials

Included written content which provided participants with information on the CLANGERS, note-taking space to reflect on their own thoughts and templates for participants to develop their own goals and action plans. Audio and video recordings were also provided to illustrate each of the CLANGERS.

The content related to each module was released to participants in each group after it was delivered in their specific group sessions.

For peer review only - http://bmjopen.bmj.com/site/about/guidelines.xhtml

Box 2. Facilitator Training Programme

FORMAT

- **Block 1** - 2 days (Lead Facilitators only). November 2018
- **Block 2 -** 2 days (Lead and Assistant Facilitators). November 2018 (three weeks following Block 1)
- **Block 3 –** 1 day (Lead and Assistant Facilitators). April 2018 (after delivery of cohort 1)

CONTENT

- **Block 1** Overview of programme; exploration of wellbeing and the CLANGERS; facilitator roles and responsibilities; modelling delivery; research processes; safeguarding
- **Block 2** Overview of programme; exploration of wellbeing and the CLANGERS; facilitator roles and responsibilities; facilitator skills and competencies; group facilitation; managing group dynamics; modelling delivery; practicing delivery; research processes; safeguarding.
- **Block 3** Refresher training; review of CLANGERS, facilitator reflections, research processes, safeguarding.

PERSONNEL – Two parent carers (MF and BM) who co-produced the programme and delivered the proof of concept pilot.

review only

For peer review only - http://bmjopen.bmj.com/site/about/guidelines.xhtml

Box 3. Process evaluation measures and sampling

Facilitator training feedback questionnaire**:** To assess self-reported knowledge, understanding, skills and confidence to deliver the intervention and facilitator reflections on the training. Data were collated and averaged to provide an overall score out of 5 for each training block for knowledge and understanding (of the programme, facilitation techniques, developing a positive group dynamic and facilitator roles and responsibilities), skills and confidence (to present information, lead activities, create a positive group dynamic, manage time and difficult situations). (Supplementary document 3)

Facilitator support calls**:** To understand and respond to delivery challenges (two per site, one following week 1 and one halfway through the programme).

Facilitator delivery checklists*:* To understand experiences and views on group delivery (weekly checklist, also included a check on content delivered) (Module 1 example, supplementary document 4)

Focus group with facilitators: To understand Lead and Assistant Facilitator experiences and views on training, delivery, and programme content (supplementary document 5). The two-hour focus group took place once all groups were completed, led by JL and supported by BCF. The focus group was audio-recorded and transcribed verbatim (with any potentially identifiable information anonymised).

Participants' feedback questionnaires: To understand participant (control and intervention) experiences and views on programme content and delivery (end of each group session (supplementary document 6), end of programme (supplementary document 7) and 6 month follow up (supplementary document 8))

Participant phone interviews: To understand and explore participant experiences, views and engagement with the group sessions and online materials (intervention and control). (Supplementary document 9 and 10) Twelve intervention (two per group/site) and 6 control participants (one per site) were sampled to ensure that two out of the four male carers in the study (1 control and 1 intervention) were interviewed and the range of parent carer challenges were represented (selection was based on Lead Facilitator comments and participant end of programme feedback). Interviews were carried out by BCF and were audio-recorded and transcribed verbatim, with names and other identifying information changed for confidentiality. Interviews took place as soon as possible after participants completed their post-intervention measures and before the six-month follow-up.

For peer review only - http://bmjopen.bmj.com/site/about/guidelines.xhtml

Box 4. Selected quotes illustrating facilitators' experiences

1.1. Motivations and expectations

I feel that this training is a natural continuation to take forward the previous training I have been involved in. I am passionate about empowering parents with high quality information, support and advice. (Facilitator pre-training questionnaire)

For me it was a mixture of interlinked personal and professional reasons… I'm keen to do good in the world, if I can, keen to use some of my professional skills and enhance my professional skills while doing that. (AF, focus group)

I know the power of groups and supporting each other… I know it's so important to have people that understand and support you, because it can be lonely. I had lots of hope that this would help other people and, yes, it definitely was fulfilled. (AF, focus group)

1.2. Training, preparation and support

Parent carers are a very specific group to deliver to and maybe something that is more specific around the baggage, if you'll excuse me for using that word. The baggage that parent carers bring into the room is extremely specific and the group dynamic isn't managing conflict, usually, with parent carers. It's very different. (…) I always find parent carers very quick to bond and support each other. Very, very quick; they really do move forward in that way. But when you've got an individual... Something about managing the individual rather than the group as a whole. (LF, focus group)

I didn't know enough about the programme before I actually joined in as an assistant… now, it would be fine going back but when you suddenly do the programme you haven't got a clue what's necessarily coming up next and you don't know how, emotionally, you're going to feel. (…) It probably would have been easier if I'd done the programme myself, because I could have then looked back at some notes, so any things that I come across being difficult, I would have had a clearer focus rather than what's actually going on with me at the time. (AF, focus group)

AF: For me, the support calls were more about the sharing of experiences - and the issues and part of the process and the research as well. I guess you [the researcher] were kind of in research mode to some extent in those calls. But the support seemed to me to be within the facilitator team.

LF: I agree absolutely with that. Totally. I think that the relationship that we had as co-facilitators is where I drew my support about anything that wasn't just on a practical basis.

AF: And that's different levels of support needed. (Focus group)

1.3. Programme content and group delivery

The repetitiveness of the way it was delivered really helped parents to predict and understand where they were going, so it put them in a very comfortable space as well. (…) Because every CLANGER is quite a repetitive process and it's unpicking the meanings, lots of self-reflection and we were using the collated feedback to constantly cross-reference and compare... (LF, focus group)

For peer review only - http://bmjopen.bmj.com/site/about/guidelines.xhtml

I found it hard work… and it was very intense and it was more counselling… because people were really disclosing lots of things about their lives and that way… the impact on us as facilitators was more intense experience… and whilst I think we didn't have anything we couldn't handle between us, it actually became more than the course is meant to be. (LF, focus group)

I think one of the things that I definitely felt was the relationship between the trainer and the assistant facilitator is amazing and I definitely feel like the absolute bonus is I've gained a really good friend who I think going on it together actually benefits our health and wellbeing, just delivering it together. It is really positive. (AF, focus group)

(see supplementary document 11 for additional quotes/comments)

Box 5. Selected quotes illustrating participants' experiences

2.1. Motivation to participate and expectations

I feel that being a parent-carer is really draining on your mental health and also I wanted to try and get healthy anyway, especially with it being January. (…) a bit of knowledge maybe about the way I feel and maybe a bit less guilt, the fact that I feel that I need to take time out for myself. (P13, control)

I just thought it could be useful because a lot of the time I find as a parent carer all the focus is on the children which is obviously the main thing but the parents need to be healthy and happy to give the children the best. (P8, intervention)

2.2. Experiences of the programme and its impact

It was fantastic. I think it was really well done. (…) I think it's changed my life. (…) I think the best thing was going to the group, meeting everybody in the group, just meeting like-minded people, having a break from life and being in a different place for a while, reflecting on aspects of my life that I didn't really think about or had put away, and I learnt more about myself. (P5, intervention)

Meeting people who understand and 'get it ' reduced my isolation. Having parent carers as facilitators helped a lot. Having 12 structured sessions made me commit to it and focus on my own health. Having structured fun informative sessions helped a lot - I definitely went through a process of change. The use of humour was really important to me - and learning to be more resilient. (End-of-programme feedback form).

A lot of it is very common sense stuff. (…) It's like a revelation without it being a revelation, because you know all that stuff, but you don't take the time to think about it and it was just very much about focusing on us and improving things for ourselves before we can do it for everyone else really. (P3, intervention)

2.3. Views on online materials

Initially I was curious and then I think it dwindled off because I felt like the course was covering everything so thoroughly that would it add anything to what I had already had? (P4, intervention)

For peer review only - http://bmjopen.bmj.com/site/about/guidelines.xhtml

What was there was absolutely fantastic and I think the videos were a good way of doing it because I am better at learning through watching the videos and being able to go back to them rather than just having loads and loads of information to read… (P18, control)

I found doing the online course quite isolating. Personally, I would have made more changes if I had been in the group. (…) I have ticked the box, I have done it (…) I found myself thinking about everything else that I should be doing, whereas if I had gone to the group and they were watching that video… you are switched on to be watching that video, whereas for me, I was there thinking, 'Okay, I have got to do this, so I will sit down and do this… and then my mind… at one point, I was so tired that I was thinking, 'Oh gosh, I have got to go and make lunch'… (P18, control)

2.4. Factors influencing ability to engage with and benefit from the programme

…on some days you are impacted by inadvertent events and there is no relaxation, there is five hours' sleep. That's just the way it is. (P14, control)

I have some really quite big social care needs and health care needs of my own that are not being met and so it is much more difficult to sit there going, "Ah well, have I eaten properly today?" But those things don't exist so it's better than nothing. (…) But I think it's working really well for some people. Yes, I can see that other members of the group were getting masses more out of it than I was. So I think if it's the right thing for you then it's clearly way more beneficial. (P2, intervention)

References

1. Barlow J, Cullen-Powell L, Cheshire A. Psychological well-being among mothers of children with cerebral palsy. Early Child Dev Care. 2006;176(3-4):421-8.
2. Brehaut JC, Kohen DE, Raina P, et al. The health of primary caregivers of children with cerebral palsy: how does it compare with that of other Canadian caregivers? Pediatrics. 2004;114(2):e182-e91.
3. Brehaut JC, Kohen DE, Garner RE, et al. Health among caregivers of children with health problems: Findings from a Canadian population-based study. Am J Public Health. 2009;99(7):1254-62.
4. Emerson E. Mothers of children and adolescents with intellectual disability: social and economic situation, mental health status, and the self-assessed social and psychological impact of the child's difficulties. J Intellect Disabil Res. 2003;47(4-5):385-99.
5. Gallagher S, Phillips AC, Oliver C, et al. Predictors of psychological morbidity in parents of children with intellectual disabilities. J Pediatr Psychol. 2008;33(10):1129-36.
6. Lach LM, Kohen DE, Garner RE, et al. The health and psychosocial functioning of caregivers of children with neurodevelopmental disorders. Disabil Rehabil. 2009;31(9):741-52.
7. Murphy NA, Christian B, Caplin DA, et al. The health of caregivers for children with disabilities: caregiver perspectives. Child Care Health Dev. 2007;33(2):180-7.
8. Oelofsen N, Richardson P. Sense of coherence and parenting stress in mothers and fathers of preschool children with developmental disability. J Intellect Dev Disabil. 2006;31(1):1-12.
9. Olsson MB, Hwang C. Depression in mothers and fathers of children with intellectual disability. J Intellect Disabil Res. 2001;45(6):535-43.
10. Singer GH, Floyd F. Meta-analysis of comparative studies of depression in mothers of children with and without developmental disabilities. Am J Ment Retard. 2006;111(3):155-69.
11. Arim RG, Miller AR, Kohen DE, et al. Changes in the health of mothers of children with neurodevelopmental disabilities: An administrative data study. Res Dev Disabil. 2019;86:76-86.

For peer review only - http://bmjopen.bmj.com/site/about/guidelines.xhtml

12. Lee M, Park C, Matthews AK, et al. Differences in physical health, and health behaviors between family caregivers of children with and without disabilities. Disabil Health J. 2017:565-70.
13. Tong HC, Haig AJ, Nelson VS, et al. Low back pain in adult female caregivers of children with physical disabilities. Pediatr Adolesc Med. 2003;157(11):1128-33.
14. Hammond P. Staying Alive: How to Get the Best Out of the NHS - advice from a doctor. London: Quercus; 2015. 320 p.
15. Bartholomew Eldredge LK, Markham CM, Ruiter RA, et al. Planning health promotion programs: an intervention mapping approach. 4th ed. San Francisco: Jossey-Bass; 2016.
16. Borek AJ, McDonald B, Fredlund M, et al. Healthy Parent Carers programme: development and feasibility of a novel group-based health-promotion intervention. BMC Public Health. 2018;18(1):270.
17. Bjornstad G, Wilkinson K, Cuffe-Fuller B, et al. Healthy Parent Carers peer-led group-based health promotion intervention for parent carers of disabled children: protocol for a feasibility study using a parallel group randomised controlled trial design. Pilot Feasibility Stud. 2019;5(1):1-13.
18. Moore GF, Evans RE, Hawkins J, et al. From complex social interventions to interventions in complex social systems: Future directions and unresolved questions for intervention development and evaluation. Evaluation (Lond). 2019;25(1):23-45.
19. Moore GF, Audrey S, Barker M, et al. Process evaluation of complex interventions: Medical Research Council guidance. BMJ. 2015;350:h1258.
20. O'Cathain A, Croot L, Duncan E, et al. Guidance on how to develop complex interventions to improve health and healthcare. BMJ Open. 2019;9(8):e029954.
21. Craig P, Di Ruggiero E, Frohlich KL, et al. Taking account of context in population health intervention research: guidance for producers, users and funders of research. NIHR Journals Library. 2018.
22. O'Cathain A, Hoddinott P, Lewin S, et al. Maximising the impact of qualitative research in feasibility studies for randomised controlled trials: guidance for researchers. Pilot Feasibility Stud. 2015;1:32.
23. Reid MJ, Webster-Stratton C, Beauchaine TP. Parent training in head start: a comparison of program response among African American, Asian American, Caucasian, and Hispanic mothers. Prev Sci. 2001;2(4):209-27.
24. van Mourik K, Crone MR, de Wolff MS, et al. Parent Training Programs for Ethnic Minorities: a Meta-analysis of Adaptations and Effect. Prev Sci. 2017;18(1):95-105.
25. Castro FG, Barrera M, Jr., Holleran Steiker LK. Issues and challenges in the design of culturally adapted evidence-based interventions. Annu Rev Clin Psychol. 2010;6:213-39.

For peer review only

For peer review only - http://bmjopen.bmj.com/site/about/guidelines.xhtml

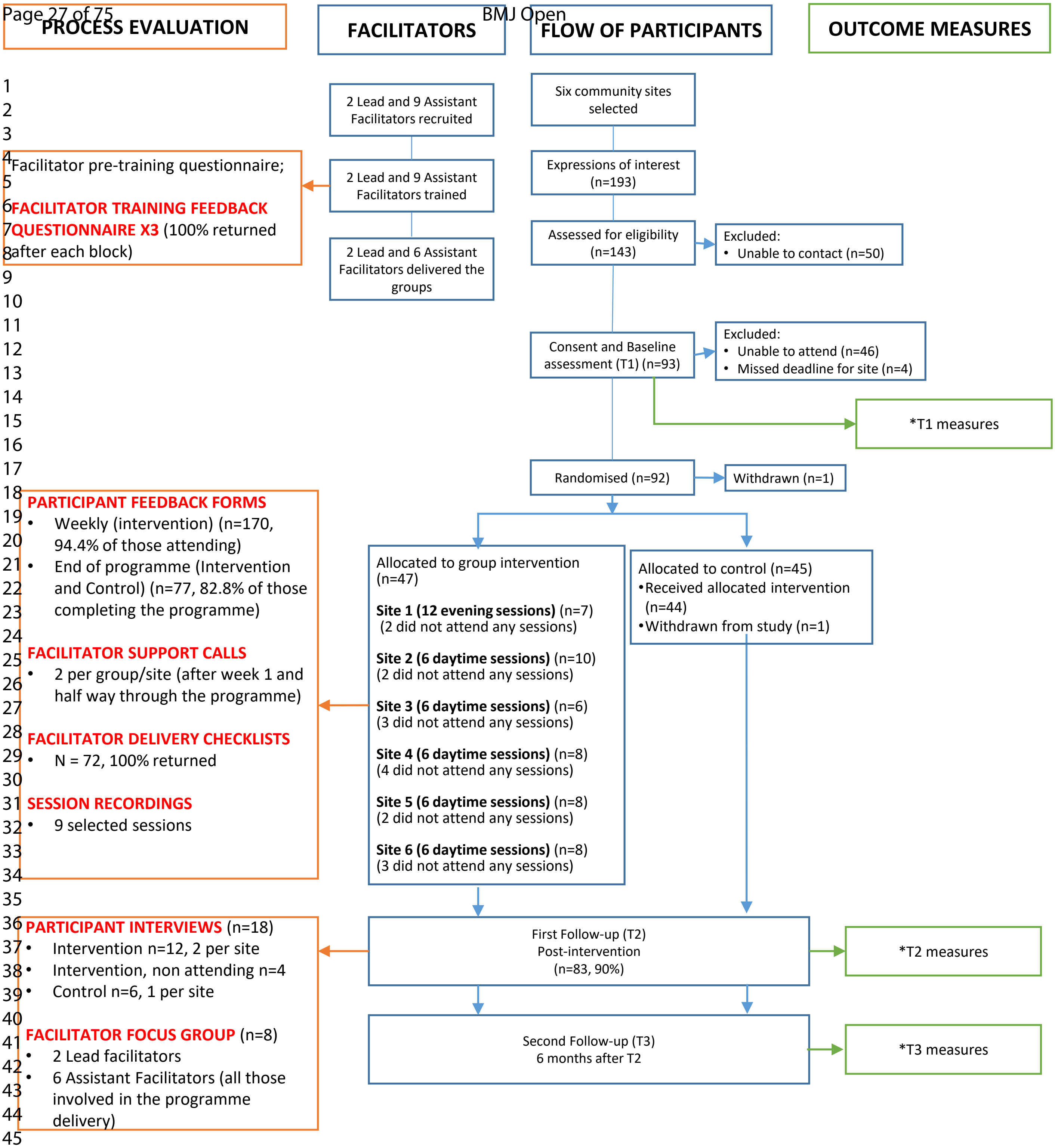

* Bjornstad G, Wilkinson K, Cuffe-Fuller B, et al. Healthy Parent Carers peer-led group-based health promotion intervention for parent carers of disabled children: protocol for a feasibility study using a parallel group randomised controlled trial design. Pilot and Feasibility Studies. 2019;5(1):1-13.

FIGURE 2 - Programme Refinement Process

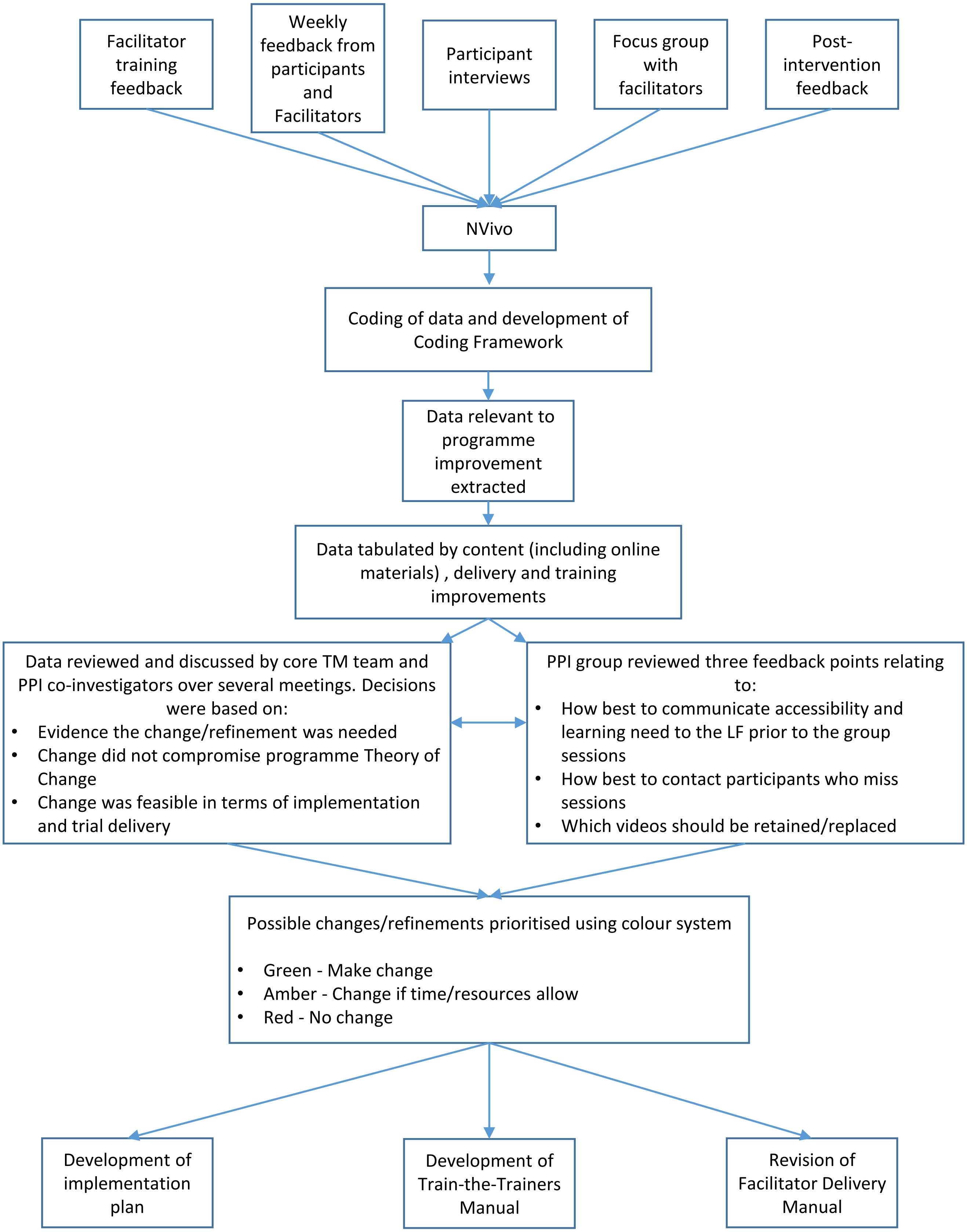

Supplementary Document 1. Healthy Parent Carers intervention details

The details of the intervention are also reported in the intervention development paper (Borek et al., 2018) and the feasibility study protocol (Bjornstad et al., 2019). The results of the feasibility study are reported separately. The intervention details below are reported using the checklist for reporting of group-based interventions (Borek et al., 2015).

**INTERVENTION DESIGN**	
1. Intervention source or development methods	Developed based on an intervention mapping approach (Bartholomew et al., 2016), in partnership with parent carers. Details are described in Borek et al., 2018.
2. General setting	Community setting (with 6 sites/venues)
3. Venue characteristics	6 sites/venues, with different characteristics (2 special schools, 1 children's hospice, 1 Parent Carer Forum premises, 1 adult learning community venue, and 1 hotel regularly used for parent carer meetings).<br><br>All rooms set up to enable interaction between participants (i.e. facing each other).
4. Total number of group sessions	6 or 12 sessions
5. Length of group sessions	6 daytime sessions of 4 hours (2 modules per session)<br>or 12 evening sessions of 2 hours (1 module per session)
6. Frequency of group sessions	Weekly
7. Duration of the intervention	6 or 12 weeks
**INTERVENTION CONTENT**	
8. Change mechanisms or theories of change	The intervention logic model and intended mechanisms of change are reported in Borek et al., 2018. In brief, the intervention aimed to increase knowledge of health-related behaviours, skills in making health-related changes, improve attitudes towards change, increase self-efficacy, and provide social support to make changes, leading to behaviour change (based on CLANGERS) and psychological change (increased empowerment and resilience), leading to improved health and wellbeing.
9. Change techniques	• Provide/exchange information about behaviour-health link<br>• Provide/exchange instructions on practical strategies<br>• Prompt barrier identification and problem solving<br>• Prompt practice<br>• Prompt specific goal setting (for behaviours)<br>• Prompt use of prompts and rewards<br>• Prompt self-monitoring<br>• Prompt goal/progress review (behaviours)<br>• Provide general support and encouragement<br>• Provide opportunities for social comparisons
10. Session content	12 modules:<br>1. Introduction to health and wellbeing<br>2. Introduction to CLANGERS<br>3. Connect<br>4. Learn<br>5. be Active

For peer review only

	6. take Notice<br>7. Give<br>8. Eat well<br>9. Relax<br>10. Sleep well<br>11. Managing stress<br>12. Keeping healthy
11. Sequencing of sessions	Yes – sessions delivered sequentially (as above)
12. Participants' materials	In the sessions: goal setting and CLANGERS diary sheets given to participants, and other materials as parts of group activities.<br><br>Online Healthy Parent Carers materials (written information for each module, links to videos watched in the sessions)
13. Activities during the sessions	Each session started with a 'warm-up' activity and a review of the past week. This was followed by discussions focused on the topic of each module (e.g. 'what does it mean to you to relax? How do you relax? Why is relaxing important? What might stop you from, or help you, relax?). For each module there was a practical activity to help illustrate the key messages or the CLANGERS (e.g., colouring, 'a day in life of a parent carer', a mindful walk). For details, see Borek et al., 2018.
14. Methods for checking fidelity of delivery	Checklists were completed by the facilitators after each session; a sample of audio-recordings of group sessions were checked using the checklist independently (double-checking) by two researchers. Details are reported separately.
**PARTICIPANTS**	
15. Group composition	Parent carers, all female, except for two groups one of which had one male carer, the other of which had two. Ages ranged from 26-71 years (mean 42.5 years)
16. Methods for group allocation	Participants were recruited locally for each site (then randomised to the group intervention or control).
17. Continuity of participants' group membership	The same participants remained in the same group for the duration of the intervention.
18. Group size	7-10 participants were assigned per group. The attendance varied between sessions and groups (lowest attendance being 2 participants over 6 sessions). Details of attendance are reported separately.
**FACILITATORS**	
19. Number of facilitators	2 facilitators per group: one lead facilitator (LF) and one assistant facilitator (AF).<br><br>Overall 2 LF and 6 AF delivered the programme
20. Continuity of facilitators' group assignment	The same pairs of LF and AF facilitated the same groups.
21. Facilitators' professional background	LF: experienced facilitators of training for parent carers delivered through the Council for Disabled Children (e.g., the Expert Parent Programme).

For peer review only

	AF: three had some experience of facilitating parent carer groups and/or training.
22. Facilitators' personal characteristics	All facilitators were parent carers. Both LF were female and one of the six AFs was male.
23. Facilitators' training in intervention delivery	LF received 4 (2 x 2) days of training in delivering the programme; one block of 2 days of these were delivered together for lead and assistant facilitators, LFs and AFs also received one day of refresher training.
24. Facilitators' training in group facilitation	LF were trained and experienced in group facilitation as part of their facilitator/trainer roles for the Council of Disabled Children.<br><br>All facilitators received an overview of facilitating groups and managing group dynamics as part of the training in delivering the intervention.
25. Facilitators' materials	Facilitators delivered the sessions using a manual with instructions outlining all session activities.
26. Intended facilitation style	Participant-centred, interactive, and discussion-based (not didactic).

References:

Bartholomew Eldredge LK, Parcel GS, Kok G, Gottlieb NH, Fernandez ME. *Planning health promotion programs: an intervention mapping approach*. 4th ed. San Francisco: Jossey-Bass; 2016.

Bjornstad G, Wilkinson K, Cuffe-Fuller B, Fitzpatrick K, Borek A, Ukoumunne OC, Hawton A, Tarrant M, Berry V, Lloyd J, McDonald A. Healthy Parent Carers peer-led group-based health promotion intervention for parent carers of disabled children: protocol for a feasibility study using a parallel group randomised controlled trial design. *Pilot and Feasibility Studies*. 2019;5(1):1-3.

Borek AJ, Abraham C, Smith JR, Greaves CJ, Tarrant M. A checklist to improve reporting of group-based behaviour-change interventions. *BMC Public Health*. 2015;15(1):963.

Borek AJ, McDonald B, Fredlund M, Bjornstad G, Logan S, Morris C. Healthy Parent Carers programme: development and feasibility of a novel group-based health-promotion intervention. *BMC Public Health*. 2018;18(1):270.

For peer review only

Bjornstad *et al. Pilot and Feasibility Studies* (2019) 5:137
https://doi.org/10.1186/s40814-019-0517-3

Pilot and Feasibility Studies

STUDY PROTOCOL Open Access

Healthy Parent Carers peer-led group-based health promotion intervention for parent carers of disabled children: protocol for a feasibility study using a parallel group randomised controlled trial design

Gretchen Bjornstad[1], Kath Wilkinson[1], Beth Cuffe-Fuller[1], Katharine Fitzpatrick[1], Aleksandra Borek[2], Obioha C. Ukoumunne[3], Annie Hawton[4], Mark Tarrant[3], Vashti Berry[3], Jenny Lloyd[3], Annabel McDonald[5], Mary Fredlund[5], Shelley Rhodes[6], Stuart Logan[1] and Christopher Morris[1*]

Abstract

Background: Parent carers of disabled children are at increased risk of mental and physical health problems. They often experience challenges to maintaining good health which have implications for their well-being and their ability to care for their children. In response to these needs, researchers and parent carers developed the Healthy Parent Carers (HPC) programme. It is a peer-led, group-based intervention that promotes behaviours associated with health and well-being. The aims of this trial are to assess the acceptability of the HPC programme and the feasibility of its delivery in the community and to assess the feasibility and acceptability of the design of the definitive trial to evaluate the programme's effectiveness and cost-effectiveness.

Methods: We will establish six research sites and train facilitators to deliver the manualised intervention. Parent carers of children with special educational needs and disabilities will be individually randomised, stratified by group delivery site, to either take part in a group programme and online resources (intervention) or to receive access to the online resources only (control). Measures of mental health; well-being; health-related quality of life; health behaviours; patient activation; protective factors such as resilience, social connections, and practical support; and use of health care, social care, and wider societal resources will be collected before randomisation (baseline), immediately post-intervention, and 6 months later. Recruitment of participants, adherence to the programme, and the dose received will be assessed. Group sessions will be audio-recorded to evaluate the fidelity of delivery and participant engagement. Participants' and facilitators' feedback on the programme content and delivery, their experience, and the acceptability of the outcome measures and trial design will be collected through feedback forms, interviews, and focus groups.

Discussion: This trial will assess whether the programme delivery and evaluative trial design are feasible, to inform whether to progress to a definitive randomised controlled trial to test the effectiveness and cost-effectiveness of the Healthy Parent Carers programme.

(Continued on next page)

* Correspondence: christopher.morris@exeter.ac.uk
[1]Peninsula Childhood Disability Research Unit (PenCRU) and National Institute for Health Research Applied Research Collaboration South West Peninsula (PenARC), University of Exeter Medical School, University of Exeter, St. Luke's Campus, Heavitree Road, Exeter EX1 2LU, UK
Full list of author information is available at the end of the article

© The Author(s). 2019 **Open Access** This article is distributed under the terms of the Creative Commons Attribution 4.0 International License (http://creativecommons.org/licenses/by/4.0/), which permits unrestricted use, distribution, and reproduction in any medium, provided you give appropriate credit to the original author(s) and the source, provide a link to the Creative Commons license, and indicate if changes were made. The Creative Commons Public Domain Dedication waiver (http://creativecommons.org/publicdomain/zero/1.0/) applies to the data made available in this article, unless otherwise stated.

(Continued from previous page)
Trial registration: ISRCTN, ISRCTN151144652, registered on 25 October 2018; ClinicalTrials.gov, NCT03705221, registered on 15 October 2018.

Keywords: Behaviour change, Well-being, Resilience, Peer support, Patient and public involvement, Disabled children, Parents, Carers

Background

There are an estimated 960,000 disabled children in the UK, which is 7.3% of the population of children aged 0–18 years [1]. Parent carers of disabled children commonly report higher levels of stress and depression [2–11] and poorer physical health [3, 4, 7, 8, 12–14] than parents of typically developing children. Population-based studies suggest these health problems persist and may worsen over time [15]. These problems have implications for their ability to care for their children.

Parent carers often find the demands of caregiving have a negative impact on their physical and emotional health. Nevertheless, it is important to acknowledge that not all parents of disabled children report that their child's difficulties negatively affect their psychological or physical health [5], and in fact, some report positive impacts [8, 16]. Indeed, some parent carers may perceive a high burden with looking after a child with a relatively 'mild' condition whereas others, whose child may have more severe disabilities, may not perceive caring as high a burden [4].

Some interventions target external factors, such as navigating healthcare services [17], while others target levels of stress [18, 19] or emotional and social support [20]. A systematic review of psychological therapies for parents of children with chronic illness suggested promising results in terms of improved parent mental health, particularly for problem-solving therapy [21]. No benefits were found for cognitive behavioural therapy or family therapy on parent outcomes; however, the quality of the evidence was low and analyses were limited by lack of data available to the reviewers. A systematic review of mindfulness interventions for parents of children with autism indicated potentially positive effects on parents' stress levels and psychological well-being, with studies reporting good attendance and retention in 8-week programmes [22]. There is growing evidence that groups can facilitate change processes beneficial to health and well-being [23, 24] by enabling the formation of strong psychological connections and/or social identification with other group members which can enhance engagement, and thus possibly increase the interventions' effectiveness [25, 26].

The idea for this research came directly from parent carers who had been involved in a study evaluating peer support for parent carers [20]. They wanted to extend the benefits of emotional support to specific strategies to improve health and well-being. Researchers and parent carers in the Peninsula Childhood Disability Research Unit (PenCRU) Family Faculty co-created the Healthy Parent Carers (HPC) programme [27].

Previously, we tested the principle and acceptability of a 6-week intervention programme with one group of seven parent carers, delivered by the intervention developers. The intervention was developed using Intervention Mapping [28] and extensive stakeholder engagement and is described in detail in a separate paper [29]. Participants had children with various conditions including autism, cerebral palsy, and acquired brain injury. Retention of participants in our preliminary study was high with all staying until the end of the 6-week programme and 2-month follow-up. Participants' and facilitators' feedback were positive, indicating the intervention was feasible to deliver and acceptable to, and valued by, participants. The intervention content and delivery methods were refined following feedback, and the manual was updated.

This feasibility trial will provide information that will be used to determine whether to progress to a definitive trial of the HPC programme, which would have the following objectives:

1) To determine whether the peer-led, group-based HPC programme is more effective at improving health and well-being compared to providing online information only
2) To estimate the costs of delivering the HPC programme, and the cost-effectiveness of the programme, versus the provision of online information
3) To understand how the Healthy Parent Carers intervention is working, for whom, and in what context to inform the implementation of the programme should it be shown to be effective

The current trial aims to assess the acceptability of the HPC programme and the feasibility of its delivery in the community, as well as the feasibility and acceptability of the design of the definitive trial in order to evaluate whether a fully powered randomised controlled trial is warranted and to determine the optimal trial design.

For peer review only - http://bmjopen.bmj.com/site/about/guidelines.xhtml

Methods

Objectives

This trial has two overarching aims:

1. To evaluate whether the programme can be delivered in the community by facilitators other than the developers, specifically to:
   (a) Assess the feasibility of establishing venues, and identifying and training group facilitators to be in a position to deliver the intervention
   (b) Assess the fidelity of intervention delivery in terms of format, content, and quality
   (c) Assess the experience and engagement of participants, facilitators, and trainers
   (d) Assess the programme attendance
2. To provide information necessary to design a definitive randomised controlled trial, specifically to:
   (a) Assess the feasibility of recruiting participants in different sites
   (b) Assess the acceptability of randomisation of parent carers
   (c) Assess the attrition and completion and proportion of any missing data in questionnaire measures
   (d) Appraise the performance of candidate health and well-being outcome measures in terms of acceptability to participants, and feasibility and interpretability for researchers
   (e) Estimate the variability (standard deviation) and the level of clustering within programme delivery groups in the intervention arm to help inform the sample size calculation for the definitive trial
   (f) Test the proposed cost-effectiveness framework for a future randomised trial

This will help inform whether to progress to a definitive randomised controlled trial to test the effectiveness and cost-effectiveness of the programme and provide information necessary to design the trial.

Design

A feasibility trial using a parallel group randomised controlled trial design will be carried out in six sites in the southwest of England. Participants will be randomly allocated to receive the group-based programme and access to online programme resources or to a control group receiving access to the online resources only. Data collection will take place at three time points in both trial arms at baseline (prior to randomisation), immediately post-intervention, and 6 months later. As the intervention can be delivered over 6 or 12 weeks, the post-intervention data collection time point will vary relative to randomisation but will be consistent in terms of the amount of time passing after completion of the intervention. Participants in the control arm in each randomised site will complete measures at the same time as participants in the intervention arm for that site. The two arms will therefore be balanced in terms of the timing of outcome measures. The trial design and the flow of participants through the trial are illustrated in the trial flow chart and SPIRIT figure (Fig. 1 and Table 1). The SPIRIT checklist is provided as an additional file (see Additional file 1).

Public involvement

This project has a strong ethos of parent carer and stakeholder engagement from inception. The public involvement in this project will ensure the following: (a) the research is conducted in an acceptable manner, (b) the research outputs are relevant and useful to parents of children with special educational needs and disabilities, and (c) our dissemination materials and methods are appropriate and accessible.

Over 40 parents of children with a range of conditions from the PenCRU Family Faculty public involvement group have participated in a study-specific working group since 2014. Our Stakeholder Advisory Group (SAG) includes representatives from the local authority, public health, parent carer forums, relevant charities, and special schools.

Parent carers have been involved in all stages of developing the intervention and designing the feasibility trial including:

a) Proposing the idea for the project based on their needs and experiences
b) Co-designing and refining the intervention and training content and delivery methods
c) Providing feedback on research methods including the selection of the comparison conditions
d) Advising on the content and form of the Resource Use Questionnaire for use as part of the cost-effectiveness framework
e) Contributing to interpreting and disseminating the findings of the previous study
f) Interviewing and hiring research staff
g) Discussing and advising on the design of the feasibility trial
h) Recommending responses to peer reviews when applying for funding.

Study setting and location

In collaboration with our SAG, we will identify six venues (e.g. schools, community centres, adult and community learning venues) where it is possible to establish and host a group. We will agree days, times, durations

For peer review only

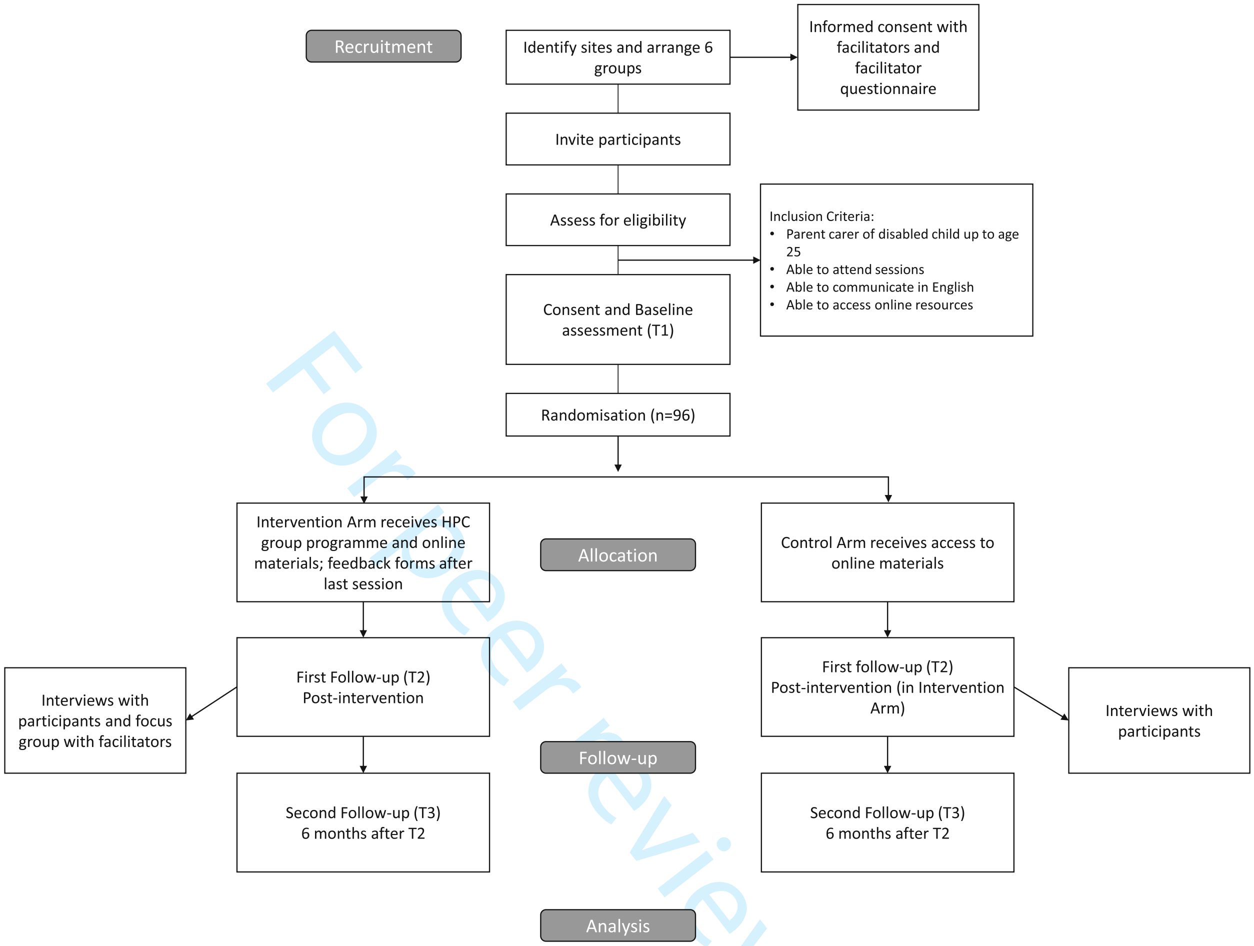

Fig. 1 Trial flow chart

For peer review only

and frequencies of sessions, and local named organisers for each venue.

Sample size

We aim to recruit 96 participants, to be allocated on 1:1 ratio to intervention and control. This is a large enough sample to estimate the percentage providing data at follow-up (assumed to be 80%—76 participants) with a margin of error of 10 percentage points based on the lower bound of the 95% confidence interval. Assuming that 38 participants are followed up in each trial arm, this will be large enough to estimate the standard deviation for continuous outcomes in each arm within 29% of its true value based on the upper bound of the 95% confidence interval. Finally, 76 participants at follow-up *are* large enough to estimate a correlation coefficient of 0.5 between baseline and follow-up scores for a continuous outcome with a margin of error of 0.19 based on the lower bound of the 95% confidence interval. We will randomise a minimum of *8* and *a* maximum of 24 participants at each of the 6 sites. This will mean that 4–12 participants will be allocated to each of the intervention and control trial arms at each site. We expect the ideal group size to be between 6 and 12 people but are allowing for potential attrition and variation in recruitment between sites.

Inclusion criteria

People meeting the inclusion criteria are (1) primary carers of children with additional needs and/or disabilities (participants who self-identify as primary carers are eligible; the child can be up to 25 years old consistent with the current Department of Health and Department of Education Special Educational Needs and Disability (SEND) legislation in England and The Children's Act; no named diagnosis is necessary, and we are not limiting to specific conditions), (2) willing and able to attend the programme group meeting session(s) on arranged dates/times, and (3) able to access online information.

Table 1 Schedule of enrolment, interventions, and assessments

**TIMEPOINT**	**Enrolment (T1)**			**Allocation**	**Post-allocation**	**Post-intervention (T2)**	**6 months post-intervention (T3)**
**ENROLMENT:**							
Eligibility screen	X						
Informed consent		X					
Allocation				X			
**INTERVENTIONS:**							
Group Healthy Parent Carers programme					—	—	
Online Healthy Parent Carers resources					—	—	
**ASSESSMENTS:**							
Demographics (15 items)			X				
About My Child -19 (19 items)			X				
Warwick-Edinburgh Mental Well-being Scale (WEMWBS) (14 items)			X			X	X
EuroQol-5 Dimensions (EQ-5D-5L) (5 items)			X			X	X
Patient Health Questionnaire-9 (PHQ-9) (9 items)			X			X	X
Health Promoting Activities Scale (HPAS) (8 items)			X			X	X
Patient Activation Measure (PAM) (13 items)			X			X	X
Parents' Assessment of Protective Factors (PAPF) (36 items) "Parent Statements"			X			X	X
ICEpop CAPability measure for Adults (ICECAP-A) (5 items)			X			X	X
Service and Resource Use questionnaire: Healthy Parent Carers (31 items)			X			X	X
Adverse event assessments					X	X	X
Compliance (attendance)					X	X	
Feedback Form (14 items)						X	
Interviews and focus groups						X	

For peer review only

Exclusion criterion

Potential participants who are not able to communicate in English are excluded. This is necessary because the programme has not yet been translated into other languages.

Recruitment

We will advertise the study in several ways. Press releases and interviews in local television and/or radio will be used to publicise the study. Members of our SAG will be asked to advertise the project to their members through their email lists and networks. We will also share study adverts and information via social media and through the PenCRU Family Faculty email list, asking *the* members to share in their networks. We will liaise with Information and Advice Services in each locality, staff in social services, and Special Educational Needs Coordinator *(*SENCOs*)* in schools in each study site to target potentially more isolated parent carers. We will also recruit participants at events for parent carers at the study venues and other venues in the southwest where interested parent carers can discuss the project with the research team. This recruitment strategy uses many different approaches because parent carers do not all access the same services and not all parent carers are connected with their local parent carer forums. We will ask all participants how they heard about the study during screening, and this information will be recorded to assess whether some recruitment methods may be more effective than others. However, we are mindful that while some methods may not result in large numbers of recruits, they may help us to reach parent carers who are more isolated and more in need of support and, as such, will be seen as important methods to take forward in a definitive trial.

Interested parent carers will contact the researchers. There will be *a* telephone or face-to-face screening to check *the* eligibility, understanding of the study, and to answer any questions. A researcher will meet each potential participant individually. Those who want to participate will sign a consent form and complete baseline questionnaires online using an electronic patient-reported outcome (ePRO) system with a researcher on hand for support as necessary [30]. Reasons for not consenting to participate will be recorded if provided by those who decline.

Allocation to trial arms

When recruitment is completed at each site, we will proceed to randomisation. Each of the six programme groups will constitute a study site, with participants who choose that group being randomised to either attend the group or receive the online resources only. A computer-generated randomisation sequence will be used to assign the participants in each site to the intervention and control arms. A block randomisation scheme will be implemented to ensure balance in the number of participants allocated to each trial arm, stratified by group delivery site. The allocation sequence will be concealed from researchers using an online central randomisation service setup and maintained by the Exeter Clinical Trials Unit (UKCRC Registration ID 65). Blinding will not be used in this trial.

All participants will receive an email and letter indicating the result of randomisation. The participants randomly allocated to the intervention arm will be sent details of the group sessions and be contacted by their lead facilitator before the first group session. Participants in both arms will receive a link to the online programme resources and instructions on the web page. We will monitor the number of participants who refuse participation and record their reasoning (if they wish to share it) to gauge the acceptability of our trial design.

Intervention

The group-based programme was developed using Intervention Mapping approach [28]. Full details of the intervention, including its development, logic model, and content (e.g. activities, behaviour change techniques), are available in a previous publication [29]. In brief, the programme aims to expand parent carers' social networks and provide social support from peers with a shared sense of social identity alongside targeted activities to improve parent carers' confidence, motivation, self-efficacy, and empowerment, thus creating the conditions for change necessary for them to feel able to make their own plan to prioritise healthy behaviours for themselves.

The programme content is based around a set of universal and evidence-based actions (called CLANGERS) associated with health and well-being. CLANGERS stands for Connect, Learn, be Active, Notice, Give, Eat well, Relax and Sleep [31]. The 'CLANG' component comprises the 'Five Ways to Wellbeing' based on the evidence from the foresight project on Mental Capital and Wellbeing [32]. Each of these behaviours is potentially more difficult for parent carers.

The programme content is organised into 12 modules lasting 2 h each. The modules can be delivered weekly over 6 sessions (comprising 2 modules per session) or 12 sessions (1 module per session). Our Family Faculty PPI working group suggested that offering either 6 longer sessions in the daytime or 12 shorter evening sessions would be reasonable for most parent carers. One or both options will be offered per area in order to maximise recruitment and to reflect likely real-world delivery in community settings, with 6 groups being delivered in the study in total. If uptake is very low for a particular

For peer review only - http://bmjopen.bmj.com/site/about/guidelines.xhtml

site, the delivery model may be adapted during recruitment to increase numbers.

Facilitators

The lead facilitators of the group-based programme will be experienced facilitators of the 'Expert Parent Programme' courses created by the Council for Disabled Children (CDC) with funding from the Department of Health. The facilitators are parent carers. CDC has a selection process, trains, and provides supervision for their facilitators to ensure that they facilitate the groups effectively. Their nationwide network of over 70 facilitators provides a sustainable model for the implementation of the programme in the future. Facilitators will use the Healthy Parent Carers Facilitator Manual that includes module outlines, content, timings, activities, and resources needed. The Facilitator Manual also includes safeguarding procedures for the facilitator to follow in case of any adverse events such as suicidal ideation or disclosure of safeguarding issues.

There will also be an assistant facilitator in each group to assist the facilitator in sessions. We will recruit a local parent carer for this role using a person specification detailing the required personal qualities and skills and a selection process.

We will provide training for lead and assistant facilitators. The training will take place over 4 days for lead facilitators and 2 days for assistant facilitators; it will be delivered by researchers and the parent carer co-investigators who co-developed the programme and facilitated the group in our previous study. Lead facilitators will receive ongoing supervision and support through the CDC and support from the research team. Conference calls with facilitators, assistants, and researchers will be convened to reflect on delivery of the sessions. These discussions will inform intervention design and training needs and provide a forum to share ideas and ways to address any challenges arising.

The Healthy Parent Carers online resources are part of the intervention. They reflect the content of the group sessions, provide space to write down reflections, and prompts to set specific goals and for self-monitoring of CLANGERS-related behaviours.

If the participants in the group programme miss a session, they will be telephoned by the facilitator, who will summarise the session and encourage the participant to reflect on their week, read the section of the HPC online resources, and set their weekly goals.

Control

Participants in the control arm will receive access to the HPC programme resources online with instructions. Risk of contamination between participants allocated to each arm is low because the intervention is predicated on participants developing a shared social identity as members of the HPC group. Participants in both arms will be asked whether they have had contact with participants in the other arm of the trial as part of a post-intervention feedback form.

Data collection

Study records

We will record the data on the feasibility of recruitment, including how many people respond to the adverts, how they heard about the study, reasons for not taking part for those who decline, and how many are successfully recruited. We will record delivery setting, delivery model, attendance, attrition, and reasons for missed sessions or withdrawal from the study. We will also monitor how long it takes to accrue the target number of participants at each site and at what point in the trial process any participants withdraw.

Sample characteristics

Demographic data will include gender, ethnicity, parent relationship status, number of children, employment status, level of education, income, housing status, and age, gender, and diagnosis (if any) of their disabled child. We will also collect information about their disabled child's functioning and health complexity using the About my Child measure (AMC-19) [33]. We will also use participants' postcodes to link with the Indices of Multiple Deprivation as an indicator of deprivation relative to England and Wales in the area where participants live [34].

Outcome measures

Participants will be asked to complete all measures before randomisation, immediately post-intervention, and 6 months post-intervention, regardless of attendance or engagement with the interventions. Based on the recommendations from our Family Faculty PPI working group, the measures will be available to complete online, using a computer, smartphone, or tablets. The Exeter Clinical Trials Unit will set up an online platform for participants to access and complete the measures. Participants may request to complete the measures on paper if they wish. Any measures completed on paper will be independently double-entered by two researchers. These requests will be monitored to track preferences for online- versus paper-based measures.

Two members of our Family Faculty PPI working group have tested the applicability and time to complete the measures (45 min). A £25 shopping voucher will be posted to participants as acknowledgement for completing measures at each time point.

The measures will comprise:

For peer review only - http://bmjopen.bmj.com/site/about/guidelines.xhtml

a) Warwick-Edinburgh Mental Well-being Scale (WEMWBS): The WEMWBS is a 14-item scale used to assess the mental well-being in the general population and in the evaluation of programmes aiming to improve mental well-being [35]. Responses are normally distributed in the general population. WEMWBS has been validated in the UK, Europe, and elsewhere. It has been tested with minority ethnic populations, users of mental health services, and carers. It is sensitive to changes occurring through participation in programmes that promote well-being such as health promotion programmes. A tariff of well-being-adjusted life-year weights is currently being developed for responses on the WEMWBS, which will enable the measure to be used in cost-effectiveness analyses recognised by the National Institute for Health and Care Excellence (NICE) [36].
b) Patient Health Questionnaire-9 (PHQ-9): The PHQ-9 is a 9-item measure that rates the frequency of symptoms and is designed for screening, diagnosing, monitoring, and measuring the severity of depression [37–39]. Categories based on the cutoff scores of 5, 10, 15, and 20 represent none, mild, moderate, moderately severe, and severe depression, respectively. As part of a safeguarding protocol, we will use the Patient Health Questionnaire (PHQ-9) to measure depressive symptoms. The PHQ-9 questionnaire is recommended by NICE to assess depression in adults [40], and its use is highlighted in clinical pathways [41], so the interpretation of scores is widely understood by GPs and primary care staff. There is good evidence, across a range of studies, for the validity, reliability, sensitivity, and specificity of the PHQ-9 for detecting depressive disorders [38, 39]. It can be administered repeatedly to assess change in depression in response to treatment. Question 9 screens for suicidal ideation. If the person scores higher than 0 on question 9, or at any other point discloses suicidal ideation, we will follow the safeguarding protocol.
c) EuroQol 5 Dimensions (EQ-5D-5 L): The EQ-5D-5 L is a measure of health-related quality of life. It consists of five items measuring five dimensions of health-related quality of life (mobility, self-care, usual activities, pain/discomfort, and anxiety/depression) and a vertical visual analogue scale measuring self-rated health [42]. QALY weights can be applied to EQ-5D scores, which can then be used to calculate QALYs, and the cost-per-QALY of the intervention in a future definitive trial. The EQ-5D is NICE's preferred measure for use in health technology cost-effectiveness analyses.
d) Parents' Assessment of Protective Factors (PAPF): The PAPF is a 36-item measure that assesses protective factors identified in the development of the Strengthening Families evidence-based parenting programme [43]. These protective factors are as follows: parental resilience, social connections, concrete support in times of need, and support of children's social and emotional competence. These factors relate to the determinants of change in the logic model for the Healthy Parent Carers Programme.
e) Health Promoting Activities Scale (HPAS): The HPAS is an 8-item measure of a person's estimation of the frequency with which they participate in a range of activities that promote or maintain health and well-being [44, 45]. It was developed for and validated with mothers of children with disabilities.
f) Patient Activation Measure (PAM): The PAM is a 13-item measure that measures the spectrum of skills, knowledge, and confidence in patients and captures the extent to which people feel engaged and confident in managing their own health and care [46, 47]. It has been tested with 100,000 patients with long-term conditions in England to establish the feasibility of using the PAM across the NHS, how activation can inform support for self-management, what support clinicians and commissioners need to use the measure effectively, and whether supporting activation can improve outcomes for patients in the NHS.
g) ICEpop CAPability measure for Adults (ICECAP-A): The ICECAP-A is a 5-item measure of capability, which includes the following aspects of well-being found to be important to adults in the UK: attachment, stability, achievement, enjoyment, and autonomy [48–50]. A set of UK well-being adjusted life-year weights are available for the ICECAP-A, enabling it to be used in economic evaluations.
h) Resource use questionnaire: We developed a study-specific resource use questionnaire in collaboration with parent carers. This includes health, social care, participant, and broader societal resource use, and draws on measures in the Database of Instruments for Resource Use Measurement (DIRUM) [51].

Process evaluation

In line with the MRC guidance on process evaluations, this study will include a process evaluation that is appropriate for the feasibility testing stage of the development-evaluation-implementation process for this intervention [52]. This process evaluation will allow for the exploration of the feasibility of implementation of the intervention by assessing uptake (recruitment) and retention, participant

For peer review only

engagement, fidelity of delivery (to content and quality), experiences of participants and facilitators, unintended consequences, and contextual factors which may influence experience and delivery.

The following data collection tools will be used to assess fidelity of intervention delivery, participant and trainer characteristics/motivations, trainer knowledge and self-efficacy to deliver the programme, participant engagement with the programme/online materials, and acceptability of the intervention and trial design.

a) Facilitator pre-training questionnaire: We will use a pre-training questionnaire to collect information about facilitators' characteristics, their motivations to take part, relevant background and experience, and expectations of delivering the programme.
b) Facilitator training feedback: Following delivery of the training, we will use a questionnaire to gather the facilitators' feedback about their self-reported knowledge, understanding, skills, and confidence to deliver the intervention and to gather their reflections on the training.
c) Facilitator delivery observations: We will use a checklist to assess lead facilitators' competence to deliver while observing their delivery of the programme content during the facilitator training. The checklist includes key skills and competencies linking to the objectives of the lead facilitator training and will enable trainers and research staff to assess facilitators' readiness to deliver the programme. This will also help to guide and plan additional or future training.
d) Facilitator checklist, records, and support calls: We will use a self-report checklist completed jointly by the facilitators to indicate which content they have covered in each session (adherence), the duration of the sessions (dose), and the participants' engagement. Facilitators will be asked to record attendance at each session. We will also arrange support calls with facilitators to gather more information about how the groups are going and any challenges to delivery.
e) Session recordings: We will audio-record group sessions and will sample two to three recordings from each group to assess fidelity to intervention content, quality of delivery, and participant engagement. A researcher will rate the delivery using the same checklist used by the facilitators after each session. A second researcher will rate one recording per group ($n = 6$, 14%). The two researchers will compare the scoring of the first three groups immediately, and any inconsistencies will be discussed with JL/MT, to ensure there is a clear understanding of the assessment criteria. The scores of the double-coded sessions will be agreed between researchers, and the sessions assessed by the researchers will be compared with the facilitators' scores.
f) Participants' feedback: We will collect feedback from treatment and control arm participants about the programme content and delivery, their experiences, and whether they had contact with participants in the other arm of the trial via feedback forms at the end of each group session for those in the intervention arm and at the end of the programme for those in both trial arms.
g) Participant interviews: We will sample purposively 12 participants (from different groups) in the intervention arm and 6 participants allocated to the control arm across all sites for semi-structured telephone interviews. For the intervention arm, these interviews will explore participants' experiences of, and engagement in, the programme and the group and their views on the group content, activities, and facilitators. All, control and intervention groups, participants will be asked in the interviews about engagement with online resources, perceived impact of the programme and any potential contextual influences, and acceptability of the trial processes and measures. We will also sample up to 4 participants (from different groups) who were allocated to the intervention arm of the trial but did not attend any group sessions to ask them about barriers to attending and whether anything could be done to promote attendance in future groups. All interviews with participants will last approximately 30 min and will be audio-recorded and transcribed verbatim, with names and other personal identifying information changed to protect confidentiality. Interviews will take place as soon as possible after the participants have completed their post-intervention measures and before they complete their 6-month follow-up measures.
h) Focus groups with facilitators: We will invite all lead facilitators and assistant facilitators to a focus group after the end of all groups. The focus group (lasting approximately 2 h) will cover facilitators' experiences of delivering, and engagement with, the programme, views on the programme content, activities and feasibility of programme delivery, facilitator training and skills, group management, and suggestions for improvements. The focus group will be audio-recorded and transcribed verbatim (with any potentially identifiable information anonymised).

For peer review only

Cost-effectiveness framework

We will develop and test a framework for assessing the cost-effectiveness of the intervention in a future randomised trial. We will:

a) Establish methods for estimating intervention resource use and costs (e.g. training of facilitators, facilitators' time, venue hire), in collaboration with the programme facilitators and site representatives
b) Develop a resource use questionnaire in collaboration with parent carers, drawing on measures in the Database of Instruments for Resource Use Measurement (DIRUM) repository [51]
c) Assess the acceptability to parent carers of the EQ-5D-5 L, the ICECAP-A, and the WEMWBS, judged by missing data and measurement properties [53].

Data analysis

Statistical/quantitative analysis

We will report the number of eligible people who self-refer and the percentage (with 95% confidence intervals (CIs)) of these that are randomised in the trial. These findings will also be reported separately for each type of delivery setting and delivery model to assess whether particular delivery settings or models are more popular and therefore would lead to higher recruitment rates in the subsequent definitive trial. We will also report numbers and percentages of people who heard about the study via different sources, organised into categories.

We will also report the percentage (with 95% confidence intervals) of participants who complete each assessment at each time point as an assessment of the acceptability of the measures and of the feasibility of collecting sufficient data in a definitive trial. We will summarise the characteristics of recruited participants using demographic data to allow for assessment of the representativeness of the sample relative to figures available from the Office for National Statistics on the population in the southwest of England. We will also report the baseline comparability of the trial arms with respect to demographics and outcome measures.

For the intervention arm, we will report the number and percentage of participants that attend each group session with 95% confidence intervals. The percentage of participants that are lost to follow-up at each follow-up point will be reported for each trial arm. Select characteristics (parent carer gender and age, child gender and age, Index of Multiple Deprivation quintile, study site (centre), and baseline scores on outcomes) will be compared between those who are and are not lost to follow-up within each trial arm using descriptive summaries, but no formal statistical tests. Acceptability, judged by missing data of EQ-5D-5 L and ICECAP-A, will help to plan methods for estimation of cost-effectiveness in the future trial.

Means, standard deviations, and the correlation between baseline and follow-up scores on continuous outcomes will be reported to inform the sample size calculation for the subsequent definitive trial.

Level of clustering within groups in the intervention arm will be quantified using the intra-cluster (intra-group) correlation coefficient to inform the sample size calculation for the definitive trial; however, we recognise the relatively small sample size for this purpose, and it will be used alongside information about levels of clustering in published studies of trials of similar group-based interventions in similar settings.

We will compare the outcomes at follow-up between the two trial arms based on the intention-to-treat principle with participants analysed according to the trial arm they were randomised to. Missing data will not be imputed. We will report only confidence intervals for the intervention effect and no p values, in line with the extension to the CONSORT statement for reporting randomised pilot and feasibility studies [54].

Analysis of process data

Descriptive statistics will be reported for the quantitative data collected in delivery observation checklists, facilitator checklists, checklists used to assess intervention session recordings, and participant feedback forms.

Qualitative data collected from feedback forms, interviews, and focus groups will be analysed thematically to provide insights into participants' experiences of the programme, intervention acceptability, and suggestions for improvement, and to enhance understanding of the impact of the intervention and the mechanisms of change in relation to the programme logic model [54]. Data will be analysed using inductive thematic analysis [55] separately for each data source (i.e. feedback comments, interviews, and focus groups), following the same approach. Some issues will emerge as more salient than others and the interpretation of findings will be influenced by the original research objectives as well as the themes emerging directly from the data.

NVivo software (version 12 Pro for Windows, QSR International) will be used to organise and analyse the qualitative data. Initially, two researchers will independently read and code line-by-line a sample of the data and discuss their coding to develop and agree on the coding framework. New codes may be added as the coding proceeds, and the codes and coded data will be reviewed. Codes will be defined, compared to each other, and organised into categories and themes. Attention will be paid to negative, or 'deviant', cases to inform developing themes and interpretation. Short summaries of each interview will be also written to explore how individual

For peer review only

experiences and views of the intervention may differ between participants. The analysis and interpretation of the data will be regularly discussed with the research team. A detailed record will be kept of the analysis process, including definitions of the themes and concepts and their application.

Discussion

We will interpret the findings of this feasibility trial and report the implications for progression to a definitive randomised controlled trial of the HPC programme. This will include any necessary amendments to the intervention content and delivery, as well as the development of a train-the-trainer manual to be used in training future facilitators of the programme. The following indicators of feasibility will be used to determine whether a definitive randomised controlled trial is feasible with the current trial design and procedures:

a) Recruit a minimum of 48 participants, which is the minimum number that will enable all six sites to be randomised and the intervention to be tested
b) Deliver 6 groups in total for the intervention arm, assessed by establishing 6 venues, and identifying and training facilitators, and groups completing the programme curriculum
c) At least 80% of participants completing measures at 6-month follow-up or a clear plan to achieve this in the trial

If any of these indicators are not met, the research team will consider whether a definitive trial may not be feasible or whether changes to the design or procedures and further feasibility testing are needed. The need to translate programme materials into other languages will also be taken into account for a subsequent trial.

A complete and transparent report of the trial will be produced with reference to recommendations of the CONSORT 2010 statement: extension to randomised pilot and feasibility trials, including a CONSORT participant flow chart [54]. The report will be written for publication in a peer-reviewed, open access, academic journal with authorship eligibility determined by following the International Committee of Medical Journal Editors recommendations [56]. A plain language summary of the findings will also be co-produced with members of our Family Faculty public involvement group and sent to trial participants and organisations that help to recruit participants and host the groups. We will consult our Family Faculty and Stakeholder Advisory Group for advice on ways to disseminate the findings.

NHS England's Commitment to Carers states 'Helping carers to provide better care and to stay well themselves will contribute to better lives for those needing care and more effective use of NHS resources' [57]. However, there is currently a paucity of interventions that promote health for parent carers. This feasibility trial and a subsequent definitive trial may have important implications for a public health strategy for parent carers of children with disabilities in the UK. It will also inform research and public health policy internationally, as the higher risk of psychological and physical health problems in parent carers is not limited to the UK.

Project timetable and milestones

The main milestones are as follows. Ethical approval for the study was received on 20 August 2018. The trial was registered on 25 October 2018 (ISRCTN 15144652). Recruitment of participants began on 29 October 2018. The analysis of data on fidelity and process evaluation will be conducted following data collection (summer to autumn 2019). The analyses of outcome measures will be conducted in February 2020. The expected date of completion is June 2020.

Supplementary information

Supplementary information accompanies this paper at https://doi.org/10.1186/s40814-019-0517-3.

Additional file 1. SPIRIT 2013 Checklist: Recommended items to address in a clinical trial protocol and related documents*.

Abbreviations

AMC-19: About my Child measure; CDC: Council for Disabled Children; CLANGERS: Connect, Learn, be Active, Notice, Give, Eat well, Relax, and Sleep; CONSORT: Consolidated Standards of Reporting Trials; DIRUM: Database of Instruments for Resource Use Management; EQ-5D-5 L: EuroQol 5 Dimensions; HPAS: Health Promoting Activities Scale; HPC: Healthy Parent Carers; ICECAP-A: ICEpop CAPability measure for Adults; NIHR: National Institute for Health Research; PAM: Patient Activation Measure; PAPF: Parents' Assessment of Protective Factors; PenCLAHRC: The National Institute for Health Research (NIHR) Collaboration for Leadership in Applied Health Research and Care (CLAHRC) South West Peninsula; PenCRU: Peninsula Cerebra Research Unit; PHQ-9: Patient Health Questionnaire-9; PPI: Patient and public involvement; SAG: Stakeholder Advisory Group; SEND: Special educational needs and disability; WEMWBS: Warwick-Edinburgh Mental Well-being Scale

Acknowledgements

We thank members of the PenCRU Family Faculty and the Stakeholders Advisory Group for their involvement in the development of the intervention and the design of this study.

Study Sponsor

University of Exeter, Research Ethics and Governance Office, University of Exeter, Lafrowda House, St Germans Road, Exeter, Devon, EX4 6TL

Authors' contributions

All authors contributed. CM led the development and preliminary evaluation of the programme and leads the study and public involvement work. GB and CM drafted the initial study design. AB, AM, MF, and CM designed the original programme. OU designed the quantitative analysis plan. AH designed the methods to develop and test a cost-effectiveness framework. AB, CM, VB, JL, MT, KW, and BCF designed the fidelity and process evaluation. KF facilitated public involvement. SR coordinated the CTU involvement in the design and conduct of the project. SL facilitated infrastructure support through National Institute for Health Research Applied Research

Collaboration South West Peninsula. All authors contributed to drafting and refining the study protocol and approved the final manuscript. The study Sponsor is the University of Exeter.

Funding
This paper presents independent research funded by the National Institute for Health Research (NIHR) under its Research for Patient Benefit (RfPB) Programme (Grant Reference Number PB-PG-0317-20044). We also acknowledge support from the National Institute for Health Research Applied Research Collaboration South West Peninsula. The views and opinions expressed in this paper are those of the authors and not necessarily those of the National Institute for Health Research or the Department of Health and Social Care. Delivery of the Healthy Parent Carers programme is funded by Public Health England, Kernow Clinical Commissioning Group, Northern, Eastern and Western Devon Clinical Commissioning Group, and a grant from the National Lottery Community Fund through the Reaching Communities programme. Peer review of the study design was provided during the NIHR funding application process. The funders have had no involvement in the writing of the manuscript and will not have any role during its execution, analyses, interpretation of the data, writing of the report, or the decision to submit the report for publication.

Availability of data and materials
Not applicable

Ethics approval and consent to participate
Ethics approval for the study was received on 20 August 2018 from the University of Exeter Medical School Research Ethics Committee in the College of Medicine and Health (UEMS REC 18/06/174); recruitment is currently ongoing.

Consent for publication
Not applicable

Competing interests
The authors declare that they have no competing interests.

Author details
[1]Peninsula Childhood Disability Research Unit (PenCRU) and National Institute for Health Research Applied Research Collaboration South West Peninsula (PenARC), University of Exeter Medical School, University of Exeter, St. Luke's Campus, Heavitree Road, Exeter EX1 2LU, UK. [2]Nuffield Department of Primary Care Health Sciences, Medical Sciences Division, Radcliffe Observatory Quarter, University of Oxford, Woodstock Road, Oxford OX2 6GG, UK. [3]National Institute for Health Research Applied Research Collaboration South West Peninsula (PenARC), University of Exeter Medical School, University of Exeter, St. Luke's Campus, Heavitree Road, Exeter EX1 2LU, UK. [4]Health Economics Group, University of Exeter Medical School, University of Exeter, St. Luke's Campus, Heavitree Road, Exeter EX1 2LU, UK. [5]PenCRU Family Faculty, University of Exeter Medical School, University of Exeter, St. Luke's Campus, Heavitree Road, Exeter EX1 2LU, UK. [6]Exeter Clinical Trials Unit, University of Exeter Medical School, University of Exeter, St. Luke's Campus, Heavitree Road, Exeter EX1 2LU, UK.

Received: 2 April 2019 Accepted: 15 October 2019
Published online: 23 November 2019

References
1. Blackburn CM, Spencer NJ, Read JM. Prevalence of childhood disability and the characteristics and circumstances of disabled children in the UK: secondary analysis of the Family Resources Survey. BMC Pediatr. 2010;10(21): 1471–2431.
2. Barlow J, Cullen-Powell L, Cheshire A. Psychological well-being among mothers of children with cerebral palsy. Early Child Dev Care. 2006;176(3–4):421–8.
3. Brehaut JC, Kohen DE, Raina P, Walter SD, Russell DJ, Swinton M, O'Donnell M, Rosenbaum P. The health of primary caregivers of children with cerebral palsy: how does it compare with that of other Canadian caregivers? Pediatrics. 2004;114(2):e182–91.
4. Brehaut JC, Kohen DE, Garner RE, Miller AR, Lach LM, Klassen AF, Rosenbaum PL. Health among caregivers of children with health problems: findings from a Canadian population-based study. Am J Public Health. 2009; 99(7):1254–62.
5. Emerson E. Mothers of children and adolescents with intellectual disability: social and economic situation, mental health status, and the self-assessed social and psychological impact of the child's difficulties. J Intellect Disabil Res. 2003;47(4–5):385–99.
6. Gallagher S, Phillips AC, Oliver C, Carroll D. Predictors of psychological morbidity in parents of children with intellectual disabilities. J Pediatr Psychol. 2008;33(10):1129–36.
7. Lach LM, Kohen DE, Garner RE, Brehaut JC, Miller AR, Klassen AF, Rosenbaum PL. The health and psychosocial functioning of caregivers of children with neurodevelopmental disorders. Disabil Rehabil. 2009;31(9):741–52.
8. Murphy NA, Christian B, Caplin DA, Young PC. The health of caregivers for children with disabilities: caregiver perspectives. Child Care Health Dev. 2007;33(2):180–7.
9. Oelofsen N, Richardson P. Sense of coherence and parenting stress in mothers and fathers of preschool children with developmental disability. J Intellect Dev Disabil. 2006;31(1):1–12.
10. Olsson MB, Hwang C. Depression in mothers and fathers of children with intellectual disability. J Intellect Disabil Res. 2001;45(6):535–43.
11. Singer GH, Floyd F. Meta-analysis of comparative studies of depression in mothers of children with and without developmental disabilities. Am J Ment Retard. 2006;111(3):155–69.
12. Tong HC, Haig AJ, Nelson VS, Yamakawa KS-J, Kandala G, Shin KY. Low back pain in adult female caregivers of children with physical disabilities. Arch Pediatr Adolesc Med. 2003;157(11):1128–33.
13. Lee M, Park C, Matthews AK, Hsieh K. Differences in physical health, and health behaviors between family caregivers of children with and without disabilities. Disabil Health J. 2017;10:565–70.
14. Arim RG, Miller AR, Kohen DE, Guèvremont A, Lach LM, Brehaut JC. Changes in the health of mothers of children with neurodevelopmental disabilities: an administrative data study. Res Dev Disabil. 2019;86:76–86.
15. Brehaut JC, Garner RE, Miller AR, Lach LM, Klassen AF, Rosenbaum PL, Kohen DE. Changes over time in the health of caregivers of children with health problems: growth-curve findings from a 10-year Canadian population-based study. Am J Public Health. 2011;101(12):2308–16.
16. Resch JA, Mireles G, Benz MR, Grenwelge C, Peterson R, Zhang D. Giving parents a voice: a qualitative study of the challenges experienced by parents of children with disabilities. Rehabil Psychol. 2010;55(2):139–50.
17. Council for Disabled Children. Expert Parent Programme. https://councilfordisabledchildren.org.uk/our-work/whole-child/practice/expert-parent-programme. Accessed 21 Jan 2019.
18. Singer GH, Ethridge BL, Aldana SI. Primary and secondary effects of parenting and stress management interventions for parents of children with developmental disabilities: a meta-analysis. Dev Disabil Res Rev. 2007;13(4):357–69.
19. Dykens EM, Fisher MH, Taylor JL, Lambert W, Miodrag N. Reducing distress in mothers of children with autism and other disabilities: a randomized trial. Pediatrics. 2014;134(2):e454–63.
20. Shilling V, Morris C, Thompson-Coon J, Ukoumunne O, Rogers M, Logan S. Peer support for parents of children with chronic disabling conditions: a systematic review of quantitative and qualitative studies. Dev Med Child Neurol. 2013;55(7):602–9.
21. Law E, Fisher E, Eccleston C, Palermo TM. Psychological interventions for parents of children and adolescents with chronic illness. Cochrane Database of Systematic Reviews 2019, Issue 3. Art. No.: CD009660. https://doi.org/10.1002/14651858.CD009660.pub4.
22. Cachia RL, Anderson A, Moore DW. Mindfulness, stress and well-being in parents of children with autism spectrum disorder: a systematic review. J Child Fam Stud. 2016;25(1):1–14.
23. Jetten J, Haslam C, Alexander SH. The social cure: identity, health and well-being: Psychology press; 2012.
24. Borek A, Abraham C, Greaves C, Gillison F, Tarrant M, Morgan-Trimmer S, McCabe R, Smith J: Identifying change processes in group-based health behaviour-change interventions: Development of the Mechanisms of Action in Group-based Interventions (MAGI) framework. Health Psychol Rev. 2019: 1–21. https://doi.org/10.1080/17437199.2019.1625282.
25. Tarrant M, Khan SS, Farrow CV, Shah P, Daly M, Kos K. Patient experiences of a bariatric group programme for managing obesity: a qualitative interview study. Br J Health Psychol. 2017;22(1):77–93.

26. Haslam C, Cruwys T, Haslam SA, Dingle G, Chang MX-L. Groups 4 Health: evidence that a social-identity intervention that builds and strengthens social group membership improves mental health. J Affect Disord. 2016;194:188–95.
27. Peninsula Childhood Disability Research Unit. Our Family Faculty. http://www.pencru.org/getinvolved/ourfamilyfaculty/. Accessed 29 Jan 2019.
28. Bartholomew LK, Parcel GS, Kok G, Gottlieb NH, Fernandez ME. Planning health promotion programs: an intervention mapping approach. 4th ed. San Francisco: Jossey-Bass; 2016.
29. Borek AJ, McDonald B, Fredlund M, Bjornstad G, Logan S, Morris C. Healthy Parent Carers programme: development and feasibility of a novel group-based health-promotion intervention. BMC Public Health. 2018;18(1):270.
30. Muehlhausen W, Doll H, Quadri N, Fordham B, O'Donohoe P, Dogar N, Wild DJ. Equivalence of electronic and paper administration of patient-reported outcome measures: a systematic review and meta-analysis of studies conducted between 2007 and 2013. Health Qual Life Outcomes. 2015;13(1):167.
31. Hammond P. Staying alive: how to get the best out of the NHS - advice from a doctor. London: Quercus; 2015.
32. Aked J, Marks N, Cordon C, Thompson S. Five ways to wellbeing: a report presented to the foresight project on communicating the evidence base for improving people's well-being. London: Nef; 2009.
33. Williams U, Rosenbaum P, Gorter JW, McCauley D, Gulko R. Psychometric properties and parental reported utility of the 19-item 'About My Child' (AMC-19) measure. BMC Pediatr. 2018;18(1):174.
34. Department for Communities and Local Government. English indices of deprivation 2015. https://www.gov.uk/government/statistics/english-indices-of-deprivation-2015. Accessed 12 May 2017.
35. Tennant R, Hiller L, Fishwick R, Platt S, Joseph S, Weich S, Parkinson J, Secker J, Stewart-Brown S. The Warwick-Edinburgh mental well-being scale (WEMWBS): development and UK validation. Health Qual Life Outcomes. 2007;5(1):63.
36. National Institute for Health and Care Excellence. The social care guidance manual. London: NICE; 2016.
37. Kroenke K, Spitzer RL. The PHQ-9: a new depression diagnostic and severity measure. Psychiatr Ann. 2002;32(9):509–15.
38. Kroenke K, Spitzer RL, Williams JB, Löwe B. The patient health questionnaire somatic, anxiety, and depressive symptom scales: a systematic review. Gen Hosp Psychiatry. 2010;32(4):345–59.
39. Wittkampf KA, Naeije L, Schene AH, Huyser J, van Weert HC. Diagnostic accuracy of the mood module of the Patient Health Questionnaire: a systematic review. Gen Hosp Psychiatry. 2007;29(5):388–95.
40. National Collaborating Centre for Mental Health, Royal College of Psychiatrists. Common mental health disorders: identification and pathways to care: RCPsych Publications; 2011.
41. National Institute for Health and Care Excellence. Depression in adults: recognition and management. https://www.nice.org.uk/guidance/cg90. Accessed 21 Jan 2019.
42. The EuroQol Group. EuroQol-a new facility for the measurement of health-related quality of life. Health Policy. 1990;16(3):199–208.
43. Kiplinger VL, Browne CH. Parents' assessment of protective factors: user's guide and technical report. Washington: Center for the Study of Social Policy; 2014.
44. Bourke-Taylor H, Law M, Howie L, Pallant JF. Initial development of the Health Promoting Activities Scale to measure the leisure participation of mothers of children with disabilities. Am J Occup Ther. 2012;66(1):e1–e10.
45. Muskett R, Bourke-Taylor H, Hewitt A. Intrarater reliability and other psychometrics of the Health Promoting Activities Scale (HPAS). Am J Occup Ther. 2017;71(4):7104190010p7104190011–8.
46. Hibbard JH, Mahoney ER, Stockard J, Tusler M. Development and testing of a short form of the patient activation measure. Health Serv Res. 2005; 40(6p1):1918–30.
47. Hibbard JH, Stockard J, Mahoney ER, Tusler M. Development of the Patient Activation Measure (PAM): conceptualizing and measuring activation in patients and consumers. Health Serv Res. 2004;39(4p1):1005–26.
48. Al-Janabi H, Flynn TN, Coast J. Development of a self-report measure of capability wellbeing for adults: the ICECAP-A. Qual Life Res. 2012;21(1):167–76.
49. Al-Janabi H, Peters TJ, Brazier J, Bryan S, Flynn TN, Clemens S, Moody A, Coast J. An investigation of the construct validity of the ICECAP-A capability measure. Qual Life Res. 2013;22(7):1831–40.
50. Mitchell PM, Al-Janabi H, Byford S, Kuyken W, Richardson J, Iezzi A, Coast J. Assessing the validity of the ICECAP-A capability measure for adults with depression. BMC Psychiatry. 2017;17(1):46.
51. Ridyard CH, Hughes DA, Team D. Development of a database of instruments for resource-use measurement: purpose, feasibility, and design. Value Health. 2012;15(5):650–5.
52. Moore GF, Audrey S, Barker M, Bond L, Bonell C, Hardeman W, Moore L, O'Cathain A, Tinati T, Wight D. Process evaluation of complex interventions: Medical Research Council guidance. BMJ. 2015;350:h1258.
53. Ridyard CH, Hughes DA. Taxonomy for methods of resource use measurement. Health Econ. 2015;24(3):372–8.
54. Eldridge SM, Chan CL, Campbell MJ, Bond CM, Hopewell S, Thabane L, Lancaster GA. CONSORT 2010 statement: extension to randomised pilot and feasibility trials. Pilot Feasibility Stud. 2016;2(1):64.
55. Braun V, Clarke V. Using thematic analysis in psychology. Qual Res Psychol. 2006;3(2):77–101.
56. International Committee of Medical Journal Editors. Defining the role of authors and contributors. http://www.icmje.org/recommendations/browse/roles-and-responsibilities/defining-the-role-of-authors-and-contributors.html. Accessed 18 Mar 2019.
57. NHS England: NHS England's commitment to carers; 2014.

Publisher's Note

Springer Nature remains neutral with regard to jurisdictional claims in published maps and institutional affiliations.

Ready to submit your research? Choose BMC and benefit from:

- fast, convenient online submission
- thorough peer review by experienced researchers in your field
- rapid publication on acceptance
- support for research data, including large and complex data types
- gold Open Access which fosters wider collaboration and increased citations
- maximum visibility for your research: over 100M website views per year

At BMC, research is always in progress.

Learn more biomedcentral.com/submissions

BMC

For peer review only

Healthy
PARENT CARERS

Facilitator training feedback form

During this phase of the research we are still developing and finalising the facilitator training. Your feedback will help us to ensure that any future training delivered is as comprehensive and useful as possible.

Your feedback is anonymous and you will not be identified in any research reports. This information will only be viewed by the research team.

For peer review only

1. Please rate, on a scale of 1-5, your knowledge and understanding, and skills and confidence in the following areas (with 1 being very little and 5 being a lot).

**Knowledge and understanding of:**	**1**	**2**	**3**	**4**	**5**
The intervention's theoretical framework (CLANGERS); the session activities and associated learning outcomes.					
Group facilitation techniques and how to create a positive group atmosphere.					
Particular challenges faced by parent carers and how this may impact on health and wellbeing.					
The role of the HPC Lead/Assistant Facilitator.					
The study design.					
Safeguarding procedures.					

**Skills and confidence**	**1**	**2**	**3**	**4**	**5**
Ability to confidently present programme information, lead activities and facilitate discussions.					
Ability to create a positive group atmosphere and inspire collective desire to make lifestyle changes to improve health and wellbeing.					
Ability to manage time effectively.					
Ability to manage difficult/sensitive issues that may arise.					

2. The time allotted for the training was sufficient** (**Please tick one)

Strong Agree	Agree	Neutral	Disagree	Strongly Disagree

3. Are there any aspects of the training that could be improved (e.g. content, time provided to cover the learning objectives and/or delivery)?

4. Any other comments?

Many thanks for taking the time to give your feedback.

For peer review only

Healthy PARENT CAREERS

CHECKLIST FOR MODULE 1 DELIVERY

Instructions to the Facilitators and Assistant Facilitators:
Please complete this checklist at the end of each session to provide us with feedback on what was covered and how the session went. This feedback will help us assess if the sessions can be delivered as planned and identify potential challenges and improvements.

Date & time of the session: ____

Session number: ________________________

Venue: ____

Facilitators' names: ____

Session duration (as delivered): _______________________

Number of participants attending: _____________________

**Activities**	**Delivered** (Please tick if delivered)	**Comment** (If partly or not delivered, please explain why)
1. Introductions / icebreaker activity		
2. Hopes and fears		
3. Ground rules		
4. What are health and wellbeing?		
5. What influences health and wellbeing?		
6. The resilience game		
7. Conclusion		
Time for informal interaction (e.g. tea break)		

For peer review only - http://bmjopen.bmj.com/site/about/guidelines.xhtml

Reflections on the session

Please indicate which picture best describes your perception of how well connected, in general, the attendees of today's session appeared to be (tick the box on the right of the picture).

For example, the first picture would suggest that there was very little connection between individuals – that they did not come together as a group at all. Whereas the final picture would suggest that individuals were very closely connected – they very much came together as a group.

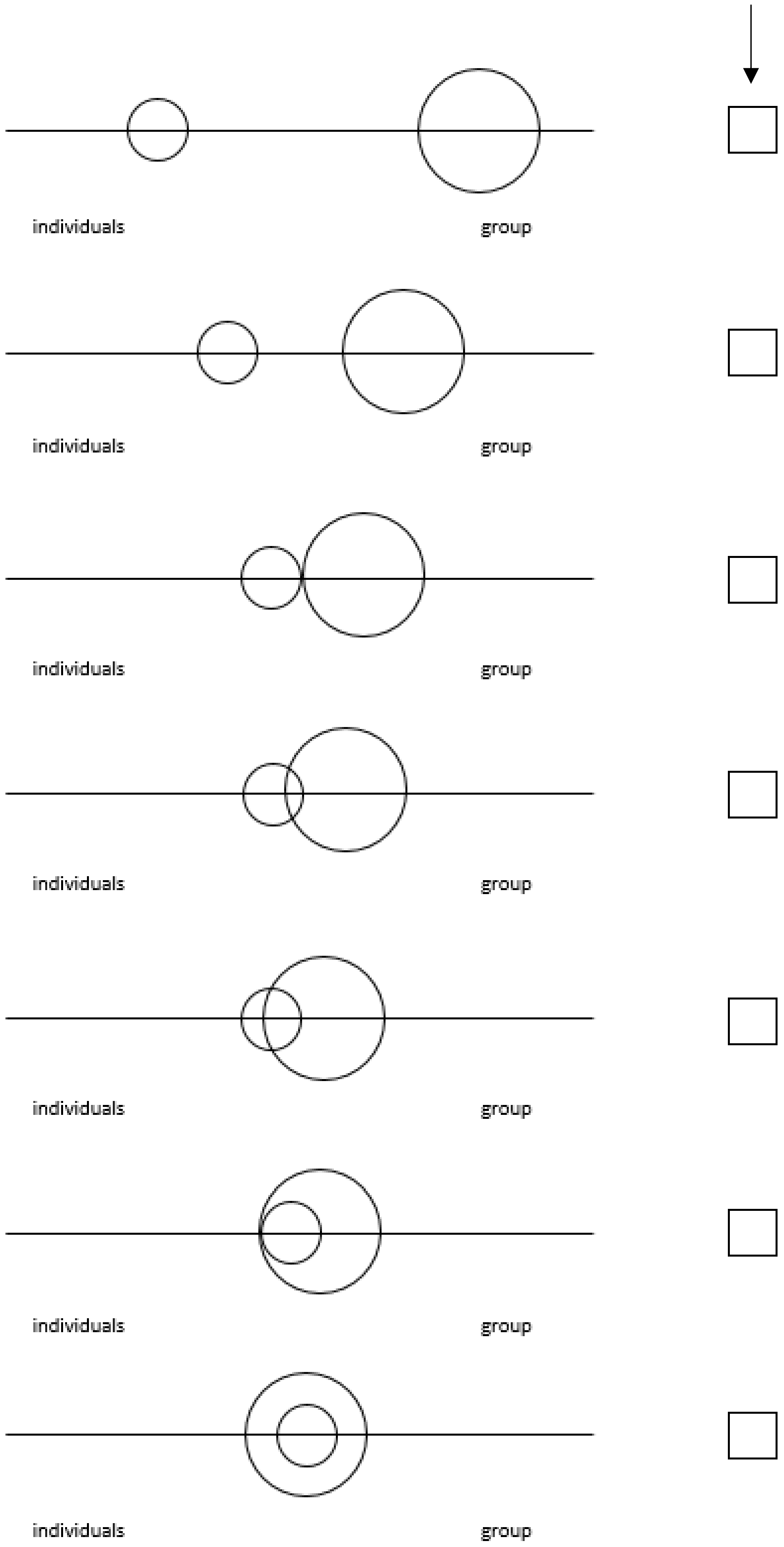

Reflections on the session cont.

Please circle one

In your opinion, how well did today's session go?

Not well at all				Very well
1	2	3	4	5

How engaged were the participants in today's session?

Not well at all				Very well
1	2	3	4	5

Do you have any comments or reflections?

(For example, related to how the group worked together, participants' engagement, session content or activities)

For peer review only

For peer review only - http://bmjopen.bmj.com/site/about/guidelines.xhtml

Healthy
PARENT CARERS

Draft topic guide for a focus group with HPC Facilitators

Topics and example questions for the focus group with HPC Facilitators and Assistant Facilitators:

- Motivation for / expectations of delivering the group programme, e.g.:
  - Why did you decide to get involved in this study?
  - What did you expect from taking part in the study / delivering the programme?
- Views about training / preparation to deliver the programme, e.g.:
  - How did you find the training to deliver the programme? (Prompt about whether it was sufficient, anything that would be helpful)
  - How prepared did you feel to deliver the programme? (Prompt about their background and previous experience, or any new skills developed by delivering HPC)
- Experience of delivering the programme / group facilitation, e.g.:
  - Having delivered the programme now, what do you think about it?
  - Generally, how did you find delivering the programme? (Prompt about following the Facilitator Manual, any adaptations made, challenges, how feasible it was to deliver)
  - How engaged was the group / the participants?
  - How did you find facilitating / managing the group? (Prompt about any challenges and how they managed those, how engaged they were in delivering it)
- What worked best in the programme? Why? (Prompt about activities, content, group)
- What didn't work well? What were the challenges or difficulties? Why?
- Do you have any suggestions for improvements? (e.g. related to the programme delivery, content, Facilitator Manual, training)
- Is there anything else that we haven't talked about and that you'd like to discuss?

For peer review only - http://bmjopen.bmj.com/site/about/guidelines.xhtml

Healthy
PARENT CARERS

HEALTHY PARENT CARERS
WEEKLY PARTICIPANT FEEDBACK FORM FOR GROUP INTERVENTION

GROUP: ____

SESSION NUMBER: ____

We would like to know what you think about this session and would be grateful for your feedback. The feedback is anonymous and it will help us refine and improve the programme in future.

Please circle one

1. Overall, how satisfied are you with today's session? (please circle one)

Not satisfied at all				Very satisfied
1	2	3	4	5

2. Do you feel that you were able to take an active part in the session? (please circle one)

Not at all				Very much
1	2	3	4	5

3. Please tell us about the best things in today's session (e.g. most enjoyed or most helpful):

4. Please tell us about the things that could be improved in today's session:

For peer review only

5. Which picture best describes how well connected, in general, you felt to the rest of the group today?

For example, the first picture would suggest that you felt very little connection to the rest of the group, whereas the final picture would suggest that you felt very connected to the group.

Please tick the box on the right hand side of the picture that best describes how you felt.

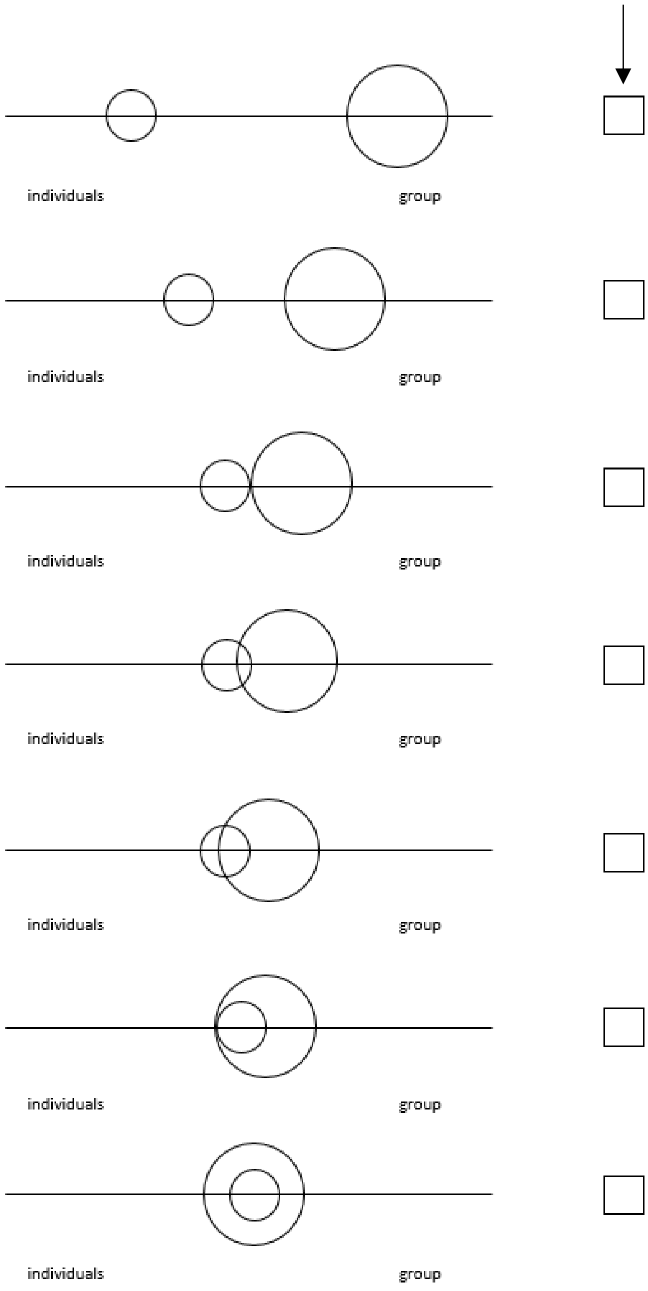

6. How much did you identify with other members of this group? (please circle one)

Not at all				Very much
1	2	3	4	5

HEALTHY PARENT CARERS
Feedback FORM

We would like to know what you think about the Healthy Parent Carers programme and the research study and would be grateful for your feedback. The feedback is anonymous and it will help us refine and improve the programme and our research in future.

1. Overall, how satisfied are you with taking part in the Healthy Parent Carers programme?

Not satisfied at all				Very satisfied
1	2	3	4	5

2. How useful has the programme been in helping you improve your health and wellbeing?

Not at all				Very much
1	2	3	4	5

Was there anything in particular that helped you?

3. Have you made any changes as a result of taking part in the programme? No / Yes

If yes, what?

If no, why?

4. Approximately how many of the online materials have you accessed?

None	25%	50%	More than 50%

5. Did you attend group sessions for the programme? Yes / No

If yes, reveal questions 6-10, then continue from question 12.

If no, reveal question 11, then continue from question 12.

6. How would you rate the length of the individual sessions?

Too short	About right	Too short

For peer review only

7. Did you miss any of the group sessions? Yes / No

If yes, why?

8. Overall, how much did you feel included and part of the group?

Not at all				Very much
1	2	3	4	5

9. How satisfied were you with the way the sessions were facilitated and delivered?

Not satisfied at all				Very satisfied
1	2	3	4	5

10. Did you discuss the programme with anyone who had access to the online materials only (i.e. they did not attend the group sessions)? Yes / No

11. Did you discuss the programme with anyone who attended the group sessions? Yes / No

12. Do you have any suggestions about how the programme or materials could be improved?

13. Would you recommend this programme to other parent carers?

Not at all				Very much
1	2	3	4	5

14. Do you have any other comments, reflections or suggestions?

For peer review only

Appendix 2: Data collection materials

Follow-up questionnaire
Version 1, 11 June 2019

HEALTHY PARENT CARERS
Follow-up questionnaire

1. Have you continued to access the online resources in the past 6 months?

Yes / No

2. Approximately how many of the online materials have you accessed in total?

None	25%	50%	More than 50%

3. Did you attend group sessions for the programme? Yes / No

If yes, reveal question 4.

If no, end survey.

4. Since the Healthy Parent Carers group ended, how many members of your group have you been in touch with (including members that you knew prior to starting)?

(number up to 10)

5. Out of these, how many were people that you did not know before the programme?

(number up to 10)

6. Since the group ended, have you been in touch with any members the group that you didn't know before in any of the following ways?

(select all that apply)

Phone

Text

Email

WhatsApp

Other Social media (e.g. Facebook, Twitter)

Meeting in person

Other (please specify – please do not include anyone's name) (free text box needed if ticked)

For peer review only - http://bmjopen.bmj.com/site/about/guidelines.xhtml

Appendix 2: Data collection materials

Follow-up questionnaire
Version 1, 11 June 2019

I have not been in touch with any of the group members that I didn't know before

7. Approximately how long ago was the last time you had contact with any members of the group that you didn't know before it started (please do not include anyone's name)?

(free text)

8. Can you please describe how often you have been in touch with any members of the group that you didn't know before (please do not include anyone's name)?

(free text)

9. How many of these new contacts would you now say are your friends?

(number up to 10)

For peer review only

For peer review only - http://bmjopen.bmj.com/site/about/guidelines.xhtml

Healthy
PARENT CARERS

Draft interview topic guide: HPC Participants (intervention arm)

- Introduce myself (PenCRU childhood disability research unit)
- The aim of the interview is to get their feedback on the HPC programme and research study
- Confirm how much time interviewee has for interview. Confirm that meant to take about 30 mins but can vary depending on length of discussion – let me know if you have somewhere you need to be and we can manage accordingly
- Remind participant that taking part is voluntary – if I ask about something you don't want to talk about, please just say you'd rather not go in to that
- Remind participants that the interview will be recorded and transcribed and analysed to help us understand how the programme and study worked. Interview transcripts will be anonymous.
- Check participant is comfortable (has drink of water etc.) and has given consent to continue

Record before/after the interview:

- Participant ID:
- Participant's group ID & facilitators' IDs:
- Gender:
- Age:

Topics and example questions for participants in the intervention arm:

- Motivation to participate & expectations, e.g.:
    - Why did you decide to take part in the study / programme?
    - Was there anything that helped you to decide to take part?
    - What did you expect from taking part in the study?
- Understanding of, and views on, the study design, e.g.:
    - How sufficient did you find the information about the study & the programme and what they involve?
    - What did you think when you found out that you were assigned to the group programme, rather than only being given access to online materials?
    - Would you change any aspect of how it was explained to you, or what information was available, about the programme or the research? (probe suggestions from them)
- Experience of the group programme (activities, group):
    - What do you think about the content of the programme? (prompt about relevance "relevant to you as a parent carer", helpfulness of the content e.g. CLANGERS)
- Views on length & frequency of sessions; any missed sessions & reasons for missing sessions
    - What do you think about the length and frequency of the sessions? Did you need to miss any sessions? (prompt about reasons for missing sessions)
- Views on & engagement with the group:
    - Tell me about your group. Did you feel included and a part of it?
- Views about facilitators and delivery style, e.g.:

For peer review only

- What do you think about the facilitators and the way they delivered the programme? How well did they do in delivering the programme and facilitating the group? (prompt about facilitator engagement)
- Usage of, and views on, the online programme materials
    - Did you access the programme materials online? (prompt about how they used them/how often, or about reasons for not using them) If yes, what prompted you to look at them?
- Impact of the programme & any changes made, e.g.:
    - How helpful did you find the programme? Did you make any changes in result of the programme? (prompt for examples) Was there anything that got in the way of you being able to make those changes? Was there anything that supported you to make those changes?
- Contextual influences, e.g.:
    - Did anything affect how much you got out of participating in the programme? Were there any barriers to you engaging with or benefiting from the programme?
- Best & worst things, suggestions for improvements, e.g.:
    - What did you like most / found most helpful (if anything) about the programme?
    - What did you not like / did not find helpful (if anything)?
    - Do you have any suggestions for how the programme could be improved? (prompts: delivery, content, group, timing / length of programme)
- Acceptability of data collection and measures, e.g.:
    - How did you find having to fill in all the questionnaires at the beginning and at the end of the study? (prompt about time, clarity, relevance)
    - The sessions were audio recorded. How would you have felt if the sessions had been video recorded instead?
- Is there anything else that we haven't talked about and that you'd like to mention?

For peer review only

Healthy PARENT CARERS

Draft interview topic guide: HPC Participants (control arm)

- Introduce myself (PenCRU childhood disability research unit)
- The aim of the interview is to get their feedback on the HPC programme and research study
- Confirm how much time interviewee has for interview. Confirm that meant to take about 30 mins but can vary depending on length of discussion – let me know if you have somewhere you need to be and we can manage accordingly
- Remind participant that taking part is voluntary – if I ask about something you don't want to talk about, please just say you'd rather not go in to that
- Remind participants that the interview will be recorded and transcribed and analysed to help us understand how the programme and study worked. Interview transcripts will be anonymous.
- Check participant is comfortable (has drink of water etc.) and has given consent to continue

Record before/after the interview:

- Participant ID:
- Gender:
- Age:

Topics and example questions for participants in the control arm:

- Motivation / expectations to participate, e.g.:
  - Why did you decide to take part in the study / programme?
  - Was there anything that helped you to decide to take part?
  - What did you expect from taking part in the study?
- Understanding of, and views on, the randomised controlled design and control group, e.g.:
  - How sufficient did you find the information about the study and the programme, and what they involve?
  - What did you think when you found out that you were assigned to the group with access to online materials only and not the group programme?
  - Would you change any aspect of how it was explained to you, or what information was available, about the programme or the research?
- Contamination with the intervention arm, e.g.:
  - Do you know anyone who also took part in the study and attended a group? (If yes, prompt about how much they discussed what happened in the group and how much that might have influenced their view on the programme materials or changes made).
- Usage of, and views on, the online programme materials, e.g.:
  - Did you access the programme materials online? (prompt about how they used them/how often, or about reasons for not using them)
  - What do you think about them?
- Impact of the programme, any changes made, e.g.:

For peer review only

  - How helpful did you find the materials? Did you make any changes in result? (examples) Was there anything that got in the way of you being able to make those changes? Was there anything that supported you to make those changes?
- Views on the data collection and measures
  - How did you find having to fill in all the questionnaires at the beginning and at the end of the study? (prompt about time, clarity, relevance)
- Best & worst things, suggestions for improvements, e.g.:
  - What did you like most / found most helpful (if anything) about the programme?
  - What did you not like / did not find helpful (if anything)?
  - Do you have any suggestions for how the materials or the research elements could be improved?

Is there anything else that we haven't talked about and that you'd like to mention?

For peer review only

Supplementary Document 11. Additional quotes and comments

1. Facilitators' experiences

1.1. Motivations and expectations

New experiences, new skills & being involved in the research project. (Facilitator pre-training questionnaire)

Recognised the need for healthy wellbeing of parent carers through myself and friends. Facilitate a small support group locally. Ready for a new challenge. (Facilitator pre-training questionnaire)

To apply my training delivery skills and experience to a programme which is unique in its focus on parent carer health, something myself as a parent carer feel I could directly benefit from. (Facilitator pre-training questionnaire)

CLANGERS has changed my life and I feel it is really important to help others by spreading the message. It's also a research project and that excites me as well! (Facilitator pre-training questionnaire)

I always believed in this course because it had such a positive impact on me and I just wanted to give back to others. (AF, focus group)

When we did the [training], I do remember thinking some of this is really resonating with me and my own challenges and thinking actually, I can see where this is going, and it has been really, really good. (LF, focus group)

1.2. Training, preparation and support

It became clear to me that there was actually quite a distinctive style that was expected as well… we would all have liked some more concrete direction in terms of what to do and how. (AF, focus group)

It's something actually a little bit more fundamental about that. It's about why are you writing this stuff down? Because there's nothing in the trainer notes that explained to the trainer what the purpose of having this stuff written down is. I always find it difficult delivering training that's been written by somebody else… I had to keep saying in my head, 'do it as it's written, don't go off and do your own thing'. (LF, focus group)

I would [practise] 'Connect' because it would give you the opportunity to identify some of the issues that are going to come up, because 'connect' can be really difficult for parent carers. So you could use it as, well this is an example of somebody who struggles, how would you do it in this situation, how would you cope with this? So it can be quite an emotional one and it's also the first one that we do in the programme… The group were also very nervous and quiet in that one, so that makes it harder. (AF, focus group)

LF: …trainers probably need to undergo the training in the first instance because I think you need to experience the programme, both to have a better understanding but you would get a lot of ideas and facilitation through that.

For peer review only

AF: I felt I was at an advantage because I had done it… because I knew the course I was able to listen and scribe, knew what was coming up because I'd done it before, but I was also able to input in my way and from my experiences… which I felt helped. So it wasn't new information to me, I'd already gone through that quite hard process of thinking and I had already adjusted and adapted and was aware of it and everything else. And you could see it on the faces of the participants that actually it was hard at times and I had been there, so I understood. (Focus group)

More time for assistants to get a clearer understanding of breath of exercises / module content. (Assistant training, feedback form)

It's all very hurried. More time practicing our role. (Refresher training, feedback form)

1.3. Programme content and group delivery

It's tricky to manage but more difficult with some groups than others, definitely… but I think for the most part parents actually said it was really nice to focus on themselves. (LF, focus group)

LF: I would be very tempted to remove SMART from there and just replace it with achievable.
AF: The most important thing about CLANGERS is that you take ownership of it and you become aware that that's what you're doing. So it's important to have those goals but it's also important to recognise when you're not achieving those goals, where did it fall down. (…) so actually you might not have achieved that goal but you've actually done bits of CLANGER here instead. So it's just raising that awareness and your own understanding and building up that way. (Focus group)

The fact that we had such small groups was really disappointing for me… I think it changed the nature of the course, being such small groups, it was far more intense, I believe, for the training team, than it would have been if we'd had lots of participants there who were able to share their views and other things. So yes, altogether a highly enjoyable and positive experience but a few things that, perhaps, my expectations were slightly different. (LF, focus group)

I think the key of the leaders being parents of children with disabilities just really, really helps everybody to bond and understand. For me, I'm like wow, you've been through it, you're this much further on the same journey that I'm on and look at you; you're out here and you're doing this and you're working and, oh, your life is okay… That's reassuring. (AF, focus group)

All participants were very engaged. Unfortunately, one has a lot of problems currently so found it difficult to discuss resilience without being extremely emotional. Other participants very supportive. (Facilitators session checklist)

Group extremely connected. Tearful at the end of evening. Have made arrangements for continued contact as a group. Great sharing + reflection of course. (Facilitator checklist at the end of the final session)

2. Participants' experiences and views

2.1. Motivation to participate and expectations

For peer review only

Wanting to improve health and wellbeing:

I feel that being a parent-carer is really draining on your mental health and also I wanted to try and get healthy anyway, especially with it being January. Yes, I just thought it would be a good thing to try out and see if it does help me. (…) a bit of knowledge maybe about the way I feel and maybe a bit less guilt, the fact that I feel that I need to take time out for myself. (P13, control)

I was aware that my own health, fitness, mental health. All of those things really have taken quite a hit over the years, so it was a bit of a chance to actually focus on that for probably the first time in a long time really. So that was my main motivation. (…) It just seemed to fall into my lap at the right moment, so I, yes, sort of grabbed the opportunity with both hands really. (P3, intervention)

We decided to take part in the study because we wanted to be proactive as we are on most things. We knew that we needed to help ourselves as carers because we've become carers abruptly and inadvertently. (…) We've been using all the materials together [with his wife]. And we wanted to take part because we aren't going to sit on our arses and wait for something to happen because what we've learnt is the public sector will do nothing for you unless you poke it very hard. (P14, control)

I just thought it could be useful because a lot of the time I find as a parent carer all the focus is on the children which is obviously the main thing but the parents need to be healthy and happy to give the children the best. (P8, intervention)

Expecting advice, resources and signposting:

I was hoping just to find some resources to help me turn the attention off my daughter and more onto myself, just learn how to look after myself in amongst the very challenging times that we have looking after my daughter. (P15, control)

It was sort of different to what I expected because, like I say, I was expecting more sort of direct, "Right, this is who you can go to," rather than sort of what seemed like giving people information that they probably already knew. (P7, intervention)

Good timing of the programme:

If you wanted me to do the course at the beginning of last year I wouldn't have been able to do it because my daughter was very ill, but now she's in a good place at the moment so I could do it. (…) I've got to that point in my life that I have to say right, [my daughter] will always come first, she has to, and my husband and the rest of the children and grandchildren. But sometimes the work you do has to change, it has to go in a different direction (…) I've just always got on with it, but I think now I've got to that point that I think right, things have to change. (P11, intervention)

I just thought that it just fitted with the times in my life that I wanted to try and get a bit healthier and a bit less stressed. (P13, control)

Probably the fact that it was on Monday night. I've got very few timeslots that are regularly available and that one happens to be free, so. I think that probably was the biggest thing that made me go, yes let's just do it. (P2, intervention)

For peer review only

Wanting to contribute to research:

I thought it would be really helpful for me and I just felt it is quite important research, because I can't say I've looked up if there is research in this area, but I don't think there is a lot of research and I don't think unless you are in the situation, no one really gets the impact on your life and your health and everything else, it just takes over. So it just felt important for me to do for myself and also part of a bigger picture really because I think they are many many people in this situation and I don't think there's much light shed on it. I think it's getting better. But it just felt like an important thing to be part of really. (P3, intervention)

Well I thought it would help me with day-to-day stuff, but it's also an area that I have looked for help with in the past and found that there isn't any specifically, mental and physical health specific to parent carers, so I wanted to contribute to it because I want it to work [laughs], I want it to be there for when people need it. (P6, intervention)

Preference for group programme:

I was just excited to be involved in something and potentially going to be around other people… (…) I was hoping to meet other people who are in a similar situation to me and I think that you can learn a lot that way and also it's a support, meeting other people. Particularly for me, I'm very isolated and I'm assuming that there are other parent carers that are the same, that you can become very isolated and also quite often friends and family don't really understand, they say they do but you know from their actions and the things that they say sometimes that they don't really understand. So you can feel quite lonely. So having the opportunity to know that I'm going to be with other people who are in a similar situation, I was really pleased about. (P4, intervention)

I just thought that was going to be a lot more likely to be, well, something I would see through because there's a kind of commitment to turning up each week and I'm dreadful for just dropping out of things and going, oh whatever, I'm too busy… (P2, intervention)

I was a bit gutted really because I know that I always struggle with things on computers. It's not because I struggle with computers, but I always put it off to the next day and the next day, whereas if it was a group, I would just go to it as if it was an appointment. (P13, control)

I would have liked to have been part of a group to be able to discuss it with as a parent going to do similar things. (…) If there was to be a Healthy Parent workshop here in [town], I would have done it, I would have been able to find a way and I think I probably would have got more out of it from just sharing experiences and getting to know other people just for networking and support for each other. (P15, control)

Preference for, or acceptance of, online-only programme:

I wouldn't have been able to attend the groups anyway. I'm working 12-hour days, so it would have been a real rush. If you'd assigned me to the group, I probably would have asked to either drop out or go online anyway. So it worked out well in the end. (P14, control)

I was disappointed [when found out about being assigned to the group programme] because I was quite looking forward to doing the online material where I didn't have to go out, see people and talk to people, but again that is just my personal preference. It is perfect because it pushed me out of my comfort zone. I was quite apprehensive on the first day. (P9, intervention)

Feeling nervous before the first session:

It took a lot of self-will to take myself to the first session. It was one of those where when it came to it I was really excited and getting ready for it, then when it actually came to it I suddenly was trying to find excuses. (…) It was the sheer fact that just as it was about to start, everything around me became 10 times busier. (…) I think that was my main excuse for why I was allowing apprehension to take over. I think it is always that self-doubt factor of, is it going to be any use to me? Am I going to have to talk to loads of people and I don't really want to? It was a combination of those factors I think. (P9, intervention)

2.2. Experiences of the programme and its impact (and mechanisms)

Positive experiences:

It was fantastic. I think it was really well done. (…) I think it's changed my life. I really do. I haven't stopped talking about it since. Yes. It just makes complete sense. (…) I think the best thing was going to the group, meeting everybody in the group, just meeting like-minded people, having a break from life and being in a different place for a while, reflecting on aspects of my life that I didn't really think about or had put away, and I learnt more about myself. (P5, intervention)

I thought it was brilliant. It really has opened my eyes on an awful lot of things and I have changed an awful lot in my life. (P10, intervention)

The impact of a course like that, if that keeps me going so that I can look after my daughter, if it helps me figure out, work out our stresses in our family, keeps my relationship together, that has not just impact for our family obviously but wider in terms of taxpayers' money. So for me, I think it's really important. (P4, intervention)

I suppose they made you feel uncomfortable, which once you have got over the feeling uncomfortable about the fact that you have analysed something and thought oh I don't really like that and you can kind of box it but you think well actually no I need to address that. But that's just more about taking you out of your comfort zone really. But that's not really a bad thing, that's just an uncomfortable thing. (P1, intervention)

Lots of things! Meeting people who understand and 'get it ' reduced my isolation. Having parent carers as facilitators helped a lot. Having 12 structured sessions made me commit to it and focus on my own health. Having structured fun informative sessions helped a lot - I definitely went through a process of change. The use of humour was really important to me - and learning to be more resilient. (End-of-programme feedback)

Learning how useful and how important it is to keep myself healthy, not only for myself but for my child family and friends. Learning how to reflect and realising the amount I already do for myself and others that I am now proud of achieving a good sense of self-worth. Using Smart goals. (End-of-programme feedback)

I just think it was excellent and totally life changing!! Every parent carer in the UK and worldwide should have access to this course!! Thank you so so so much!! (End-of-programme feedback)

For peer review only

The group:

Being part of a group feeling like my challenges were understood and acknowledged. We laughed a lot cried sometimes and developed a lovely supportive friendship. I felt that the course was a really good balance of talking to off-load whilst also being 'solution focussed'. This was very empowering. Whilst my situation remains very challenging, I feel I have the skills and knowledge to help myself whilst also now having a wonderful new group of supportive friends. I am so grateful for this experience. Thank you. (End-of-programme feedback)

Our group worked really well actually and it really evolved, which was lovely because there were people who were thinking they weren't going to make it through to the end and they did. (…) We had quite an empathetic group and there was sort of, now that I'm thinking about it, possibly an unwritten code. (…) There was a lot of respect in the group. Obviously, there's some people whose kids have mental health issues and having major issues. There was a lot of well let's just sit and make sure we listen. There was a lot of support and everybody injected into the study, nobody sort of sat back, or if they were uncomfortable they said they were. As a group, it did, it evolved and it moulded and it's a really nice group. (P1, intervention)

I found it all helpful. I think the… and again, because it was a group, and that's why I would say I'm really glad that I was on the group side of it, the interaction and people's knowledge and people's just experiences and you kind of… but that sort of camaraderie type of feeling really, that's probably what I got more… probably, and then, yes, you put that down and the connecting that we did really. There's lots and lots of connecting really and… Yes, there was lots and lots of all of it actually, thinking about it, but just people putting through ideas of, "Well I do this, I don't know if that would work for you". There was a lot of that and it was, yes, that's probably what I got mostly from it. Yes. (P1, intervention)

It was a really nice group of people, everyone was very… I'd say everyone was very open and honest, people were able to talk about quite difficult circumstances and situations and it felt like a supportive environment and people were able to get upset and feel supported I think and also we did have a really good laugh, which was brilliant. I think we were really lucky. We are planning to keep in touch. Typical carers, no one can find the time when we are all available. It just sums it up really, doesn't it? But it was such a positive experience. After the first night I came out on a bit of a high because it's just always so… because friends and family and they care, but people just don't get it in the same way as when you are with a group of people who just know how bleak it can be, yes. (P3, intervention)

What I loved about the course was that it was very solution focused and I think quite often you can go to groups and things that it's just about talking, and as helpful as that is, you still come away with it going, well I'm not really sure… It was nice, but I don't really know how to make things better still, so to go to a group that's like you have the opportunity to talk and to gain help from other people and be listened to, great, but let's put that to one side, what are the barriers and how can we overcome them and let's work together to help each other overcome our barriers to having a more healthy and greater wellbeing in our lives. So I think if I'd have known that beforehand, I would have been even more excited. So maybe to have known that that was what it was trying to achieve. (P4, intervention)

For peer review only - http://bmjopen.bmj.com/site/about/guidelines.xhtml

I liked being around other people who were in a similar position to me. I liked the fact that we could listen to each other and offer support and help and useful advice. There was a lot of that that went on between us. Thinking creatively about how… from everybody's own experiences, what knowledge do I have that can help you? So I liked the supportiveness of the group. It wasn't all serious either, we laughed a lot, and that was lovely to have those moments where we shared silly jokes. It really was a case of we laughed together and we cried together, do you know what I mean, and we helped each other. So it was a really lovely group to be in and I really liked that. (P4, intervention)

We all got to share our stories, we all got to know each other, we all gave each other presents at the end and had a little party. It was really good. I think we all really became different people throughout the course. (…) I think the best thing was going to the group, meeting everybody in the group, just meeting like-minded people, having a break from life and being in a different place for a while, reflecting on aspects of my life that I didn't really think about or had put away, and I learnt more about myself. (P5, intervention)

When somebody was missing it kind of really felt like somebody was missing because we all had very different perspectives and different situations and it was really interesting. The biggest part of the thing for me was not what came out of the structure but out of the conversations that we had between us. Everybody interacted equally with everybody else across the six weeks, so there wasn't like two people who got on really well and had lots more in common and went off and talked between themselves, it was like completely among the group and it wasn't… In some weeks one of us might be quiet for our own reasons and that was fine. Everybody was very… I don't know, sort of accepting of other people's contribution, whether they were contributing a lot or not very much that week. (P6, intervention)

Everybody was listening and it was nice… near the end of the… the latter sessions, we were very much… if you understand what I mean, as a group, we were able to have little jokes together and bring up things that we discussed before. It was very nice. It was very much a camaraderie if you know what I mean. It was really nice and everybody was nice to everybody. There was not any anger or frustration. You could see people were struggling with certain things. We were able to advise them 'Actually, you are better than what you think' so it was really nice. (P12, intervention)

Group size:
It was smaller than I expected. I was expecting a group twice the size. And in some ways that might have been better because it might have been easier to find maybe subgroups within that that I could get… because there were definitely individuals in that group I could definitely get along with better but instead because it was very small it felt quite kind of intense which then meant that, yes, each person ends up trying to make it large and that… yes, and in a way that almost makes it harder to share. Yes, I don't know. I think I probably would have preferred a slightly larger group actually, but not massive. (P2, intervention)

There was only three of us, which was kind of a shame there wasn't more but, at the same time, we couldn't have got as much out of it as we did because we all got to share our stories, we all got to know each other, we all gave each other presents at the end and had a little party. (P5, intervention)

I think if the group was a little bit bigger, maybe not massively bigger but maybe, say, nine to ten people, I think you would get more input and there would be more discussion because even though

For peer review only

we were able to discuss things, I think a bit more of an open, broader amount of different people with different lives would make it a lot more interesting. (P12, intervention)

Peer facilitators:

We were sometimes a bit naughty on time when we talked too much, but I think that just happens when you are in that group. But yes, we managed to cover everything. There was ample time. They made sure everyone was included and had a say at every step really. I don't think there was anyone who didn't feel they could contribute or felt left out. (P3, intervention)

[Facilitators were] Brilliant, brilliant. And they were bouncing off each other. It was not just coming from one person. Because sometimes, you know when they do these things and you are only getting it from one perspective and [name] was able to challenge that perspective and then we were all able to challenge it and it was nice to be able to share rather than actually going, 'We are learning off a book that they were using to get their information' but actually, we were having to break it up even more on the sheets in front of us and where they were writing it all down, we were able to take notes and everything, so it was really good. (P12, intervention)

They really kept things going. They both had quite different approaches which I think complemented each other. Yes, it just seemed to work really well as a complete group including the facilitators really. (P3, intervention)

I think it makes a massive difference that they were both parent carers because they obviously have that innate understanding. I think it would have been different if they hadn't been, if the facilitators hadn't experienced some of those issues, I think it would have been very different. So I think that was a massive bonus. Because it's almost like a short hand, isn't it, there's things you don't even have to explain or go into because you just know that there's an understanding there already. So I think that was really helpful. (P3, intervention)

I think that's really helpful and important because it's not... Both of them are parent carers themselves and have been through very difficult situations and were open about sharing some of their challenges and their experiences and I think that that helps you then feel that it's okay to express what you are going through and that it's understood, because I think in those situations where it's being delivered by somebody who is reading the material without having had any of those challenges themselves, makes it less believable, less... like going to a dieting class that's being led by someone who has never had any problems, challenges with their weight, do you know what I mean? (...) It makes it less believable and somebody preaching to you but coming from a place that they have no experience of but they are preaching to you about how you can make changes in your life. And I think for me then I go I'm not sure if I can... It makes me not want to... It's less genuine, I guess. So for me having people facilitate the programme who have both had those challenges and, as I said, were quite open about their own challenges, and each week when we were going through the different clangers or whatever, they were honest at times, saying, "Well I've struggled this week to do it," and so it makes you feel it's okay that I'm struggling with this as well. And we all kind of worked together to help each other and encourage each other and that was a really lovely thing. Really lovely thing. (P4, intervention)

The facilitators of the programme were excellent in that they helped to create an environment where, as I said, it felt safe to talk about these things, that you were understood, that they cared about what you were going through and that very clearly came across from both of the facilitators, that they genuinely cared about what we were all going through and our challenges and also that they were honest about their own challenges. (P4, intervention)

For peer review only

The facilitators were very experienced (…) [they] were really knowledgeable, made us feel very relaxed, and I think that was a great part of it as well. (P11, intervention)

[Facilitator] was great, really good and whist she was delivering a programme she was absolutely excellent at tuning into individual people's situations and responses to things without singling them out. I could see she was being very empathetic and helping different people to get through different bits of it individually but as a group, if that makes sense, which I think was a real skill. She wasn't just delivering a programme, she was also doing it as individuals and again I think being a very small group really helped that. [Facilitator 2], yes, she was lovely. I mean, she was very good, kind. (P6, intervention)

They were lovely. Both of them were fantastic. I cried at times and they were caring, they were welcoming when you got there, they understood and remembered things. You felt like you got to know them and they got to know you and they'd talk about their experiences as well. I couldn't fault them at all, either of them, they were both fantastic. (P8, intervention)

This was doable because the facilitators weren't just 'professionals' preaching at us. (End-of-programme feedback)

The 2 tutors delivering the course were absolutely amazing! Being parent carers themselves really made them part of the group and not just 'teachers'. They were both friendly, welcoming, caring and informative. They made the course something to look forward to each week. (End-of-programme feedback)

Having the course delivered by another parent carer helped you to feel more at ease with disclosing information about your life and to someone who could totally relate and understand you circumstances. (End-of-programme feedback)

Content and activities:

The content of the course was good, it was really helpful and I know that it's going to help me going forward and I know that I am going to keep referring back to that to help me. (P4, intervention)

I think on every feedback form after every week it was like what was good about it and I was like discussion and sharing [laughs] which didn't come directly from the content of the programme but it made that happen. So that for me was the biggest thing because those are the things that stick, someone else's experience or if somebody else is maybe struggling with something, you'd support each other and you would kind of remember those things, you remember the personal stuff, human interaction that you have with people, whichever way, more than stuff you have been told. So I thought that was great. (…) It just seemed like a very natural way with that structure and the structure almost kind of… kind of like disappeared a bit into the background. I mean, sometimes to keep things moving, [facilitator] would have to bring us back to it and move on and stop one thing and start another thing, but it was almost like that became like a supporting thing, so the structure supported what happened in the session, it didn't drive what happened in the session, although it gave it structure but the session didn't hang on the content. (P6, intervention)

I think it would be relevant to everybody actually. I think everyone could probably do with doing that course. I think as parent carers we have additional challenges, obviously certainly lots of the stress and the anxiety associated with your children who are struggling, but I thought that the content

For peer review only

really helped you to focus on how do I keep myself healthy and look after my wellbeing, because if I fall down then I can't support my children. And I think that's really really important because I think so often families don't get support until it's too late. (P4, intervention)

I think it's relevant to all human beings [laughs], but I think putting it in the context of being a parent carer, yes I did find it very helpful and yes they were all the things. I don't think there was anything in there that was kind of superfluous and I don't think there was anything in there that was, I don't know. Yes, just when you blend them, when you blend the CLANGERS, they cover everything in life really. So parent carers, I didn't feel there was anything there, they were all things that were accessible. So there weren't things in there like for a lot of parent carers would feel like a non-starter, overwhelming, unachievable. They were all sort of day-to-day things that we all need to look at. Yes, I think they absolutely covered the typical things. Not to say that other people don't struggle with them but that parent carers particularly are more likely to struggle with more than the average person. (P6, intervention)

Learning about the CLANGERS and realistic ways to apply them - small steps. Having a laugh. (Weekly feedback, module 2)

Everything today I could relate to, enjoyed the videos, very informative and has made me look at how I can change to help improve my life. (Weekly feedback, module 3)

Looking at achievability of fitting in more exercise / active stuff. Looking for opportunities rather than barriers. (Weekly feedback, module 5)

Really enjoyed doing a 'top tips' exercise. It was a nice reminder of the CLANGERS. Revisiting the 'wheel' to see how far I have come. Very positive for me. (Weekly feedback, module 12)

Having 12 structured sessions made me commit to it and focus on my own health. Having structured, fun, informative, sessions helped a lot - I definitely went through a process of change. (End-of-the-programme feedback)

Loved the CLANGERS definitely made me think about the areas of my own wellbeing I need to work on. (End-of-the-programme feedback)

Reading the information made me think more about things I can do to help myself feel better. I even completed a cross stitch for the learning something new part. The CLANGERS acted as prompts to get me thinking about things I needed to do to give myself some time for myself. (End-of-the-programme feedback)

Being able to reflect on what's working and what isn't by going through the resources and questionnaire in a structured way. (End-of-the-programme feedback)

Being reminded and prompted:

I just am aware of… perhaps more aware of what's wrong, but I don't know that I've made, yes, any great changes to anything. I mean, I went away and said I'd do various things, and some of them I think were okay. I suppose it gives me more of a picture of what doing well might look like. (…) I'd have made them before if I could. I wasn't unaware… (P2, intervention)

It gave me a big kick up the bum to do something, I think that's been helpful. (P13, control)

For peer review only

A lot of it is very common sense stuff… you don't think about or focus on things enough. The CLANGERS, I've discussed that with quite a few people, because it's so simple, isn't it, but it really did help me to focus on yes just all of those things really. (…) It's like a revelation without it being a revelation, if you know what I mean, because you know all that stuff, but you don't take the time to think about it and it was just very much about focusing on us and improving things for ourselves before we can do it for everyone else really. (P3, intervention)

It was all about trying to make sure we reminded ourselves what we already knew but take the time to try and make sure we can sustain ourselves because sometimes you just get really tired, physically and emotionally. (P14, control)

It's not like a six-week programme where you go in not knowing something at the beginning and you come out knowing something at the end or being able to do something at the end that you didn't already know in your heart. It's a six-week programme that concentrates on reminding you of things that you can do and having had that reminder in a concentrated way it's with me all the time. (P6, intervention)

The CLANGERS acted as prompts to get me thinking about things I needed to do to give myself some time for myself. Being able to reflect on what's working and what isn't by going through the resources and questionnaire in a structured way. (End-of-programme feedback)

Reflecting and setting goals:
I think acceptance is a big thing, so accepting what you can't change. And we talked a lot about that on the course, looking at what are our challenges. (…) So it's kind of separating what you can and can't change I think helps you make steps forward, so going, okay, well this is the way it is, I can't do anything about that, so I'm just going to accept it, and now okay what can I change, what do I have control over and then breaking that down again into what are the specific barriers to me making those changes and working on those. (P4, intervention)

I think what causes the stress and anxiety and things like that for me and kind of the downward spiral that you can get into is that feeling that you don't have control, that everything is happening to you and you can't take control of it (…) So you can end up feeling like you don't have control over anything in your life anymore and that's when things start to spiral. So to stop for a minute and go, okay, well let's just think about my health and wellbeing and to break it down in a way that you can go, okay, this is what I know I need to do to be healthy and for my wellbeing for me to carry on doing this, but often everything can feel so overwhelming that you don't know where to start. So to be able to break it down like that and then go, right, I know I need to do this for my wellbeing, what are my barriers, what's stopping me from doing it, and then to analyse that and go, okay, well how can I realistically overcome that to make a positive change gives you… makes you feel like you have more control again. It brings back some control into being able to help yourself, which I think is really important, is having the tools and the skills to be able to help yourself, which is what I felt that course gave me. (P4, intervention)

It taught me how to break down those things into manageable chunks and to know that even a small change is good, that it doesn't… because I think so often we get caught up in the fact that it's all or nothing, right? You have to make that change and you have to do it all the time otherwise it's not worth it, and I think it's recognising that even small changes can make a big difference and being

For peer review only

able to work out how to make those changes, as I said, by breaking it down and looking at what my barriers are. (P4, intervention)

The message was you don't have to make massive changes to improve things. So I'm just trying to, for me and the kids really, tweak things a little bit to try and improve things a bit so it doesn't feel like an onerous task and I don't have to go to a class, I can sort of fit in activity as and when. That's quite reassuring really. So it's about making little changes that you can achieve rather than setting massive goals that you are just going to fail and then feel like a failure. (P3, intervention)

When you do the diary, ticking those and understanding what each planner meant it was suddenly occurring to me that I didn't eat very well, I didn't relax, I didn't sleep, so I needed to focus more on that side of my life. That really helped – the tick chart and setting goals. (…) What might stop you was literally time, so I know I needed to make adjustments. (P10, intervention)

Focusing on oneself:

You know all that stuff, but you don't take the time to think about it and it was just very much about focusing on us and improving things for ourselves before we can do it for everyone else really. (…) it has made me focus much more on my health and wellbeing and thinking I've just got to give myself that time. There's loads of things that I need to do, but at the minute I just need to get better. (P3, intervention

To be honest, it is all basic stuff, isn't it? It is just bringing it to the forefront, that actually, you are a worthy person and it is important that you do something for you, as well as your family and everybody else. (P18, control)

It is about making the carer feel that they are just as important as the caree. Because if we do not focus on ourselves, which is something we learnt, then you cannot work well for the person that you are caring for. (P12, intervention)

I think because of the way it was designed as well, we found out more about the people ourselves. We were ourselves in that. Even though we were carers and we were there because we were carers, we were talking about how we were. (P1, intervention)

Less positive experiences, limited impact:

The thing is, I found it helpful and I know I will continue to find it helpful. I can't say anything has changed as a direct result. Well, maybe some things have changed as a direct result of the programme but because I had an injury and because I'm just tired all the time, it's sort of like well that hasn't really changed [laughs] and it's not going to change. I think it gave me more hope that I will use… It's not like a six-week programme where you go in not knowing something at the beginning and you come out knowing something at the end or being able to do something at the end that you didn't already know in your heart. It's a six-week programme that concentrates on reminding you of things that you can do and having had that reminder in a concentrated way it's with me all the time. So as an example, I'm in a fairly poor state at the moment, not in life, nothing terrible but I'm really tired and struggling with this damn finger and feeling a bit like I'll never get things back to normal, but I know I will and I know what things I'm going to do to do that and the CLANGERS thing just… yes, it just stuck with me and I'm not speaking for other people but I could say that it did for the group, the other participants. It didn't make a massive short-term difference in my life. It did make a massive difference in my

For peer review only

thinking and my awareness and hope that [laughs]… I think it's hope. It gives you hope that you can get back on track no matter what, I'd say. (P6, intervention)

It was interesting, it showed me lots of aspects that I could look at. It wasn't just one thing to change, there were lots of things and one of the videos stuck in my mind. I keep thinking of that, but I haven't actually motivated myself to do anything about that just half an hour of your day. There's like 24 hours in the day and it's just like all we are asking for is half an hour to actually go and do some exercise. So that was the most beneficial that you could do to try and lift your wellbeing and your health and everything, I thought yeah, yeah. I'd got it in my head, but I still hadn't done anything which is a bit… I feel a bit annoyed with myself that I haven't. I thought that in my head all the time why don't I just do that half hour? (P16, control)

I think I probably would have got more if there had been more time for chatting because that was pretty much banned and the time was very limited because of the research element of it, whereas I think in a group like that people would chat normally, so probably the loo break was the best bit in terms of chatting, which sounds weird but it's a bit unnatural to ban chatting. (…) Some of the topics were much heavier than others and bigger than others and that wasn't reflected in the time allocation, so some of it felt like a bit of time filling and all the kind of introductory and end sessions felt a bit time filling when the actual topics really could have done with a lot more depth in some cases. (P2, intervention)

Right at the start was when we did the connecting. Well, it was a big one… I know CLANGERS starts with a C so you start with connect, but connecting is actually a really big one and actually can cause… I know we were talking about connecting with people outside, you are walking the dog, you chat with people you see and say hi, but you can actually come away feeling more lonely than you did when you went out and connected with them. (P1, intervention)

The one I disliked most of all was… It might have been relax. It was a lot to do with managing stress and it was all related to work type stress and very unrelated to the experiences that you have as a parent carer. Yes. Mmm. Once upon a time I had a job and that just pissed me off even more because actually I miss having a job enormously and it's just like [sighs] right, yes, great, yes, people get stressed at work, mmm, that would be nice [laughs]. Just a bit sort of… that one missed the mark quite badly for me but not necessarily for everybody else because it was hitting a sore spot really. (P2, intervention)

A lot of the videos were based on how to eat properly and how the brain works when you have exercise and I think that most people sort of understand these things and how beneficial they are but not everyone has access to them and not everyone can achieve what is put across in the videos and I was sort of more expecting help on… The thing is I think what you can't get away with being a parent carer is it's supposed to be for you as a parent carer but everything that you think about and you do, you think about your children, so you can't really get away from that aspect of it. So maybe if it was done in a way that it was for a parent carer and their children and what they can achieve then it would be different. (P7, intervention)

I must admit when I was watching the 'How to make a box' video, I was thinking, 'This is quite random.' For me, that was right up my street because I love making things like that, but I was thinking, 'If this was my husband watching it, he would probably be watching it thinking "Why am I watching this video?"' (P18, control)

For peer review only

2.3. Views on online materials

Mixed views:

I probably accessed about, well, 7 or 8 of them. Nearly all of them. I don't think I looked at the last one. But it just reinforced what we did in… I didn't do them… Of course, they only came up after, didn't they? I was going to say I didn't do them before because I didn't come up before, that's why. Yes, so I only accessed them after and then I think there was one occasion where the video didn't work or something… there was some reason why we couldn't see it and then I accessed it online. So it was great to have that as a backup if other things didn't quite work out in the session and to reinforce. (P1, intervention)

There's almost always something that I need to get done and if I've got access to a computer I'd rather be doing something more pleasant or relaxing or social than going through an online course on something that isn't central to my interests I guess. So yes, I think that goes back to why I was pleased that I got the real life group because it's like yes I actually will turn up and listen to that, whereas I'm not sure I will actually get round to doing an online course, I'd probably just go yes I just dropped out. (P2, intervention)

Initially I was curious and then I think it dwindled off because I felt like the course was covering everything so thoroughly that would it add anything to what I had already had? So I think that's probably why I didn't bother so much was because, yes, I felt that everything had been covered well during the session. (P4, intervention)

I missed most of [one] session. But then I went online and did it from online. Yes. It was really easy to get into and everything was on there, but I didn't take it in as well as I did on all the other days. It was the Connect and Learn were the two CLANGERS I wasn't there for. All of the other ones I think I got more out of than that one because even though I wrote stuff down from the internet onto the pages she gave me, it didn't sink in as well discussing it. (P5, intervention)

The week that I missed, I went through and used the online materials for that. So yes, a little bit, but I didn't do it systematically alongside the group each week… Once I'm out of the room, group, I've got a hundred million other things that take priority over that. (P6, intervention)

I thought they were really good, the little videos and things like that, I thought it was really helpful information and I think had I have been accessing it every week I would have found it helpful. I had a quick flick through the weeks to just have a quick nosey what it was about at the start. But yes, I do think it was helpful information, but I just didn't follow it through. (P13, control)

I really appreciated the reminders and I really appreciated the way it was laid out in saying what commitments are you going to make today to yourself, to anybody to do these things? But some of the online stuff was very good. (…) Some of the other stuff was just lowbrow and moved on, to be honest. It didn't keep my attention. Making a box, well, get real, come on. (…) So yes, there's some very good video content and yes it fitted together quite well, it's just that there was some demeaning stuff that I didn't do anything with, I just sort of saw it and thought that's not for me, moved it to one side and moved on. (P14, control)

What was there was absolutely fantastic and I think the videos were a good way of doing it because I, for one, am better at learning through watching the videos and being able to go back to them rather than just having loads and loads of information to read because obviously, you do get jaded and you do get tired, so… yeah, I thought it was done really well. (P18, control)

For peer review only - http://bmjopen.bmj.com/site/about/guidelines.xhtml

I found some of them were a bit patronising, a bit sort of childish [laughs] and very Americanised. There were like a few which I found more helpful or were quite pleasant to do, but some of the videos I thought were a bit, yeah, a little bit childish. (P17, control)

Lack of groups as impeding the learning:

I always put it off to the next day and the next day, whereas if it was a group I would just go to it as if it was an appointment, and I forget about things all the time otherwise. (P13, control)

I think although it was very useful to have it online, I did like that, but I wish there had been an option to maybe meet up once or twice in the group. I'm the sort of person that when I'm (recording inaudible 0:15:05.2) myself I do like getting out and about and meeting other people who have got similar things and I think it would have been… If you'd have had this group locally, I would have loved to have gone every week and I think that if I'd have gone every week I probably would have finished it no matter what and I think that personally I would have maybe got even more out of it by engaging with other people. (…) Maybe just gaining new friendships, to be able to have that contact with people after the group had finished. Yes, just sharing experiences, sharing tips. Lots of people have different ways of dealing with things and you get ideas from other people or you find out about things to support your children or your family or yourself that maybe you have not heard about. (…) I think it's really useful to be able to have that physical contact with other people really. (…) if you are in a group and you went back the next week and you said, "How did you get on?" it would just encourage you to discuss what you'd done that week and, as I said, maybe learn from other people and just taking it forward, being able to just be perhaps more motivated, I guess. If you are in a group and you see lots of people benefitting it just encourages you. (P15, control)

I was a bit disappointed because I'm in [town] and I thought oh I could do this every week, it will get me out of the house, because that would have given me the incentive to go somewhere and get it and it would have been a walk and some exercise to get there. I was thinking all that in my head, but actually even getting there would be part of it, part of the looking after myself. So I thought online it wasn't going to be as useful. (…) it took a discipline to sit down and actually make that time to look at the second, go back to it. (…) I did forget about it because you've got lots of things to do, whether you had a little reminder or something where you could check in or just say what you… you know what I mean? How did you find doing that part of it or maybe a bit of homework or something [laughs]. You could say, "Oh I didn't manage to do it," but it might be that incentive just to do something, whereas if no one is checking in on you just to say, "How did you find that?" or "Did you do anything?" then you are more likely to not and to put it lower down your list, aren't you? So if I had someone who I thought they are going to ask me what I've done, even like an email, they are going to ask me what I've done, I'll feel like I've got to do it, to try it, yes, but because there was nobody doing that, I didn't push myself. (P16, control)

I found doing the online course quite isolating. Personally, I would have… I think it would have made more changes in a quicker timespan if I had been in the group one. For me, it is not so much… like I said earlier, it is having that group session, it is being with people who are going through very similar things and I think if you have got the pressure, for want of a better word, of a group session, you are more likely to make those changes. Do you know where I am coming from? It is very difficult to put into words. But for me, I have done it online and I have ticked the box, if that makes sense. I have ticked the box. Yeah, I have done it, but it is… I think it is going to be more of a… it is going to be more difficult for me to be able to actually put those things into practice having done it online than if I was going to a course and they said, 'Right. Okay, what have you done for yourself this week?' (…) I think

For peer review only

that whilst it is brilliant to have had access to the course online, I do think it is something that possibly needs to be more personable, more face-to-face rather than a computer screen. Because that does isolate you in yourself. I am taking myself away from my husband and my family to go up and do it, which is great because it is time for me. (…) I do contradict myself, I know, because in one sense, it is easier to do it online because of the timings involved and things like that with work and all the balls that you are juggling, but on the other side, it is harder. It has been harder to do. (…) There was one video that was really quite long and I will not lie, I cannot even remember what it was about now, but it was probably the day I watched it, I had a really difficult emotional day and when I was watching this really long video, I think it was about eleven minutes, and I did find that I was very tired and I did find myself… my mind wandered because you are looking at a computer screen and you are thinking around you, 'I have got to do the washing. I have got to do this.' Because I was in my own environment, I found myself thinking about everything else that I should be doing, whereas if I was in… if I had gone to the group and they were watching that video, you have got nothing else to do, you are switched off. You are switched on to be watching that video, whereas for me, I was there thinking, 'Yeah. Okay, I have got to do this, so I will sit down and do this… and then my mind… like I say, I was… at one point, I was so tired that I was thinking, 'Oh gosh, I have got to go and make lunch, something for [name] and I to have for lunch tomorrow because I have got work, I have got issues at work.' (P18, control)

I found being in the online group really hard as well I could never find time to look at the resources. If I'd have been in the face to face group I'd have had allocated time to get out and access the sessions. (End-of-the-programme feedback)

I wasn't able to attend the groups so looked at the online resources and I don't tend to follow up on things from online. (End-of-the-programme feedback)

2.4. Factors influencing ability to engage with and benefit from the programme

Commitments and inadvertent events:

Just giving myself time to do it really. I didn't prioritise it highly enough; I was just putting other things before it. I knew it was all at the back of my mind and I put a note up for me to look at it, but because there was nobody telling me, I did prioritise other things really. (P16, control)

There were certain things that came up in my life that I hadn't planned, an interview one day and stuff, that it was kind of a big commitment for that sort of six weeks to be able to sort of… if something came up and I was like, oh no, really, and I didn't want to let people down obviously, but there was a few of us that had to take days off here, there and everywhere and maybe it was a bit too long. In hindsight, it probably would have been easier to have done the online stuff. (P7, intervention)

I've had this chest infection, which I missed the last session unfortunately because I was really quite ill and I didn't want to pass it on to other people who are looking after vulnerable people particularly. (P3, intervention)

I think the people who are kind of at the not coping end of the spectrum are likely to not make a lot of the sessions because you have to drop everything all the time. (P2, intervention)

For peer review only

I put it in a sheet, put it in Excel, added a few columns, put in words and scoring and, yes, I'm quite happy for you to see it. It just explains on some days you are impacted by inadvertent events and there is no relaxation, there is five hours' sleep. That's just the way it is. (P14, control)

At the end of a very long day when you are exhausted, you have been to work, you have had a meltdown from an unstable children, you are then getting them to bed, cooking them tea, bla bla bla and then you sit down, you can read something online, but it does not necessarily go in, so it was good to print some bits off, so that I have got that for future. (P18, control)

External factors:

When you have got too much in the way of other things that you are not getting what you need, you are not going to be able to look at it. So yes, I don't know that it felt massively relevant to me because I have got bigger fish to fry. (…) I have some really quite big social care needs and health care needs of my own that are not being met and so it is much more difficult to sit there going, "Ah well, have I eaten properly today or have I…?" That kind of stuff isn't the kind of… But those things don't exist so it's better than nothing. (…) But I think it's working really well for some people. Yes, I can see that other members of the group were getting masses more out of it than I was. So I think if it's the right thing for you then it's clearly way more beneficial. (P2, intervention)

To be honest, the timing of this course could not have been worse [laughter] because my colleague has just been diagnosed with lymphoma, so he has been taken away and he is undergoing really aggressive chemo at the moment, so that means we are a man short in the office and I am thinking, 'Oh my goodness, I have got all this to contend with at the minute,' but that is a parameter that you cannot… as a group, that is not your fault. That is just one of those things. That is not something that you can control. (…) Ironically, I do not know if you have got access to my before and my after, but I actually think my after ones are possibly the answers that are not quite so good in terms of my mental health than the beginning ones but like I say, that is circumstances out of your control because it is nothing to do with reflecting on the course; it is just purely that we have had so much negativity and bad things happening in the last six weeks, that that is possibly reflected in that, but like I say, there is not an awful lot you can do because that is outside of your control. (P18, control)

Unfortunately I have experienced many personal stressful situations since starting the programme which has not helped me to focus on it as much as I had hoped if things had been stable. (End-of-programme feedback)

If there is no respite available and you'll see very little difference as you can't implement what you learn - care is 24-7 in some cases. (End-of-programme feedback)

My son was poorly so I was only able to access so much. (End-of-programme feedback)

For peer review only

BMJ Open

The Healthy Parent Carers Programme: mixed-methods process evaluation and refinement of a health promotion intervention

Journal:	*BMJ Open*
Manuscript ID	bmjopen-2020-045570.R1
Article Type:	Original research
Date Submitted by the Author:	12-Apr-2021
Complete List of Authors:	Lloyd, Jenny; University of Exeter, University of Exeter Medical School, NIHR Applied Research Collaboration South West Peninsula (PenARC)<br>Bjornstad, Gretchen; University of Exeter, University of Exeter Medical School, Peninsula Childhood Disability Research Unit (PenCRU) and NIHR Applied Research Collaboration South West Peninsula (PenARC)<br>Borek , Aleksandra ; Oxford University, Nuffield Department of Primary Care Health Sciences, Medical Sciences Division<br>Cuffe-Fuller, Beth; University of Exeter, University of Exeter Medical School, Peninsula Childhood Disability Research Unit (PenCRU)<br>Fredlund, Mary; University of Exeter, University of Exeter Medical School, Peninsula Childhood Disability Research Unit (PenCRU) and NIHR Applied Research Collaboration South West Peninsula (PenARC)<br>McDonald, Annabel; University of Exeter, University of Exeter Medical School, Peninsula Childhood Disability Research Unit (PenCRU)<br>Tarrant, Mark; University of Exeter, University of Exeter Medical School, NIHR Applied Research Collaboration South West Peninsula (PenARC)<br>Berry, Vashti; University of Exeter, University of Exeter Medical School, NIHR Applied Research Collaboration South West Peninsula (PenARC)<br>Wilkinson, Kath; University of Exeter, University of Exeter Medical School, Peninsula Childhood Disability Research Unit (PenCRU)<br>Mitchell, Siobhan; University of Exeter, University of Exeter Medical School, NIHR Applied Research Collaboration South West Peninsula (PenARC)<br>Gillett, Annette; University of Exeter, University of Exeter Medical School, Peninsula Childhood Disability Research Unit (PenCRU)<br>Logan, Stuart; University of Exeter, University of Exeter Medical School, Peninsula Childhood Disability Research Unit (PenCRU) and NIHR Applied Research Collaboration South West Peninsula (PenARC)<br>Morris, Christopher; University of Exeter, University of Exeter Medical School, Peninsula Childhood Disability Research Unit (PenCRU) and NIHR Applied Research Collaboration South West Peninsula (PenARC)
**Primary Subject Heading**:	Health services research
Secondary Subject Heading:	Research methods
Keywords:	QUALITATIVE RESEARCH, STATISTICS & RESEARCH METHODS, PREVENTIVE MEDICINE

SCHOLARONE™
Manuscripts

I, the Submitting Author has the right to grant and does grant on behalf of all authors of the Work (as defined in the below author licence), an exclusive licence and/or a non-exclusive licence for contributions from authors who are: i) UK Crown employees; ii) where BMJ has agreed a CC-BY licence shall apply, and/or iii) in accordance with the terms applicable for US Federal Government officers or employees acting as part of their official duties; on a worldwide, perpetual, irrevocable, royalty-free basis to BMJ Publishing Group Ltd ("BMJ") its licensees and where the relevant Journal is co-owned by BMJ to the co-owners of the Journal, to publish the Work in this journal and any other BMJ products and to exploit all rights, as set out in our licence.

The Submitting Author accepts and understands that any supply made under these terms is made by BMJ to the Submitting Author unless you are acting as an employee on behalf of your employer or a postgraduate student of an affiliated institution which is paying any applicable article publishing charge ("APC") for Open Access articles. Where the Submitting Author wishes to make the Work available on an Open Access basis (and intends to pay the relevant APC), the terms of reuse of such Open Access shall be governed by a Creative Commons licence – details of these licences and which Creative Commons licence will apply to this Work are set out in our licence referred to above.

Other than as permitted in any relevant BMJ Author's Self Archiving Policies, I confirm this Work has not been accepted for publication elsewhere, is not being considered for publication elsewhere and does not duplicate material already published. I confirm all authors consent to publication of this Work and authorise the granting of this licence.

For peer review only

The Healthy Parent Carers Programme: mixed-methods process evaluation and refinement of a health promotion intervention

Lloyd J, Bjornstad G, Borek A, Cuffe-Fuller B, Fredlund M, McDonald A, Tarrant M, Berry V, Wilkinson K, Mitchell S, Gillett A, Logan S, Morris C

Jenny Lloyd 01392 722972 (Corresponding author)
J.J.Lloyd@exeter.ac.uk
NIHR Applied Research Collaboration South West Peninsula (PenARC), University of Exeter Medical School, University of Exeter, St Luke's Campus, Heavitree Road, Exeter, EX1 2LU, UK

Gretchen Bjornstad
g.j.bjornstad@exeter.ac.uk
Peninsula Childhood Disability Research Unit (PenCRU) and NIHR Applied Research Collaboration South West Peninsula (PenARC), University of Exeter Medical School, University of Exeter, St. Luke's Campus, Heavitree Road, Exeter, EX1 2LU, UK

Aleksandra Borek
aleksandra.borek@phc.ox.ac.uk
Nuffield Department of Primary Care Health Sciences, Medical Sciences Division, University of Oxford, Radcliffe Observatory Quarter, Woodstock Road, Oxford, OX2 6GG, UK

Beth Cuffe-Fuller
bethany.cuffefuller@nhs.net
Peninsula Childhood Disability Research Unit (PenCRU), University of Exeter Medical School, University of Exeter, St. Luke's Campus, Heavitree Road, Exeter, EX1 2LU, UK

Mary Fredlund
maf215@exeter.ac.uk
Peninsula Childhood Disability Research Unit (PenCRU) and NIHR Applied Research Collaboration South West Peninsula (PenARC), University of Exeter Medical School, University of Exeter, St. Luke's Campus, Heavitree Road, Exeter, EX1 2LU, UK

Annabel McDonald
a.l.mcdonald@exeter.ac.uk
Peninsula Childhood Disability Research Unit (PenCRU), University of Exeter Medical School, University of Exeter, St. Luke's Campus, Heavitree Road, Exeter, EX1 2LU, UK

Mark Tarrant
m.tarrant@exeter.ac.uk
NIHR Applied Research Collaboration South West Peninsula (PenARC), University of Exeter Medical School, University of Exeter, St Luke's Campus, Heavitree Road, Exeter, EX1 2LU, UK

Vashti Berry
V.Berry@exeter.ac.uk
NIHR Applied Research Collaboration South West Peninsula (PenARC), University of Exeter Medical School, University of Exeter, St Luke's Campus, Heavitree Road, Exeter, EX1 2LU, UK

For peer review only - http://bmjopen.bmj.com/site/about/guidelines.xhtml

Kath Wilkinson
K.Wilkinson2@exeter.ac.uk
Peninsula Childhood Disability Research Unit (PenCRU), University of Exeter Medical School, University of Exeter, St. Luke's Campus, Heavitree Road, Exeter, EX1 2LU, UK

Siobhan Mitchell
s.b.mitchell@exeter.ac.uk
NIHR Applied Research Collaboration South West Peninsula (PenARC), University of Exeter Medical School, University of Exeter, St. Luke's Campus, Heavitree Road, Exeter, EX1 2LU, UK

Annette Gillett
A.Gillett@exeter.ac.uk
Peninsula Childhood Disability Research Unit (PenCRU), University of Exeter Medical School, University of Exeter, St. Luke's Campus, Heavitree Road, Exeter, EX1 2LU, UK

Stuart Logan
Stuart.Logan@exeter.ac.uk
Peninsula Childhood Disability Research Unit (PenCRU) and NIHR Applied Research Collaboration South West Peninsula (PenARC), University of Exeter Medical School, University of Exeter, St. Luke's Campus, Heavitree Road, Exeter, EX1 2LU, UK

Christopher Morris
christopher.morris@exeter.ac.uk
Peninsula Childhood Disability Research Unit (PenCRU) and NIHR Applied Research Collaboration South West Peninsula (PenARC), University of Exeter Medical School, University of Exeter, St. Luke's Campus, Heavitree Road, Exeter, EX1 2LU, UK

For peer review only

Abstract

Objectives: Parent carers of children with special educational needs or disability are at risk of poorer mental and physical health. In response to these needs, we co-developed the 'Healthy Parent Carers' (HPC) programme. This study examined views and experiences of participants in the HPC feasibility trial to inform programme refinement.

Intervention, setting and participants: HPC is a peer-led group-based intervention (supported by online materials) for primary carers of disabled children, encouraging behaviours linked with health and wellbeing. It was delivered by two lead and six assistant peer facilitators, in six community sites (one lead and one assistant per group), in South West England over six or twelve sessions. Control participants had online materials only. The trial involved 47 intervention and 45 control parent carers (97% female and 97% white) and eight facilitators (1 male).

Design: A pre-planned mixed methods process evaluation using questionnaires and checklists (during and after the intervention), qualitative interviews with participants post intervention (n=18) and a focus group with facilitators post trial.

Results: HPC was highly acceptable to participants and facilitators and experiences were very positive. Participants thought that the programme increased awareness of what parent carers could and could not change and their self-efficacy to engage in health-promoting behaviours. The intended mechanisms of action (social identification and peer support) matched participants' expectations and experiences. Control participants found the online-only programme flexible but isolating, as there were no opportunities to share ideas and problem-solve with peers, the key function of the programme. Areas for improvement were identified for programme content, facilitator training and delivery.

Conclusion: HPC was acceptable, well-received and offers considerable potential to improve the health of parent carers. Under the pandemic, the challenge going forward is how best to maintain reach and fidelity to function whilst delivering a more virtual programme.

Trial registration: ISRCTN151144652

For peer review only

Strengths and Limitations of this study

- The HPC programme has followed the key principles of intervention development
- Qualitative and quantitative data have been synthesised systematically to refine and optimise the intervention
- The intervention refinement process is transparent, and adaptations reflect the process data collected
- Key uncertainties to be addressed in future research have been identified
- Experiences and views are from a predominantly white population, therefore, are not necessarily representative of ethnically/culturally and linguistically diverse parent carers.

Introduction

Parent carers of children with special educational needs or disability are at increased risk of poorer mental [1-10] and physical health,[2, 3, 6, 7, 11-13] a problem recognised in the NHS Long Term Plan as requiring action to support the personal needs of carers.[14] Parent carers experience challenges to maintaining good health that have implications for their wellbeing and their ability to care for their children and recent reviews conclude that there are insufficient programmes that aim to support parental health, which are likely to be the best strategy to advance both child and family outcomes.[15, 16] In response to this need we co-developed the 'Healthy Parent Carers' (HPC) programme, a community based behaviour change approach to improve health and wellbeing, advocated by Public Health England.[17, 18] This health promotion intervention targets specific behaviours based around a set of universal and evidence-based actions (called CLANGERS) associated with health and wellbeing. CLANGERS stands for Connect, Learn, be Active, take Notice, Give, Eat well, Relax and Sleep.[19] The 'CLANG' component comprises the 'Five Ways to Wellbeing' based on the evidence from the Foresight Project on Mental Capital and Wellbeing.[20] Each of these behaviours is potentially more difficult for parent carers to sustain because of the demands and disruptions of their caring role. The programme involves a range of activities to improve parent carer confidence, motivation and self-efficacy to plan, prioritise and enact these universal actions to improve their own health and wellbeing, whilst expanding their social network and providing peer-to-peer social support.

Intervention Mapping [21] with extensive stakeholder involvement was used to develop programme content and delivery strategies (supplementary documents 1a and 1b) which was piloted with one group of seven parent carers, delivered by two peer facilitators (MF/BM), with whom the intervention was co-created. The findings of this proof-of-concept study and details of the

For peer review only - http://bmjopen.bmj.com/site/about/guidelines.xhtml

intervention development, logic model and content were published previously.[22] Box 1 summarises programme content, format, and delivery.

Insert Box 1

The Feasibility Study

The feasibility study aimed to assess whether the programme could be delivered in the community and evaluated the acceptability of a randomised controlled trial (RCT) design.[23] The trial ran between July 2018 and June 2020. Ninety-two participants were randomised: 47 to the HPC group programme (delivered in 6 community sites across Cornwall, Devon and Somerset) and 45 to the control group, which involved access to the HPC online resources only. The group sessions ran between January and July 2019. Outcome measures were collected at baseline (prior to randomisation), immediately post intervention and six months later (supplementary document 2).

HPC group sessions were delivered by pairs of peer Lead and Assistant Facilitators who were themselves parent carers of children with chronic health conditions. Two Lead Facilitators, experienced in delivering group training to parent carers, were recruited from the Council of Disabled Children (CDC). Nine Assistant Facilitators (including three reserves) were recruited through recommendations from our Stakeholder Advisory Group and through local network adverts. Volunteers were shortlisted and interviewed by telephone. Selection decisions were made based on a practical understanding of the challenges faced by parent carers in relation to their own health and wellbeing with reference to our person specification, and availability to deliver at one of the selected venues. Details of the training programme are in Box 2.

One of the Lead Facilitators delivered the six-week programme to two groups (two sites) and one delivered to four groups (four sites), one of which followed the 12-session format (Box 1). Each Assistant Facilitator was assigned to support delivery in one of the six groups.

Insert Box 2

Figure 1 shows the flow of participants in the trial and details of data collection.

Insert Fig 1

For peer review only - http://bmjopen.bmj.com/site/about/guidelines.xhtml

The aim of this paper is to present mixed methods process data on the motivations of parent carer participants and facilitators to take part in the HPC programme, their views and experiences of receiving/delivering the programme and its refinement in response to these data. Trial findings related to recruitment and retention, fidelity of intervention delivery, the feasibility and acceptability of trial processes and outcome data will be reported separately.[24]

Methods

Patient and Public Involvement

Parent carers have been involved all stages of developing the intervention, designing the feasibility study and refining the programme in light of the findings from this feasibility trial, through the involvement of the Peninsula Cerebra Research Unit (PenCRU) Family Faculty (www.pencru.org/getinvolved/ourfamilyfaculty). The Family Faculty are parent carers who are offered opportunities to be involved in research.

Participants

Recruitment of participants and facilitators to the trial is reported above. For quantitative process evaluation data, all trial participants in both arms were sent online feedback forms after the programme, and all group participants were asked to complete feedback forms after each session. For qualitative process evaluation data collection, participants were purposively sampled for interviews to ensure variation (see Box 3), and all facilitators involved in delivering the programme were invited to a focus group.

Data Collection

Qualitative and quantitative data were collected from participants in both arms and facilitators on programme uptake, reach, training, delivery, and experience as well as suggestions for improvement. Researchers (BCF, JL) who were not involved in programme delivery collected qualitative data. Box 3 presents the measures, (details in supplementary documents 3-10) and sampling process.

Insert Box 3

For peer review only - http://bmjopen.bmj.com/site/about/guidelines.xhtml

Data Analysis

Qualitative data

All qualitative data was uploaded to NVivo (version 12). Taking both an inductive and deductive thematic approach to analysis, we developed a coding framework to categorise the data from participant interviews, facilitator focus group, facilitator support calls, and free-text data from the participant and facilitator questionnaires. Three parent interviews were coded independently by three researchers (BCF, AB, JL) initially and compared to agree the coding framework, which included combining codes and arranging them into higher-level categories to organise the data. While the detailed codes were developed inductively, the categories were more deductive and followed the key areas of interest in the process evaluation, reflecting the interview topic guide. The agreed framework was then used to code the remaining interviews, adding new inductive codes when identified. Facilitator support calls, and free-text data from the participant and facilitator questionnaires were coded by AG and BCF, and 20% were double coded by SM. All interview transcripts (n=18) were coded by BCF, and 50% (n=9) were double-coded by AB and JL. The focus group transcript was coded by BCF and checked by JL and AB. The double-coding and cross-checking of the coding, and regular team discussions on analysis and interpretation helped ensure the quality of analysis and minimise any potential researcher bias.

Quantitative data

Relevant quantitative questionnaire data in relation to participant and facilitator experiences and views were collated and presented descriptively alongside the qualitative findings.

Data Synthesis for Intervention Refinement

Data relevant to programme improvement (i.e. suggestions, critical comments, negative experiences) were extracted from NVivo. These data were coded and sorted into three categories: facilitator training, intervention delivery, and intervention content (including online materials). For each suggestion/comment, the source of data was noted, including when there were mixed or contradictory views. Figure 2 shows the refinement process. Potential refinements were discussed and prioritised using a colour-coded system.

Insert Fig 2

For peer review only - http://bmjopen.bmj.com/site/about/guidelines.xhtml

Findings

Trial participants were aged 42.5 (8.0) years (mean (SD)), with 96% female and 97% white. Figure 1 shows the number of participants who provided each type of process evaluation data. Completeness of questionnaire data was high for participants (>80%) and facilitators (100%). According to facilitators' self-report delivery checklists, 90% of activities were delivered across all groups. Scores from researcher checklists of the nine audio-recorded modules [24] similarly indicated that 91% of activities were delivered. Interviews were carried out with twelve intervention (two per group/site; 11 female, 1 male) and six control participants (one per site; 5 female, 1 male). The focus group involved all lead (n=2 females) and assistant (n=6; 5 females, 1 male) facilitators. The mixed-methods findings are summarised below, with illustrative quotes reported in boxes 4 and 5 and additional quotes and comments in supplementary document 11.

1. Facilitator experiences

1.1. Motivations and expectations

Five out of eight facilitators had experience of facilitating parent carer groups. Most reported being interested in delivering the programme because it fitted with their skills and interests, and offered an opportunity to further develop their professional skills and confidence. Facilitators recognised the need for, and importance of, health and wellbeing training for parent carers, believed in the value of the programme (and group support), and wanted to support other parent carers. All reported interest in contributing to the research and programme development, and five hoped to benefit from the programme themselves.

1.2. Training, preparation and support

Following block 1, 2 and 3 of training (Box 2), all Lead and Assistant Facilitators completed the facilitator training feedback questionnaire. Overall scores were out of 5. Knowledge/understanding and skills/confidence for each block were high at 4.5 and 4.6; 4.5 and 4.3; 4.6 and 4.45, respectively. The only component to decrease over the training was the facilitator's skills and confidence to manage difficult/sensitive issues. Thus, unsurprisingly, in the focus group following programme delivery, they suggested the training could focus more on the delivery style and practice, and managing difficult issues specific to parent carers likely to arise in group discussions. Facilitators suggested that future facilitators could be recruited from those completing the programme, so facilitators would possess a greater understanding of the content and process and be able to build on their own experience of the programme. They also suggested better clarifying the roles and responsibilities of Lead and Assistant Facilitators, and to be matched and trained together. They reported satisfactory support throughout programme delivery (i.e. supervision from CDC, support

For peer review only - http://bmjopen.bmj.com/site/about/guidelines.xhtml

calls with the research team), but particularly valued the sharing and support among fellow facilitators. Nearly three quarters of facilitators believed that sufficient time had been allocated to training.

1.3. Programme content and group delivery

Facilitators perceived the following benefits of the programme for participants: the focus on parent health/wellbeing (rather than their children); the face-to-face group delivery (rather than online); the consistent and predictable session structure; the weekly goal setting (but suggested simplifying 'SMART' to ‘achievable’ goals). They perceived both 6- and 12-week delivery as suitable.

Facilitators found the practical activities an important component (in addition to group discussions) but some activities triggered difficult feelings among some participants. The facilitators reported that some participants disclosed difficult personal issues, which were challenging to address in the group without turning into counselling sessions; thus, the facilitators stressed the importance of training in managing such situations. This was more problematic when groups were small, and the facilitators agreed that 6-8 participants was an optimal group size. The facilitators emphasised the importance of facilitators being parent carers, and how the positive experiences of co-facilitating and the programme helped them reflect on their own health and wellbeing.

Facilitators thought that each session went well, and that participant engagement was high, their average ratings across groups for each session ranging between 4.4 and 4.8 out of 5 for how well they thought the session went and 4.5-5 out of 5 for parent engagement. Facilitator judgement of how connected their groups were following each session was very high and increased over the course of the programme from 5/7 to 7/7. Box 4 presents selected quotes illustrating facilitators’ experiences.

Insert Box 4

2. Participants’ experiences

Below we refer to ‘interviewees’ to indicate the views only of those interviewed and to ‘participants’ to indicate data collected from the questionnaires. Box 5 presents selected quotes illustrating participants’ experiences.

2.1. Motivation to participate and expectations

All interviewees (n=18) reported participating in the programme to focus on and find ways to improve their health and wellbeing. Some wanted to do that to better support their families, and to feel less guilt for taking time to take care of themselves. A few interviewees thought the programme

For peer review only - http://bmjopen.bmj.com/site/about/guidelines.xhtml

took place at the right time for them: when they were able, ready and available to participate and focus on their health. Most interviewees also reported wanting to contribute to research and help other parent carers, especially as they perceived little available support for parent carers.

The majority of interviewees in the group programme reported positive aspects of face-to-face delivery, such as peer support and dedicated time to share and discuss issues affecting their wellbeing. A few participants, mainly from the online-only control arm, reported that the flexibility of the online programme enabled them to engage with the programme more easily than attending face-to-face groups. Overall, group-based delivery was preferred, including those who had not experienced it, as groups were perceived to instigate a stronger commitment to engage with the programme.

2.2. Experiences of the group programme and its impact

Positive experiences: Overall 84% (37/44) of participants were satisfied or very satisfied with the group programme, with 67% (28/42) finding it useful in helping them to improve their health and wellbeing. Most of the interviewees echoed this, noting the positive impact of the programme on them and their families. Two reported that it 'pushed them out of their comfort zones' by addressing some uncomfortable but important issues. Several participants commented that the programme should be rolled-out to benefit all parent carers.

Groups: All participants who responded to the post intervention questionnaire reported feeling included and part or very much 'included and part of the group' with 85% (17/20) indicating that session length was 'about right'. All intervention interviewees described one of the main benefits of the programme as having the opportunity to discuss and share with other parent carers in a supportive, empathetic, safe, and respectful group context. They highlighted the value of peer support and discussions in motivating change. Interviewees also perceived the group programme as providing more than support groups because of the practical activities and exploring barriers and solutions in specific areas. Some were surprised by the small group size but liked it as it enabled them to participate and get to know each other and bond as a group; yet, most thought that slightly larger groups would provide more varied perspectives. Interviewees noted also that the group programme gave them time to focus on themselves and permission (to take time) to take care of themselves.

Peer facilitators: All participants who responded to the questionnaire were satisfied or very satisfied with the way the programme was delivered, and interviewees highlighted the critical importance of the facilitators being parent carers, enabling shared understanding and empathy. Participants valued

For peer review only - http://bmjopen.bmj.com/site/about/guidelines.xhtml

facilitators delivering the programme together ('complementing each other' and 'bouncing off each other'), facilitating learning through group discussions (rather than 'teaching'), creating positive group context (with facilitators seen as part of the groups), and being knowledgeable, understanding and kind.

Content and activities: Interviewees generally had positive comments about programme content and preferences for different activities. The content and activities were mainly valued for providing structure and prompts to group discussions and focus on different life areas. The promoted messages were seen as 'common sense' and applicable to all people, but more challenging to parent carers. The programme reminded participants about the importance of health/wellbeing, and discussing/sharing reinforced that, thus prompting them to make changes.

Reflecting and setting small achievable goals: Interviewees valued the programme's focus on simple and meaningful actions (CLANGERS) that increased their awareness of areas of their lives in which they could make positive changes (while also helping accept things that they could not change). Interviewees also valued focusing on small steps that they can take, setting achievable goals at each session/module, and then reflecting on them. This helped them feel more in control of doing something positive about their health/wellbeing. 76% (35/46) reported making changes.

Less positive experiences: Despite overwhelmingly positive experiences, nine participants did not find the programme useful, with five indicating ambivalence. A few interviewees reported a less positive experience and *not* making changes, mainly due to factors outside the programme (described below); however, they still valued the raised awareness and 'hope' it provided. This was reflected in the quantitative feedback with 24% (11/46) reporting not making changes. A few interviewees reported feeling apprehensive and nervous at the start of groups; finding different activities or content difficult, challenging or less appealing; feeling uncomfortable with others in the group (e.g., due to expressed views/comments); perceiving less shared experience and challenges with others in the group (e.g., due to personal or system-related contexts).

2.3. Views on online materials

Participants' experiences of, and views on, using online materials were mixed. Interviewees attending the groups reported finding the online materials unnecessary or just reinforcing the sessions. Interviewees from the control group reported some positive experiences of the online-only programme (valuing videos as an engaging way of providing information) but found some content less relevant or helpful. Overall satisfaction of control participants with the programme was good, although lower than intervention participants (66% vs 84%), with only 38% (11/29) finding the programme 'useful in helping them to improve their health and wellbeing', although 65% (20/31)

For peer review only - http://bmjopen.bmj.com/site/about/guidelines.xhtml

reported making changes.

Interviewees in both study arms described the importance of group discussions in enhancing the learning and programme impact. Without group sessions, some found the online-only programme isolating (without opportunities to discuss, share ideas and problem-solve with other parent carers) and harder to focus on because of lacking reminders and scheduled time (with other matters taking priority).

2.4. Factors influencing ability to engage with and benefit from the programme

Interviewees in both study arms reported factors that affected their ability to attend the sessions or use online materials, including: other (unexpected) commitments, lack of time, inadvertent events (their or children's illness), access/transport, and childcare. A few also discussed external factors out of their control (e.g., social care needs, access to respite, work situation) that affected their wellbeing and ability to benefit from the programme, reinforcing the importance of participating in the programme at the right time in their lives.

Insert Box 5

3. Programme refinement

The process evaluation confirmed that the HPC programme was highly acceptable to both parent carers and facilitators with the vast majority expressing positive experiences; however less positive experiences and suggestions were carefully considered and used in the refinement process (Figure 2). Several potential areas for improvement were identified and, where possible, incorporated into the refined HPC programme. Key changes are outlined below.

3.1. Optimising session and online content

- Wording of the online materials simplified to increase understanding, engagement, and usage.
- Following suggestions and less positive views on goal setting, 'SMART' replaced with 'achievable' goals, and examples of SMART goals were added to online materials and delivery manual to increase understanding.
- Create or select videos more relevant to parent carers (as some participants were less positive about some of the more generic health-related videos).

3.2. Optimising training

- Explanation of the rationale for certain activities included to increase understanding of their purpose.
- Training in managing challenges that specific content/activities may generate, and worked examples on how these may be addressed.
- Training in how best to present and deliver the videos to elicit discussion around the key 'take away' messages.

For peer review only - http://bmjopen.bmj.com/site/about/guidelines.xhtml

- Facilitators to complete the online modules prior to training so that that they are familiar with the CLANGERS and resources, providing more time to focus on delivery strategies.
- Group dynamics session refined so that it is more practical and interactive (scenarios and practice included in how to build, enhance and maintain group cohesion under challenging situations; how to find commonalities in shared experience despite having differing challenges/situations).
- Delivery process for each of the CLANGERS to be modelled in detail using 'Connect' (as it is the most challenging of the CLANGERS to deliver and experience) to ensure that facilitators understand the application of the programme's theory of change.
- Trainers asked to create and disseminate a FAQ document to follow up any questions/concerns not addressed in the training due to time constraints.
- Increased focus on developing delivery skills using modelling techniques to improve confidence and quality of delivery (e.g. modelling good responses to parent questions, supporting parents who are struggling).
- Roles and responsibilities of the Lead and Assistant Facilitators clearly explained to avoid misunderstanding and enhance team working.

3.3. Optimising delivery

- Lead and Assistant Facilitators to communicate with each other prior to the first session (using the 'preparation' checklist) to allay any delivery fears/concerns.
- Prior to session 1, to allay any participant concerns before joining the group, facilitator photo, with a written introduction to be sent to participants; Lead Facilitators to call participants to introduce themselves, ascertain any practical support required and take steps to provide this support where possible.
- Advice on managing difficult situations added to the delivery manual.
- Description of roles and responsibilities of the Lead and Assistant Facilitators added to the delivery manual.
- Key 'take away' messages from each video added to the delivery manual.
- Delivery manual adapted to accommodate the 12x2 hour format to improve coherence.
- Completion of a simple participant feedback form at the end to provide information to improve future group delivery.
- Following participants' and facilitators' less positive comments about smaller groups, change minimum viable group number from 4 to 6 to increase the range of perspectives and create better conditions for peer-to-peer learning and support.

Discussion

This paper presents facilitator and parent carers' views on and experiences of the HPC programme from the feasibility RCT, which have informed the refinement of the programme. We are now exploring implementation uncertainties, funded by the Economic and Social Research Council Impact Accelerator Account Strategic Initiative Award (ES/T501906/1) in preparation for a further evaluation of programme effectiveness. Fidelity to delivery and qualitative data show that the training of facilitators was successful. The group programme was valued for providing peer support and practical activities, where difficult and emotional conversations are facilitated and explored. This is a key function of the programme, which accounted for the high levels of satisfaction and reported

For peer review only - http://bmjopen.bmj.com/site/about/guidelines.xhtml

impact on both the parent and the wider family. It appears that this aspect of the programme necessitates larger groups (6-12) to allow for varied perspectives and a 'facilitation' rather than 'counselling' approach. Group size, therefore, will be explored as part of intervention fidelity to function [25, 26] in the definitive trial.

As intended, consistent with our logic model, participants reported that the programme increased awareness of what parent carers could and could not change and their self-efficacy to engage in health-promoting behaviours (CLANGERS). Participant motivations and expectations showed that, overall, the target group was reached (i.e. those who wanted to and reported feeling ready to do something to improve their own health/wellbeing), with the intended mechanisms of action (social identification and peer support) matching the participants' expectations and experiences.

Facilitators reported that the relationship between the Leads and Assistants was important for effective delivery and that clarity on and practice of these different roles were a crucial part of training and quality delivery. Both intervention participants and facilitators thought that, to deliver the programme effectively, Leads and Assistants needed to have completed the programme themselves and that the development of a network of facilitators to share experiences of delivery using support calls and/or online meetings was important in supporting them to improve their practice.

The strength of this research is that it has systematically followed key principles of intervention development [27, 28] and refinement, using a dynamic, iterative and creative process with extensive stakeholder consultation, where the developers have been open to change based on data collected in a series of iterations.[22, 23] We examined how the intervention will be evaluated in the next phase of research and identified learning and key uncertainties to be addressed, such as blended (online and face-to-face) delivery of group sessions, commissioning and implementation. Reporting mixed methods data on participant views and experiences and the subsequent refinement process in feasibility studies is recommended.[29] Not only does it increase knowledge about the intervention refinement/optimisation process but it also allows others to link up intervention development processes and subsequent trial outcomes.

However, the study has limitations. The lack of ethnic diversity in South West England, where this study was conducted, meant that the sample was ethnically homogeneous, thus experiences and views may not represent parent carers from different cultures and contexts. Most work on culturally and linguistically diverse groups and parenting interventions has been conducted in the USA and is equivocal about whether outcomes differ by ethnicity.[30, 31] However, adapting parenting interventions for different ethnic groups poses many issues. Adaptation may neither be practical or a

For peer review only - http://bmjopen.bmj.com/site/about/guidelines.xhtml

desirable service model for multi-ethnic European cities.[32] Presently, we do not have the available data as individual trials are not powered to test intervention effects by ethnicity.

Nevertheless, we will explore in future evaluative work with mixed and single ethnic groups, how ethnicity, social disadvantage, gender and/or other personal factors might intersect to exacerbate the health issues arising from being a parent carer,[33] and affect the development of a shared group identity and implementation more generally.

This study has helped to refine the programme in many aspects; however, there remain a number of barriers to parent carers' capacity to engage in all group sessions and benefit from the programme. We need to explore these barriers in further detail and the extent to which they can be mitigated to enhance accessibility to participate in the programme. As we write, the COVID-19 pandemic has meant social distancing and increased challenges to running groups. We are therefore keen to explore to what extent the group programme could be delivered virtually using videoconferencing, which may overcome some other barriers to participation, but we would need to evaluate the extent to which peer support and cohesive support of the group is maintained.

Conclusion

The format, content and delivery of the HPC programme was highly acceptable to participants and for facilitators to deliver. The process evaluation data enabled programme refinement to optimise impact going forward. Although the programme focuses on promoting health and wellbeing at an individual level (i.e. individual psychological and behavioural change), we acknowledge the importance of other factors at interpersonal, community and societal levels that affect parent carers' health and wellbeing, such as access to services, negative public attitudes towards disability, which in turn impact on parent carers' capacity to make and sustain changes. The programme does, however, provide support and hope for those who find it difficult, both practically and psychologically, to attend to their own wellbeing. The challenge we face going forward is delivery of the programme in the shadow of COVID-19. The team plan to explore how this might be reimagined to accommodate a new way of supporting parents, minimising risk to health, whilst delivering an accessible and inclusive package that maintains fidelity to function.

(4484 word count)

Declarations

Funding

For peer review only - http://bmjopen.bmj.com/site/about/guidelines.xhtml

This paper presents independent research funded by the National Institute for Health Research (NIHR) under its Research for Patient Benefit (RfPB) Programme (Grant Reference Number PB-PG-0317-20044). We also acknowledge support from the National Institute for Health Research Applied Research Collaboration South West Peninsula. The views and opinions expressed in this paper are those of the authors and not necessarily those of the National Institute for Health Research or the Department of Health and Social Care. Delivery of the Healthy Parent Carers programme was funded by a grant from the National Lottery Community Fund through the Reaching Communities programme (Grant number 10343962) and supported by the Public Health England, Kernow Clinical Commissioning Group, Northern, Eastern and Western Devon Clinical Commissioning Group. Peer review of the study design was provided during the NIHR funding application process. The funders have had no involvement in the writing of the manuscript and will not have any role during its execution, analyses, interpretation of the data, writing of the report, or the decision to submit the report for publication.

For peer review only

Ethics approval

The study procedures were approved by University of Exeter Medical School Research Ethics Committee (reference 18/06/174). All participants gave consent to participate in the study and safeguarding protocols were also included in the study protocol and approved by the ethics committee.

Competing interests

None

Author contributions

All authors contributed. CM led the development and preliminary evaluation of the programme and was principal investigator of this study. GB managed the project, including overseeing day-to-day recruitment and data collection. GB and CM drafted the initial study design with input from AB, JL VB, MT, MF, AM and SL. AB, AM, MF, and CM designed the original programme. KW recruited programme facilitators and arranged delivery sites. GB, BCF, KW and AG recruited participants and facilitated data collection. AM, MF, BCF, and KW planned, prepared, and delivered facilitator training and support. JL led the programme refinement process supported by GB, AB, VB and MT. AB, BCF, AG, SM and JL coded and analysed the qualitative data. SL facilitated infrastructure support through

For peer review only - http://bmjopen.bmj.com/site/about/guidelines.xhtml

the National Institute for Health Research Applied Research Collaboration South West Peninsula. All authors served on the Trial Management Group, contributed to drafting this paper, and approved the final manuscript. The study sponsor is the University of Exeter.

Acknowledgments

We are grateful for the contributions of the PenCRU Family Faculty and feedback provided by the participants in both trials arms. We also thank our colleagues within the wider PenCRU team, Katharine Fitzpatrick for organising the parent carer working group meetings and Tania Hind for administrative support.

Data availability statement

The datasets analysed during the current study are available from the corresponding author on reasonable request.

Licence statement

The Submitting Author accepts and understands that any supply made under these terms is made by BMJ to the Submitting Author unless you are acting as an employee on behalf of your employer or a postgraduate student of an affiliated institution which is paying any applicable article publishing charge ("APC") for Open Access articles. Where the Submitting Author wishes to make the Work available on an Open Access basis (and intends to pay the relevant APC), the terms of reuse of such Open Access shall be governed by a Creative Commons licence.

List of Boxes

Box 1. Healthy Parent Carer's Programme and online materials

Box 2. Facilitator training programme

Box 3. Process evaluation measures and sampling

Box 4. Selected quotes illustrating facilitators' experiences

Box 5. Selected quotes illustrating participant' experiences

List of Figures

Figure 1. Flow of participants and measures

Figure 2. Programme refinement process

List of Supplementary Documents

Supplementary document 1a - HPC intervention details

Supplementary document 1b – TIDIER checklist

Supplementary document 2 - Study protocol publication

Supplementary document 3 - Facilitator training feedback questionnaire

Supplementary document 4 - Facilitator delivery checklist (Module 1)

Supplementary document 5 - Facilitator focus group topic guide

Supplementary document 6 - Participant end of session questionnaire

Supplementary document 7 - Participant end of programme questionnaire

Supplementary document 8 - Participant 6 month follow up questionnaire

Supplementary document 9 - Participant interview topic guide (intervention)

Supplementary document 10 - Participant interview topic guide (control)

Supplementary document 11 - Additional quotes and comments from facilitators and participants

For peer review only

For peer review only - http://bmjopen.bmj.com/site/about/guidelines.xhtml

Boxes

Box 1. Summary of the Healthy Parent Carer's Programme (including the online materials)

<u>**Face-to-face sessions**</u>

Content - Activities based on CLANGERS, an extension of the '5 ways to wellbeing'.

- Connect, Learn, be Active, Notice, Give, Eat well, Relax and Sleep.

Format – 12 modules over 24 hours

Setting – Community sites (2 special schools, 1 children's hospice, 1 Parent Carer Forum premises, 1 adult learning community venue, and 1 hotel regularly used for parent carer meetings).

Delivery – Six four-hour daytime sessions (comprising 2 modules per session) or 12 two-hour evening sessions (one module per session) delivered to groups of 4-12 parent carers.

Personnel - One Lead and one Assistant Facilitator per group.

<u>**Online materials**</u>
Included written content which provided participants with information on the CLANGERS, note-taking space to reflect on their own thoughts and templates for participants to develop their own goals and action plans. Audio and video recordings were also provided to illustrate each of the CLANGERS.

The content related to each module was released to participants in each group after it was delivered in their specific group sessions.

For peer review only - http://bmjopen.bmj.com/site/about/guidelines.xhtml

Box 2. Facilitator Training Programme

FORMAT

- **Block 1** - 2 days (Lead Facilitators only). November 2018
- **Block 2 -** 2 days (Lead and Assistant Facilitators). November 2018 (three weeks following Block 1)
- **Block 3 –** 1 day (Lead and Assistant Facilitators). April 2018 (after delivery of cohort 1)

CONTENT

- **Block 1** Overview of programme; exploration of wellbeing and the CLANGERS; facilitator roles and responsibilities; modelling delivery; research processes; safeguarding
- **Block 2** Overview of programme; exploration of wellbeing and the CLANGERS; facilitator roles and responsibilities; facilitator skills and competencies; group facilitation; managing group dynamics; modelling delivery; practicing delivery; research processes; safeguarding.
- **Block 3** Refresher training; review of CLANGERS, facilitator reflections, research processes, safeguarding.

PERSONNEL –Researchers and two parent carers (MF and BM) who co-produced the programme and delivered the proof of concept pilot.

FACILITATOR RECRUITMENT - Parent carers who are senior facilitators of the Council for Disabled Children's Expert Parent Programme were referred to us to become Lead Facilitators, based on their experience in developing and facilitating programmes for parent carers.
Assistant facilitators were recruited through adverts shared through contacts in the project Stakeholders Advisory Group. Adverts included information about the role and person specification criteria. Applicants were interviewed by a researcher and selection decisions were made by the research team.

SAFEGUARDING - The safeguarding protocol for facilitators was outlined in training and provided in the delivery manual. If any concerns arose during the programme, facilitators were instructed to inform the study team and follow the protocol, which included a reporting flow chart and information for Multi-Agency Referral Units and other relevant contacts at each site.

For peer review only

Box 3. Process evaluation measures and sampling

Facilitator training feedback questionnaire: To assess self-reported knowledge, understanding, skills and confidence to deliver the intervention and facilitator reflections on the training. Data were collated and averaged to provide an overall score out of 5 for each training block for knowledge and understanding (of the programme, facilitation techniques, developing a positive group dynamic and facilitator roles and responsibilities), skills and confidence (to present information, lead activities, create a positive group dynamic, manage time and difficult situations). (supplementary document 3)

Facilitator support calls: To understand and respond to delivery challenges (two per site, one following week 1 and one halfway through the programme).

Facilitator delivery checklists*:* To understand experiences and views on group delivery (weekly checklist, also included a check on content delivered) (Module 1 example, supplementary document 4)

Focus group with facilitators: To understand Lead and Assistant Facilitator experiences and views on training, delivery, and programme content (supplementary document 5). All Lead and Assistant Facilitators involved in delivering the programme were invited to attend the focus group, which took place at a meeting room within the University of Exeter. The two-hour focus group took place once all groups were completed, led by JL and supported by BCF (researchers not involved in delivering the programme). The focus group was audio-recorded and transcribed verbatim (with any potentially identifiable information anonymised).

Participants' feedback questionnaires: To understand participant (control and intervention) experiences and views on programme content and delivery at the end of each group session (supplementary document 6) and at the end of the programme (supplementary document 7) and 6 month follow up (supplementary document 8).

Participant phone interviews: To understand and explore participant experiences, views and engagement with the group sessions and online materials (intervention and control). (supplementary documents 9 and 10). Twelve intervention (two per group/site) and 6 control participants (one per site) were sampled to ensure that two out of the four male carers in the study (1 control and 1 intervention) were interviewed and the range of parent carer challenges were represented (selection was based on Lead Facilitator comments and participant end of programme feedback). Interviews were carried out by a researcher not involved in delivering the programme (BCF) and were audio-recorded and transcribed verbatim, with names and other identifying information changed for confidentiality. Interviews took place as soon as possible after participants completed their post-intervention measures and before the six-month follow-up.

For peer review only

For peer review only - http://bmjopen.bmj.com/site/about/guidelines.xhtml

Box 4. Selected quotes illustrating facilitators' experiences

1.1. Motivations and expectations

I feel that this training is a natural continuation to take forward the previous training I have been involved in. I am passionate about empowering parents with high quality information, support and advice. (Facilitator pre-training questionnaire)

For me it was a mixture of interlinked personal and professional reasons… I'm keen to do good in the world, if I can, keen to use some of my professional skills and enhance my professional skills while doing that. (AF, focus group)

I know the power of groups and supporting each other… I know it's so important to have people that understand and support you, because it can be lonely. I had lots of hope that this would help other people and, yes, it definitely was fulfilled. (AF, focus group)

1.2. Training, preparation and support

Parent carers are a very specific group to deliver to and maybe something that is more specific around the baggage, if you'll excuse me for using that word. The baggage that parent carers bring into the room is extremely specific and the group dynamic isn't managing conflict, usually, with parent carers. It's very different. (…) I always find parent carers very quick to bond and support each other. Very, very quick; they really do move forward in that way. But when you've got an individual... Something about managing the individual rather than the group as a whole. (LF, focus group)

I didn't know enough about the programme before I actually joined in as an assistant… now, it would be fine going back but when you suddenly do the programme you haven't got a clue what's necessarily coming up next and you don't know how, emotionally, you're going to feel. (…) It probably would have been easier if I'd done the programme myself, because I could have then looked back at some notes, so any things that I come across being difficult, I would have had a clearer focus rather than what's actually going on with me at the time. (AF, focus group)

AF: For me, the support calls were more about the sharing of experiences - and the issues and part of the process and the research as well. I guess you [the researcher] were kind of in research mode to some extent in those calls. But the support seemed to me to be within the facilitator team.
LF: I agree absolutely with that. Totally. I think that the relationship that we had as co-facilitators is where I drew my support about anything that wasn't just on a practical basis.
AF: And that's different levels of support needed. (Focus group)

1.3. Programme content and group delivery

The repetitiveness of the way it was delivered really helped parents to predict and understand where they were going, so it put them in a very comfortable space as well. (…) Because every CLANGER is quite a repetitive process and it's unpicking the meanings, lots of self-reflection and we were using the collated feedback to constantly cross-reference and compare... (LF, focus group)

For peer review only - http://bmjopen.bmj.com/site/about/guidelines.xhtml

I found it hard work… and it was very intense and it was more counselling… because people were really disclosing lots of things about their lives and that way… the impact on us as facilitators was more intense experience… and whilst I think we didn't have anything we couldn't handle between us, it actually became more than the course is meant to be. (LF, focus group)

I think one of the things that I definitely felt was the relationship between the trainer and the assistant facilitator is amazing and I definitely feel like the absolute bonus is I've gained a really good friend who I think going on it together actually benefits our health and wellbeing, just delivering it together. It is really positive. (AF, focus group)

(see supplementary document 11 for additional quotes/comments)

Box 5. Selected quotes illustrating participants' experiences

2.1. Motivation to participate and expectations

I feel that being a parent-carer is really draining on your mental health and also I wanted to try and get healthy anyway, especially with it being January. (…) a bit of knowledge maybe about the way I feel and maybe a bit less guilt, the fact that I feel that I need to take time out for myself. (P13, control)

I just thought it could be useful because a lot of the time I find as a parent carer all the focus is on the children which is obviously the main thing but the parents need to be healthy and happy to give the children the best. (P8, intervention)

2.2. Experiences of the programme and its impact

It was fantastic. I think it was really well done. (…) I think it's changed my life. (…) I think the best thing was going to the group, meeting everybody in the group, just meeting like-minded people, having a break from life and being in a different place for a while, reflecting on aspects of my life that I didn't really think about or had put away, and I learnt more about myself. (P5, intervention)

Meeting people who understand and 'get it ' reduced my isolation. Having parent carers as facilitators helped a lot. Having 12 structured sessions made me commit to it and focus on my own health. Having structured fun informative sessions helped a lot - I definitely went through a process of change. The use of humour was really important to me - and learning to be more resilient. (End-of-programme feedback form).

A lot of it is very common sense stuff. (…) It's like a revelation without it being a revelation, because you know all that stuff, but you don't take the time to think about it and it was just very much about focusing on us and improving things for ourselves before we can do it for everyone else really. (P3, intervention)

Unfortunately, I think we were one of the groups that was very small, which was good in some ways because you got to know the people better and you had a bit more time, but then on the other side, at one point there was only two of us there and you've not got everybody's stories, you've not got everybody's experiences, it was just a couple of us. (P4, intervention)

For peer review only

If the group was a little bit bigger…you would get more input and there would be more discussion because even though we were able to discuss things, I think a bit more of an open, broader amount of different people with different lives would make it a lot more interesting. (P6, intervention)
Was there anything that I didn't like? I think the only thing that made me feel... was sometimes like the icebreaker thing, but that's just me feeling nervous about (…) sometimes in group situations if I'm asked my opinion on something or asked to think about something and you've got to kind of think quickly, I get really nervous. (…) I think at the beginning also you are worried about what will people think of me and how will I be perceived by other people. You don't want to say the wrong thing… (P2, intervention)

2.3. Views on online materials

Initially I was curious and then I think it dwindled off because I felt like the course was covering everything so thoroughly that would it add anything to what I had already had? (P4, intervention)

What was there was absolutely fantastic and I think the videos were a good way of doing it because I am better at learning through watching the videos and being able to go back to them rather than just having loads and loads of information to read… (P18, control)

I found doing the online course quite isolating. Personally, I would have made more changes if I had been in the group. (…) I have ticked the box, I have done it (…) I found myself thinking about everything else that I should be doing, whereas if I had gone to the group and they were watching that video… you are switched on to be watching that video, whereas for me, I was there thinking, 'Okay, I have got to do this, so I will sit down and do this… and then my mind… at one point, I was so tired that I was thinking, 'Oh gosh, I have got to go and make lunch'… (P18, control)

2.4. Factors influencing ability to engage with and benefit from the programme

…on some days you are impacted by inadvertent events and there is no relaxation, there is five hours' sleep. That's just the way it is. (P14, control)

I have some really quite big social care needs and health care needs of my own that are not being met and so it is much more difficult to sit there going, "Ah well, have I eaten properly today?" But those things don't exist so it's better than nothing. (…) But I think it's working really well for some people. Yes, I can see that other members of the group were getting masses more out of it than I was. So I think if it's the right thing for you then it's clearly way more beneficial. (P2, intervention)

I couldn't really make out whether I liked it or not. It was alright, it was pleasant enough, but I think I've got a whole bunch of underlying issues that are not being addressed… for some people it was clearly really hitting the spot… and for me, I'm going, if I don't actually get some proper mental health care and social care support I can't really look… well I can look at it but I can just go, "Yes, that will be nice, one day, maybe, mmm, okay". (…) (Interviewer: What did you not like or not find helpful, if anything?) I suppose the assumption that's it in my power to change some of these things when it just isn't. (P2, intervention)

For peer review only

For peer review only - http://bmjopen.bmj.com/site/about/guidelines.xhtml

References

1. Barlow J, Cullen-Powell L, Cheshire A. Psychological well-being among mothers of children with cerebral palsy. *Early Child Dev Care*. 2006;176(3-4):421-8.
2. Brehaut JC, Kohen DE, Raina P, et al. The health of primary caregivers of children with cerebral palsy: how does it compare with that of other Canadian caregivers? *Pediatrics*. 2004;114(2):e182-e91.
3. Brehaut JC, Kohen DE, Garner RE, et al. Health among caregivers of children with health problems: Findings from a Canadian population-based study. *Am J Public Health*. 2009;99(7):1254-62.
4. Emerson E. Mothers of children and adolescents with intellectual disability: social and economic situation, mental health status, and the self-assessed social and psychological impact of the child's difficulties. *Journal of Intellectual Disability Research*. 2003;47(4-5):385-99.
5. Gallagher S, Phillips AC, Oliver C, et al. Predictors of psychological morbidity in parents of children with intellectual disabilities. *J Pediatr Psychol*. 2008;33(10):1129-36.
6. Lach LM, Kohen DE, Garner RE, et al. The health and psychosocial functioning of caregivers of children with neurodevelopmental disorders. *Disabil Rehabil*. 2009;31(9):741-52.
7. Murphy NA, Christian B, Caplin DA, et al. The health of caregivers for children with disabilities: caregiver perspectives. *Child Care Health Dev*. 2007;33(2):180-7.
8. Oelofsen N, Richardson P. Sense of coherence and parenting stress in mothers and fathers of preschool children with developmental disability. *Journal of Intellectual and Developmental Disability*. 2006;31(1):1-12.
9. Olsson MB, Hwang C. Depression in mothers and fathers of children with intellectual disability. *Journal of Intellectual Disability Research*. 2001;45(6):535-43.
10. Singer GH, Floyd F. Meta-analysis of comparative studies of depression in mothers of children with and without developmental disabilities. *Am J Ment Retard.* 2006;111(3):155-69.
11. Arim RG, Miller AR, Kohen DE, et al. Changes in the health of mothers of children with neurodevelopmental disabilities: An administrative data study. *Res Dev Disabil*. 2019;86:76-86.
12. Lee M, Park C, Matthews AK, et al. Differences in physical health, and health behaviors between family caregivers of children with and without disabilities. *Disabil Health J.* 2017:565-70.
13. Tong HC, Haig AJ, Nelson VS, et al. Low back pain in adult female caregivers of children with physical disabilities. *Arch Pediatr Adolesc Med*. 2003;157(11):1128-33.
14. National Health Service. The NHS Long Term Plan 2019. https://www.longtermplan.nhs.uk/wp-content/uploads/2019/08/nhs-long-term-plan-version-1.2.pdf (accessed 30 Mar 2021)
15. Bourke-Taylor HM, Lee D-CA, Tirlea L, et al. Interventions to Improve the Mental Health of Mothers of Children with a Disability: Systematic Review, Meta-analysis and Description of Interventions. *J Autism Dev Disord*. 2021. DOI: 10.1007/s10803-020-04826-4
16. Ogourtsova T, O'Donnell M, De Souza Silva W, et al. Health coaching for parents of children with developmental disabilities: a systematic review. *Dev Med Child Neurol*. 2019;61(11):1259-65.
17. South J. A guide to community-centred approaches for health and wellbeing London: Public Health England/NHS England. 2015. https://assets.publishing.service.gov.uk/government/uploads/system/uploads/attachment_data/file/768979/A_guide_to_community-centred_approaches_for_health_and_wellbeing__full_report_.pdf (accessed 30 Mar 2021)

For peer review only - http://bmjopen.bmj.com/site/about/guidelines.xhtml

18. Johnstone P. Realising the potential of community assets to improve our health and wellbeing: Public Health England; 2017 https://publichealthmatters.blog.gov.uk/2017/09/27/realising-the-potential-of-community-assets-to-improve-our-health-and-wellbeing. (accessed 30 Mar 2021)
19. Hammond P. Staying Alive: How to Get the Best Out of the NHS - advice from a doctor. London: Quercus; 2015. 320 p.
20. Aked J, Cordon C, Marks N, et al. Five Ways to Wellbeing: A report presented to the Foresight Project on communicating the evidence base for improving people's well-being. London: New Economics Foundation; 2008.
21. Bartholomew Eldredge LK, Markham CM, Ruiter RA, et al. Planning health promotion programs: an intervention mapping approach. 4th ed. San Francisco: Jossey-Bass; 2016.
22. Borek AJ, McDonald B, Fredlund M, et al. Healthy Parent Carers programme: development and feasibility of a novel group-based health-promotion intervention. *BMC Public Health*. 2018;18(1):270.
23. Bjornstad G, Wilkinson K, Cuffe-Fuller B, et al. Healthy Parent Carers peer-led group-based health promotion intervention for parent carers of disabled children: protocol for a feasibility study using a parallel group randomised controlled trial design. *Pilot Feasibility Stud*. 2019;5(1):1-13.
24. Bjornstad G, Cuffe-Fuller B, Ukoumunne OC, et al. Healthy Parent Carers: feasibility randomised controlled trial of a peer-led group-based health promotion intervention for parent carers of disabled children. Under review.
25. Moore GF, Evans RE, Hawkins J, et al. From complex social interventions to interventions in complex social systems: Future directions and unresolved questions for intervention development and evaluation. *Evaluation (Lond)*. 2019;25(1):23-45.
26. Moore GF, Audrey S, Barker M, et al. Process evaluation of complex interventions: Medical Research Council guidance. *BMJ*. 2015;350:h1258.
27. O'Cathain A, Croot L, Duncan E, et al. Guidance on how to develop complex interventions to improve health and healthcare. *BMJ Open*. 2019;9(8):e029954.
28. Craig P, Di Ruggiero E, Frohlich KL, et al. Taking account of context in population health intervention research: guidance for producers, users and funders of research. NIHR Journals Library. 2018.
29. O'Cathain A, Hoddinott P, Lewin S, et al. Maximising the impact of qualitative research in feasibility studies for randomised controlled trials: guidance for researchers. *Pilot Feasibility Stud*. 2015;1:32.
30. Reid MJ, Webster-Stratton C, Beauchaine TP. Parent training in head start: a comparison of program response among African American, Asian American, Caucasian, and Hispanic mothers. *Prev Sci.* 2001;2(4):209-27.
31. van Mourik K, Crone MR, de Wolff MS, et al. Parent Training Programs for Ethnic Minorities: a Meta-analysis of Adaptations and Effect. *Prev Sci.* 2017;18(1):95-105.
32. Castro FG, Barrera M, Jr., Holleran Steiker LK. Issues and challenges in the design of culturally adapted evidence-based interventions. *Annu Rev Clin Psychol*. 2010;6:213-39.
33. Berghs M, Atkin K, Graham H, et al. Implications for public health research of models and theories of disability: a scoping study and evidence synthesis. *Public Health Research*. 2016;4(8).

For peer review only

For peer review only - http://bmjopen.bmj.com/site/about/guidelines.xhtml

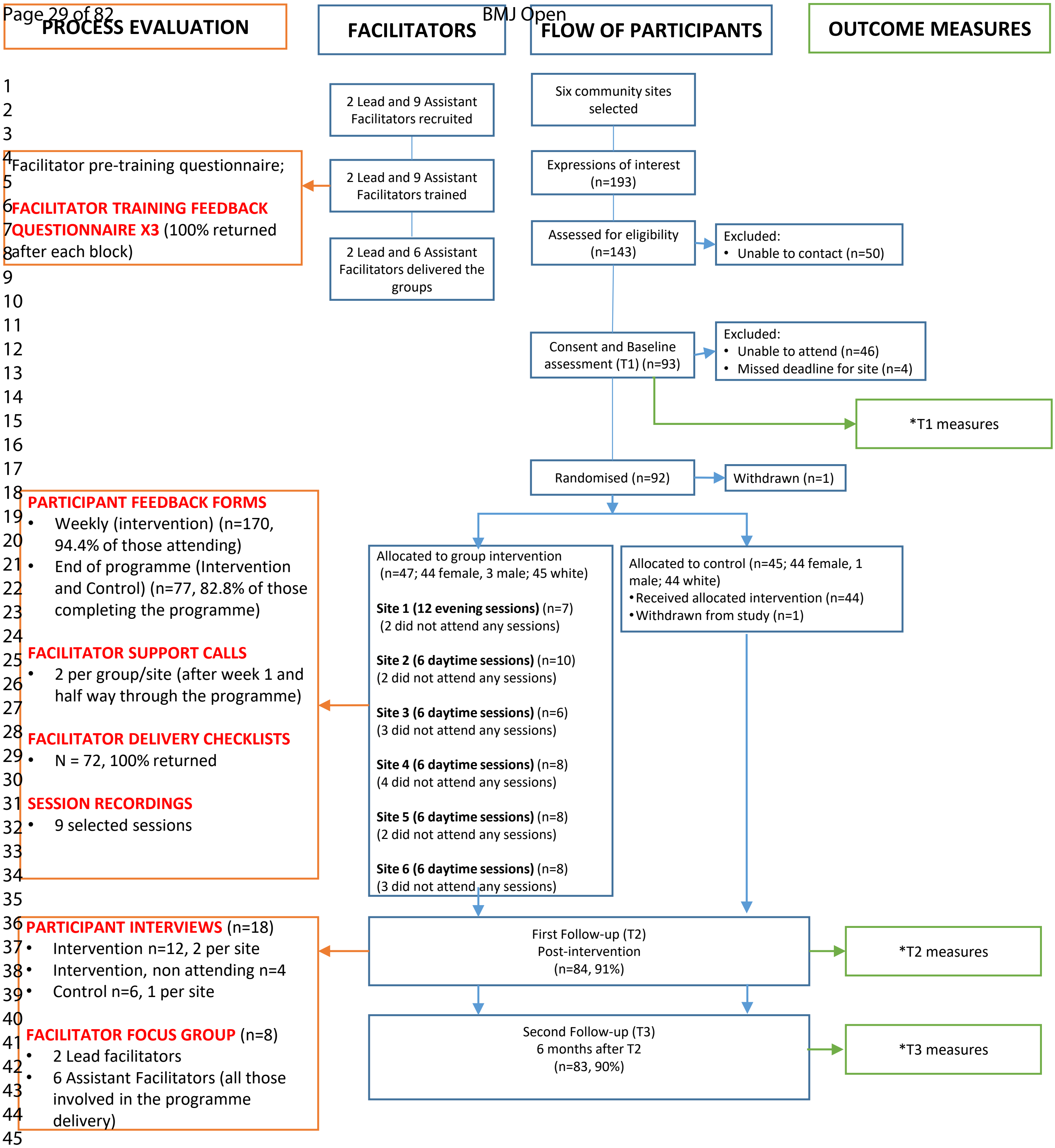

* Bjornstad G, Wilkinson K, Cuffe-Fuller B, et al. Healthy Parent Carers peer-led group-based health promotion intervention for parent carers of disabled children: protocol for a feasibility study using a parallel group randomised controlled trial design. Pilot and Feasibility Studies. 2019;5(1):1-13.

FIGURE 2 - Programme Refinement Process

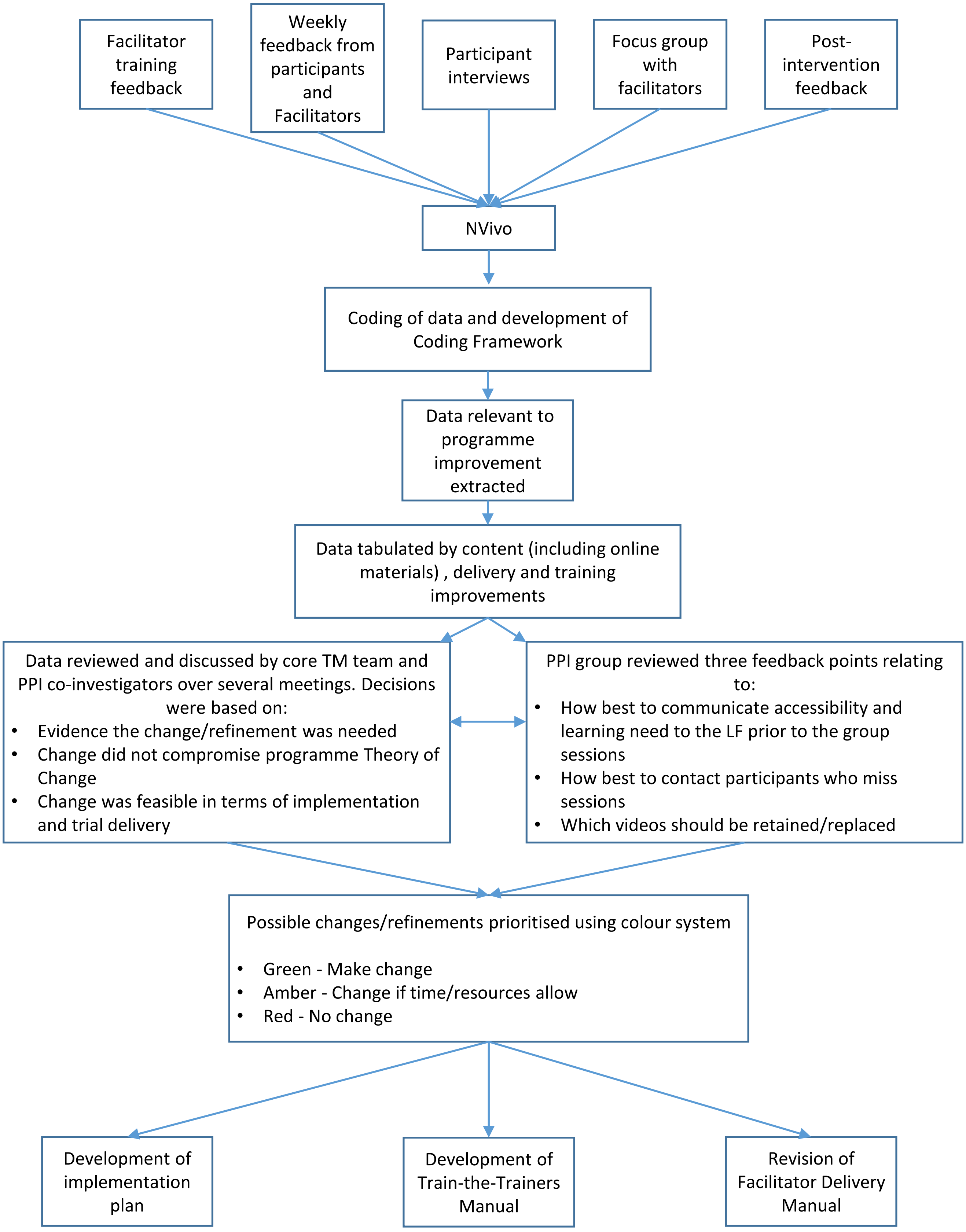

Supplementary Document 1a. Healthy Parent Carers intervention details

The details of the intervention are also reported in the intervention development paper (Borek et al., 2018) and the feasibility study protocol (Bjornstad et al., 2019). The results of the feasibility study are reported separately. The intervention details below are reported using the checklist for reporting of group-based interventions (Borek et al., 2015).

**INTERVENTION DESIGN**	
1. Intervention source or development methods	Developed based on an intervention mapping approach (Bartholomew et al., 2016), in partnership with parent carers. Details are described in Borek et al., 2018.
2. General setting	Community setting (with 6 sites/venues)
3. Venue characteristics	6 sites/venues, with different characteristics (2 special schools, 1 children's hospice, 1 Parent Carer Forum premises, 1 adult learning community venue, and 1 hotel regularly used for parent carer meetings).<br><br>All rooms set up to enable interaction between participants (i.e. facing each other).
4. Total number of group sessions	6 or 12 sessions
5. Length of group sessions	6 daytime sessions of 4 hours (2 modules per session)<br>or 12 evening sessions of 2 hours (1 module per session)
6. Frequency of group sessions	Weekly
7. Duration of the intervention	6 or 12 weeks
**INTERVENTION CONTENT**	
8. Change mechanisms or theories of change	The intervention logic model and intended mechanisms of change are reported in Borek et al., 2018. In brief, the intervention aimed to increase knowledge of health-related behaviours, skills in making health-related changes, improve attitudes towards change, increase self-efficacy, and provide social support to make changes, leading to behaviour change (based on CLANGERS) and psychological change (increased empowerment and resilience), leading to improved health and wellbeing.
9. Change techniques	• Provide/exchange information about behaviour-health link<br>• Provide/exchange instructions on practical strategies<br>• Prompt barrier identification and problem solving<br>• Prompt practice<br>• Prompt specific goal setting (for behaviours)<br>• Prompt use of prompts and rewards<br>• Prompt self-monitoring<br>• Prompt goal/progress review (behaviours)<br>• Provide general support and encouragement<br>• Provide opportunities for social comparisons
10. Session content	12 modules:<br>1. Introduction to health and wellbeing<br>2. Introduction to CLANGERS<br>3. Connect<br>4. Learn<br>5. be Active

For peer review only

	6. take Notice<br>7. Give<br>8. Eat well<br>9. Relax<br>10. Sleep well<br>11. Managing stress<br>12. Keeping healthy
11. Sequencing of sessions	Yes – sessions delivered sequentially (as above)
12. Participants' materials	In the sessions: goal setting and CLANGERS diary sheets given to participants, and other materials as parts of group activities.<br><br>Online Healthy Parent Carers materials (written information for each module, links to videos watched in the sessions)
13. Activities during the sessions	Each session started with a 'warm-up' activity and a review of the past week. This was followed by discussions focused on the topic of each module (e.g. 'what does it mean to you to relax? How do you relax? Why is relaxing important? What might stop you from, or help you, relax?). For each module there was a practical activity to help illustrate the key messages or the CLANGERS (e.g., colouring, 'a day in life of a parent carer', a mindful walk). For details, see Borek et al., 2018.
14. Methods for checking fidelity of delivery	Checklists were completed by the facilitators after each session; a sample of audio-recordings of group sessions were checked using the checklist independently (double-checking) by two researchers. Details are reported separately.
**PARTICIPANTS**	
15. Group composition	Parent carers, all female, except for two groups one of which had one male carer, the other of which had two. Ages ranged from 26-71 years (mean 42.5 years)
16. Methods for group allocation	Participants were recruited locally for each site (then randomised to the group intervention or control).
17. Continuity of participants' group membership	The same participants remained in the same group for the duration of the intervention.
18. Group size	7-10 participants were assigned per group. The attendance varied between sessions and groups (lowest attendance being 2 participants over 6 sessions). Details of attendance are reported separately.
**FACILITATORS**	
19. Number of facilitators	2 facilitators per group: one lead facilitator (LF) and one assistant facilitator (AF).<br><br>Overall 2 LF and 6 AF delivered the programme
20. Continuity of facilitators' group assignment	The same pairs of LF and AF facilitated the same groups.
21. Facilitators' professional background	LF: experienced facilitators of training for parent carers delivered through the Council for Disabled Children (e.g., the Expert Parent Programme).

For peer review only

	AF: three had some experience of facilitating parent carer groups and/or training.
22. Facilitators' personal characteristics	All facilitators were parent carers. Both LF were female and one of the six AFs was male.
23. Facilitators' training in intervention delivery	LF received 4 (2 x 2) days of training in delivering the programme; one block of 2 days of these were delivered together for lead and assistant facilitators, LFs and AFs also received one day of refresher training.
24. Facilitators' training in group facilitation	LF were trained and experienced in group facilitation as part of their facilitator/trainer roles for the Council of Disabled Children.<br><br>All facilitators received an overview of facilitating groups and managing group dynamics as part of the training in delivering the intervention.
25. Facilitators' materials	Facilitators delivered the sessions using a manual with instructions outlining all session activities.
26. Intended facilitation style	Participant-centred, interactive, and discussion-based (not didactic).

References:

Bartholomew Eldredge LK, Parcel GS, Kok G, Gottlieb NH, Fernandez ME. *Planning health promotion programs: an intervention mapping approach*. 4th ed. San Francisco: Jossey-Bass; 2016.

Bjornstad G, Wilkinson K, Cuffe-Fuller B, Fitzpatrick K, Borek A, Ukoumunne OC, Hawton A, Tarrant M, Berry V, Lloyd J, McDonald A. Healthy Parent Carers peer-led group-based health promotion intervention for parent carers of disabled children: protocol for a feasibility study using a parallel group randomised controlled trial design. *Pilot and Feasibility Studies*. 2019;5(1):1-3.

Borek AJ, Abraham C, Smith JR, Greaves CJ, Tarrant M. A checklist to improve reporting of group-based behaviour-change interventions. *BMC Public Health*. 2015;15(1):963.

Borek AJ, McDonald B, Fredlund M, Bjornstad G, Logan S, Morris C. Healthy Parent Carers programme: development and feasibility of a novel group-based health-promotion intervention. *BMC Public Health*. 2018;18(1):270.

For peer review only

TIDieR
Template for Intervention Description and Replication

The TIDieR (Template for Intervention Description and Replication) Checklist*:

Information to include when describing an intervention and the location of the information

Item number	Item	Where located ** Primary paper (page or appendix number)	Other † (details)
	**BRIEF NAME**		
**1.**	Provide the name or a phrase that describes the intervention.	___p4________	_______________
	**WHY**		
**2.**	Describe any rationale, theory, or goal of the elements essential to the intervention.	___p4________	______________
	**WHAT**		
**3.**	Materials: Describe any physical or informational materials used in the intervention, including those provided to participants or used in intervention delivery or in training of intervention providers. Provide information on where the materials can be accessed (e.g. online appendix, URL).	___p5________	Box 1________
**4.**	Procedures: Describe each of the procedures, activities, and/or processes used in the intervention, including any enabling or support activities.	___p6________	Supplementary document 1a parts 8-13
	**WHO PROVIDED**		
**5.**	For each category of intervention provider (e.g. psychologist, nursing assistant), describe their expertise, background and any specific training given.	___p5_______	Box 2, Supplementary document 1a parts 21-25
	**HOW**		
**6.**	Describe the modes of delivery (e.g. face-to-face or by some other mechanism, such as internet or telephone) of the intervention and whether it was provided individually or in a group.	___p5________	Box 1________

For peer review only

	**WHERE**		
**7.**	Describe the type(s) of location(s) where the intervention occurred, including any necessary infrastructure or relevant features.	___p5________	Supplementary document 1a, parts 2-3
	**WHEN and HOW MUCH**		
**8.**	Describe the number of times the intervention was delivered and over what period of time including the number of sessions, their schedule, and their duration, intensity or dose.	___p5_______	Supplementary document 1a, parts 4-7
	**TAILORING**		
**9.**	If the intervention was planned to be personalised, titrated or adapted, then describe what, why, when, and how.	___N/A_______	___________
	**MODIFICATIONS**		
**10.‡**	If the intervention was modified during the course of the study, describe the changes (what, why, when, and how).	___N/A_______	____________
	**HOW WELL**		
**11.**	Planned: If intervention adherence or fidelity was assessed, describe how and by whom, and if any strategies were used to maintain or improve fidelity, describe them.	___p7________	Supplementary document 4, Bjornstad et al. (under review)
**12.‡**	Actual: If intervention adherence or fidelity was assessed, describe the extent to which the intervention was delivered as planned.	___p7________	___________

** **Authors** - use N/A if an item is not applicable for the intervention being described. **Reviewers** – use '?' if information about the element is not reported/not sufficiently reported.

† If the information is not provided in the primary paper, give details of where this information is available. This may include locations such as a published protocol or other published papers (provide citation details) or a website (provide the URL).

‡ If completing the TIDieR checklist for a protocol, these items are not relevant to the protocol and cannot be described until the study is complete.

For peer review only - http://bmjopen.bmj.com/site/about/guidelines.xhtml

For peer review only

* We strongly recommend using this checklist in conjunction with the TIDieR guide (see *BMJ* 2014;348:g1687) which contains an explanation and elaboration for each item.

* The focus of TIDieR is on reporting details of the intervention elements (and where relevant, comparison elements) of a study. Other elements and methodological features of studies are covered by other reporting statements and checklists and have not been duplicated as part of the TIDieR checklist. When a **randomised trial** is being reported, the TIDieR checklist should be used in conjunction with the CONSORT statement (see www.consort-statement.org) as an extension of **Item 5 of the CONSORT 2010 Statement.** When a **clinical trial protocol** is being reported, the TIDieR checklist should be used in conjunction with the SPIRIT statement as an extension of **Item 11 of the SPIRIT 2013 Statement** (see www.spirit-statement.org). For alternate study designs, TIDieR can be used in conjunction with the appropriate checklist for that study design (see www.equator-network.org).

For peer review only

For peer review only - http://bmjopen.bmj.com/site/about/guidelines.xhtml

Bjornstad *et al. Pilot and Feasibility Studies* (2019) 5:137
https://doi.org/10.1186/s40814-019-0517-3

Pilot and Feasibility Studies

STUDY PROTOCOL **Open Access**

Healthy Parent Carers peer-led group-based health promotion intervention for parent carers of disabled children: protocol for a feasibility study using a parallel group randomised controlled trial design

Gretchen Bjornstad[1], Kath Wilkinson[1], Beth Cuffe-Fuller[1], Katharine Fitzpatrick[1], Aleksandra Borek[2], Obioha C. Ukoumunne[3], Annie Hawton[4], Mark Tarrant[3], Vashti Berry[3], Jenny Lloyd[3], Annabel McDonald[5], Mary Fredlund[5], Shelley Rhodes[6], Stuart Logan[1] and Christopher Morris[1*]

Abstract

Background: Parent carers of disabled children are at increased risk of mental and physical health problems. They often experience challenges to maintaining good health which have implications for their well-being and their ability to care for their children. In response to these needs, researchers and parent carers developed the Healthy Parent Carers (HPC) programme. It is a peer-led, group-based intervention that promotes behaviours associated with health and well-being. The aims of this trial are to assess the acceptability of the HPC programme and the feasibility of its delivery in the community and to assess the feasibility and acceptability of the design of the definitive trial to evaluate the programme's effectiveness and cost-effectiveness.

Methods: We will establish six research sites and train facilitators to deliver the manualised intervention. Parent carers of children with special educational needs and disabilities will be individually randomised, stratified by group delivery site, to either take part in a group programme and online resources (intervention) or to receive access to the online resources only (control). Measures of mental health; well-being; health-related quality of life; health behaviours; patient activation; protective factors such as resilience, social connections, and practical support; and use of health care, social care, and wider societal resources will be collected before randomisation (baseline), immediately post-intervention, and 6 months later. Recruitment of participants, adherence to the programme, and the dose received will be assessed. Group sessions will be audio-recorded to evaluate the fidelity of delivery and participant engagement. Participants' and facilitators' feedback on the programme content and delivery, their experience, and the acceptability of the outcome measures and trial design will be collected through feedback forms, interviews, and focus groups.

Discussion: This trial will assess whether the programme delivery and evaluative trial design are feasible, to inform whether to progress to a definitive randomised controlled trial to test the effectiveness and cost-effectiveness of the Healthy Parent Carers programme.

(Continued on next page)

* Correspondence: christopher.morris@exeter.ac.uk
[1]Peninsula Childhood Disability Research Unit (PenCRU) and National Institute for Health Research Applied Research Collaboration South West Peninsula (PenARC), University of Exeter Medical School, University of Exeter, St. Luke's Campus, Heavitree Road, Exeter EX1 2LU, UK
Full list of author information is available at the end of the article

© The Author(s). 2019 **Open Access** This article is distributed under the terms of the Creative Commons Attribution 4.0 International License (http://creativecommons.org/licenses/by/4.0/), which permits unrestricted use, distribution, and reproduction in any medium, provided you give appropriate credit to the original author(s) and the source, provide a link to the Creative Commons license, and indicate if changes were made. The Creative Commons Public Domain Dedication waiver (http://creativecommons.org/publicdomain/zero/1.0/) applies to the data made available in this article, unless otherwise stated.

For peer review only - http://bmjopen.bmj.com/site/about/guidelines.xhtml

(Continued from previous page)
Trial registration: ISRCTN, ISRCTN151144652, registered on 25 October 2018; ClinicalTrials.gov, NCT03705221, registered on 15 October 2018.

Keywords: Behaviour change, Well-being, Resilience, Peer support, Patient and public involvement, Disabled children, Parents, Carers

Background

There are an estimated 960,000 disabled children in the UK, which is 7.3% of the population of children aged 0–18 years [1]. Parent carers of disabled children commonly report higher levels of stress and depression [2–11] and poorer physical health [3, 4, 7, 8, 12–14] than parents of typically developing children. Population-based studies suggest these health problems persist and may worsen over time [15]. These problems have implications for their ability to care for their children.

Parent carers often find the demands of caregiving have a negative impact on their physical and emotional health. Nevertheless, it is important to acknowledge that not all parents of disabled children report that their child's difficulties negatively affect their psychological or physical health [5], and in fact, some report positive impacts [8, 16]. Indeed, some parent carers may perceive a high burden with looking after a child with a relatively 'mild' condition whereas others, whose child may have more severe disabilities, may not perceive caring as high a burden [4].

Some interventions target external factors, such as navigating healthcare services [17], while others target levels of stress [18, 19] or emotional and social support [20]. A systematic review of psychological therapies for parents of children with chronic illness suggested promising results in terms of improved parent mental health, particularly for problem-solving therapy [21]. No benefits were found for cognitive behavioural therapy or family therapy on parent outcomes; however, the quality of the evidence was low and analyses were limited by lack of data available to the reviewers. A systematic review of mindfulness interventions for parents of children with autism indicated potentially positive effects on parents' stress levels and psychological well-being, with studies reporting good attendance and retention in 8-week programmes [22]. There is growing evidence that groups can facilitate change processes beneficial to health and well-being [23, 24] by enabling the formation of strong psychological connections and/or social identification with other group members which can enhance engagement, and thus possibly increase the interventions' effectiveness [25, 26].

The idea for this research came directly from parent carers who had been involved in a study evaluating peer support for parent carers [20]. They wanted to extend the benefits of emotional support to specific strategies to improve health and well-being. Researchers and parent carers in the Peninsula Childhood Disability Research Unit (PenCRU) Family Faculty co-created the Healthy Parent Carers (HPC) programme [27].

Previously, we tested the principle and acceptability of a 6-week intervention programme with one group of seven parent carers, delivered by the intervention developers. The intervention was developed using Intervention Mapping [28] and extensive stakeholder engagement and is described in detail in a separate paper [29]. Participants had children with various conditions including autism, cerebral palsy, and acquired brain injury. Retention of participants in our preliminary study was high with all staying until the end of the 6-week programme and 2-month follow-up. Participants' and facilitators' feedback were positive, indicating the intervention was feasible to deliver and acceptable to, and valued by, participants. The intervention content and delivery methods were refined following feedback, and the manual was updated.

This feasibility trial will provide information that will be used to determine whether to progress to a definitive trial of the HPC programme, which would have the following objectives:

1) To determine whether the peer-led, group-based HPC programme is more effective at improving health and well-being compared to providing online information only
2) To estimate the costs of delivering the HPC programme, and the cost-effectiveness of the programme, versus the provision of online information
3) To understand how the Healthy Parent Carers intervention is working, for whom, and in what context to inform the implementation of the programme should it be shown to be effective

The current trial aims to assess the acceptability of the HPC programme and the feasibility of its delivery in the community, as well as the feasibility and acceptability of the design of the definitive trial in order to evaluate whether a fully powered randomised controlled trial is warranted and to determine the optimal trial design.

For peer review only - http://bmjopen.bmj.com/site/about/guidelines.xhtml

Methods

Objectives

This trial has two overarching aims:

1. To evaluate whether the programme can be delivered in the community by facilitators other than the developers, specifically to:
   (a) Assess the feasibility of establishing venues, and identifying and training group facilitators to be in a position to deliver the intervention
   (b) Assess the fidelity of intervention delivery in terms of format, content, and quality
   (c) Assess the experience and engagement of participants, facilitators, and trainers
   (d) Assess the programme attendance
2. To provide information necessary to design a definitive randomised controlled trial, specifically to:
   (a) Assess the feasibility of recruiting participants in different sites
   (b) Assess the acceptability of randomisation of parent carers
   (c) Assess the attrition and completion and proportion of any missing data in questionnaire measures
   (d) Appraise the performance of candidate health and well-being outcome measures in terms of acceptability to participants, and feasibility and interpretability for researchers
   (e) Estimate the variability (standard deviation) and the level of clustering within programme delivery groups in the intervention arm to help inform the sample size calculation for the definitive trial
   (f) Test the proposed cost-effectiveness framework for a future randomised trial

This will help inform whether to progress to a definitive randomised controlled trial to test the effectiveness and cost-effectiveness of the programme and provide information necessary to design the trial.

Design

A feasibility trial using a parallel group randomised controlled trial design will be carried out in six sites in the southwest of England. Participants will be randomly allocated to receive the group-based programme and access to online programme resources or to a control group receiving access to the online resources only. Data collection will take place at three time points in both trial arms at baseline (prior to randomisation), immediately post-intervention, and 6 months later. As the intervention can be delivered over 6 or 12 weeks, the post-intervention data collection time point will vary relative to randomisation but will be consistent in terms of the amount of time passing after completion of the intervention. Participants in the control arm in each randomised site will complete measures at the same time as participants in the intervention arm for that site. The two arms will therefore be balanced in terms of the timing of outcome measures. The trial design and the flow of participants through the trial are illustrated in the trial flow chart and SPIRIT figure (Fig. 1 and Table 1). The SPIRIT checklist is provided as an additional file (see Additional file 1).

Public involvement

This project has a strong ethos of parent carer and stakeholder engagement from inception. The public involvement in this project will ensure the following: (a) the research is conducted in an acceptable manner, (b) the research outputs are relevant and useful to parents of children with special educational needs and disabilities, and (c) our dissemination materials and methods are appropriate and accessible.

Over 40 parents of children with a range of conditions from the PenCRU Family Faculty public involvement group have participated in a study-specific working group since 2014. Our Stakeholder Advisory Group (SAG) includes representatives from the local authority, public health, parent carer forums, relevant charities, and special schools.

Parent carers have been involved in all stages of developing the intervention and designing the feasibility trial including:

a) Proposing the idea for the project based on their needs and experiences
b) Co-designing and refining the intervention and training content and delivery methods
c) Providing feedback on research methods including the selection of the comparison conditions
d) Advising on the content and form of the Resource Use Questionnaire for use as part of the cost-effectiveness framework
e) Contributing to interpreting and disseminating the findings of the previous study
f) Interviewing and hiring research staff
g) Discussing and advising on the design of the feasibility trial
h) Recommending responses to peer reviews when applying for funding.

Study setting and location

In collaboration with our SAG, we will identify six venues (e.g. schools, community centres, adult and community learning venues) where it is possible to establish and host a group. We will agree days, times, durations

For peer review only

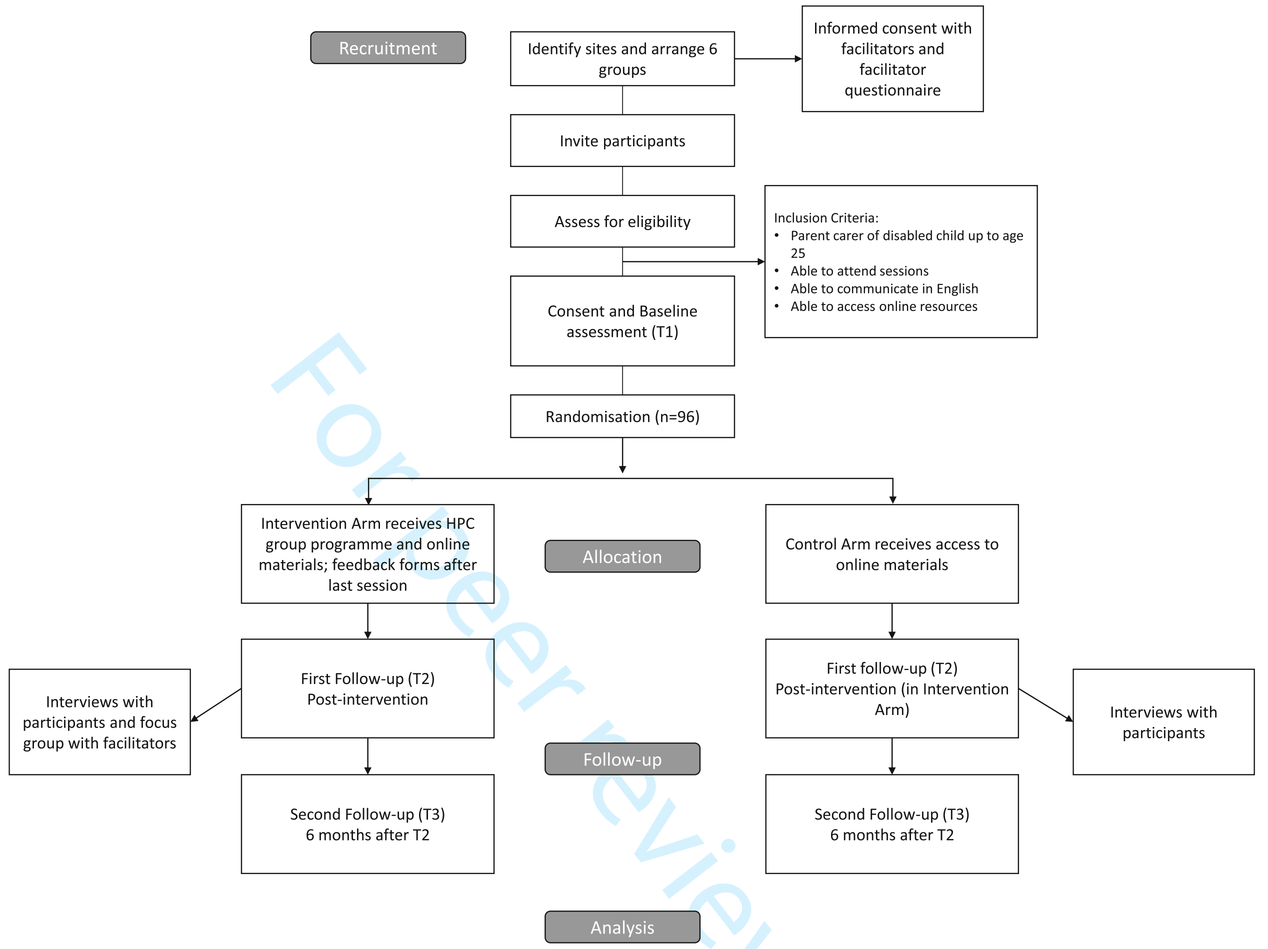

Fig. 1 Trial flow chart

and frequencies of sessions, and local named organisers for each venue.

Sample size

We aim to recruit 96 participants, to be allocated on 1:1 ratio to intervention and control. This is a large enough sample to estimate the percentage providing data at follow-up (assumed to be 80%—76 participants) with a margin of error of 10 percentage points based on the lower bound of the 95% confidence interval. Assuming that 38 participants are followed up in each trial arm, this will be large enough to estimate the standard deviation for continuous outcomes in each arm within 29% of its true value based on the upper bound of the 95% confidence interval. Finally, 76 participants at follow-up *are* large enough to estimate a correlation coefficient of 0.5 between baseline and follow-up scores for a continuous outcome with a margin of error of 0.19 based on the lower bound of the 95% confidence interval. We will randomise a minimum of *8* and *a* maximum of 24 participants at each of the 6 sites. This will mean that 4–12 participants will be allocated to each of the intervention and control trial arms at each site. We expect the ideal group size to be between 6 and 12 people but are allowing for potential attrition and variation in recruitment between sites.

Inclusion criteria

People meeting the inclusion criteria are (1) primary carers of children with additional needs and/or disabilities (participants who self-identify as primary carers are eligible; the child can be up to 25 years old consistent with the current Department of Health and Department of Education Special Educational Needs and Disability (SEND) legislation in England and The Children's Act; no named diagnosis is necessary, and we are not limiting to specific conditions), (2) willing and able to attend the programme group meeting session(s) on arranged dates/times, and (3) able to access online information.

For peer review only - http://bmjopen.bmj.com/site/about/guidelines.xhtml

Table 1 Schedule of enrolment, interventions, and assessments

**TIMEPOINT**	**Enrolment (T1)**			**Allocation**	**Post-allocation**	**Post-intervention (T2)**	**6 months post-intervention (T3)**
**ENROLMENT:**							
Eligibility screen	X						
Informed consent		X					
Allocation				X			
**INTERVENTIONS:**							
Group Healthy Parent Carers programme					—	—	
Online Healthy Parent Carers resources					—	—	
**ASSESSMENTS:**							
Demographics (15 items)			X				
About My Child -19 (19 items)			X				
Warwick-Edinburgh Mental Well-being Scale (WEMWBS) (14 items)			X			X	X
EuroQol-5 Dimensions (EQ-5D-5L) (5 items)			X			X	X
Patient Health Questionnaire-9 (PHQ-9) (9 items)			X			X	X
Health Promoting Activities Scale (HPAS) (8 items)			X			X	X
Patient Activation Measure (PAM) (13 items)			X			X	X
Parents' Assessment of Protective Factors (PAPF) (36 items) "Parent Statements"			X			X	X
ICEpop CAPability measure for Adults (ICECAP-A) (5 items)			X			X	X
Service and Resource Use questionnaire: Healthy Parent Carers (31 items)			X			X	X
Adverse event assessments					X	X	X
Compliance (attendance)					X	X	
Feedback Form (14 items)						X	
Interviews and focus groups						X	

For peer review only

Exclusion criterion

Potential participants who are not able to communicate in English are excluded. This is necessary because the programme has not yet been translated into other languages.

Recruitment

We will advertise the study in several ways. Press releases and interviews in local television and/or radio will be used to publicise the study. Members of our SAG will be asked to advertise the project to their members through their email lists and networks. We will also share study adverts and information via social media and through the PenCRU Family Faculty email list, asking *the* members to share in their networks. We will liaise with Information and Advice Services in each locality, staff in social services, and Special Educational Needs Coordinator *(*SENCOs*)* in schools in each study site to target potentially more isolated parent carers. We will also recruit participants at events for parent carers at the study venues and other venues in the southwest where interested parent carers can discuss the project with the research team. This recruitment strategy uses many different approaches because parent carers do not all access the same services and not all parent carers are connected with their local parent carer forums. We will ask all participants how they heard about the study during screening, and this information will be recorded to assess whether some recruitment methods may be more effective than others. However, we are mindful that while some methods may not result in large numbers of recruits, they may help us to reach parent carers who are more isolated and more in need of support and, as such, will be seen as important methods to take forward in a definitive trial.

Interested parent carers will contact the researchers. There will be *a* telephone or face-to-face screening to check *the* eligibility, understanding of the study, and to answer any questions. A researcher will meet each potential participant individually. Those who want to participate will sign a consent form and complete baseline questionnaires online using an electronic patient-reported outcome (ePRO) system with a researcher on hand for support as necessary [30]. Reasons for not consenting to participate will be recorded if provided by those who decline.

Allocation to trial arms

When recruitment is completed at each site, we will proceed to randomisation. Each of the six programme groups will constitute a study site, with participants who choose that group being randomised to either attend the group or receive the online resources only. A computer-generated randomisation sequence will be used to assign the participants in each site to the intervention and control arms. A block randomisation scheme will be implemented to ensure balance in the number of participants allocated to each trial arm, stratified by group delivery site. The allocation sequence will be concealed from researchers using an online central randomisation service setup and maintained by the Exeter Clinical Trials Unit (UKCRC Registration ID 65). Blinding will not be used in this trial.

All participants will receive an email and letter indicating the result of randomisation. The participants randomly allocated to the intervention arm will be sent details of the group sessions and be contacted by their lead facilitator before the first group session. Participants in both arms will receive a link to the online programme resources and instructions on the web page. We will monitor the number of participants who refuse participation and record their reasoning (if they wish to share it) to gauge the acceptability of our trial design.

Intervention

The group-based programme was developed using Intervention Mapping approach [28]. Full details of the intervention, including its development, logic model, and content (e.g. activities, behaviour change techniques), are available in a previous publication [29]. In brief, the programme aims to expand parent carers' social networks and provide social support from peers with a shared sense of social identity alongside targeted activities to improve parent carers' confidence, motivation, self-efficacy, and empowerment, thus creating the conditions for change necessary for them to feel able to make their own plan to prioritise healthy behaviours for themselves.

The programme content is based around a set of universal and evidence-based actions (called CLANGERS) associated with health and well-being. CLANGERS stands for Connect, Learn, be Active, Notice, Give, Eat well, Relax and Sleep [31]. The 'CLANG' component comprises the 'Five Ways to Wellbeing' based on the evidence from the foresight project on Mental Capital and Wellbeing [32]. Each of these behaviours is potentially more difficult for parent carers.

The programme content is organised into 12 modules lasting 2 h each. The modules can be delivered weekly over 6 sessions (comprising 2 modules per session) or 12 sessions (1 module per session). Our Family Faculty PPI working group suggested that offering either 6 longer sessions in the daytime or 12 shorter evening sessions would be reasonable for most parent carers. One or both options will be offered per area in order to maximise recruitment and to reflect likely real-world delivery in community settings, with 6 groups being delivered in the study in total. If uptake is very low for a particular

For peer review only - http://bmjopen.bmj.com/site/about/guidelines.xhtml

site, the delivery model may be adapted during recruitment to increase numbers.

Facilitators

The lead facilitators of the group-based programme will be experienced facilitators of the 'Expert Parent Programme' courses created by the Council for Disabled Children (CDC) with funding from the Department of Health. The facilitators are parent carers. CDC has a selection process, trains, and provides supervision for their facilitators to ensure that they facilitate the groups effectively. Their nationwide network of over 70 facilitators provides a sustainable model for the implementation of the programme in the future. Facilitators will use the Healthy Parent Carers Facilitator Manual that includes module outlines, content, timings, activities, and resources needed. The Facilitator Manual also includes safeguarding procedures for the facilitator to follow in case of any adverse events such as suicidal ideation or disclosure of safeguarding issues.

There will also be an assistant facilitator in each group to assist the facilitator in sessions. We will recruit a local parent carer for this role using a person specification detailing the required personal qualities and skills and a selection process.

We will provide training for lead and assistant facilitators. The training will take place over 4 days for lead facilitators and 2 days for assistant facilitators; it will be delivered by researchers and the parent carer co-investigators who co-developed the programme and facilitated the group in our previous study. Lead facilitators will receive ongoing supervision and support through the CDC and support from the research team. Conference calls with facilitators, assistants, and researchers will be convened to reflect on delivery of the sessions. These discussions will inform intervention design and training needs and provide a forum to share ideas and ways to address any challenges arising.

The Healthy Parent Carers online resources are part of the intervention. They reflect the content of the group sessions, provide space to write down reflections, and prompts to set specific goals and for self-monitoring of CLANGERS-related behaviours.

If the participants in the group programme miss a session, they will be telephoned by the facilitator, who will summarise the session and encourage the participant to reflect on their week, read the section of the HPC online resources, and set their weekly goals.

Control

Participants in the control arm will receive access to the HPC programme resources online with instructions. Risk of contamination between participants allocated to each arm is low because the intervention is predicated on participants developing a shared social identity as members of the HPC group. Participants in both arms will be asked whether they have had contact with participants in the other arm of the trial as part of a post-intervention feedback form.

Data collection

Study records

We will record the data on the feasibility of recruitment, including how many people respond to the adverts, how they heard about the study, reasons for not taking part for those who decline, and how many are successfully recruited. We will record delivery setting, delivery model, attendance, attrition, and reasons for missed sessions or withdrawal from the study. We will also monitor how long it takes to accrue the target number of participants at each site and at what point in the trial process any participants withdraw.

Sample characteristics

Demographic data will include gender, ethnicity, parent relationship status, number of children, employment status, level of education, income, housing status, and age, gender, and diagnosis (if any) of their disabled child. We will also collect information about their disabled child's functioning and health complexity using the About my Child measure (AMC-19) [33]. We will also use participants' postcodes to link with the Indices of Multiple Deprivation as an indicator of deprivation relative to England and Wales in the area where participants live [34].

Outcome measures

Participants will be asked to complete all measures before randomisation, immediately post-intervention, and 6 months post-intervention, regardless of attendance or engagement with the interventions. Based on the recommendations from our Family Faculty PPI working group, the measures will be available to complete online, using a computer, smartphone, or tablets. The Exeter Clinical Trials Unit will set up an online platform for participants to access and complete the measures. Participants may request to complete the measures on paper if they wish. Any measures completed on paper will be independently double-entered by two researchers. These requests will be monitored to track preferences for online- versus paper-based measures.

Two members of our Family Faculty PPI working group have tested the applicability and time to complete the measures (45 min). A £25 shopping voucher will be posted to participants as acknowledgement for completing measures at each time point.

The measures will comprise:

For peer review only

a) Warwick-Edinburgh Mental Well-being Scale (WEMWBS): The WEMWBS is a 14-item scale used to assess the mental well-being in the general population and in the evaluation of programmes aiming to improve mental well-being [35]. Responses are normally distributed in the general population. WEMWBS has been validated in the UK, Europe, and elsewhere. It has been tested with minority ethnic populations, users of mental health services, and carers. It is sensitive to changes occurring through participation in programmes that promote well-being such as health promotion programmes. A tariff of well-being-adjusted life-year weights is currently being developed for responses on the WEMWBS, which will enable the measure to be used in cost-effectiveness analyses recognised by the National Institute for Health and Care Excellence (NICE) [36].
b) Patient Health Questionnaire-9 (PHQ-9): The PHQ-9 is a 9-item measure that rates the frequency of symptoms and is designed for screening, diagnosing, monitoring, and measuring the severity of depression [37–39]. Categories based on the cutoff scores of 5, 10, 15, and 20 represent none, mild, moderate, moderately severe, and severe depression, respectively. As part of a safeguarding protocol, we will use the Patient Health Questionnaire (PHQ-9) to measure depressive symptoms. The PHQ-9 questionnaire is recommended by NICE to assess depression in adults [40], and its use is highlighted in clinical pathways [41], so the interpretation of scores is widely understood by GPs and primary care staff. There is good evidence, across a range of studies, for the validity, reliability, sensitivity, and specificity of the PHQ-9 for detecting depressive disorders [38, 39]. It can be administered repeatedly to assess change in depression in response to treatment. Question 9 screens for suicidal ideation. If the person scores higher than 0 on question 9, or at any other point discloses suicidal ideation, we will follow the safeguarding protocol.
c) EuroQol 5 Dimensions (EQ-5D-5 L): The EQ-5D-5 L is a measure of health-related quality of life. It consists of five items measuring five dimensions of health-related quality of life (mobility, self-care, usual activities, pain/discomfort, and anxiety/depression) and a vertical visual analogue scale measuring self-rated health [42]. QALY weights can be applied to EQ-5D scores, which can then be used to calculate QALYs, and the cost-per-QALY of the intervention in a future definitive trial. The EQ-5D is NICE's preferred measure for use in health technology cost-effectiveness analyses.
d) Parents' Assessment of Protective Factors (PAPF): The PAPF is a 36-item measure that assesses protective factors identified in the development of the Strengthening Families evidence-based parenting programme [43]. These protective factors are as follows: parental resilience, social connections, concrete support in times of need, and support of children's social and emotional competence. These factors relate to the determinants of change in the logic model for the Healthy Parent Carers Programme.
e) Health Promoting Activities Scale (HPAS): The HPAS is an 8-item measure of a person's estimation of the frequency with which they participate in a range of activities that promote or maintain health and well-being [44, 45]. It was developed for and validated with mothers of children with disabilities.
f) Patient Activation Measure (PAM): The PAM is a 13-item measure that measures the spectrum of skills, knowledge, and confidence in patients and captures the extent to which people feel engaged and confident in managing their own health and care [46, 47]. It has been tested with 100,000 patients with long-term conditions in England to establish the feasibility of using the PAM across the NHS, how activation can inform support for self-management, what support clinicians and commissioners need to use the measure effectively, and whether supporting activation can improve outcomes for patients in the NHS.
g) ICEpop CAPability measure for Adults (ICECAP-A): The ICECAP-A is a 5-item measure of capability, which includes the following aspects of well-being found to be important to adults in the UK: attachment, stability, achievement, enjoyment, and autonomy [48–50]. A set of UK well-being adjusted life-year weights are available for the ICECAP-A, enabling it to be used in economic evaluations.
h) Resource use questionnaire: We developed a study-specific resource use questionnaire in collaboration with parent carers. This includes health, social care, participant, and broader societal resource use, and draws on measures in the Database of Instruments for Resource Use Measurement (DIRUM) [51].

Process evaluation

In line with the MRC guidance on process evaluations, this study will include a process evaluation that is appropriate for the feasibility testing stage of the development-evaluation-implementation process for this intervention [52]. This process evaluation will allow for the exploration of the feasibility of implementation of the intervention by assessing uptake (recruitment) and retention, participant

For peer review only - http://bmjopen.bmj.com/site/about/guidelines.xhtml

engagement, fidelity of delivery (to content and quality), experiences of participants and facilitators, unintended consequences, and contextual factors which may influence experience and delivery.

The following data collection tools will be used to assess fidelity of intervention delivery, participant and trainer characteristics/motivations, trainer knowledge and self-efficacy to deliver the programme, participant engagement with the programme/online materials, and acceptability of the intervention and trial design.

a) Facilitator pre-training questionnaire: We will use a pre-training questionnaire to collect information about facilitators' characteristics, their motivations to take part, relevant background and experience, and expectations of delivering the programme.
b) Facilitator training feedback: Following delivery of the training, we will use a questionnaire to gather the facilitators' feedback about their self-reported knowledge, understanding, skills, and confidence to deliver the intervention and to gather their reflections on the training.
c) Facilitator delivery observations: We will use a checklist to assess lead facilitators' competence to deliver while observing their delivery of the programme content during the facilitator training. The checklist includes key skills and competencies linking to the objectives of the lead facilitator training and will enable trainers and research staff to assess facilitators' readiness to deliver the programme. This will also help to guide and plan additional or future training.
d) Facilitator checklist, records, and support calls: We will use a self-report checklist completed jointly by the facilitators to indicate which content they have covered in each session (adherence), the duration of the sessions (dose), and the participants' engagement. Facilitators will be asked to record attendance at each session. We will also arrange support calls with facilitators to gather more information about how the groups are going and any challenges to delivery.
e) Session recordings: We will audio-record group sessions and will sample two to three recordings from each group to assess fidelity to intervention content, quality of delivery, and participant engagement. A researcher will rate the delivery using the same checklist used by the facilitators after each session. A second researcher will rate one recording per group ($n = 6$, 14%). The two researchers will compare the scoring of the first three groups immediately, and any inconsistencies will be discussed with JL/MT, to ensure there is a clear understanding of the assessment criteria. The scores of the double-coded sessions will be agreed between researchers, and the sessions assessed by the researchers will be compared with the facilitators' scores.
f) Participants' feedback: We will collect feedback from treatment and control arm participants about the programme content and delivery, their experiences, and whether they had contact with participants in the other arm of the trial via feedback forms at the end of each group session for those in the intervention arm and at the end of the programme for those in both trial arms.
g) Participant interviews: We will sample purposively 12 participants (from different groups) in the intervention arm and 6 participants allocated to the control arm across all sites for semi-structured telephone interviews. For the intervention arm, these interviews will explore participants' experiences of, and engagement in, the programme and the group and their views on the group content, activities, and facilitators. All, control and intervention groups, participants will be asked in the interviews about engagement with online resources, perceived impact of the programme and any potential contextual influences, and acceptability of the trial processes and measures. We will also sample up to 4 participants (from different groups) who were allocated to the intervention arm of the trial but did not attend any group sessions to ask them about barriers to attending and whether anything could be done to promote attendance in future groups. All interviews with participants will last approximately 30 min and will be audio-recorded and transcribed verbatim, with names and other personal identifying information changed to protect confidentiality. Interviews will take place as soon as possible after the participants have completed their post-intervention measures and before they complete their 6-month follow-up measures.
h) Focus groups with facilitators: We will invite all lead facilitators and assistant facilitators to a focus group after the end of all groups. The focus group (lasting approximately 2 h) will cover facilitators' experiences of delivering, and engagement with, the programme, views on the programme content, activities and feasibility of programme delivery, facilitator training and skills, group management, and suggestions for improvements. The focus group will be audio-recorded and transcribed verbatim (with any potentially identifiable information anonymised).

For peer review only

Cost-effectiveness framework

We will develop and test a framework for assessing the cost-effectiveness of the intervention in a future randomised trial. We will:

a) Establish methods for estimating intervention resource use and costs (e.g. training of facilitators, facilitators' time, venue hire), in collaboration with the programme facilitators and site representatives
b) Develop a resource use questionnaire in collaboration with parent carers, drawing on measures in the Database of Instruments for Resource Use Measurement (DIRUM) repository [51]
c) Assess the acceptability to parent carers of the EQ-5D-5 L, the ICECAP-A, and the WEMWBS, judged by missing data and measurement properties [53].

Data analysis

Statistical/quantitative analysis

We will report the number of eligible people who self-refer and the percentage (with 95% confidence intervals (CIs)) of these that are randomised in the trial. These findings will also be reported separately for each type of delivery setting and delivery model to assess whether particular delivery settings or models are more popular and therefore would lead to higher recruitment rates in the subsequent definitive trial. We will also report numbers and percentages of people who heard about the study via different sources, organised into categories.

We will also report the percentage (with 95% confidence intervals) of participants who complete each assessment at each time point as an assessment of the acceptability of the measures and of the feasibility of collecting sufficient data in a definitive trial. We will summarise the characteristics of recruited participants using demographic data to allow for assessment of the representativeness of the sample relative to figures available from the Office for National Statistics on the population in the southwest of England. We will also report the baseline comparability of the trial arms with respect to demographics and outcome measures.

For the intervention arm, we will report the number and percentage of participants that attend each group session with 95% confidence intervals. The percentage of participants that are lost to follow-up at each follow-up point will be reported for each trial arm. Select characteristics (parent carer gender and age, child gender and age, Index of Multiple Deprivation quintile, study site (centre), and baseline scores on outcomes) will be compared between those who are and are not lost to follow-up within each trial arm using descriptive summaries, but no formal statistical tests. Acceptability, judged by missing data of EQ-5D-5 L and ICECAP-A, will help to plan methods for estimation of cost-effectiveness in the future trial.

Means, standard deviations, and the correlation between baseline and follow-up scores on continuous outcomes will be reported to inform the sample size calculation for the subsequent definitive trial.

Level of clustering within groups in the intervention arm will be quantified using the intra-cluster (intra-group) correlation coefficient to inform the sample size calculation for the definitive trial; however, we recognise the relatively small sample size for this purpose, and it will be used alongside information about levels of clustering in published studies of trials of similar group-based interventions in similar settings.

We will compare the outcomes at follow-up between the two trial arms based on the intention-to-treat principle with participants analysed according to the trial arm they were randomised to. Missing data will not be imputed. We will report only confidence intervals for the intervention effect and no p values, in line with the extension to the CONSORT statement for reporting randomised pilot and feasibility studies [54].

Analysis of process data

Descriptive statistics will be reported for the quantitative data collected in delivery observation checklists, facilitator checklists, checklists used to assess intervention session recordings, and participant feedback forms.

Qualitative data collected from feedback forms, interviews, and focus groups will be analysed thematically to provide insights into participants' experiences of the programme, intervention acceptability, and suggestions for improvement, and to enhance understanding of the impact of the intervention and the mechanisms of change in relation to the programme logic model [54]. Data will be analysed using inductive thematic analysis [55] separately for each data source (i.e. feedback comments, interviews, and focus groups), following the same approach. Some issues will emerge as more salient than others and the interpretation of findings will be influenced by the original research objectives as well as the themes emerging directly from the data.

NVivo software (version 12 Pro for Windows, QSR International) will be used to organise and analyse the qualitative data. Initially, two researchers will independently read and code line-by-line a sample of the data and discuss their coding to develop and agree on the coding framework. New codes may be added as the coding proceeds, and the codes and coded data will be reviewed. Codes will be defined, compared to each other, and organised into categories and themes. Attention will be paid to negative, or 'deviant', cases to inform developing themes and interpretation. Short summaries of each interview will be also written to explore how individual

For peer review only - http://bmjopen.bmj.com/site/about/guidelines.xhtml

experiences and views of the intervention may differ between participants. The analysis and interpretation of the data will be regularly discussed with the research team. A detailed record will be kept of the analysis process, including definitions of the themes and concepts and their application.

Discussion

We will interpret the findings of this feasibility trial and report the implications for progression to a definitive randomised controlled trial of the HPC programme. This will include any necessary amendments to the intervention content and delivery, as well as the development of a train-the-trainer manual to be used in training future facilitators of the programme. The following indicators of feasibility will be used to determine whether a definitive randomised controlled trial is feasible with the current trial design and procedures:

a) Recruit a minimum of 48 participants, which is the minimum number that will enable all six sites to be randomised and the intervention to be tested
b) Deliver 6 groups in total for the intervention arm, assessed by establishing 6 venues, and identifying and training facilitators, and groups completing the programme curriculum
c) At least 80% of participants completing measures at 6-month follow-up or a clear plan to achieve this in the trial

If any of these indicators are not met, the research team will consider whether a definitive trial may not be feasible or whether changes to the design or procedures and further feasibility testing are needed. The need to translate programme materials into other languages will also be taken into account for a subsequent trial.

A complete and transparent report of the trial will be produced with reference to recommendations of the CONSORT 2010 statement: extension to randomised pilot and feasibility trials, including a CONSORT participant flow chart [54]. The report will be written for publication in a peer-reviewed, open access, academic journal with authorship eligibility determined by following the International Committee of Medical Journal Editors recommendations [56]. A plain language summary of the findings will also be co-produced with members of our Family Faculty public involvement group and sent to trial participants and organisations that help to recruit participants and host the groups. We will consult our Family Faculty and Stakeholder Advisory Group for advice on ways to disseminate the findings.

NHS England's Commitment to Carers states 'Helping carers to provide better care and to stay well themselves will contribute to better lives for those needing care and more effective use of NHS resources' [57]. However, there is currently a paucity of interventions that promote health for parent carers. This feasibility trial and a subsequent definitive trial may have important implications for a public health strategy for parent carers of children with disabilities in the UK. It will also inform research and public health policy internationally, as the higher risk of psychological and physical health problems in parent carers is not limited to the UK.

Project timetable and milestones

The main milestones are as follows. Ethical approval for the study was received on 20 August 2018. The trial was registered on 25 October 2018 (ISRCTN 15144652). Recruitment of participants began on 29 October 2018. The analysis of data on fidelity and process evaluation will be conducted following data collection (summer to autumn 2019). The analyses of outcome measures will be conducted in February 2020. The expected date of completion is June 2020.

Supplementary information

Supplementary information accompanies this paper at https://doi.org/10.1186/s40814-019-0517-3.

Additional file 1. SPIRIT 2013 Checklist: Recommended items to address in a clinical trial protocol and related documents*.

Abbreviations

AMC-19: About my Child measure; CDC: Council for Disabled Children; CLANGERS: Connect, Learn, be Active, Notice, Give, Eat well, Relax, and Sleep; CONSORT: Consolidated Standards of Reporting Trials; DIRUM: Database of Instruments for Resource Use Management; EQ-5D-5 L: EuroQol 5 Dimensions; HPAS: Health Promoting Activities Scale; HPC: Healthy Parent Carers; ICECAP-A: ICEpop CAPability measure for Adults; NIHR: National Institute for Health Research; PAM: Patient Activation Measure; PAPF: Parents' Assessment of Protective Factors; PenCLAHRC: The National Institute for Health Research (NIHR) Collaboration for Leadership in Applied Health Research and Care (CLAHRC) South West Peninsula; PenCRU: Peninsula Cerebra Research Unit; PHQ-9: Patient Health Questionnaire-9; PPI: Patient and public involvement; SAG: Stakeholder Advisory Group; SEND: Special educational needs and disability; WEMWBS: Warwick-Edinburgh Mental Well-being Scale

Acknowledgements

We thank members of the PenCRU Family Faculty and the Stakeholders Advisory Group for their involvement in the development of the intervention and the design of this study.

Study Sponsor

University of Exeter, Research Ethics and Governance Office, University of Exeter, Lafrowda House, St Germans Road, Exeter, Devon, EX4 6TL

Authors' contributions

All authors contributed. CM led the development and preliminary evaluation of the programme and leads the study and public involvement work. GB and CM drafted the initial study design. AB, AM, MF, and CM designed the original programme. OU designed the quantitative analysis plan. AH designed the methods to develop and test a cost-effectiveness framework. AB, CM, VB, JL, MT, KW, and BCF designed the fidelity and process evaluation. KF facilitated public involvement. SR coordinated the CTU involvement in the design and conduct of the project. SL facilitated infrastructure support through National Institute for Health Research Applied Research

For peer review only

Collaboration South West Peninsula. All authors contributed to drafting and refining the study protocol and approved the final manuscript. The study Sponsor is the University of Exeter.

Funding
This paper presents independent research funded by the National Institute for Health Research (NIHR) under its Research for Patient Benefit (RfPB) Programme (Grant Reference Number PB-PG-0317-20044). We also acknowledge support from the National Institute for Health Research Applied Research Collaboration South West Peninsula. The views and opinions expressed in this paper are those of the authors and not necessarily those of the National Institute for Health Research or the Department of Health and Social Care. Delivery of the Healthy Parent Carers programme is funded by Public Health England, Kernow Clinical Commissioning Group, Northern, Eastern and Western Devon Clinical Commissioning Group, and a grant from the National Lottery Community Fund through the Reaching Communities programme. Peer review of the study design was provided during the NIHR funding application process. The funders have had no involvement in the writing of the manuscript and will not have any role during its execution, analyses, interpretation of the data, writing of the report, or the decision to submit the report for publication.

Availability of data and materials
Not applicable

Ethics approval and consent to participate
Ethics approval for the study was received on 20 August 2018 from the University of Exeter Medical School Research Ethics Committee in the College of Medicine and Health (UEMS REC 18/06/174); recruitment is currently ongoing.

Consent for publication
Not applicable

Competing interests
The authors declare that they have no competing interests.

Author details
[1]Peninsula Childhood Disability Research Unit (PenCRU) and National Institute for Health Research Applied Research Collaboration South West Peninsula (PenARC), University of Exeter Medical School, University of Exeter, St. Luke's Campus, Heavitree Road, Exeter EX1 2LU, UK. [2]Nuffield Department of Primary Care Health Sciences, Medical Sciences Division, Radcliffe Observatory Quarter, University of Oxford, Woodstock Road, Oxford OX2 6GG, UK. [3]National Institute for Health Research Applied Research Collaboration South West Peninsula (PenARC), University of Exeter Medical School, University of Exeter, St. Luke's Campus, Heavitree Road, Exeter EX1 2LU, UK. [4]Health Economics Group, University of Exeter Medical School, University of Exeter, St. Luke's Campus, Heavitree Road, Exeter EX1 2LU, UK. [5]PenCRU Family Faculty, University of Exeter Medical School, University of Exeter, St. Luke's Campus, Heavitree Road, Exeter EX1 2LU, UK. [6]Exeter Clinical Trials Unit, University of Exeter Medical School, University of Exeter, St. Luke's Campus, Heavitree Road, Exeter EX1 2LU, UK.

Received: 2 April 2019 Accepted: 15 October 2019
Published online: 23 November 2019

References
1. Blackburn CM, Spencer NJ, Read JM. Prevalence of childhood disability and the characteristics and circumstances of disabled children in the UK: secondary analysis of the Family Resources Survey. BMC Pediatr. 2010;10(21):1471–2431.
2. Barlow J, Cullen-Powell L, Cheshire A. Psychological well-being among mothers of children with cerebral palsy. Early Child Dev Care. 2006;176(3–4):421–8.
3. Brehaut JC, Kohen DE, Raina P, Walter SD, Russell DJ, Swinton M, O'Donnell M, Rosenbaum P. The health of primary caregivers of children with cerebral palsy: how does it compare with that of other Canadian caregivers? Pediatrics. 2004;114(2):e182–91.
4. Brehaut JC, Kohen DE, Garner RE, Miller AR, Lach LM, Klassen AF, Rosenbaum PL. Health among caregivers of children with health problems: findings from a Canadian population-based study. Am J Public Health. 2009;99(7):1254–62.
5. Emerson E. Mothers of children and adolescents with intellectual disability: social and economic situation, mental health status, and the self-assessed social and psychological impact of the child's difficulties. J Intellect Disabil Res. 2003;47(4–5):385–99.
6. Gallagher S, Phillips AC, Oliver C, Carroll D. Predictors of psychological morbidity in parents of children with intellectual disabilities. J Pediatr Psychol. 2008;33(10):1129–36.
7. Lach LM, Kohen DE, Garner RE, Brehaut JC, Miller AR, Klassen AF, Rosenbaum PL. The health and psychosocial functioning of caregivers of children with neurodevelopmental disorders. Disabil Rehabil. 2009;31(9):741–52.
8. Murphy NA, Christian B, Caplin DA, Young PC. The health of caregivers for children with disabilities: caregiver perspectives. Child Care Health Dev. 2007;33(2):180–7.
9. Oelofsen N, Richardson P. Sense of coherence and parenting stress in mothers and fathers of preschool children with developmental disability. J Intellect Dev Disabil. 2006;31(1):1–12.
10. Olsson MB, Hwang C. Depression in mothers and fathers of children with intellectual disability. J Intellect Disabil Res. 2001;45(6):535–43.
11. Singer GH, Floyd F. Meta-analysis of comparative studies of depression in mothers of children with and without developmental disabilities. Am J Ment Retard. 2006;111(3):155–69.
12. Tong HC, Haig AJ, Nelson VS, Yamakawa KS-J, Kandala G, Shin KY. Low back pain in adult female caregivers of children with physical disabilities. Arch Pediatr Adolesc Med. 2003;157(11):1128–33.
13. Lee M, Park C, Matthews AK, Hsieh K. Differences in physical health, and health behaviors between family caregivers of children with and without disabilities. Disabil Health J. 2017;10:565–70.
14. Arim RG, Miller AR, Kohen DE, Guèvremont A, Lach LM, Brehaut JC. Changes in the health of mothers of children with neurodevelopmental disabilities: an administrative data study. Res Dev Disabil. 2019;86:76–86.
15. Brehaut JC, Garner RE, Miller AR, Lach LM, Klassen AF, Rosenbaum PL, Kohen DE. Changes over time in the health of caregivers of children with health problems: growth-curve findings from a 10-year Canadian population-based study. Am J Public Health. 2011;101(12):2308–16.
16. Resch JA, Mireles G, Benz MR, Grenwelge C, Peterson R, Zhang D. Giving parents a voice: a qualitative study of the challenges experienced by parents of children with disabilities. Rehabil Psychol. 2010;55(2):139–50.
17. Council for Disabled Children. Expert Parent Programme. https://councilfordisabledchildren.org.uk/our-work/whole-child/practice/expert-parent-programme. Accessed 21 Jan 2019.
18. Singer GH, Ethridge BL, Aldana SI. Primary and secondary effects of parenting and stress management interventions for parents of children with developmental disabilities: a meta-analysis. Dev Disabil Res Rev. 2007;13(4):357–69.
19. Dykens EM, Fisher MH, Taylor JL, Lambert W, Miodrag N. Reducing distress in mothers of children with autism and other disabilities: a randomized trial. Pediatrics. 2014;134(2):e454–63.
20. Shilling V, Morris C, Thompson-Coon J, Ukoumunne O, Rogers M, Logan S. Peer support for parents of children with chronic disabling conditions: a systematic review of quantitative and qualitative studies. Dev Med Child Neurol. 2013;55(7):602–9.
21. Law E, Fisher E, Eccleston C, Palermo TM. Psychological interventions for parents of children and adolescents with chronic illness. Cochrane Database of Systematic Reviews 2019, Issue 3. Art. No.: CD009660. https://doi.org/10.1002/14651858.CD009660.pub4.
22. Cachia RL, Anderson A, Moore DW. Mindfulness, stress and well-being in parents of children with autism spectrum disorder: a systematic review. J Child Fam Stud. 2016;25(1):1–14.
23. Jetten J, Haslam C, Alexander SH. The social cure: identity, health and well-being: Psychology press; 2012.
24. Borek A, Abraham C, Greaves C, Gillison F, Tarrant M, Morgan-Trimmer S, McCabe R, Smith J: Identifying change processes in group-based health behaviour-change interventions: Development of the Mechanisms of Action in Group-based Interventions (MAGI) framework. Health Psychol Rev. 2019:1–21. https://doi.org/10.1080/17437199.2019.1625282.
25. Tarrant M, Khan SS, Farrow CV, Shah P, Daly M, Kos K. Patient experiences of a bariatric group programme for managing obesity: a qualitative interview study. Br J Health Psychol. 2017;22(1):77–93.

For peer review only - http://bmjopen.bmj.com/site/about/guidelines.xhtml

26. Haslam C, Cruwys T, Haslam SA, Dingle G, Chang MX-L. Groups 4 Health: evidence that a social-identity intervention that builds and strengthens social group membership improves mental health. J Affect Disord. 2016;194:188–95.
27. Peninsula Childhood Disability Research Unit. Our Family Faculty. http://www.pencru.org/getinvolved/ourfamilyfaculty/. Accessed 29 Jan 2019.
28. Bartholomew LK, Parcel GS, Kok G, Gottlieb NH, Fernandez ME. Planning health promotion programs: an intervention mapping approach. 4th ed. San Francisco: Jossey-Bass; 2016.
29. Borek AJ, McDonald B, Fredlund M, Bjornstad G, Logan S, Morris C. Healthy Parent Carers programme: development and feasibility of a novel group-based health-promotion intervention. BMC Public Health. 2018;18(1):270.
30. Muehlhausen W, Doll H, Quadri N, Fordham B, O'Donohoe P, Dogar N, Wild DJ. Equivalence of electronic and paper administration of patient-reported outcome measures: a systematic review and meta-analysis of studies conducted between 2007 and 2013. Health Qual Life Outcomes. 2015;13(1):167.
31. Hammond P. Staying alive: how to get the best out of the NHS - advice from a doctor. London: Quercus; 2015.
32. Aked J, Marks N, Cordon C, Thompson S. Five ways to wellbeing: a report presented to the foresight project on communicating the evidence base for improving people's well-being. London: Nef; 2009.
33. Williams U, Rosenbaum P, Gorter JW, McCauley D, Gulko R. Psychometric properties and parental reported utility of the 19-item 'About My Child' (AMC-19) measure. BMC Pediatr. 2018;18(1):174.
34. Department for Communities and Local Government. English indices of deprivation 2015. https://www.gov.uk/government/statistics/english-indices-of-deprivation-2015. Accessed 12 May 2017.
35. Tennant R, Hiller L, Fishwick R, Platt S, Joseph S, Weich S, Parkinson J, Secker J, Stewart-Brown S. The Warwick-Edinburgh mental well-being scale (WEMWBS): development and UK validation. Health Qual Life Outcomes. 2007;5(1):63.
36. National Institute for Health and Care Excellence. The social care guidance manual. London: NICE; 2016.
37. Kroenke K, Spitzer RL. The PHQ-9: a new depression diagnostic and severity measure. Psychiatr Ann. 2002;32(9):509–15.
38. Kroenke K, Spitzer RL, Williams JB, Löwe B. The patient health questionnaire somatic, anxiety, and depressive symptom scales: a systematic review. Gen Hosp Psychiatry. 2010;32(4):345–59.
39. Wittkampf KA, Naeije L, Schene AH, Huyser J, van Weert HC. Diagnostic accuracy of the mood module of the Patient Health Questionnaire: a systematic review. Gen Hosp Psychiatry. 2007;29(5):388–95.
40. National Collaborating Centre for Mental Health, Royal College of Psychiatrists. Common mental health disorders: identification and pathways to care: RCPsych Publications; 2011.
41. National Institute for Health and Care Excellence. Depression in adults: recognition and management. https://www.nice.org.uk/guidance/cg90. Accessed 21 Jan 2019.
42. The EuroQol Group. EuroQol-a new facility for the measurement of health-related quality of life. Health Policy. 1990;16(3):199–208.
43. Kiplinger VL, Browne CH. Parents' assessment of protective factors: user's guide and technical report. Washington: Center for the Study of Social Policy; 2014.
44. Bourke-Taylor H, Law M, Howie L, Pallant JF. Initial development of the Health Promoting Activities Scale to measure the leisure participation of mothers of children with disabilities. Am J Occup Ther. 2012;66(1):e1–e10.
45. Muskett R, Bourke-Taylor H, Hewitt A. Intrarater reliability and other psychometrics of the Health Promoting Activities Scale (HPAS). Am J Occup Ther. 2017;71(4):7104190010p7104190011–8.
46. Hibbard JH, Mahoney ER, Stockard J, Tusler M. Development and testing of a short form of the patient activation measure. Health Serv Res. 2005; 40(6p1):1918–30.
47. Hibbard JH, Stockard J, Mahoney ER, Tusler M. Development of the Patient Activation Measure (PAM): conceptualizing and measuring activation in patients and consumers. Health Serv Res. 2004;39(4p1):1005–26.
48. Al-Janabi H, Flynn TN, Coast J. Development of a self-report measure of capability wellbeing for adults: the ICECAP-A. Qual Life Res. 2012;21(1):167–76.
49. Al-Janabi H, Peters TJ, Brazier J, Bryan S, Flynn TN, Clemens S, Moody A, Coast J. An investigation of the construct validity of the ICECAP-A capability measure. Qual Life Res. 2013;22(7):1831–40.
50. Mitchell PM, Al-Janabi H, Byford S, Kuyken W, Richardson J, Iezzi A, Coast J. Assessing the validity of the ICECAP-A capability measure for adults with depression. BMC Psychiatry. 2017;17(1):46.
51. Ridyard CH, Hughes DA, Team D. Development of a database of instruments for resource-use measurement: purpose, feasibility, and design. Value Health. 2012;15(5):650–5.
52. Moore GF, Audrey S, Barker M, Bond L, Bonell C, Hardeman W, Moore L, O'Cathain A, Tinati T, Wight D. Process evaluation of complex interventions: Medical Research Council guidance. BMJ. 2015;350:h1258.
53. Ridyard CH, Hughes DA. Taxonomy for methods of resource use measurement. Health Econ. 2015;24(3):372–8.
54. Eldridge SM, Chan CL, Campbell MJ, Bond CM, Hopewell S, Thabane L, Lancaster GA. CONSORT 2010 statement: extension to randomised pilot and feasibility trials. Pilot Feasibility Stud. 2016;2(1):64.
55. Braun V, Clarke V. Using thematic analysis in psychology. Qual Res Psychol. 2006;3(2):77–101.
56. International Committee of Medical Journal Editors. Defining the role of authors and contributors. http://www.icmje.org/recommendations/browse/roles-and-responsibilities/defining-the-role-of-authors-and-contributors.html. Accessed 18 Mar 2019.
57. NHS England: NHS England's commitment to carers; 2014.

Publisher's Note

Springer Nature remains neutral with regard to jurisdictional claims in published maps and institutional affiliations.

Ready to submit your research? Choose BMC and benefit from:

- fast, convenient online submission
- thorough peer review by experienced researchers in your field
- rapid publication on acceptance
- support for research data, including large and complex data types
- gold Open Access which fosters wider collaboration and increased citations
- maximum visibility for your research: over 100M website views per year

At BMC, research is always in progress.

Learn more biomedcentral.com/submissions

For peer review only

Healthy PARENT CARERS

Facilitator training feedback form

During this phase of the research we are still developing and finalising the facilitator training. Your feedback will help us to ensure that any future training delivered is as comprehensive and useful as possible.

Your feedback is anonymous and you will not be identified in any research reports. This information will only be viewed by the research team.

1. Please rate, on a scale of 1-5, your knowledge and understanding, and skills and confidence in the following areas (with 1 being very little and 5 being a lot).

**Knowledge and understanding of:**	**1**	**2**	**3**	**4**	**5**
The intervention's theoretical framework (CLANGERS); the session activities and associated learning outcomes.					
Group facilitation techniques and how to create a positive group atmosphere.					
Particular challenges faced by parent carers and how this may impact on health and wellbeing.					
The role of the HPC Lead/Assistant Facilitator.					
The study design.					
Safeguarding procedures.					

**Skills and confidence**	**1**	**2**	**3**	**4**	**5**
Ability to confidently present programme information, lead activities and facilitate discussions.					
Ability to create a positive group atmosphere and inspire collective desire to make lifestyle changes to improve health and wellbeing.					
Ability to manage time effectively.					
Ability to manage difficult/sensitive issues that may arise.					

For peer review only

2. The time allotted for the training was sufficient** (**Please tick one)

Strong Agree	Agree	Neutral	Disagree	Strongly Disagree

3. Are there any aspects of the training that could be improved (e.g. content, time provided to cover the learning objectives and/or delivery)?

4. Any other comments?

Many thanks for taking the time to give your feedback.

For peer review only

Healthy
PARENT CARERS

CHECKLIST FOR MODULE 1 DELIVERY

Instructions to the Facilitators and Assistant Facilitators:
Please complete this checklist at the end of each session to provide us with feedback on what was covered and how the session went. This feedback will help us assess if the sessions can be delivered as planned and identify potential challenges and improvements.

Date & time of the session: ____

Session number: _________________________

Venue: ____

Facilitators' names: ____

Session duration (as delivered): ________________________

Number of participants attending: ______________________

**Activities**	**Delivered** (Please tick if delivered)	**Comment** (If partly or not delivered, please explain why)
1. Introductions / icebreaker activity		
2. Hopes and fears		
3. Ground rules		
4. What are health and wellbeing?		
5. What influences health and wellbeing?		
6. The resilience game		
7. Conclusion		
Time for informal interaction (e.g. tea break)		

For peer review only - http://bmjopen.bmj.com/site/about/guidelines.xhtml

<u>Reflections on the session</u>

Please indicate which picture best describes your perception of how well connected, in general, the attendees of today's session appeared to be (tick the box on the right of the picture).

For example, the first picture would suggest that there was very little connection between individuals – that they did not come together as a group at all. Whereas the final picture would suggest that individuals were very closely connected – they very much came together as a group.

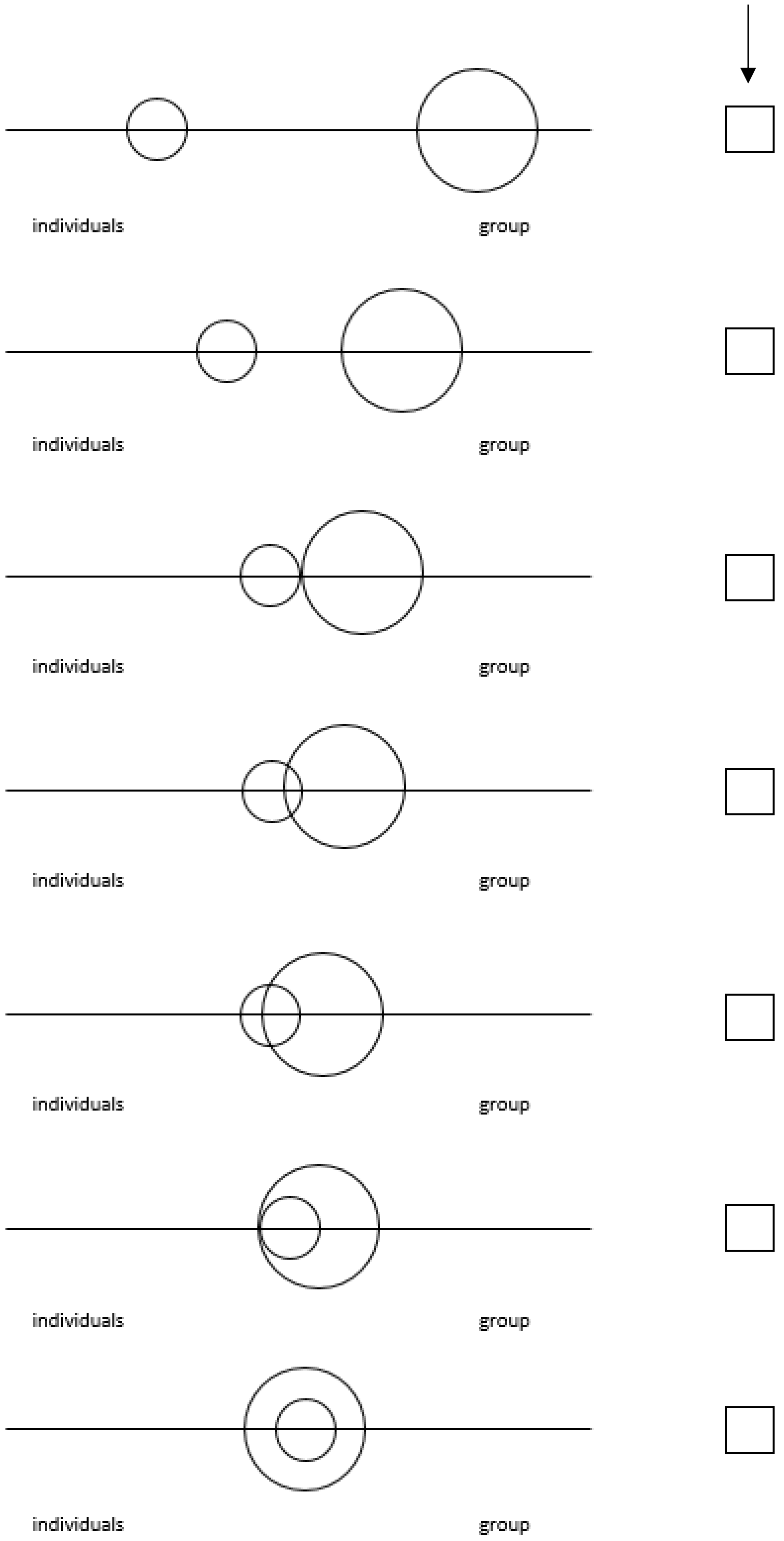

Reflections on the session cont.

Please circle one

In your opinion, how well did today's session go?

Not well at all				Very well
1	2	3	4	5

How engaged were the participants in today's session?

Not well at all				Very well
1	2	3	4	5

Do you have any comments or reflections?

(For example, related to how the group worked together, participants' engagement, session content or activities)

For peer review only

For peer review only - http://bmjopen.bmj.com/site/about/guidelines.xhtml

Healthy PARENT CARERS

Draft topic guide for a focus group with HPC Facilitators

Topics and example questions for the focus group with HPC Facilitators and Assistant Facilitators:

- Motivation for / expectations of delivering the group programme, e.g.:
    - Why did you decide to get involved in this study?
    - What did you expect from taking part in the study / delivering the programme?
- Views about training / preparation to deliver the programme, e.g.:
    - How did you find the training to deliver the programme? (Prompt about whether it was sufficient, anything that would be helpful)
    - How prepared did you feel to deliver the programme? (Prompt about their background and previous experience, or any new skills developed by delivering HPC)
- Experience of delivering the programme / group facilitation, e.g.:
    - Having delivered the programme now, what do you think about it?
    - Generally, how did you find delivering the programme? (Prompt about following the Facilitator Manual, any adaptations made, challenges, how feasible it was to deliver)
    - How engaged was the group / the participants?
    - How did you find facilitating / managing the group? (Prompt about any challenges and how they managed those, how engaged they were in delivering it)
- What worked best in the programme? Why? (Prompt about activities, content, group)
- What didn't work well? What were the challenges or difficulties? Why?
- Do you have any suggestions for improvements? (e.g. related to the programme delivery, content, Facilitator Manual, training)
- Is there anything else that we haven't talked about and that you'd like to discuss?

For peer review only

For peer review only - http://bmjopen.bmj.com/site/about/guidelines.xhtml

HEALTHY PARENT CARERS

HEALTHY PARENT CARERS
WEEKLY PARTICIPANT FEEDBACK FORM FOR GROUP INTERVENTION

GROUP: ____

SESSION NUMBER: ______________________________________

We would like to know what you think about this session and would be grateful for your feedback. The feedback is anonymous and it will help us refine and improve the programme in future.

Please circle one

1. Overall, how satisfied are you with today's session? (please circle one)

Not satisfied at all				Very satisfied
1	2	3	4	5

2. Do you feel that you were able to take an active part in the session? (please circle one)

Not at all				Very much
1	2	3	4	5

3. Please tell us about the best things in today's session (e.g. most enjoyed or most helpful):

4. Please tell us about the things that could be improved in today's session:

For peer review only

5. Which picture best describes how well connected, in general, you felt to the rest of the group today?

For example, the first picture would suggest that you felt very little connection to the rest of the group, whereas the final picture would suggest that you felt very connected to the group.

Please tick the box on the right hand side of the picture that best describes how you felt.

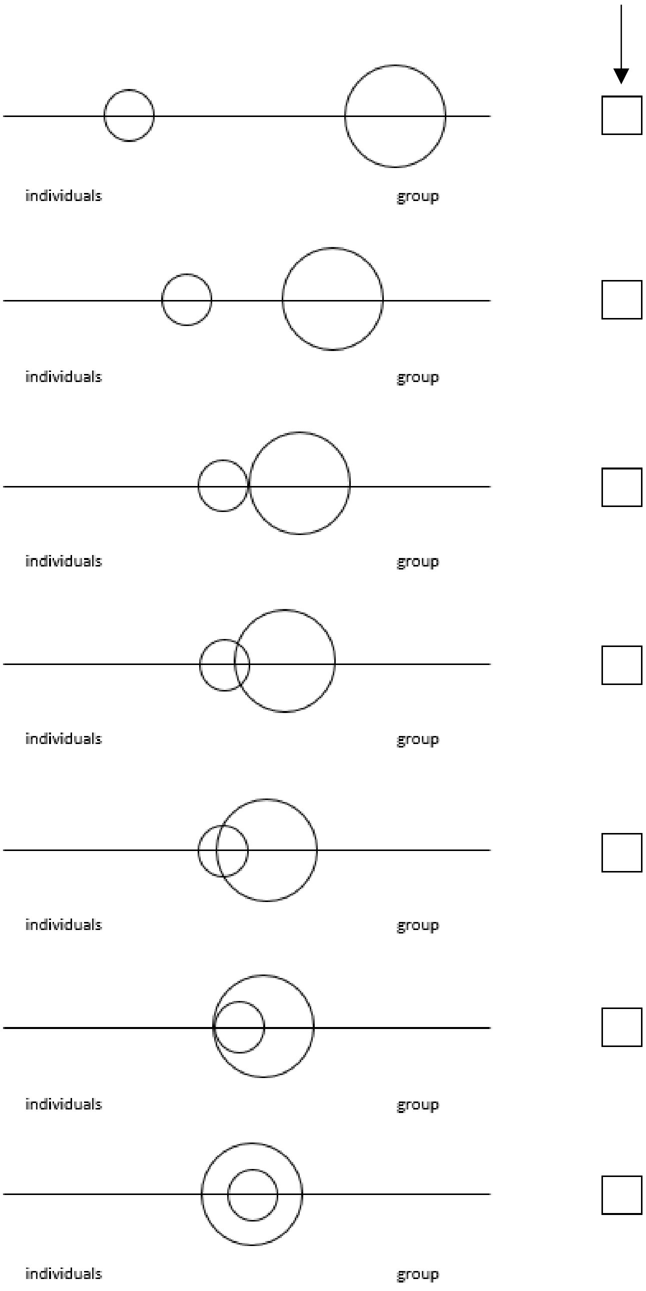

6. How much did you identify with other members of this group? (please circle one)

Not at all				Very much
1	2	3	4	5

HEALTHY PARENT CARERS
Feedback FORM

We would like to know what you think about the Healthy Parent Carers programme and the research study and would be grateful for your feedback. The feedback is anonymous and it will help us refine and improve the programme and our research in future.

1. Overall, how satisfied are you with taking part in the Healthy Parent Carers programme?

Not satisfied at all — Very satisfied

1 2 3 4 5

2. How useful has the programme been in helping you improve your health and wellbeing?

Not at all — Very much

1 2 3 4 5

Was there anything in particular that helped you?

3. Have you made any changes as a result of taking part in the programme? No / Yes

If yes, what?

If no, why?

4. Approximately how many of the online materials have you accessed?

None	25%	50%	More than 50%

5. Did you attend group sessions for the programme? Yes / No

If yes, reveal questions 6-10, then continue from question 12.

If no, reveal question 11, then continue from question 12.

6. How would you rate the length of the individual sessions?

Too short	About right	Too short

For peer review only

7. Did you miss any of the group sessions? Yes / No

If yes, why?

8. Overall, how much did you feel included and part of the group?

Not at all				Very much
1	2	3	4	5

9. How satisfied were you with the way the sessions were facilitated and delivered?

Not satisfied at all				Very satisfied
1	2	3	4	5

10. Did you discuss the programme with anyone who had access to the online materials only (i.e. they did not attend the group sessions)? Yes / No

11. Did you discuss the programme with anyone who attended the group sessions? Yes / No

12. Do you have any suggestions about how the programme or materials could be improved?

13. Would you recommend this programme to other parent carers?

Not at all				Very much
1	2	3	4	5

14. Do you have any other comments, reflections or suggestions?

For peer review only

Appendix 2: Data collection materials

Follow-up questionnaire
Version 1, 11 June 2019

HEALTHY PARENT CARERS
Follow-up questionnaire

1. Have you continued to access the online resources in the past 6 months?

Yes / No

2. Approximately how many of the online materials have you accessed in total?

None	25%	50%	More than 50%

3. Did you attend group sessions for the programme? Yes / No

If yes, reveal question 4.

If no, end survey.

4. Since the Healthy Parent Carers group ended, how many members of your group have you been in touch with (including members that you knew prior to starting)?

(number up to 10)

5. Out of these, how many were people that you did not know before the programme?

(number up to 10)

6. Since the group ended, have you been in touch with any members the group that you didn't know before in any of the following ways?

(select all that apply)

Phone

Text

Email

WhatsApp

Other Social media (e.g. Facebook, Twitter)

Meeting in person

Other (please specify – please do not include anyone's name) (free text box needed if ticked)

For peer review only - http://bmjopen.bmj.com/site/about/guidelines.xhtml

Appendix 2: Data collection materials

Follow-up questionnaire
Version 1, 11 June 2019

I have not been in touch with any of the group members that I didn't know before

7. Approximately how long ago was the last time you had contact with any members of the group that you didn't know before it started (please do not include anyone's name)?

(free text)

8. Can you please describe how often you have been in touch with any members of the group that you didn't know before (please do not include anyone's name)?

(free text)

9. How many of these new contacts would you now say are your friends?

(number up to 10)

For peer review only

Healthy
PARENT CARERS

Draft interview topic guide: HPC Participants (intervention arm)

- Introduce myself (PenCRU childhood disability research unit)
- The aim of the interview is to get their feedback on the HPC programme and research study
- Confirm how much time interviewee has for interview. Confirm that meant to take about 30 mins but can vary depending on length of discussion – let me know if you have somewhere you need to be and we can manage accordingly
- Remind participant that taking part is voluntary – if I ask about something you don't want to talk about, please just say you'd rather not go in to that
- Remind participants that the interview will be recorded and transcribed and analysed to help us understand how the programme and study worked. Interview transcripts will be anonymous.
- Check participant is comfortable (has drink of water etc.) and has given consent to continue

Record before/after the interview:

- Participant ID:
- Participant's group ID & facilitators' IDs:
- Gender:
- Age:

Topics and example questions for participants in the intervention arm:

- Motivation to participate & expectations, e.g.:
  - Why did you decide to take part in the study / programme?
  - Was there anything that helped you to decide to take part?
  - What did you expect from taking part in the study?
- Understanding of, and views on, the study design, e.g.:
  - How sufficient did you find the information about the study & the programme and what they involve?
  - What did you think when you found out that you were assigned to the group programme, rather than only being given access to online materials?
  - Would you change any aspect of how it was explained to you, or what information was available, about the programme or the research? (probe suggestions from them)
- Experience of the group programme (activities, group):
  - What do you think about the content of the programme? (prompt about relevance "relevant to you as a parent carer", helpfulness of the content e.g. CLANGERS)
- Views on length & frequency of sessions; any missed sessions & reasons for missing sessions
  - What do you think about the length and frequency of the sessions? Did you need to miss any sessions? (prompt about reasons for missing sessions)
- Views on & engagement with the group:
  - Tell me about your group. Did you feel included and a part of it?
- Views about facilitators and delivery style, e.g.:

For peer review only

  - What do you think about the facilitators and the way they delivered the programme? How well did they do in delivering the programme and facilitating the group? (prompt about facilitator engagement)
- Usage of, and views on, the online programme materials
  - Did you access the programme materials online? (prompt about how they used them/how often, or about reasons for not using them) If yes, what prompted you to look at them?
- Impact of the programme & any changes made, e.g.:
  - How helpful did you find the programme? Did you make any changes in result of the programme? (prompt for examples) Was there anything that got in the way of you being able to make those changes? Was there anything that supported you to make those changes?
- Contextual influences, e.g.:
  - Did anything affect how much you got out of participating in the programme? Were there any barriers to you engaging with or benefiting from the programme?
- Best & worst things, suggestions for improvements, e.g.:
  - What did you like most / found most helpful (if anything) about the programme?
  - What did you not like / did not find helpful (if anything)?
  - Do you have any suggestions for how the programme could be improved? (prompts: delivery, content, group, timing / length of programme)
- Acceptability of data collection and measures, e.g.:
  - How did you find having to fill in all the questionnaires at the beginning and at the end of the study? (prompt about time, clarity, relevance)
  - The sessions were audio recorded. How would you have felt if the sessions had been video recorded instead?
- Is there anything else that we haven't talked about and that you'd like to mention?

For peer review only

Healthy PARENT CARERS

Draft interview topic guide: HPC Participants (control arm)

- Introduce myself (PenCRU childhood disability research unit)
- The aim of the interview is to get their feedback on the HPC programme and research study
- Confirm how much time interviewee has for interview. Confirm that meant to take about 30 mins but can vary depending on length of discussion – let me know if you have somewhere you need to be and we can manage accordingly
- Remind participant that taking part is voluntary – if I ask about something you don't want to talk about, please just say you'd rather not go in to that
- Remind participants that the interview will be recorded and transcribed and analysed to help us understand how the programme and study worked. Interview transcripts will be anonymous.
- Check participant is comfortable (has drink of water etc.) and has given consent to continue

Record before/after the interview:

- Participant ID:
- Gender:
- Age:

Topics and example questions for participants in the control arm:

- Motivation / expectations to participate, e.g.:
    - Why did you decide to take part in the study / programme?
    - Was there anything that helped you to decide to take part?
    - What did you expect from taking part in the study?
- Understanding of, and views on, the randomised controlled design and control group, e.g.:
    - How sufficient did you find the information about the study and the programme, and what they involve?
    - What did you think when you found out that you were assigned to the group with access to online materials only and not the group programme?
    - Would you change any aspect of how it was explained to you, or what information was available, about the programme or the research?
- Contamination with the intervention arm, e.g.:
    - Do you know anyone who also took part in the study and attended a group? (If yes, prompt about how much they discussed what happened in the group and how much that might have influenced their view on the programme materials or changes made).
- Usage of, and views on, the online programme materials, e.g.:
    - Did you access the programme materials online? (prompt about how they used them/how often, or about reasons for not using them)
    - What do you think about them?
- Impact of the programme, any changes made, e.g.:

For peer review only

    - How helpful did you find the materials? Did you make any changes in result? (examples) Was there anything that got in the way of you being able to make those changes? Was there anything that supported you to make those changes?
- Views on the data collection and measures
    - How did you find having to fill in all the questionnaires at the beginning and at the end of the study? (prompt about time, clarity, relevance)
- Best & worst things, suggestions for improvements, e.g.:
    - What did you like most / found most helpful (if anything) about the programme?
    - What did you not like / did not find helpful (if anything)?
    - Do you have any suggestions for how the materials or the research elements could be improved?

Is there anything else that we haven't talked about and that you'd like to mention?

For peer review only

Supplementary Document 11. Additional quotes and comments

1. Facilitators' experiences

1.1. Motivations and expectations

New experiences, new skills & being involved in the research project. (Facilitator pre-training questionnaire)

Recognised the need for healthy wellbeing of parent carers through myself and friends. Facilitate a small support group locally. Ready for a new challenge. (Facilitator pre-training questionnaire)

To apply my training delivery skills and experience to a programme which is unique in its focus on parent carer health, something myself as a parent carer feel I could directly benefit from. (Facilitator pre-training questionnaire)

CLANGERS has changed my life and I feel it is really important to help others by spreading the message. It's also a research project and that excites me as well! (Facilitator pre-training questionnaire)

I always believed in this course because it had such a positive impact on me and I just wanted to give back to others. (AF, focus group)

When we did the [training], I do remember thinking some of this is really resonating with me and my own challenges and thinking actually, I can see where this is going, and it has been really, really good. (LF, focus group)

1.2. Training, preparation and support

It became clear to me that there was actually quite a distinctive style that was expected as well… we would all have liked some more concrete direction in terms of what to do and how. (AF, focus group)

It's something actually a little bit more fundamental about that. It's about why are you writing this stuff down? Because there's nothing in the trainer notes that explained to the trainer what the purpose of having this stuff written down is. I always find it difficult delivering training that's been written by somebody else… I had to keep saying in my head, 'do it as it's written, don't go off and do your own thing'. (LF, focus group)

I would [practise] 'Connect' because it would give you the opportunity to identify some of the issues that are going to come up, because 'connect' can be really difficult for parent carers. So you could use it as, well this is an example of somebody who struggles, how would you do it in this situation, how would you cope with this? So it can be quite an emotional one and it's also the first one that we do in the programme… The group were also very nervous and quiet in that one, so that makes it harder. (AF, focus group)

LF: …trainers probably need to undergo the training in the first instance because I think you need to experience the programme, both to have a better understanding but you would get a lot of ideas and facilitation through that.

For peer review only

AF: I felt I was at an advantage because I had done it… because I knew the course I was able to listen and scribe, knew what was coming up because I'd done it before, but I was also able to input in my way and from my experiences… which I felt helped. So it wasn't new information to me, I'd already gone through that quite hard process of thinking and I had already adjusted and adapted and was aware of it and everything else. And you could see it on the faces of the participants that actually it was hard at times and I had been there, so I understood. (Focus group)

More time for assistants to get a clearer understanding of breath of exercises / module content. (Assistant training, feedback form)

It's all very hurried. More time practicing our role. (Refresher training, feedback form)

1.3. Programme content and group delivery

It's tricky to manage but more difficult with some groups than others, definitely… but I think for the most part parents actually said it was really nice to focus on themselves. (LF, focus group)

LF: I would be very tempted to remove SMART from there and just replace it with achievable.
AF: The most important thing about CLANGERS is that you take ownership of it and you become aware that that's what you're doing. So it's important to have those goals but it's also important to recognise when you're not achieving those goals, where did it fall down. (…) so actually you might not have achieved that goal but you've actually done bits of CLANGER here instead. So it's just raising that awareness and your own understanding and building up that way. (Focus group)

The fact that we had such small groups was really disappointing for me… I think it changed the nature of the course, being such small groups, it was far more intense, I believe, for the training team, than it would have been if we'd had lots of participants there who were able to share their views and other things. So yes, altogether a highly enjoyable and positive experience but a few things that, perhaps, my expectations were slightly different. (LF, focus group)

I think the key of the leaders being parents of children with disabilities just really, really helps everybody to bond and understand. For me, I'm like wow, you've been through it, you're this much further on the same journey that I'm on and look at you; you're out here and you're doing this and you're working and, oh, your life is okay… That's reassuring. (AF, focus group)

All participants were very engaged. Unfortunately, one has a lot of problems currently so found it difficult to discuss resilience without being extremely emotional. Other participants very supportive. (Facilitators session checklist)

Group extremely connected. Tearful at the end of evening. Have made arrangements for continued contact as a group. Great sharing + reflection of course. (Facilitator checklist at the end of the final session)

2. Participants' experiences and views

2.1. Motivation to participate and expectations

For peer review only

Wanting to improve health and wellbeing:

I feel that being a parent-carer is really draining on your mental health and also I wanted to try and get healthy anyway, especially with it being January. Yes, I just thought it would be a good thing to try out and see if it does help me. (…) a bit of knowledge maybe about the way I feel and maybe a bit less guilt, the fact that I feel that I need to take time out for myself. (P13, control)

I was aware that my own health, fitness, mental health. All of those things really have taken quite a hit over the years, so it was a bit of a chance to actually focus on that for probably the first time in a long time really. So that was my main motivation. (…) It just seemed to fall into my lap at the right moment, so I, yes, sort of grabbed the opportunity with both hands really. (P3, intervention)

We decided to take part in the study because we wanted to be proactive as we are on most things. We knew that we needed to help ourselves as carers because we've become carers abruptly and inadvertently. (…) We've been using all the materials together [with his wife]. And we wanted to take part because we aren't going to sit on our arses and wait for something to happen because what we've learnt is the public sector will do nothing for you unless you poke it very hard. (P14, control)

I just thought it could be useful because a lot of the time I find as a parent carer all the focus is on the children which is obviously the main thing but the parents need to be healthy and happy to give the children the best. (P8, intervention)

Expecting advice, resources and signposting:

I was hoping just to find some resources to help me turn the attention off my daughter and more onto myself, just learn how to look after myself in amongst the very challenging times that we have looking after my daughter. (P15, control)

It was sort of different to what I expected because, like I say, I was expecting more sort of direct, "Right, this is who you can go to," rather than sort of what seemed like giving people information that they probably already knew. (P7, intervention)

Good timing of the programme:

If you wanted me to do the course at the beginning of last year I wouldn't have been able to do it because my daughter was very ill, but now she's in a good place at the moment so I could do it. (…) I've got to that point in my life that I have to say right, [my daughter] will always come first, she has to, and my husband and the rest of the children and grandchildren. But sometimes the work you do has to change, it has to go in a different direction (…) I've just always got on with it, but I think now I've got to that point that I think right, things have to change. (P11, intervention)

I just thought that it just fitted with the times in my life that I wanted to try and get a bit healthier and a bit less stressed. (P13, control)

Probably the fact that it was on Monday night. I've got very few timeslots that are regularly available and that one happens to be free, so. I think that probably was the biggest thing that made me go, yes let's just do it. (P2, intervention)

For peer review only

Wanting to contribute to research:

I thought it would be really helpful for me and I just felt it is quite important research, because I can't say I've looked up if there is research in this area, but I don't think there is a lot of research and I don't think unless you are in the situation, no one really gets the impact on your life and your health and everything else, it just takes over. So it just felt important for me to do for myself and also part of a bigger picture really because I think they are many many people in this situation and I don't think there's much light shed on it. I think it's getting better. But it just felt like an important thing to be part of really. (P3, intervention)

Well I thought it would help me with day-to-day stuff, but it's also an area that I have looked for help with in the past and found that there isn't any specifically, mental and physical health specific to parent carers, so I wanted to contribute to it because I want it to work [laughs], I want it to be there for when people need it. (P6, intervention)

Preference for group programme:

I was just excited to be involved in something and potentially going to be around other people… (…) I was hoping to meet other people who are in a similar situation to me and I think that you can learn a lot that way and also it's a support, meeting other people. Particularly for me, I'm very isolated and I'm assuming that there are other parent carers that are the same, that you can become very isolated and also quite often friends and family don't really understand, they say they do but you know from their actions and the things that they say sometimes that they don't really understand. So you can feel quite lonely. So having the opportunity to know that I'm going to be with other people who are in a similar situation, I was really pleased about. (P4, intervention)

I just thought that was going to be a lot more likely to be, well, something I would see through because there's a kind of commitment to turning up each week and I'm dreadful for just dropping out of things and going, oh whatever, I'm too busy… (P2, intervention)

I was a bit gutted really because I know that I always struggle with things on computers. It's not because I struggle with computers, but I always put it off to the next day and the next day, whereas if it was a group, I would just go to it as if it was an appointment. (P13, control)

I would have liked to have been part of a group to be able to discuss it with as a parent going to do similar things. (…) If there was to be a Healthy Parent workshop here in [town], I would have done it, I would have been able to find a way and I think I probably would have got more out of it from just sharing experiences and getting to know other people just for networking and support for each other. (P15, control)

Preference for, or acceptance of, online-only programme:

I wouldn't have been able to attend the groups anyway. I'm working 12-hour days, so it would have been a real rush. If you'd assigned me to the group, I probably would have asked to either drop out or go online anyway. So it worked out well in the end. (P14, control)

I was disappointed [when found out about being assigned to the group programme] because I was quite looking forward to doing the online material where I didn't have to go out, see people and talk to people, but again that is just my personal preference. It is perfect because it pushed me out of my comfort zone. I was quite apprehensive on the first day. (P9, intervention)

For peer review only

Feeling nervous before the first session:

It took a lot of self-will to take myself to the first session. It was one of those where when it came to it I was really excited and getting ready for it, then when it actually came to it I suddenly was trying to find excuses. (…) It was the sheer fact that just as it was about to start, everything around me became 10 times busier. (…) I think that was my main excuse for why I was allowing apprehension to take over. I think it is always that self-doubt factor of, is it going to be any use to me? Am I going to have to talk to loads of people and I don't really want to? It was a combination of those factors I think. (P9, intervention)

2.2. Experiences of the programme and its impact (and mechanisms)

Positive experiences:

It was fantastic. I think it was really well done. (…) I think it's changed my life. I really do. I haven't stopped talking about it since. Yes. It just makes complete sense. (…) I think the best thing was going to the group, meeting everybody in the group, just meeting like-minded people, having a break from life and being in a different place for a while, reflecting on aspects of my life that I didn't really think about or had put away, and I learnt more about myself. (P5, intervention)

I thought it was brilliant. It really has opened my eyes on an awful lot of things and I have changed an awful lot in my life. (P10, intervention)

The impact of a course like that, if that keeps me going so that I can look after my daughter, if it helps me figure out, work out our stresses in our family, keeps my relationship together, that has not just impact for our family obviously but wider in terms of taxpayers' money. So for me, I think it's really important. (P4, intervention)

I suppose they made you feel uncomfortable, which once you have got over the feeling uncomfortable about the fact that you have analysed something and thought oh I don't really like that and you can kind of box it but you think well actually no I need to address that. But that's just more about taking you out of your comfort zone really. But that's not really a bad thing, that's just an uncomfortable thing. (P1, intervention)

Lots of things! Meeting people who understand and 'get it ' reduced my isolation. Having parent carers as facilitators helped a lot. Having 12 structured sessions made me commit to it and focus on my own health. Having structured fun informative sessions helped a lot - I definitely went through a process of change. The use of humour was really important to me - and learning to be more resilient. (End-of-programme feedback)

Learning how useful and how important it is to keep myself healthy, not only for myself but for my child family and friends. Learning how to reflect and realising the amount I already do for myself and others that I am now proud of achieving a good sense of self-worth. Using Smart goals. (End-of-programme feedback)

I just think it was excellent and totally life changing!! Every parent carer in the UK and worldwide should have access to this course!! Thank you so so so much!! (End-of-programme feedback)

For peer review only

The group:

Being part of a group feeling like my challenges were understood and acknowledged. We laughed a lot cried sometimes and developed a lovely supportive friendship. I felt that the course was a really good balance of talking to off-load whilst also being 'solution focussed'. This was very empowering. Whilst my situation remains very challenging, I feel I have the skills and knowledge to help myself whilst also now having a wonderful new group of supportive friends. I am so grateful for this experience. Thank you. (End-of-programme feedback)

Our group worked really well actually and it really evolved, which was lovely because there were people who were thinking they weren't going to make it through to the end and they did. (…) We had quite an empathetic group and there was sort of, now that I'm thinking about it, possibly an unwritten code. (…) There was a lot of respect in the group. Obviously, there's some people whose kids have mental health issues and having major issues. There was a lot of well let's just sit and make sure we listen. There was a lot of support and everybody injected into the study, nobody sort of sat back, or if they were uncomfortable they said they were. As a group, it did, it evolved and it moulded and it's a really nice group. (P1, intervention)

I found it all helpful. I think the… and again, because it was a group, and that's why I would say I'm really glad that I was on the group side of it, the interaction and people's knowledge and people's just experiences and you kind of… but that sort of camaraderie type of feeling really, that's probably what I got more… probably, and then, yes, you put that down and the connecting that we did really. There's lots and lots of connecting really and… Yes, there was lots and lots of all of it actually, thinking about it, but just people putting through ideas of, "Well I do this, I don't know if that would work for you". There was a lot of that and it was, yes, that's probably what I got mostly from it. Yes. (P1, intervention)

It was a really nice group of people, everyone was very… I'd say everyone was very open and honest, people were able to talk about quite difficult circumstances and situations and it felt like a supportive environment and people were able to get upset and feel supported I think and also we did have a really good laugh, which was brilliant. I think we were really lucky. We are planning to keep in touch. Typical carers, no one can find the time when we are all available. It just sums it up really, doesn't it? But it was such a positive experience. After the first night I came out on a bit of a high because it's just always so… because friends and family and they care, but people just don't get it in the same way as when you are with a group of people who just know how bleak it can be, yes. (P3, intervention)

What I loved about the course was that it was very solution focused and I think quite often you can go to groups and things that it's just about talking, and as helpful as that is, you still come away with it going, well I'm not really sure… It was nice, but I don't really know how to make things better still, so to go to a group that's like you have the opportunity to talk and to gain help from other people and be listened to, great, but let's put that to one side, what are the barriers and how can we overcome them and let's work together to help each other overcome our barriers to having a more healthy and greater wellbeing in our lives. So I think if I'd have known that beforehand, I would have been even more excited. So maybe to have known that that was what it was trying to achieve. (P4, intervention)

For peer review only

I liked being around other people who were in a similar position to me. I liked the fact that we could listen to each other and offer support and help and useful advice. There was a lot of that that went on between us. Thinking creatively about how… from everybody's own experiences, what knowledge do I have that can help you? So I liked the supportiveness of the group. It wasn't all serious either, we laughed a lot, and that was lovely to have those moments where we shared silly jokes. It really was a case of we laughed together and we cried together, do you know what I mean, and we helped each other. So it was a really lovely group to be in and I really liked that. (P4, intervention)

We all got to share our stories, we all got to know each other, we all gave each other presents at the end and had a little party. It was really good. I think we all really became different people throughout the course. (…) I think the best thing was going to the group, meeting everybody in the group, just meeting like-minded people, having a break from life and being in a different place for a while, reflecting on aspects of my life that I didn't really think about or had put away, and I learnt more about myself. (P5, intervention)

When somebody was missing it kind of really felt like somebody was missing because we all had very different perspectives and different situations and it was really interesting. The biggest part of the thing for me was not what came out of the structure but out of the conversations that we had between us. Everybody interacted equally with everybody else across the six weeks, so there wasn't like two people who got on really well and had lots more in common and went off and talked between themselves, it was like completely among the group and it wasn't… In some weeks one of us might be quiet for our own reasons and that was fine. Everybody was very… I don't know, sort of accepting of other people's contribution, whether they were contributing a lot or not very much that week. (P6, intervention)

Everybody was listening and it was nice… near the end of the… the latter sessions, we were very much… if you understand what I mean, as a group, we were able to have little jokes together and bring up things that we discussed before. It was very nice. It was very much a camaraderie if you know what I mean. It was really nice and everybody was nice to everybody. There was not any anger or frustration. You could see people were struggling with certain things. We were able to advise them 'Actually, you are better than what you think' so it was really nice. (P12, intervention)

<u>Group size:</u>
It was smaller than I expected. I was expecting a group twice the size. And in some ways that might have been better because it might have been easier to find maybe subgroups within that that I could get… because there were definitely individuals in that group I could definitely get along with better but instead because it was very small it felt quite kind of intense which then meant that, yes, each person ends up trying to make it large and that… yes, and in a way that almost makes it harder to share. Yes, I don't know. I think I probably would have preferred a slightly larger group actually, but not massive. (P2, intervention)

There was only three of us, which was kind of a shame there wasn't more but, at the same time, we couldn't have got as much out of it as we did because we all got to share our stories, we all got to know each other, we all gave each other presents at the end and had a little party. (P5, intervention)

I think if the group was a little bit bigger, maybe not massively bigger but maybe, say, nine to ten people, I think you would get more input and there would be more discussion because even though

For peer review only

we were able to discuss things, I think a bit more of an open, broader amount of different people with different lives would make it a lot more interesting. (P12, intervention)

Peer facilitators:

We were sometimes a bit naughty on time when we talked too much, but I think that just happens when you are in that group. But yes, we managed to cover everything. There was ample time. They made sure everyone was included and had a say at every step really. I don't think there was anyone who didn't feel they could contribute or felt left out. (P3, intervention)

[Facilitators were] Brilliant, brilliant. And they were bouncing off each other. It was not just coming from one person. Because sometimes, you know when they do these things and you are only getting it from one perspective and [name] was able to challenge that perspective and then we were all able to challenge it and it was nice to be able to share rather than actually going, 'We are learning off a book that they were using to get their information' but actually, we were having to break it up even more on the sheets in front of us and where they were writing it all down, we were able to take notes and everything, so it was really good. (P12, intervention)

They really kept things going. They both had quite different approaches which I think complemented each other. Yes, it just seemed to work really well as a complete group including the facilitators really. (P3, intervention)

I think it makes a massive difference that they were both parent carers because they obviously have that innate understanding. I think it would have been different if they hadn't been, if the facilitators hadn't experienced some of those issues, I think it would have been very different. So I think that was a massive bonus. Because it's almost like a short hand, isn't it, there's things you don't even have to explain or go into because you just know that there's an understanding there already. So I think that was really helpful. (P3, intervention)

I think that's really helpful and important because it's not… Both of them are parent carers themselves and have been through very difficult situations and were open about sharing some of their challenges and their experiences and I think that that helps you then feel that it's okay to express what you are going through and that it's understood, because I think in those situations where it's being delivered by somebody who is reading the material without having had any of those challenges themselves, makes it less believable, less… like going to a dieting class that's being led by someone who has never had any problems, challenges with their weight, do you know what I mean? (…) It makes it less believable and somebody preaching to you but coming from a place that they have no experience of but they are preaching to you about how you can make changes in your life. And I think for me then I go I'm not sure if I can… It makes me not want to… It's less genuine, I guess. So for me having people facilitate the programme who have both had those challenges and, as I said, were quite open about their own challenges, and each week when we were going through the different clangers or whatever, they were honest at times, saying, "Well I've struggled this week to do it," and so it makes you feel it's okay that I'm struggling with this as well. And we all kind of worked together to help each other and encourage each other and that was a really lovely thing. Really lovely thing. (P4, intervention)

The facilitators of the programme were excellent in that they helped to create an environment where, as I said, it felt safe to talk about these things, that you were understood, that they cared about what you were going through and that very clearly came across from both of the facilitators, that they genuinely cared about what we were all going through and our challenges and also that they were honest about their own challenges. (P4, intervention)

The facilitators were very experienced (…) [they] were really knowledgeable, made us feel very relaxed, and I think that was a great part of it as well. (P11, intervention)

[Facilitator] was great, really good and whist she was delivering a programme she was absolutely excellent at tuning into individual people's situations and responses to things without singling them out. I could see she was being very empathetic and helping different people to get through different bits of it individually but as a group, if that makes sense, which I think was a real skill. She wasn't just delivering a programme, she was also doing it as individuals and again I think being a very small group really helped that. [Facilitator 2], yes, she was lovely. I mean, she was very good, kind. (P6, intervention)

They were lovely. Both of them were fantastic. I cried at times and they were caring, they were welcoming when you got there, they understood and remembered things. You felt like you got to know them and they got to know you and they'd talk about their experiences as well. I couldn't fault them at all, either of them, they were both fantastic. (P8, intervention)

This was doable because the facilitators weren't just 'professionals' preaching at us. (End-of-programme feedback)

The 2 tutors delivering the course were absolutely amazing! Being parent carers themselves really made them part of the group and not just 'teachers'. They were both friendly, welcoming, caring and informative. They made the course something to look forward to each week. (End-of-programme feedback)

Having the course delivered by another parent carer helped you to feel more at ease with disclosing information about your life and to someone who could totally relate and understand you circumstances. (End-of-programme feedback)

Content and activities:
The content of the course was good, it was really helpful and I know that it's going to help me going forward and I know that I am going to keep referring back to that to help me. (P4, intervention)

I think on every feedback form after every week it was like what was good about it and I was like discussion and sharing [laughs] which didn't come directly from the content of the programme but it made that happen. So that for me was the biggest thing because those are the things that stick, someone else's experience or if somebody else is maybe struggling with something, you'd support each other and you would kind of remember those things, you remember the personal stuff, human interaction that you have with people, whichever way, more than stuff you have been told. So I thought that was great. (…) It just seemed like a very natural way with that structure and the structure almost kind of… kind of like disappeared a bit into the background. I mean, sometimes to keep things moving, [facilitator] would have to bring us back to it and move on and stop one thing and start another thing, but it was almost like that became like a supporting thing, so the structure supported what happened in the session, it didn't drive what happened in the session, although it gave it structure but the session didn't hang on the content. (P6, intervention)

I think it would be relevant to everybody actually. I think everyone could probably do with doing that course. I think as parent carers we have additional challenges, obviously certainly lots of the stress and the anxiety associated with your children who are struggling, but I thought that the content

For peer review only

really helped you to focus on how do I keep myself healthy and look after my wellbeing, because if I fall down then I can't support my children. And I think that's really really important because I think so often families don't get support until it's too late. (P4, intervention)

I think it's relevant to all human beings [laughs], but I think putting it in the context of being a parent carer, yes I did find it very helpful and yes they were all the things. I don't think there was anything in there that was kind of superfluous and I don't think there was anything in there that was, I don't know. Yes, just when you blend them, when you blend the CLANGERS, they cover everything in life really. So parent carers, I didn't feel there was anything there, they were all things that were accessible. So there weren't things in there like for a lot of parent carers would feel like a non-starter, overwhelming, unachievable. They were all sort of day-to-day things that we all need to look at. Yes, I think they absolutely covered the typical things. Not to say that other people don't struggle with them but that parent carers particularly are more likely to struggle with more than the average person. (P6, intervention)

Learning about the CLANGERS and realistic ways to apply them - small steps. Having a laugh. (Weekly feedback, module 2)

Everything today I could relate to, enjoyed the videos, very informative and has made me look at how I can change to help improve my life. (Weekly feedback, module 3)

Looking at achievability of fitting in more exercise / active stuff. Looking for opportunities rather than barriers. (Weekly feedback, module 5)

Really enjoyed doing a 'top tips' exercise. It was a nice reminder of the CLANGERS. Revisiting the 'wheel' to see how far I have come. Very positive for me. (Weekly feedback, module 12)

Having 12 structured sessions made me commit to it and focus on my own health. Having structured, fun, informative, sessions helped a lot - I definitely went through a process of change. (End-of-the-programme feedback)

Loved the CLANGERS definitely made me think about the areas of my own wellbeing I need to work on. (End-of-the-programme feedback)

Reading the information made me think more about things I can do to help myself feel better. I even completed a cross stitch for the learning something new part. The CLANGERS acted as prompts to get me thinking about things I needed to do to give myself some time for myself. (End-of-the-programme feedback)

Being able to reflect on what's working and what isn't by going through the resources and questionnaire in a structured way. (End-of-the-programme feedback)

Being reminded and prompted:
I just am aware of… perhaps more aware of what's wrong, but I don't know that I've made, yes, any great changes to anything. I mean, I went away and said I'd do various things, and some of them I think were okay. I suppose it gives me more of a picture of what doing well might look like. (…) I'd have made them before if I could. I wasn't unaware… (P2, intervention)

It gave me a big kick up the bum to do something, I think that's been helpful. (P13, control)

For peer review only

A lot of it is very common sense stuff… you don't think about or focus on things enough. The CLANGERS, I've discussed that with quite a few people, because it's so simple, isn't it, but it really did help me to focus on yes just all of those things really. (…) It's like a revelation without it being a revelation, if you know what I mean, because you know all that stuff, but you don't take the time to think about it and it was just very much about focusing on us and improving things for ourselves before we can do it for everyone else really. (P3, intervention)

It was all about trying to make sure we reminded ourselves what we already knew but take the time to try and make sure we can sustain ourselves because sometimes you just get really tired, physically and emotionally. (P14, control)

It's not like a six-week programme where you go in not knowing something at the beginning and you come out knowing something at the end or being able to do something at the end that you didn't already know in your heart. It's a six-week programme that concentrates on reminding you of things that you can do and having had that reminder in a concentrated way it's with me all the time. (P6, intervention)

The CLANGERS acted as prompts to get me thinking about things I needed to do to give myself some time for myself. Being able to reflect on what's working and what isn't by going through the resources and questionnaire in a structured way. (End-of-programme feedback)

Reflecting and setting goals:
I think acceptance is a big thing, so accepting what you can't change. And we talked a lot about that on the course, looking at what are our challenges. (…) So it's kind of separating what you can and can't change I think helps you make steps forward, so going, okay, well this is the way it is, I can't do anything about that, so I'm just going to accept it, and now okay what can I change, what do I have control over and then breaking that down again into what are the specific barriers to me making those changes and working on those. (P4, intervention)

I think what causes the stress and anxiety and things like that for me and kind of the downward spiral that you can get into is that feeling that you don't have control, that everything is happening to you and you can't take control of it (…) So you can end up feeling like you don't have control over anything in your life anymore and that's when things start to spiral. So to stop for a minute and go, okay, well let's just think about my health and wellbeing and to break it down in a way that you can go, okay, this is what I know I need to do to be healthy and for my wellbeing for me to carry on doing this, but often everything can feel so overwhelming that you don't know where to start. So to be able to break it down like that and then go, right, I know I need to do this for my wellbeing, what are my barriers, what's stopping me from doing it, and then to analyse that and go, okay, well how can I realistically overcome that to make a positive change gives you… makes you feel like you have more control again. It brings back some control into being able to help yourself, which I think is really important, is having the tools and the skills to be able to help yourself, which is what I felt that course gave me. (P4, intervention)

It taught me how to break down those things into manageable chunks and to know that even a small change is good, that it doesn't… because I think so often we get caught up in the fact that it's all or nothing, right? You have to make that change and you have to do it all the time otherwise it's not worth it, and I think it's recognising that even small changes can make a big difference and being

For peer review only

able to work out how to make those changes, as I said, by breaking it down and looking at what my barriers are. (P4, intervention)

The message was you don't have to make massive changes to improve things. So I'm just trying to, for me and the kids really, tweak things a little bit to try and improve things a bit so it doesn't feel like an onerous task and I don't have to go to a class, I can sort of fit in activity as and when. That's quite reassuring really. So it's about making little changes that you can achieve rather than setting massive goals that you are just going to fail and then feel like a failure. (P3, intervention)

When you do the diary, ticking those and understanding what each planner meant it was suddenly occurring to me that I didn't eat very well, I didn't relax, I didn't sleep, so I needed to focus more on that side of my life. That really helped – the tick chart and setting goals. (…) What might stop you was literally time, so I know I needed to make adjustments. (P10, intervention)

Focusing on oneself:

You know all that stuff, but you don't take the time to think about it and it was just very much about focusing on us and improving things for ourselves before we can do it for everyone else really. (…) it has made me focus much more on my health and wellbeing and thinking I've just got to give myself that time. There's loads of things that I need to do, but at the minute I just need to get better. (P3, intervention

To be honest, it is all basic stuff, isn't it? It is just bringing it to the forefront, that actually, you are a worthy person and it is important that you do something for you, as well as your family and everybody else. (P18, control)

It is about making the carer feel that they are just as important as the caree. Because if we do not focus on ourselves, which is something we learnt, then you cannot work well for the person that you are caring for. (P12, intervention)

I think because of the way it was designed as well, we found out more about the people ourselves. We were ourselves in that. Even though we were carers and we were there because we were carers, we were talking about how we were. (P1, intervention)

Less positive experiences, limited impact:

The thing is, I found it helpful and I know I will continue to find it helpful. I can't say anything has changed as a direct result. Well, maybe some things have changed as a direct result of the programme but because I had an injury and because I'm just tired all the time, it's sort of like well that hasn't really changed [laughs] and it's not going to change. I think it gave me more hope that I will use… It's not like a six-week programme where you go in not knowing something at the beginning and you come out knowing something at the end or being able to do something at the end that you didn't already know in your heart. It's a six-week programme that concentrates on reminding you of things that you can do and having had that reminder in a concentrated way it's with me all the time. So as an example, I'm in a fairly poor state at the moment, not in life, nothing terrible but I'm really tired and struggling with this damn finger and feeling a bit like I'll never get things back to normal, but I know I will and I know what things I'm going to do to do that and the CLANGERS thing just… yes, it just stuck with me and I'm not speaking for other people but I could say that it did for the group, the other participants. It didn't make a massive short-term difference in my life. It did make a massive difference in my

thinking and my awareness and hope that [laughs]… I think it's hope. It gives you hope that you can get back on track no matter what, I'd say. (P6, intervention)

It was interesting, it showed me lots of aspects that I could look at. It wasn't just one thing to change, there were lots of things and one of the videos stuck in my mind. I keep thinking of that, but I haven't actually motivated myself to do anything about that just half an hour of your day. There's like 24 hours in the day and it's just like all we are asking for is half an hour to actually go and do some exercise. So that was the most beneficial that you could do to try and lift your wellbeing and your health and everything, I thought yeah, yeah. I'd got it in my head, but I still hadn't done anything which is a bit… I feel a bit annoyed with myself that I haven't. I thought that in my head all the time why don't I just do that half hour? (P16, control)

I think I probably would have got more if there had been more time for chatting because that was pretty much banned and the time was very limited because of the research element of it, whereas I think in a group like that people would chat normally, so probably the loo break was the best bit in terms of chatting, which sounds weird but it's a bit unnatural to ban chatting. (…) Some of the topics were much heavier than others and bigger than others and that wasn't reflected in the time allocation, so some of it felt like a bit of time filling and all the kind of introductory and end sessions felt a bit time filling when the actual topics really could have done with a lot more depth in some cases. (P2, intervention)

Right at the start was when we did the connecting. Well, it was a big one… I know CLANGERS starts with a C so you start with connect, but connecting is actually a really big one and actually can cause… I know we were talking about connecting with people outside, you are walking the dog, you chat with people you see and say hi, but you can actually come away feeling more lonely than you did when you went out and connected with them. (P1, intervention)

The one I disliked most of all was… It might have been relax. It was a lot to do with managing stress and it was all related to work type stress and very unrelated to the experiences that you have as a parent carer. Yes. Mmm. Once upon a time I had a job and that just pissed me off even more because actually I miss having a job enormously and it's just like [sighs] right, yes, great, yes, people get stressed at work, mmm, that would be nice [laughs]. Just a bit sort of… that one missed the mark quite badly for me but not necessarily for everybody else because it was hitting a sore spot really. (P2, intervention)

A lot of the videos were based on how to eat properly and how the brain works when you have exercise and I think that most people sort of understand these things and how beneficial they are but not everyone has access to them and not everyone can achieve what is put across in the videos and I was sort of more expecting help on… The thing is I think what you can't get away with being a parent carer is it's supposed to be for you as a parent carer but everything that you think about and you do, you think about your children, so you can't really get away from that aspect of it. So maybe if it was done in a way that it was for a parent carer and their children and what they can achieve then it would be different. (P7, intervention)

I must admit when I was watching the 'How to make a box' video, I was thinking, 'This is quite random.' For me, that was right up my street because I love making things like that, but I was thinking, 'If this was my husband watching it, he would probably be watching it thinking "Why am I watching this video?"' (P18, control)

For peer review only

2.3. Views on online materials

Mixed views:

I probably accessed about, well, 7 or 8 of them. Nearly all of them. I don't think I looked at the last one. But it just reinforced what we did in… I didn't do them… Of course, they only came up after, didn't they? I was going to say I didn't do them before because I didn't come up before, that's why. Yes, so I only accessed them after and then I think there was one occasion where the video didn't work or something… there was some reason why we couldn't see it and then I accessed it online. So it was great to have that as a backup if other things didn't quite work out in the session and to reinforce. (P1, intervention)

There's almost always something that I need to get done and if I've got access to a computer I'd rather be doing something more pleasant or relaxing or social than going through an online course on something that isn't central to my interests I guess. So yes, I think that goes back to why I was pleased that I got the real life group because it's like yes I actually will turn up and listen to that, whereas I'm not sure I will actually get round to doing an online course, I'd probably just go yes I just dropped out. (P2, intervention)

Initially I was curious and then I think it dwindled off because I felt like the course was covering everything so thoroughly that would it add anything to what I had already had? So I think that's probably why I didn't bother so much was because, yes, I felt that everything had been covered well during the session. (P4, intervention)

I missed most of [one] session. But then I went online and did it from online. Yes. It was really easy to get into and everything was on there, but I didn't take it in as well as I did on all the other days. It was the Connect and Learn were the two CLANGERS I wasn't there for. All of the other ones I think I got more out of than that one because even though I wrote stuff down from the internet onto the pages she gave me, it didn't sink in as well discussing it. (P5, intervention)

The week that I missed, I went through and used the online materials for that. So yes, a little bit, but I didn't do it systematically alongside the group each week… Once I'm out of the room, group, I've got a hundred million other things that take priority over that. (P6, intervention)

I thought they were really good, the little videos and things like that, I thought it was really helpful information and I think had I have been accessing it every week I would have found it helpful. I had a quick flick through the weeks to just have a quick nosey what it was about at the start. But yes, I do think it was helpful information, but I just didn't follow it through. (P13, control)

I really appreciated the reminders and I really appreciated the way it was laid out in saying what commitments are you going to make today to yourself, to anybody to do these things? But some of the online stuff was very good. (…) Some of the other stuff was just lowbrow and moved on, to be honest. It didn't keep my attention. Making a box, well, get real, come on. (…) So yes, there's some very good video content and yes it fitted together quite well, it's just that there was some demeaning stuff that I didn't do anything with, I just sort of saw it and thought that's not for me, moved it to one side and moved on. (P14, control)

What was there was absolutely fantastic and I think the videos were a good way of doing it because I, for one, am better at learning through watching the videos and being able to go back to them rather than just having loads and loads of information to read because obviously, you do get jaded and you do get tired, so… yeah, I thought it was done really well. (P18, control)

For peer review only

I found some of them were a bit patronising, a bit sort of childish [laughs] and very Americanised. There were like a few which I found more helpful or were quite pleasant to do, but some of the videos I thought were a bit, yeah, a little bit childish. (P17, control)

Lack of groups as impeding the learning:

I always put it off to the next day and the next day, whereas if it was a group I would just go to it as if it was an appointment, and I forget about things all the time otherwise. (P13, control)

I think although it was very useful to have it online, I did like that, but I wish there had been an option to maybe meet up once or twice in the group. I'm the sort of person that when I'm (recording inaudible 0:15:05.2) myself I do like getting out and about and meeting other people who have got similar things and I think it would have been… If you'd have had this group locally, I would have loved to have gone every week and I think that if I'd have gone every week I probably would have finished it no matter what and I think that personally I would have maybe got even more out of it by engaging with other people. (…) Maybe just gaining new friendships, to be able to have that contact with people after the group had finished. Yes, just sharing experiences, sharing tips. Lots of people have different ways of dealing with things and you get ideas from other people or you find out about things to support your children or your family or yourself that maybe you have not heard about. (…) I think it's really useful to be able to have that physical contact with other people really. (…) if you are in a group and you went back the next week and you said, "How did you get on?" it would just encourage you to discuss what you'd done that week and, as I said, maybe learn from other people and just taking it forward, being able to just be perhaps more motivated, I guess. If you are in a group and you see lots of people benefitting it just encourages you. (P15, control)

I was a bit disappointed because I'm in [town] and I thought oh I could do this every week, it will get me out of the house, because that would have given me the incentive to go somewhere and get it and it would have been a walk and some exercise to get there. I was thinking all that in my head, but actually even getting there would be part of it, part of the looking after myself. So I thought online it wasn't going to be as useful. (…) it took a discipline to sit down and actually make that time to look at the second, go back to it. (…) I did forget about it because you've got lots of things to do, whether you had a little reminder or something where you could check in or just say what you… you know what I mean? How did you find doing that part of it or maybe a bit of homework or something [laughs]. You could say, "Oh I didn't manage to do it," but it might be that incentive just to do something, whereas if no one is checking in on you just to say, "How did you find that?" or "Did you do anything?" then you are more likely to not and to put it lower down your list, aren't you? So if I had someone who I thought they are going to ask me what I've done, even like an email, they are going to ask me what I've done, I'll feel like I've got to do it, to try it, yes, but because there was nobody doing that, I didn't push myself. (P16, control)

I found doing the online course quite isolating. Personally, I would have… I think it would have made more changes in a quicker timespan if I had been in the group one. For me, it is not so much… like I said earlier, it is having that group session, it is being with people who are going through very similar things and I think if you have got the pressure, for want of a better word, of a group session, you are more likely to make those changes. Do you know where I am coming from? It is very difficult to put into words. But for me, I have done it online and I have ticked the box, if that makes sense. I have ticked the box. Yeah, I have done it, but it is… I think it is going to be more of a… it is going to be more difficult for me to be able to actually put those things into practice having done it online than if I was going to a course and they said, 'Right. Okay, what have you done for yourself this week?' (…) I think

For peer review only

that whilst it is brilliant to have had access to the course online, I do think it is something that possibly needs to be more personable, more face-to-face rather than a computer screen. Because that does isolate you in yourself. I am taking myself away from my husband and my family to go up and do it, which is great because it is time for me. (…) I do contradict myself, I know, because in one sense, it is easier to do it online because of the timings involved and things like that with work and all the balls that you are juggling, but on the other side, it is harder. It has been harder to do. (…) There was one video that was really quite long and I will not lie, I cannot even remember what it was about now, but it was probably the day I watched it, I had a really difficult emotional day and when I was watching this really long video, I think it was about eleven minutes, and I did find that I was very tired and I did find myself… my mind wandered because you are looking at a computer screen and you are thinking around you, 'I have got to do the washing. I have got to do this.' Because I was in my own environment, I found myself thinking about everything else that I should be doing, whereas if I was in… if I had gone to the group and they were watching that video, you have got nothing else to do, you are switched off. You are switched on to be watching that video, whereas for me, I was there thinking, 'Yeah. Okay, I have got to do this, so I will sit down and do this… and then my mind… like I say, I was… at one point, I was so tired that I was thinking, 'Oh gosh, I have got to go and make lunch, something for [name] and I to have for lunch tomorrow because I have got work, I have got issues at work.' (P18, control)

I found being in the online group really hard as well I could never find time to look at the resources. If I'd have been in the face to face group I'd have had allocated time to get out and access the sessions. (End-of-the-programme feedback)

I wasn't able to attend the groups so looked at the online resources and I don't tend to follow up on things from online. (End-of-the-programme feedback)

2.4. Factors influencing ability to engage with and benefit from the programme

Commitments and inadvertent events:

Just giving myself time to do it really. I didn't prioritise it highly enough; I was just putting other things before it. I knew it was all at the back of my mind and I put a note up for me to look at it, but because there was nobody telling me, I did prioritise other things really. (P16, control)

There were certain things that came up in my life that I hadn't planned, an interview one day and stuff, that it was kind of a big commitment for that sort of six weeks to be able to sort of… if something came up and I was like, oh no, really, and I didn't want to let people down obviously, but there was a few of us that had to take days off here, there and everywhere and maybe it was a bit too long. In hindsight, it probably would have been easier to have done the online stuff. (P7, intervention)

I've had this chest infection, which I missed the last session unfortunately because I was really quite ill and I didn't want to pass it on to other people who are looking after vulnerable people particularly. (P3, intervention)

I think the people who are kind of at the not coping end of the spectrum are likely to not make a lot of the sessions because you have to drop everything all the time. (P2, intervention)

For peer review only

I put it in a sheet, put it in Excel, added a few columns, put in words and scoring and, yes, I'm quite happy for you to see it. It just explains on some days you are impacted by inadvertent events and there is no relaxation, there is five hours' sleep. That's just the way it is. (P14, control)

At the end of a very long day when you are exhausted, you have been to work, you have had a meltdown from an unstable children, you are then getting them to bed, cooking them tea, bla bla bla and then you sit down, you can read something online, but it does not necessarily go in, so it was good to print some bits off, so that I have got that for future. (P18, control)

External factors:

When you have got too much in the way of other things that you are not getting what you need, you are not going to be able to look at it. So yes, I don't know that it felt massively relevant to me because I have got bigger fish to fry. (…) I have some really quite big social care needs and health care needs of my own that are not being met and so it is much more difficult to sit there going, "Ah well, have I eaten properly today or have I…?" That kind of stuff isn't the kind of… But those things don't exist so it's better than nothing. (…) But I think it's working really well for some people. Yes, I can see that other members of the group were getting masses more out of it than I was. So I think if it's the right thing for you then it's clearly way more beneficial. (P2, intervention)

To be honest, the timing of this course could not have been worse [laughter] because my colleague has just been diagnosed with lymphoma, so he has been taken away and he is undergoing really aggressive chemo at the moment, so that means we are a man short in the office and I am thinking, 'Oh my goodness, I have got all this to contend with at the minute,' but that is a parameter that you cannot… as a group, that is not your fault. That is just one of those things. That is not something that you can control. (…) Ironically, I do not know if you have got access to my before and my after, but I actually think my after ones are possibly the answers that are not quite so good in terms of my mental health than the beginning ones but like I say, that is circumstances out of your control because it is nothing to do with reflecting on the course; it is just purely that we have had so much negativity and bad things happening in the last six weeks, that that is possibly reflected in that, but like I say, there is not an awful lot you can do because that is outside of your control. (P18, control)

Unfortunately I have experienced many personal stressful situations since starting the programme which has not helped me to focus on it as much as I had hoped if things had been stable. (End-of-programme feedback)

If there is no respite available and you'll see very little difference as you can't implement what you learn - care is 24-7 in some cases. (End-of-programme feedback)

My son was poorly so I was only able to access so much. (End-of-programme feedback)

For peer review only

Standards for Reporting Qualitative Research (SRQR)

No	Topic	Item	Page & Line #
	**Title and abstract**		
S1	Title	Concise description of the nature and topic of the study Identifying the study as qualitative or indicating the approach (e.g., ethnography, grounded theory) or data collection methods (e.g., interview, focus group) is recommended	p1, line 1
S2	Abstract	Summary of key elements of the study using the abstract format of the intended publication; typically includes background, purpose, methods, results, and conclusions	p3, line 1-25
	**Introduction**		
S3	Problem formulation	Description and significance of the problem/phenomenon studied; review of relevant theory and empirical work; problem statement	p4, line 11-25
S4	Purpose or research question	Purpose of the study and specific objectives or questions	p6, line 1-3
	Methods		
S5	Qualitative approach and research paradigm	Qualitative approach (e.g., ethnography, grounded theory, case study, phenomenology, narrative research) and guiding theory if appropriate; identifying the research paradigm (e.g., postpositivist, constructivist/interpretivist) is also recommended; rationale[b]	p7, line 2-15
S6	Researcher characteristics and reflexivity	Researchers' characteristics that may influence the research, including personal attributes, qualifications/experience, relationship with participants, assumptions, and/or presuppositions; potential or actual interaction between researchers' characteristics and the research questions, approach, methods, results, and/or transferability	p6, line 23-28
S7	Context	Setting/site and salient contextual factors; rationale[b]	Box 3, p21
S8	Sampling strategy	How and why research participants, documents, or events were selected; criteria for deciding when no further sampling was necessary (e.g., sampling saturation); rationale[b]	Box 3, p21
S9	Ethical issues pertaining to human subjects	Documentation of approval by an appropriate ethics review board and participant consent, or explanation for lack thereof; other confidentiality and data security issues	P16, line 7-10
S10	Data collection methods	Types of data collected; details of data collection procedures including (as appropriate) start and stop dates of data collection and analysis, iterative process, triangulation of sources/methods, and modification of procedures in response to evolving study findings; rationale[b]	P6, line 23 to p7, line 19; Box 3, p21
S11	Data collection instruments and technologies	Description of instruments (e.g., interview guides, questionnaires) and devices (e.g., audio recorders) used for data collection; if/how the instrument(s) changed over the course of the study	p6, line 24; Box 3, p21
S12	Units of study	Number and relevant characteristics of participants, documents, or events included in the study; level of participation (could be reported in results)	p7, line 29
S13	Data processing	Methods for processing data prior to and during analysis, including transcription, data entry, data management and security, verification of data integrity, data coding, and anonymization/deidentification of excerpts	p6, line 23
S14	Data analysis	Process by which inferences, themes, etc., were identified and developed, including the researchers involved in data analysis; usually references a specific paradigm or approach; rationale [b]	p7, line 1
S15	Techniques to enhance trustworthiness	Techniques to enhance trustworthiness and credibility of data analysis (e.g., member checking, audit trail, triangulation); rationale[b]	p7, line 11-15
	**Results/findings**		
S16	Synthesis and interpretation	Main findings (e.g., interpretations, inferences, and themes); might include development of a theory or model, or integration with prior research or theory	p8, line 9 - p12, line 9
S17	Links to empirical data	Evidence (e.g., quotes, field notes, text excerpts, photographs) to substantiate analytic findings	Box 4, 5 & supp doc 11

For peer review only - http://bmjopen.bmj.com/site/about/guidelines.xhtml

	**Discussion**		
S18	Integration with prior work, implications, transferability, and contribution(s) to the field	Short summary of main findings; explanation of how findings and conclusions connect to, support, elaborate on, or challenge conclusions of earlier scholarship; discussion of scope of application/ generalizability; identification of unique contribution(s) to scholarship in a discipline or field	p13, line 28
S19	Limitations	Trustworthiness and limitations of findings	p4, line 1
	**Other**		
S20	Conflicts of interest	Potential sources of influence or perceived influence on study conduct and conclusions; how these were managed	p16, line 12
S21	Funding	Sources of funding and other support; role of funders in data collection, interpretation, and reporting	p15, line 25

O'Brien BC, Harris IB, Beckman TJ, Reed DA, Cook DA. Standards for reporting qualitative research: a synthesis of recommendations. Acad Med 2014 Sep;89(9):1245-51.

[a]The authors created the SRQR by searching the literature to identify guidelines, reporting standards, and critical appraisal criteria for qualitative research; reviewing the reference lists of retrieved sources; and contacting experts to gain feedback. The SRQR aims to improve the transparency of all aspects of qualitative research by providing clear standards for reporting qualitative research. [b]The rationale should briefly discuss the justification for choosing that theory, approach, method, or technique rather than other options available, the assumptions and limitations implicit in those choices, and how those choices influence study conclusions and transferability. As appropriate, the rationale for several items might be discussed together.

For peer review only

BMJ Open

The Healthy Parent Carers Programme: mixed-methods process evaluation and refinement of a health promotion intervention

Journal:	*BMJ Open*
Manuscript ID	bmjopen-2020-045570.R2
Article Type:	Original research
Date Submitted by the Author:	04-Aug-2021
Complete List of Authors:	Lloyd, Jenny; University of Exeter, NIHR Applied Research Collaboration South West Peninsula (PenARC), University of Exeter Medical School ; University of Exeter, Relational Health Group, Institute of Health Research, University of Exeter Medical School<br>Bjornstad, Gretchen; University of Exeter, Peninsula Childhood Disability Research Unit (PenCRU), University of Exeter Medical School; University of Exeter, NIHR Applied Research Collaboration South West Peninsula (PenARC), University of Exeter Medical School<br>Borek , Aleksandra ; Oxford University, Nuffield Department of Primary Care Health Sciences, Medical Sciences Division<br>Cuffe-Fuller, Beth; University of Exeter, Peninsula Childhood Disability Research Unit (PenCRU), University of Exeter Medical School<br>Fredlund, Mary; University of Exeter, Peninsula Childhood Disability Research Unit (PenCRU), University of Exeter Medical School; University of Exeter, NIHR Applied Research Collaboration South West Peninsula (PenARC), University of Exeter Medical School<br>McDonald, Annabel; University of Exeter, Peninsula Childhood Disability Research Unit (PenCRU), University of Exeter Medical School<br>Tarrant, Mark; University of Exeter, NIHR Applied Research Collaboration South West Peninsula (PenARC), University of Exeter Medical School<br>Berry, Vashti; University of Exeter, NIHR Applied Research Collaboration South West Peninsula (PenARC), University of Exeter Medical School<br>Wilkinson, Kath; University of Exeter, Peninsula Childhood Disability Research Unit (PenCRU), University of Exeter Medical School<br>Mitchell, Siobhan; University of Exeter, NIHR Applied Research Collaboration South West Peninsula (PenARC), University of Exeter Medical School<br>Gillett, Annette; University of Exeter, Peninsula Childhood Disability Research Unit (PenCRU), University of Exeter Medical School<br>Logan, Stuart; University of Exeter, Peninsula Childhood Disability Research Unit (PenCRU), University of Exeter Medical School ; University of Exeter, NIHR Applied Research Collaboration South West Peninsula (PenARC), University of Exeter Medical School<br>Morris, Christopher; University of Exeter, Peninsula Childhood Disability Research Unit (PenCRU), University of Exeter Medical School; University of Exeter, NIHR Applied Research Collaboration South West Peninsula (PenARC), University of Exeter Medical School
Primary Subject	Health services research

Heading:	
Secondary Subject Heading:	Research methods
Keywords:	QUALITATIVE RESEARCH, STATISTICS & RESEARCH METHODS, PREVENTIVE MEDICINE

SCHOLARONE™ Manuscripts

I, the Submitting Author has the right to grant and does grant on behalf of all authors of the Work (as defined in the below author licence), an exclusive licence and/or a non-exclusive licence for contributions from authors who are: i) UK Crown employees; ii) where BMJ has agreed a CC-BY licence shall apply, and/or iii) in accordance with the terms applicable for US Federal Government officers or employees acting as part of their official duties; on a worldwide, perpetual, irrevocable, royalty-free basis to BMJ Publishing Group Ltd ("BMJ") its licensees and where the relevant Journal is co-owned by BMJ to the co-owners of the Journal, to publish the Work in this journal and any other BMJ products and to exploit all rights, as set out in our licence.

The Submitting Author accepts and understands that any supply made under these terms is made by BMJ to the Submitting Author unless you are acting as an employee on behalf of your employer or a postgraduate student of an affiliated institution which is paying any applicable article publishing charge ("APC") for Open Access articles. Where the Submitting Author wishes to make the Work available on an Open Access basis (and intends to pay the relevant APC), the terms of reuse of such Open Access shall be governed by a Creative Commons licence – details of these licences and which Creative Commons licence will apply to this Work are set out in our licence referred to above.

Other than as permitted in any relevant BMJ Author's Self Archiving Policies, I confirm this Work has not been accepted for publication elsewhere, is not being considered for publication elsewhere and does not duplicate material already published. I confirm all authors consent to publication of this Work and authorise the granting of this licence.

For peer review only

The Healthy Parent Carers Programme: mixed-methods process evaluation and refinement of a health promotion intervention

Lloyd J, Bjornstad G, Borek A, Cuffe-Fuller B, Fredlund M, McDonald A, Tarrant M, Berry V, Wilkinson K, Mitchell S, Gillett A, Logan S, Morris C

Jenny Lloyd 01392 722972 (Corresponding author)
J.J.Lloyd@exeter.ac.uk
NIHR Applied Research Collaboration South West Peninsula (PenARC) and Relational Health Group, University of Exeter Medical School, University of Exeter, St Luke's Campus, Heavitree Road, Exeter, EX1 2LU, UK

Gretchen Bjornstad
g.j.bjornstad@exeter.ac.uk
Peninsula Childhood Disability Research Unit (PenCRU) and NIHR Applied Research Collaboration South West Peninsula (PenARC), University of Exeter Medical School, University of Exeter, St. Luke's Campus, Heavitree Road, Exeter, EX1 2LU, UK

Aleksandra Borek
aleksandra.borek@phc.ox.ac.uk
Nuffield Department of Primary Care Health Sciences, Medical Sciences Division, University of Oxford, Radcliffe Observatory Quarter, Woodstock Road, Oxford, OX2 6GG, UK

Beth Cuffe-Fuller
bethany.cuffefuller@nhs.net
Peninsula Childhood Disability Research Unit (PenCRU), University of Exeter Medical School, University of Exeter, St. Luke's Campus, Heavitree Road, Exeter, EX1 2LU, UK

Mary Fredlund
maf215@exeter.ac.uk
Peninsula Childhood Disability Research Unit (PenCRU) and NIHR Applied Research Collaboration South West Peninsula (PenARC), University of Exeter Medical School, University of Exeter, St. Luke's Campus, Heavitree Road, Exeter, EX1 2LU, UK

Annabel McDonald
a.l.mcdonald@exeter.ac.uk
Peninsula Childhood Disability Research Unit (PenCRU), University of Exeter Medical School, University of Exeter, St. Luke's Campus, Heavitree Road, Exeter, EX1 2LU, UK

Mark Tarrant
m.tarrant@exeter.ac.uk
NIHR Applied Research Collaboration South West Peninsula (PenARC), University of Exeter Medical School, University of Exeter, St Luke's Campus, Heavitree Road, Exeter, EX1 2LU, UK

Vashti Berry
V.Berry@exeter.ac.uk
NIHR Applied Research Collaboration South West Peninsula (PenARC), University of Exeter Medical School, University of Exeter, St Luke's Campus, Heavitree Road, Exeter, EX1 2LU, UK

For peer review only

For peer review only - http://bmjopen.bmj.com/site/about/guidelines.xhtml

Kath Wilkinson
K.Wilkinson2@exeter.ac.uk
Peninsula Childhood Disability Research Unit (PenCRU), University of Exeter Medical School, University of Exeter, St. Luke's Campus, Heavitree Road, Exeter, EX1 2LU, UK

Siobhan Mitchell
s.b.mitchell@exeter.ac.uk
NIHR Applied Research Collaboration South West Peninsula (PenARC), University of Exeter Medical School, University of Exeter, St. Luke's Campus, Heavitree Road, Exeter, EX1 2LU, UK

Annette Gillett
A.Gillett@exeter.ac.uk
Peninsula Childhood Disability Research Unit (PenCRU), University of Exeter Medical School, University of Exeter, St. Luke's Campus, Heavitree Road, Exeter, EX1 2LU, UK

Stuart Logan
Stuart.Logan@exeter.ac.uk
Peninsula Childhood Disability Research Unit (PenCRU) and NIHR Applied Research Collaboration South West Peninsula (PenARC), University of Exeter Medical School, University of Exeter, St. Luke's Campus, Heavitree Road, Exeter, EX1 2LU, UK

Christopher Morris
christopher.morris@exeter.ac.uk
Peninsula Childhood Disability Research Unit (PenCRU) and NIHR Applied Research Collaboration South West Peninsula (PenARC), University of Exeter Medical School, University of Exeter, St. Luke's Campus, Heavitree Road, Exeter, EX1 2LU, UK

For peer review only

For peer review only - http://bmjopen.bmj.com/site/about/guidelines.xhtml

Abstract

Objectives: Parent carers of children with special educational needs or disability are at risk of poorer mental and physical health. In response to these needs, we co-developed the 'Healthy Parent Carers' (HPC) programme. This study examined views and experiences of participants in the HPC feasibility trial to inform programme refinement.

Intervention, setting and participants: HPC is a peer-led group-based intervention (supported by online materials) for primary carers of disabled children, encouraging behaviours linked with health and wellbeing. It was delivered by two lead and six assistant peer facilitators, in six community sites (one lead and one assistant per group), in South West England over six or twelve sessions. Control participants had online materials only. The trial involved 47 intervention and 45 control parent carers (97% female and 97% white) and eight facilitators (1 male).

Design: A pre-planned mixed methods process evaluation using questionnaires and checklists (during and after the intervention), qualitative interviews with participants post intervention (n=18) and a focus group with facilitators post trial.

Results: HPC was highly acceptable to participants and facilitators and experiences were very positive. Participants reported that the programme increased awareness of what parent carers could and could not change and their self-efficacy to engage in health-promoting behaviours. The intended mechanisms of action (social identification and peer support) matched participants' expectations and experiences. Control participants found the online-only programme flexible but isolating, as there were no opportunities to share ideas and problem-solve with peers, the key function of the programme. Areas for improvement were identified for programme content, facilitator training and delivery.

Conclusion: HPC was acceptable, well-received and offers considerable potential to improve the health of parent carers. Under the pandemic, the challenge going forward is how best to maintain reach and fidelity to function whilst delivering a more virtual programme.

Trial registration: ISRCTN151144652

For peer review only

For peer review only - http://bmjopen.bmj.com/site/about/guidelines.xhtml

Strengths and Limitations of this study

- The HPC programme has followed the key principles of intervention development
- Qualitative and quantitative data have been synthesised systematically to refine and optimise the intervention
- The intervention refinement process is transparent, and adaptations reflect the process data collected
- Key uncertainties to be addressed in future research have been identified
- Experiences and views are from a predominantly white population, therefore, are not necessarily representative of ethnically/culturally and linguistically diverse parent carers.

Introduction

Parent carers of children with special educational needs or disability are at increased risk of poorer mental [1-10] and physical health,[2, 3, 6, 7, 11-13] a problem recognised in the NHS Long Term Plan as requiring action to support the personal needs of carers.[14] Parent carers experience challenges to maintaining good health that have implications for their wellbeing and their ability to care for their children and recent reviews conclude that there are insufficient programmes that aim to support parental health, which are likely to be the best strategy to advance both child and family outcomes.[15, 16] In response to this need we co-developed the 'Healthy Parent Carers' (HPC) programme, a community based behaviour change approach to improve health and wellbeing, advocated by Public Health England.[17, 18] This health promotion intervention targets specific behaviours based around a set of universal and evidence-based actions (called CLANGERS) associated with health and wellbeing. CLANGERS stands for Connect, Learn, be Active, take Notice, Give, Eat well, Relax and Sleep.[19] The 'CLANG' component comprises the 'Five Ways to Wellbeing' based on the evidence from the Foresight Project on Mental Capital and Wellbeing.[20] Each of these behaviours is potentially more difficult for parent carers to sustain because of the demands and disruptions of their caring role. The programme involves a range of activities to improve parent carer confidence, motivation and self-efficacy to plan, prioritise and enact these universal actions to improve their own health and wellbeing, whilst expanding their social network and providing peer-to-peer social support.

Intervention Mapping [21] with extensive stakeholder involvement was used to develop programme content and delivery strategies (supplementary documents 1a and 1b) which was piloted with one group of seven parent carers, delivered by two peer facilitators (MF/BM), with whom the intervention was co-created. The findings of this proof-of-concept study and details of the

For peer review only - http://bmjopen.bmj.com/site/about/guidelines.xhtml

intervention development, logic model and content were published previously.[22] Box 1 summarises programme content, format, and delivery.

Insert Box 1

The Feasibility Study

The feasibility study aimed to assess whether the programme could be delivered in the community and evaluated the acceptability of a randomised controlled trial (RCT) design.[23] The trial ran between July 2018 and June 2020. Ninety-two participants were randomised: 47 to the HPC group programme (delivered in 6 community sites across Cornwall, Devon and Somerset) and 45 to the control group, which involved access to the HPC online resources only. The group sessions ran between January and July 2019. Outcome measures were collected at baseline (prior to randomisation), immediately post intervention and six months later (supplementary document 2).

HPC group sessions were delivered by pairs of peer Lead and Assistant Facilitators who were themselves parent carers of children with chronic health conditions. Two Lead Facilitators, experienced in delivering group training to parent carers, were recruited from the Council of Disabled Children (CDC). Nine Assistant Facilitators (including three reserves) were recruited through recommendations from our Stakeholder Advisory Group and through local network adverts. Volunteers were shortlisted and interviewed by telephone. Selection decisions were made based on a practical understanding of the challenges faced by parent carers in relation to their own health and wellbeing with reference to our person specification, and availability to deliver at one of the selected venues. Details of the training programme are in Box 2.

One of the Lead Facilitators delivered the six-week programme to two groups (two sites) and one delivered to four groups (four sites), one of which followed the 12-session format (Box 1). Each Assistant Facilitator was assigned to support delivery in one of the six groups.

Insert Box 2

Figure 1 shows the flow of participants in the trial and details of data collection.

Insert Fig 1

For peer review only - http://bmjopen.bmj.com/site/about/guidelines.xhtml

The aim of this paper is to present mixed methods process data on the motivations of parent carer participants and facilitators to take part in the HPC programme, their views and experiences of receiving/delivering the programme and its refinement in response to these data. Trial findings related to recruitment and retention, fidelity of intervention delivery, the feasibility and acceptability of trial processes and outcome data will be reported separately.[24]

Methods

Patient and Public Involvement

Parent carers have been involved in all stages of developing the intervention, designing the feasibility study and refining the programme in light of the findings from this feasibility trial, through the involvement of the Peninsula Childhood Disability Research Unit (PenCRU) Family Faculty (www.pencru.org/getinvolved/ourfamilyfaculty). The Family Faculty are parent carers who are offered opportunities to be involved in research.

Participants

Recruitment of participants and facilitators to the trial is reported above. For quantitative process evaluation data, all trial participants in both arms were sent online feedback forms after the programme, and all group participants were asked to complete feedback forms after each session. For qualitative process evaluation data collection, participants were purposively sampled for maximum variation to include male and female perspectives, a range of engagement levels, and parent carer challenges (see Box 3), and all facilitators involved in delivering the programme were invited to a focus group.

Data Collection

Qualitative and quantitative data were collected from participants in both arms and facilitators on programme uptake, reach, training, delivery, and experience as well as suggestions for improvement. Researchers (BCF, JL) who were not involved in programme delivery collected qualitative data. Box 3 presents the measures, (details in supplementary documents 3-10) and sampling process.

Insert Box 3

For peer review only - http://bmjopen.bmj.com/site/about/guidelines.xhtml

Data Analysis

Qualitative data

All qualitative data was uploaded to NVivo (version 12). Taking both an inductive and deductive thematic approach to analysis, we developed a coding framework to categorise the data from participant interviews, facilitator focus group, facilitator support calls, and free-text data from the participant and facilitator questionnaires. Three parent interviews were coded independently by three researchers (BCF, AB, JL) initially and compared to agree the coding framework, which included combining codes and arranging them into higher-level categories to organise the data. While the detailed codes were developed inductively, the categories were more deductive and followed the key areas of interest in the process evaluation, reflecting the interview topic guide. The agreed framework was then used to code the remaining interviews, adding new inductive codes when identified. Facilitator support calls, and free-text data from the participant and facilitator questionnaires were coded by AG and BCF, and 20% were double coded by SM. All interview transcripts (n=18) were coded by BCF, and 50% (n=9) were double-coded by AB and JL. The focus group transcript was coded by BCF and checked by JL and AB. The double-coding and cross-checking of the coding, and regular team discussions on analysis and interpretation helped ensure the quality of analysis and minimise any potential researcher bias. Findings from the parent interviews were triangulated with data arising from the focus group and the questionnaires.

Quantitative data

Relevant quantitative questionnaire data in relation to participant and facilitator experiences and views were collated and presented descriptively alongside the qualitative findings.

Data Synthesis for Intervention Refinement

Data relevant to programme improvement (i.e. suggestions, critical comments, negative experiences) were extracted from NVivo. These data were coded and sorted into three categories: facilitator training, intervention delivery, and intervention content (including online materials). For each suggestion/comment, the source of data was noted, including when there were mixed or contradictory views. Figure 2 shows the refinement process. Potential refinements were discussed and prioritised using a colour-coded system.

Insert Fig 2

For peer review only - http://bmjopen.bmj.com/site/about/guidelines.xhtml

Findings

Trial participants were aged 42.5 (8.0) years (mean (SD)), with 96% female and 97% white. Figure 1 shows the number of participants who provided each type of process evaluation data. Completeness of questionnaire data was high for participants (>80%) and facilitators (100%). According to facilitators' self-report delivery checklists, 90% of activities were delivered across all groups. Scores from researcher checklists of the nine audio-recorded modules [24] similarly indicated that 91% of activities were delivered. Interviews were carried out with twelve intervention (two per group/site; 11 female, 1 male) and six control participants (one per site; 5 female, 1 male). The focus group involved all lead (n=2 females) and assistant (n=6; 5 females, 1 male) facilitators. The mixed-methods findings are summarised below, with illustrative quotes reported in boxes 4 and 5 and additional quotes and comments in supplementary document 11.

1. Facilitator experiences

1.1. Motivations and expectations

Five out of eight facilitators had experience of facilitating parent carer groups. Most reported being interested in delivering the programme because it fitted with their skills and interests, and offered an opportunity to further develop their professional skills and confidence. Facilitators recognised the need for, and importance of, health and wellbeing training for parent carers, believed in the value of the programme (and group support), and wanted to support other parent carers. All reported interest in contributing to the research and programme development, and five hoped to benefit from the programme themselves.

1.2. Training, preparation and support

Following block 1, 2 and 3 of training (Box 2), all Lead and Assistant Facilitators completed the facilitator training feedback questionnaire. Overall scores were out of 5. Knowledge/understanding and skills/confidence for each block were high at 4.5 and 4.6; 4.5 and 4.3; 4.6 and 4.45, respectively. The only component to decrease over the training was the facilitator's skills and confidence to manage difficult/sensitive issues. Thus, unsurprisingly, in the focus group following programme delivery, they suggested the training could focus more on the delivery style and practice, and managing difficult issues specific to parent carers likely to arise in group discussions. Facilitators suggested that future facilitators could be recruited from those completing the programme, so facilitators would possess a greater understanding of the content and process and be able to build on their own experience of the programme. They also suggested better clarifying the roles and responsibilities of Lead and Assistant Facilitators, and to be matched and trained together. They reported satisfactory support throughout programme delivery (i.e. supervision from CDC, support

For peer review only - http://bmjopen.bmj.com/site/about/guidelines.xhtml

For peer review only

calls with the research team), but particularly valued the sharing and support among fellow facilitators. Nearly three quarters of facilitators believed that sufficient time had been allocated to training.

1.3. Programme content and group delivery

Facilitators perceived the following benefits of the programme for participants: the focus on parent health/wellbeing (rather than their children); the face-to-face group delivery (rather than online); the consistent and predictable session structure; the weekly goal setting (but suggested simplifying 'SMART' to 'achievable' goals). They perceived both 6- and 12-week delivery as suitable.

Facilitators found the practical activities an important component (in addition to group discussions) but some activities triggered difficult feelings among some participants. The facilitators reported that some participants disclosed difficult personal issues, which were challenging to address in the group without turning into counselling sessions; thus, the facilitators stressed the importance of training in managing such situations. This was more problematic when groups were small, and the facilitators agreed that 6-8 participants was an optimal group size. The facilitators emphasised the importance of facilitators being parent carers, and how the positive experiences of co-facilitating and the programme helped them reflect on their own health and wellbeing.

Facilitators thought that each session went well, and that participant engagement was high, their average ratings across groups for each session ranging between 4.4 and 4.8 out of 5 for how well they thought the session went and 4.5-5 out of 5 for parent engagement. Facilitator judgement of how connected their groups were following each session was very high and increased over the course of the programme from 5/7 to 7/7. Box 4 presents selected quotes illustrating facilitators' experiences.

Insert Box 4

2. Participants' experiences

Below we refer to 'interviewees' to indicate the views only of those interviewed and to 'participants' to indicate data collected from the questionnaires. Box 5 presents selected quotes illustrating participants' experiences.

2.1. Motivation to participate and expectations

All interviewees (n=18) reported participating in the programme to focus on and find ways to improve their health and wellbeing. Some wanted to do that to better support their families, and to feel less guilt for taking time to take care of themselves. A few interviewees thought the programme

For peer review only - http://bmjopen.bmj.com/site/about/guidelines.xhtml

took place at the right time for them: when they were able, ready and available to participate and focus on their health. Most interviewees also reported wanting to contribute to research and help other parent carers, especially as they perceived little available support for parent carers.

The majority of interviewees in the group programme reported positive aspects of face-to-face delivery, such as peer support and dedicated time to share and discuss issues affecting their wellbeing. A few participants, mainly from the online-only control arm, reported that the flexibility of the online programme enabled them to engage with the programme more easily than attending face-to-face groups. Overall, group-based delivery was preferred, including those who had not experienced it, as groups were perceived to instigate a stronger commitment to engage with the programme.

2.2. Experiences of the group programme and its impact

Positive experiences: Overall 84% (37/44) of participants were satisfied or very satisfied with the group programme, with 67% (28/42) finding it useful in helping them to improve their health and wellbeing. Most of the interviewees echoed this, noting the positive impact of the programme on them and their families. Two reported that it 'pushed them out of their comfort zones' by addressing some uncomfortable but important issues. Several participants commented that the programme should be rolled-out to benefit all parent carers.

Groups: All participants who responded to the post intervention questionnaire reported feeling included and part or very much 'included and part of the group' with 85% (17/20) indicating that session length was 'about right'. All intervention interviewees described one of the main benefits of the programme as having the opportunity to discuss and share with other parent carers in a supportive, empathetic, safe, and respectful group context. They highlighted the value of peer support and discussions in motivating change. Interviewees also perceived the group programme as providing more than support groups because of the practical activities and exploring barriers and solutions in specific areas. Some were surprised by the small group size but liked it as it enabled them to participate and get to know each other and bond as a group; yet, most thought that slightly larger groups would provide more varied perspectives. Interviewees noted also that the group programme gave them time to focus on themselves and permission (to take time) to take care of themselves.

Peer facilitators: All participants who responded to the questionnaire were satisfied or very satisfied with the way the programme was delivered, and interviewees highlighted the critical importance of the facilitators being parent carers, enabling shared understanding and empathy. Participants valued

For peer review only - http://bmjopen.bmj.com/site/about/guidelines.xhtml

facilitators delivering the programme together ('complementing each other' and 'bouncing off each other'), facilitating learning through group discussions (rather than 'teaching'), creating positive group context (with facilitators seen as part of the groups), and being knowledgeable, understanding and kind.

Content and activities: Interviewees generally had positive comments about programme content and preferences for different activities. The content and activities were mainly valued for providing structure and prompts to group discussions and focus on different life areas. The promoted messages were seen as 'common sense' and applicable to all people, but more challenging to parent carers. The programme reminded participants about the importance of health/wellbeing, and discussing/sharing reinforced that, thus prompting them to make changes.

Reflecting and setting small achievable goals: Interviewees valued the programme's focus on simple and meaningful actions (CLANGERS) that increased their awareness of areas of their lives in which they could make positive changes (while also helping accept things that they could not change). Interviewees also valued focusing on small steps that they can take, setting achievable goals at each session/module, and then reflecting on them. This helped them feel more in control of doing something positive about their health/wellbeing. 76% (35/46) reported making changes.

Less positive experiences: Despite overwhelmingly positive experiences, nine participants did not find the programme useful, with five indicating ambivalence. A few interviewees reported a less positive experience and *not* making changes, mainly due to factors outside the programme (described below); however, they still valued the raised awareness and 'hope' it provided. This was reflected in the quantitative feedback with 24% (11/46) reporting not making changes. A few interviewees reported feeling apprehensive and nervous at the start of groups; finding different activities or content difficult, challenging or less appealing; feeling uncomfortable with others in the group (e.g., due to expressed views/comments); perceiving less shared experience and challenges with others in the group (e.g., due to personal or system-related contexts).

2.3. Views on online materials

Participants' experiences of, and views on, using online materials were mixed. Interviewees attending the groups reported finding the online materials unnecessary or just reinforcing the sessions. Interviewees from the control group reported some positive experiences of the online-only programme (valuing videos as an engaging way of providing information) but found some content less relevant or helpful. Overall satisfaction of control participants with the programme was good, although lower than intervention participants (66% vs 84%), with only 38% (11/29) finding the programme 'useful in helping them to improve their health and wellbeing', although 65% (20/31)

For peer review only - http://bmjopen.bmj.com/site/about/guidelines.xhtml

reported making changes.

Interviewees in both study arms described the importance of group discussions in enhancing the learning and programme impact. Without group sessions, some found the online-only programme isolating (without opportunities to discuss, share ideas and problem-solve with other parent carers) and harder to focus on because of lacking reminders and scheduled time (with other matters taking priority).

2.4. Factors influencing ability to engage with and benefit from the programme

Interviewees in both study arms reported factors that affected their ability to attend the sessions or use online materials, including: other (unexpected) commitments, lack of time, inadvertent events (their or children's illness), access/transport, and childcare. A few also discussed external factors out of their control (e.g., social care needs, access to respite, work situation) that affected their wellbeing and ability to benefit from the programme, reinforcing the importance of participating in the programme at the right time in their lives.

Insert Box 5

3. Programme refinement

The process evaluation confirmed that the HPC programme was highly acceptable to both parent carers and facilitators with the vast majority expressing positive experiences; however less positive experiences and suggestions were carefully considered and used in the refinement process (Figure 2). Several potential areas for improvement were identified and, where possible, incorporated into the refined HPC programme. Key changes are outlined below.

3.1. Optimising session and online content

- Wording of the online materials simplified to increase understanding, engagement, and usage.
- Following suggestions and less positive views on goal setting, 'SMART' replaced with 'achievable' goals, and examples of SMART goals were added to online materials and delivery manual to increase understanding.
- Create or select videos more relevant to parent carers (as some participants were less positive about some of the more generic health-related videos).

3.2. Optimising training

- Explanation of the rationale for certain activities included to increase understanding of their purpose.
- Training in managing challenges that specific content/activities may generate, and worked examples on how these may be addressed.
- Training in how best to present and deliver the videos to elicit discussion around the key 'take away' messages.

For peer review only - http://bmjopen.bmj.com/site/about/guidelines.xhtml

- Facilitators to complete the online modules prior to training so that that they are familiar with the CLANGERS and resources, providing more time to focus on delivery strategies.
- Group dynamics session refined so that it is more practical and interactive (scenarios and practice included in how to build, enhance and maintain group cohesion under challenging situations; how to find commonalities in shared experience despite having differing challenges/situations).
- Delivery process for each of the CLANGERS to be modelled in detail using 'Connect' (as it is the most challenging of the CLANGERS to deliver and experience) to ensure that facilitators understand the application of the programme's theory of change.
- Trainers asked to create and disseminate a FAQ document to follow up any questions/concerns not addressed in the training due to time constraints.
- Increased focus on developing delivery skills using modelling techniques to improve confidence and quality of delivery (e.g. modelling good responses to parent questions, supporting parents who are struggling).
- Roles and responsibilities of the Lead and Assistant Facilitators clearly explained to avoid misunderstanding and enhance team working.

3.3. Optimising delivery

- Lead and Assistant Facilitators to communicate with each other prior to the first session (using the 'preparation' checklist) to allay any delivery fears/concerns.
- Prior to session 1, to allay any participant concerns before joining the group, facilitator photo, with a written introduction to be sent to participants; Lead Facilitators to call participants to introduce themselves, ascertain any practical support required and take steps to provide this support where possible.
- Advice on managing difficult situations added to the delivery manual.
- Description of roles and responsibilities of the Lead and Assistant Facilitators added to the delivery manual.
- Key 'take away' messages from each video added to the delivery manual.
- Delivery manual adapted to accommodate the 12x2 hour format to improve coherence.
- Completion of a simple participant feedback form at the end to provide information to improve future group delivery.
- Following participants' and facilitators' less positive comments about smaller groups, change minimum viable group number from 4 to 6 to increase the range of perspectives and create better conditions for peer-to-peer learning and support.

Discussion

This paper presents facilitator and parent carers' views on and experiences of the HPC programme from the feasibility RCT, which have informed the refinement of the programme. We are now exploring implementation uncertainties, funded by the Economic and Social Research Council Impact Accelerator Account Strategic Initiative Award (ES/T501906/1) in preparation for a further evaluation of programme effectiveness. Fidelity to delivery and qualitative data show that the training of facilitators was successful. The group programme was valued for providing peer support and practical activities, where difficult and emotional conversations are facilitated and explored. This is a key function of the programme, which accounted for the high levels of satisfaction and reported

For peer review only - http://bmjopen.bmj.com/site/about/guidelines.xhtml

impact on both the parent and the wider family. It appears that this aspect of the programme necessitates larger groups (6-12) to allow for varied perspectives and a ‘facilitation’ rather than ‘counselling’ approach. Group size, therefore, will be explored as part of intervention fidelity to function [25, 26] in the definitive trial.

As intended, consistent with our logic model, participants reported that the programme increased awareness of what parent carers could and could not change and their self-efficacy to engage in health-promoting behaviours (CLANGERS). Participant motivations and expectations showed that, overall, the target group was reached (i.e. those who wanted to and reported feeling ready to do something to improve their own health/wellbeing), with the intended mechanisms of action (social identification and peer support) matching the participants’ expectations and experiences.

Facilitators reported that the relationship between the Leads and Assistants was important for effective delivery and that clarity on and practice of these different roles were a crucial part of training and quality delivery. Both intervention participants and facilitators thought that, to deliver the programme effectively, Leads and Assistants needed to have completed the programme themselves and that the development of a network of facilitators to share experiences of delivery using support calls and/or online meetings was important in supporting them to improve their practice.

The strength of this research is that it has systematically followed key principles of intervention development [27, 28] and refinement, using a dynamic, iterative and creative process with extensive stakeholder consultation, where the developers have been open to change based on data collected in a series of iterations.[22, 23] We examined how the intervention will be evaluated in the next phase of research and identified learning and key uncertainties to be addressed, such as blended (online and face-to-face) delivery of group sessions, commissioning and implementation. Reporting mixed methods data on participant views and experiences and the subsequent refinement process in feasibility studies is recommended.[29] Not only does it increase knowledge about the intervention refinement/optimisation process but it also allows others to link up intervention development processes and subsequent trial outcomes.

However, the study has limitations. The lack of ethnic diversity in South West England, where this study was conducted, coupled with the low representation of men as ‘primary care givers’ (an inclusion criterion) meant that the sample was gender and ethnically homogeneous. Experiences and views, therefore, may not represent fathers or parent carers from different cultures and contexts.

For peer review only - http://bmjopen.bmj.com/site/about/guidelines.xhtml

Most work on culturally and linguistically diverse groups and parenting interventions has been conducted in the USA and is equivocal about whether outcomes differ by ethnicity.[30, 31] However, adapting interventions for different ethnic groups poses many issues. Adaptation may neither be practical or a desirable service model for multi-ethnic European cities.[32] Presently, we do not have the available data as individual trials are not powered to test intervention effects by ethnicity.

Nevertheless, we will explore in future evaluative work with mixed and single ethnic groups, how ethnicity, social disadvantage, gender and/or other personal factors might intersect to exacerbate the health issues arising from being a parent carer,[33] and affect the development of a shared group identity and implementation more generally.

This study has helped to refine the programme in many aspects; however, there remain a number of barriers to parent carers' capacity to engage in all group sessions and benefit from the programme. We need to explore these barriers in further detail and the extent to which they can be mitigated to enhance accessibility to participate in the programme. As we write, the COVID-19 pandemic has meant social distancing and increased challenges to running groups. We are therefore keen to explore to what extent the group programme could be delivered virtually using videoconferencing, which may overcome some other barriers to participation, but we would need to evaluate the extent to which peer support and cohesive support of the group is maintained.

Conclusion

The format, content and delivery of the HPC programme was highly acceptable to participants and for facilitators to deliver. The process evaluation data enabled programme refinement to optimise impact going forward. Although the programme focuses on promoting health and wellbeing at an individual level (i.e. individual psychological and behavioural change), we acknowledge the importance of other factors at interpersonal, community and societal levels that affect parent carers' health and wellbeing, such as access to services, negative public attitudes towards disability, which in turn impact on parent carers' capacity to make and sustain changes. The programme does, however, provide support and hope for those who find it difficult, both practically and psychologically, to attend to their own wellbeing. The challenge we face going forward is delivery of the programme in the shadow of COVID-19. The team plan to explore how this might be reimagined to accommodate a new way of supporting parents, minimising risk to health, whilst delivering an accessible and inclusive package that maintains fidelity to function.

(4533 word count)

For peer review only - http://bmjopen.bmj.com/site/about/guidelines.xhtml

Declarations

Funding

This paper presents independent research funded by the National Institute for Health Research (NIHR) under its Research for Patient Benefit (RfPB) Programme (Grant Reference Number PB-PG-0317-20044). We also acknowledge support from the National Institute for Health Research Applied Research Collaboration South West Peninsula. The views and opinions expressed in this paper are those of the authors and not necessarily those of the National Institute for Health Research or the Department of Health and Social Care. Delivery of the Healthy Parent Carers programme was funded by a grant from the National Lottery Community Fund through the Reaching Communities programme (Grant number 10343962) and supported by the Public Health England, Kernow Clinical Commissioning Group, Northern, Eastern and Western Devon Clinical Commissioning Group. Peer review of the study design was provided during the NIHR funding application process. The funders have had no involvement in the writing of the manuscript and will not have any role during its execution, analyses, interpretation of the data, writing of the report, or the decision to submit the report for publication.

Ethics approval

The study procedures were approved by University of Exeter Medical School Research Ethics Committee in the College of Medicine and Health (UEMS REC 18/06/174). All participants in the study provided written consent to participate.

Competing interests

The authors declare they have no competing interests.

Author contributions

All authors contributed. CM led the development and preliminary evaluation of the programme and was principal investigator of this study. GB managed the project, including overseeing day-to-day recruitment and data collection. GB and CM drafted the initial study design with input from AB, JL VB, MT, MF, AM and SL. AB, AM, MF, and CM designed the original programme. KW recruited programme facilitators and arranged delivery sites. GB, BCF, KW and AG recruited participants and facilitated data collection. AM, MF, BCF, and KW planned, prepared, and delivered facilitator training and support. JL led the programme refinement process supported by GB, AB, VB and MT. AB, BCF,

For peer review only - http://bmjopen.bmj.com/site/about/guidelines.xhtml

AG, SM and JL coded and analysed the qualitative data. SL facilitated infrastructure support through the National Institute for Health Research Applied Research Collaboration South West Peninsula. All authors served on the Trial Management Group, contributed to drafting this paper, and approved the final manuscript. The study sponsor is the University of Exeter.

Acknowledgments

We thank members of the PenCRU Family Faculty and the Stakeholders Advisory Group for their involvement in the development of the intervention and the design and execution of this study, and the Council for Disabled Children for their collaboration. The authors are grateful to all participating parent carers and their families and the facilitators who delivered the programme. We thank our colleagues within the wider PenCRU team, Katharine Fitzpatrick for organising the parent carer working group meetings and Tania Hind for administrative support. We also acknowledge the support of the National Institute for Health Research Clinical Research Network (NIHR CRN).

Data availability statement

The datasets analysed during the current study are available from the corresponding author on reasonable request.

Licence statement

The Submitting Author accepts and understands that any supply made under these terms is made by BMJ to the Submitting Author unless you are acting as an employee on behalf of your employer or a postgraduate student of an affiliated institution which is paying any applicable article publishing charge (“APC”) for Open Access articles. Where the Submitting Author wishes to make the Work available on an Open Access basis (and intends to pay the relevant APC), the terms of reuse of such Open Access shall be governed by a Creative Commons licence.

For peer review only - http://bmjopen.bmj.com/site/about/guidelines.xhtml

List of Boxes

Box 1. Healthy Parent Carer's Programme and online materials

Box 2. Facilitator training programme

Box 3. Process evaluation measures and sampling

Box 4. Selected quotes illustrating facilitators' experiences

Box 5. Selected quotes illustrating participant' experiences

List of Figures

Figure 1. Flow of participants and measures

Figure 2. Programme refinement process

List of Supplementary Documents

Supplementary document 1a - HPC intervention details

Supplementary document 1b – TIDIER checklist

Supplementary document 2 - Study protocol publication

Supplementary document 3 - Facilitator training feedback questionnaire

Supplementary document 4 - Facilitator delivery checklist (Module 1)

Supplementary document 5 - Facilitator focus group topic guide

Supplementary document 6 - Participant end of session questionnaire

Supplementary document 7 - Participant end of programme questionnaire

Supplementary document 8 - Participant 6 month follow up questionnaire

Supplementary document 9 - Participant interview topic guide (intervention)

Supplementary document 10 - Participant interview topic guide (control)

Supplementary document 11 - Additional quotes and comments from facilitators and participants

For peer review only

Boxes

Box 1. Summary of the Healthy Parent Carer's Programme (including the online materials)

Face-to-face sessions

Content - Activities based on CLANGERS, an extension of the '5 ways to wellbeing'.

- Connect, Learn, be Active, Notice, Give, Eat well, Relax and Sleep.

Format – 12 modules over 24 hours

Setting – Community sites (2 special schools, 1 children's hospice, 1 Parent Carer Forum premises, 1 adult learning community venue, and 1 hotel regularly used for parent carer meetings).

Delivery – Six four-hour daytime sessions (comprising 2 modules per session) or 12 two-hour evening sessions (one module per session) delivered to groups of 4-12 parent carers.

Personnel - One Lead and one Assistant Facilitator per group.

Online materials

Included written content which provided participants with information on the CLANGERS, note-taking space to reflect on their own thoughts and templates for participants to develop their own goals and action plans. Audio and video recordings were also provided to illustrate each of the CLANGERS.

The content related to each module was released to participants in each group after it was delivered in their specific group sessions.

only

For peer review only - http://bmjopen.bmj.com/site/about/guidelines.xhtml

Box 2. Facilitator Training Programme

FORMAT

- **Block 1** - 2 days (Lead Facilitators only). November 2018
- **Block 2 -** 2 days (Lead and Assistant Facilitators). November 2018 (three weeks following Block 1)
- **Block 3 –** 1 day (Lead and Assistant Facilitators). April 2018 (after delivery of cohort 1)

CONTENT

- **Block 1** Overview of programme; exploration of wellbeing and the CLANGERS; facilitator roles and responsibilities; modelling delivery; research processes; safeguarding
- **Block 2** Overview of programme; exploration of wellbeing and the CLANGERS; facilitator roles and responsibilities; facilitator skills and competencies; group facilitation; managing group dynamics; modelling delivery; practicing delivery; research processes; safeguarding.
- **Block 3** Refresher training; review of CLANGERS, facilitator reflections, research processes, safeguarding.

PERSONNEL –Researchers and two parent carers (MF and BM) who co-produced the programme and delivered the proof of concept pilot.

FACILITATOR RECRUITMENT - Parent carers who are senior facilitators of the Council for Disabled Children's Expert Parent Programme were referred to us to become Lead Facilitators, based on their experience in developing and facilitating programmes for parent carers.
Assistant facilitators were recruited through adverts shared through contacts in the project Stakeholders Advisory Group. Adverts included information about the role and person specification criteria. Applicants were interviewed by a researcher and selection decisions were made by the research team.

SAFEGUARDING - The safeguarding protocol for facilitators was outlined in training and provided in the delivery manual. If any concerns arose during the programme, facilitators were instructed to inform the study team and follow the protocol, which included a reporting flow chart and information for Multi-Agency Referral Units and other relevant contacts at each site.

For peer review only - http://bmjopen.bmj.com/site/about/guidelines.xhtml

Box 3. Process evaluation measures and sampling

Facilitator training feedback questionnaire: To assess self-reported knowledge, understanding, skills and confidence to deliver the intervention and facilitator reflections on the training. Data were collated and averaged to provide an overall score out of 5 for each training block for knowledge and understanding (of the programme, facilitation techniques, developing a positive group dynamic and facilitator roles and responsibilities), skills and confidence (to present information, lead activities, create a positive group dynamic, manage time and difficult situations). (supplementary document 3)

Facilitator support calls: To understand and respond to delivery challenges (two per site, one following week 1 and one halfway through the programme).

Facilitator delivery checklists*:* To understand experiences and views on group delivery (weekly checklist, also included a check on content delivered) (Module 1 example, supplementary document 4)

Focus group with facilitators: To understand Lead and Assistant Facilitator experiences and views on training, delivery, and programme content (supplementary document 5). All Lead and Assistant Facilitators involved in delivering the programme were invited to attend the focus group, which took place at a meeting room within the University of Exeter. The two-hour focus group took place once all groups were completed, led by JL and supported by BCF (researchers not involved in delivering the programme). The focus group was audio-recorded and transcribed verbatim (with any potentially identifiable information anonymised).

Participants' feedback questionnaires: To understand participant (control and intervention) experiences and views on programme content and delivery at the end of each group session (supplementary document 6) and at the end of the programme (supplementary document 7) and 6 month follow up (supplementary document 8).

Participant phone interviews: To understand and explore participant experiences, views and engagement with the group sessions and online materials (intervention and control). (supplementary documents 9 and 10). Twelve intervention (two per group/site) and 6 control participants (one per site) were sampled to ensure that two out of the four male carers in the study (1 control and 1 intervention) were interviewed and the range of parent carer challenges were represented (selection was based on Lead Facilitator comments and participant end of programme feedback). Interviews were carried out by a researcher not involved in delivering the programme (BCF) and were audio-recorded and transcribed verbatim, with names and other identifying information changed for confidentiality. Interviews took place as soon as possible after participants completed their post-intervention measures and before the six-month follow-up.

For peer review only

Box 4. Selected quotes illustrating facilitators' experiences

1.1. Motivations and expectations

I feel that this training is a natural continuation to take forward the previous training I have been involved in. I am passionate about empowering parents with high quality information, support and advice. (Facilitator pre-training questionnaire)

For me it was a mixture of interlinked personal and professional reasons… I'm keen to do good in the world, if I can, keen to use some of my professional skills and enhance my professional skills while doing that. (AF, focus group)

I know the power of groups and supporting each other… I know it's so important to have people that understand and support you, because it can be lonely. I had lots of hope that this would help other people and, yes, it definitely was fulfilled. (AF, focus group)

1.2. Training, preparation and support

Parent carers are a very specific group to deliver to and maybe something that is more specific around the baggage, if you'll excuse me for using that word. The baggage that parent carers bring into the room is extremely specific and the group dynamic isn't managing conflict, usually, with parent carers. It's very different. (…) I always find parent carers very quick to bond and support each other. Very, very quick; they really do move forward in that way. But when you've got an individual... Something about managing the individual rather than the group as a whole. (LF, focus group)

I didn't know enough about the programme before I actually joined in as an assistant… now, it would be fine going back but when you suddenly do the programme you haven't got a clue what's necessarily coming up next and you don't know how, emotionally, you're going to feel. (…) It probably would have been easier if I'd done the programme myself, because I could have then looked back at some notes, so any things that I come across being difficult, I would have had a clearer focus rather than what's actually going on with me at the time. (AF, focus group)

AF: For me, the support calls were more about the sharing of experiences - and the issues and part of the process and the research as well. I guess you [the researcher] were kind of in research mode to some extent in those calls. But the support seemed to me to be within the facilitator team.

LF: I agree absolutely with that. Totally. I think that the relationship that we had as co-facilitators is where I drew my support about anything that wasn't just on a practical basis.

AF: And that's different levels of support needed. (Focus group)

1.3. Programme content and group delivery

The repetitiveness of the way it was delivered really helped parents to predict and understand where they were going, so it put them in a very comfortable space as well. (…) Because every CLANGER is quite a repetitive process and it's unpicking the meanings, lots of self-reflection and we were using the collated feedback to constantly cross-reference and compare... (LF, focus group)

For peer review only - http://bmjopen.bmj.com/site/about/guidelines.xhtml

I found it hard work… and it was very intense and it was more counselling… because people were really disclosing lots of things about their lives and that way… the impact on us as facilitators was more intense experience… and whilst I think we didn't have anything we couldn't handle between us, it actually became more than the course is meant to be. (LF, focus group)

I think one of the things that I definitely felt was the relationship between the trainer and the assistant facilitator is amazing and I definitely feel like the absolute bonus is I've gained a really good friend who I think going on it together actually benefits our health and wellbeing, just delivering it together. It is really positive. (AF, focus group)

(see supplementary document 11 for additional quotes/comments)

For peer review only

Box 5. Selected quotes illustrating participants' experiences

2.1. Motivation to participate and expectations

I feel that being a parent-carer is really draining on your mental health and also I wanted to try and get healthy anyway, especially with it being January. (…) a bit of knowledge maybe about the way I feel and maybe a bit less guilt, the fact that I feel that I need to take time out for myself. (P13, control)

I just thought it could be useful because a lot of the time I find as a parent carer all the focus is on the children which is obviously the main thing but the parents need to be healthy and happy to give the children the best. (P8, intervention)

2.2. Experiences of the programme and its impact

It was fantastic. I think it was really well done. (…) I think it's changed my life. (…) I think the best thing was going to the group, meeting everybody in the group, just meeting like-minded people, having a break from life and being in a different place for a while, reflecting on aspects of my life that I didn't really think about or had put away, and I learnt more about myself. (P5, intervention)

Meeting people who understand and 'get it ' reduced my isolation. Having parent carers as facilitators helped a lot. Having 12 structured sessions made me commit to it and focus on my own health. Having structured fun informative sessions helped a lot - I definitely went through a process of change. The use of humour was really important to me - and learning to be more resilient. (End-of-programme feedback form).

A lot of it is very common sense stuff. (…) It's like a revelation without it being a revelation, because you know all that stuff, but you don't take the time to think about it and it was just very much about focusing on us and improving things for ourselves before we can do it for everyone else really. (P3, intervention)

Unfortunately, I think we were one of the groups that was very small, which was good in some ways because you got to know the people better and you had a bit more time, but then on the other side, at one point there was only two of us there and you've not got everybody's stories, you've not got everybody's experiences, it was just a couple of us. (P4, intervention)

For peer review only - http://bmjopen.bmj.com/site/about/guidelines.xhtml

If the group was a little bit bigger…you would get more input and there would be more discussion because even though we were able to discuss things, I think a bit more of an open, broader amount of different people with different lives would make it a lot more interesting. (P6, intervention)
Was there anything that I didn't like? I think the only thing that made me feel... was sometimes like the icebreaker thing, but that's just me feeling nervous about (…) sometimes in group situations if I'm asked my opinion on something or asked to think about something and you've got to kind of think quickly, I get really nervous. (…) I think at the beginning also you are worried about what will people think of me and how will I be perceived by other people. You don't want to say the wrong thing… (P2, intervention)

2.3. Views on online materials

Initially I was curious and then I think it dwindled off because I felt like the course was covering everything so thoroughly that would it add anything to what I had already had? (P4, intervention)

What was there was absolutely fantastic and I think the videos were a good way of doing it because I am better at learning through watching the videos and being able to go back to them rather than just having loads and loads of information to read… (P18, control)

I found doing the online course quite isolating. Personally, I would have made more changes if I had been in the group. (…) I have ticked the box, I have done it (…) I found myself thinking about everything else that I should be doing, whereas if I had gone to the group and they were watching that video… you are switched on to be watching that video, whereas for me, I was there thinking, 'Okay, I have got to do this, so I will sit down and do this… and then my mind… at one point, I was so tired that I was thinking, 'Oh gosh, I have got to go and make lunch'… (P18, control)

2.4. Factors influencing ability to engage with and benefit from the programme

…on some days you are impacted by inadvertent events and there is no relaxation, there is five hours' sleep. That's just the way it is. (P14, control)

I have some really quite big social care needs and health care needs of my own that are not being met and so it is much more difficult to sit there going, "Ah well, have I eaten properly today?" But those things don't exist so it's better than nothing. (…) But I think it's working really well for some people. Yes, I can see that other members of the group were getting masses more out of it than I was. So I think if it's the right thing for you then it's clearly way more beneficial. (P2, intervention)

I couldn't really make out whether I liked it or not. It was alright, it was pleasant enough, but I think I've got a whole bunch of underlying issues that are not being addressed… for some people it was clearly really hitting the spot… and for me, I'm going, if I don't actually get some proper mental health care and social care support I can't really look… well I can look at it but I can just go, "Yes, that will be nice, one day, maybe, mmm, okay". (…) (Interviewer: What did you not like or not find helpful, if anything?) I suppose the assumption that's it in my power to change some of these things when it just isn't. (P2, intervention)

For peer review only

For peer review only - http://bmjopen.bmj.com/site/about/guidelines.xhtml

References

1. Barlow J, Cullen-Powell L, Cheshire A. Psychological well-being among mothers of children with cerebral palsy. *Early Child Dev Care*. 2006;176(3-4):421-8.
2. Brehaut JC, Kohen DE, Raina P, et al. The health of primary caregivers of children with cerebral palsy: how does it compare with that of other Canadian caregivers? *Pediatrics*. 2004;114(2):e182-e91.
3. Brehaut JC, Kohen DE, Garner RE, et al. Health among caregivers of children with health problems: Findings from a Canadian population-based study. *Am J Public Health*. 2009;99(7):1254-62.
4. Emerson E. Mothers of children and adolescents with intellectual disability: social and economic situation, mental health status, and the self-assessed social and psychological impact of the child's difficulties. *Journal of Intellectual Disability Research*. 2003;47(4-5):385-99.
5. Gallagher S, Phillips AC, Oliver C, et al. Predictors of psychological morbidity in parents of children with intellectual disabilities. *J Pediatr Psychol*. 2008;33(10):1129-36.
6. Lach LM, Kohen DE, Garner RE, et al. The health and psychosocial functioning of caregivers of children with neurodevelopmental disorders. *Disabil Rehabil*. 2009;31(9):741-52.
7. Murphy NA, Christian B, Caplin DA, et al. The health of caregivers for children with disabilities: caregiver perspectives. *Child Care Health Dev*. 2007;33(2):180-7.
8. Oelofsen N, Richardson P. Sense of coherence and parenting stress in mothers and fathers of preschool children with developmental disability. *Journal of Intellectual and Developmental Disability*. 2006;31(1):1-12.
9. Olsson MB, Hwang C. Depression in mothers and fathers of children with intellectual disability. *Journal of Intellectual Disability Research*. 2001;45(6):535-43.
10. Singer GH, Floyd F. Meta-analysis of comparative studies of depression in mothers of children with and without developmental disabilities. *Am J Ment Retard.* 2006;111(3):155-69.
11. Arim RG, Miller AR, Kohen DE, et al. Changes in the health of mothers of children with neurodevelopmental disabilities: An administrative data study. *Res Dev Disabil*. 2019;86:76-86.
12. Lee M, Park C, Matthews AK, et al. Differences in physical health, and health behaviors between family caregivers of children with and without disabilities. *Disabil Health J.* 2017:565-70.
13. Tong HC, Haig AJ, Nelson VS, et al. Low back pain in adult female caregivers of children with physical disabilities. *Arch Pediatr Adolesc Med*. 2003;157(11):1128-33.
14. National Health Service. The NHS Long Term Plan 2019. https://www.longtermplan.nhs.uk/wp-content/uploads/2019/08/nhs-long-term-plan-version-1.2.pdf (accessed 30 Mar 2021)
15. Bourke-Taylor HM, Lee D-CA, Tirlea L, et al. Interventions to Improve the Mental Health of Mothers of Children with a Disability: Systematic Review, Meta-analysis and Description of Interventions. *J Autism Dev Disord*. 2021. DOI: 10.1007/s10803-020-04826-4
16. Ogourtsova T, O'Donnell M, De Souza Silva W, et al. Health coaching for parents of children with developmental disabilities: a systematic review. *Dev Med Child Neurol*. 2019;61(11):1259-65.
17. South J. A guide to community-centred approaches for health and wellbeing London: Public Health England/NHS England. 2015. https://assets.publishing.service.gov.uk/government/uploads/system/uploads/attachment_data/file/768979/A_guide_to_community-centred_approaches_for_health_and_wellbeing__full_report_.pdf (accessed 30 Mar 2021)

For peer review only - http://bmjopen.bmj.com/site/about/guidelines.xhtml

18. Johnstone P. Realising the potential of community assets to improve our health and wellbeing: Public Health England; 2017 https://publichealthmatters.blog.gov.uk/2017/09/27/realising-the-potential-of-community-assets-to-improve-our-health-and-wellbeing. (accessed 30 Mar 2021)
19. Hammond P. Staying Alive: How to Get the Best Out of the NHS - advice from a doctor. London: Quercus; 2015. 320 p.
20. Aked J, Cordon C, Marks N, et al. Five Ways to Wellbeing: A report presented to the Foresight Project on communicating the evidence base for improving people's well-being. London: New Economics Foundation; 2008.
21. Bartholomew Eldredge LK, Markham CM, Ruiter RA, et al. Planning health promotion programs: an intervention mapping approach. 4th ed. San Francisco: Jossey-Bass; 2016.
22. Borek AJ, McDonald B, Fredlund M, et al. Healthy Parent Carers programme: development and feasibility of a novel group-based health-promotion intervention. *BMC Public Health*. 2018;18(1):270.
23. Bjornstad G, Wilkinson K, Cuffe-Fuller B, et al. Healthy Parent Carers peer-led group-based health promotion intervention for parent carers of disabled children: protocol for a feasibility study using a parallel group randomised controlled trial design. *Pilot Feasibility Stud*. 2019;5(1):1-13.
24. Bjornstad G, Cuffe-Fuller B, Ukoumunne OC, et al. Healthy Parent Carers: feasibility randomised controlled trial of a peer-led group-based health promotion intervention for parent carers of disabled children. *Pilot Feasibility Stud*. 2021;7(1):144.
25. Moore GF, Evans RE, Hawkins J, et al. From complex social interventions to interventions in complex social systems: Future directions and unresolved questions for intervention development and evaluation. *Evaluation (Lond)*. 2019;25(1):23-45.
26. Moore GF, Audrey S, Barker M, et al. Process evaluation of complex interventions: Medical Research Council guidance. *BMJ*. 2015;350:h1258.
27. O'Cathain A, Croot L, Duncan E, et al. Guidance on how to develop complex interventions to improve health and healthcare. *BMJ Open*. 2019;9(8):e029954.
28. Craig P, Di Ruggiero E, Frohlich KL, et al. Taking account of context in population health intervention research: guidance for producers, users and funders of research. NIHR Journals Library. 2018.
29. O'Cathain A, Hoddinott P, Lewin S, et al. Maximising the impact of qualitative research in feasibility studies for randomised controlled trials: guidance for researchers. *Pilot Feasibility Stud*. 2015;1:32.
30. Reid MJ, Webster-Stratton C, Beauchaine TP. Parent training in head start: a comparison of program response among African American, Asian American, Caucasian, and Hispanic mothers. *Prev Sci.* 2001;2(4):209-27.
31. van Mourik K, Crone MR, de Wolff MS, et al. Parent Training Programs for Ethnic Minorities: a Meta-analysis of Adaptations and Effect. *Prev Sci.* 2017;18(1):95-105.
32. Castro FG, Barrera M, Jr., Holleran Steiker LK. Issues and challenges in the design of culturally adapted evidence-based interventions. *Annu Rev Clin Psychol*. 2010;6:213-39.
33. Berghs M, Atkin K, Graham H, et al. Implications for public health research of models and theories of disability: a scoping study and evidence synthesis. *Public Health Research*. 2016;4(8).

For peer review only - http://bmjopen.bmj.com/site/about/guidelines.xhtml

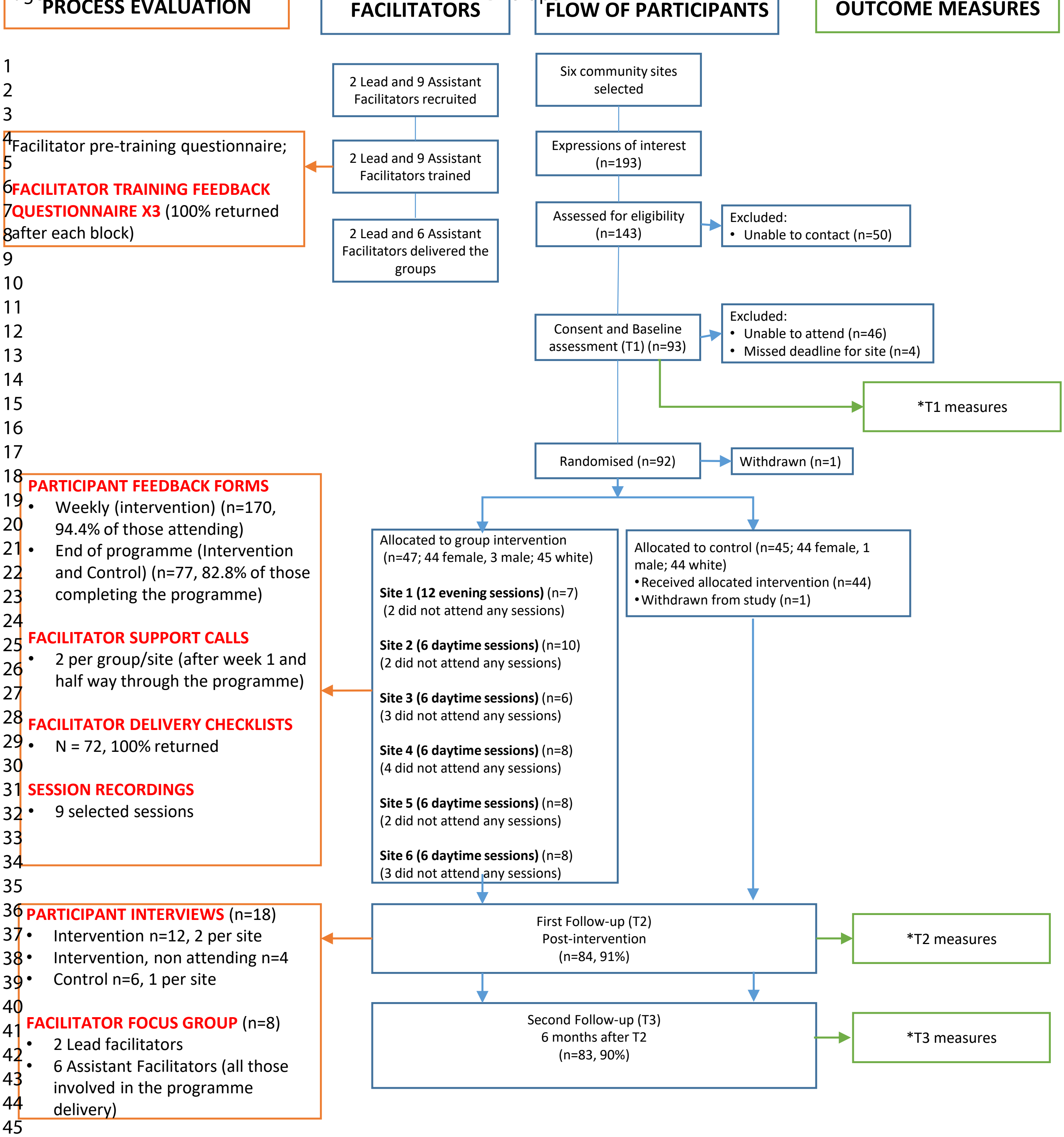

* Bjornstad G, Wilkinson K, Cuffe-Fuller B, et al. Healthy Parent Carers peer-led group-based health promotion intervention for parent carers of disabled children: protocol for a feasibility study using a parallel group randomised controlled trial design. Pilot and Feasibility Studies. 2019;5(1):1-13.

FIGURE 2 - Programme Refinement Process

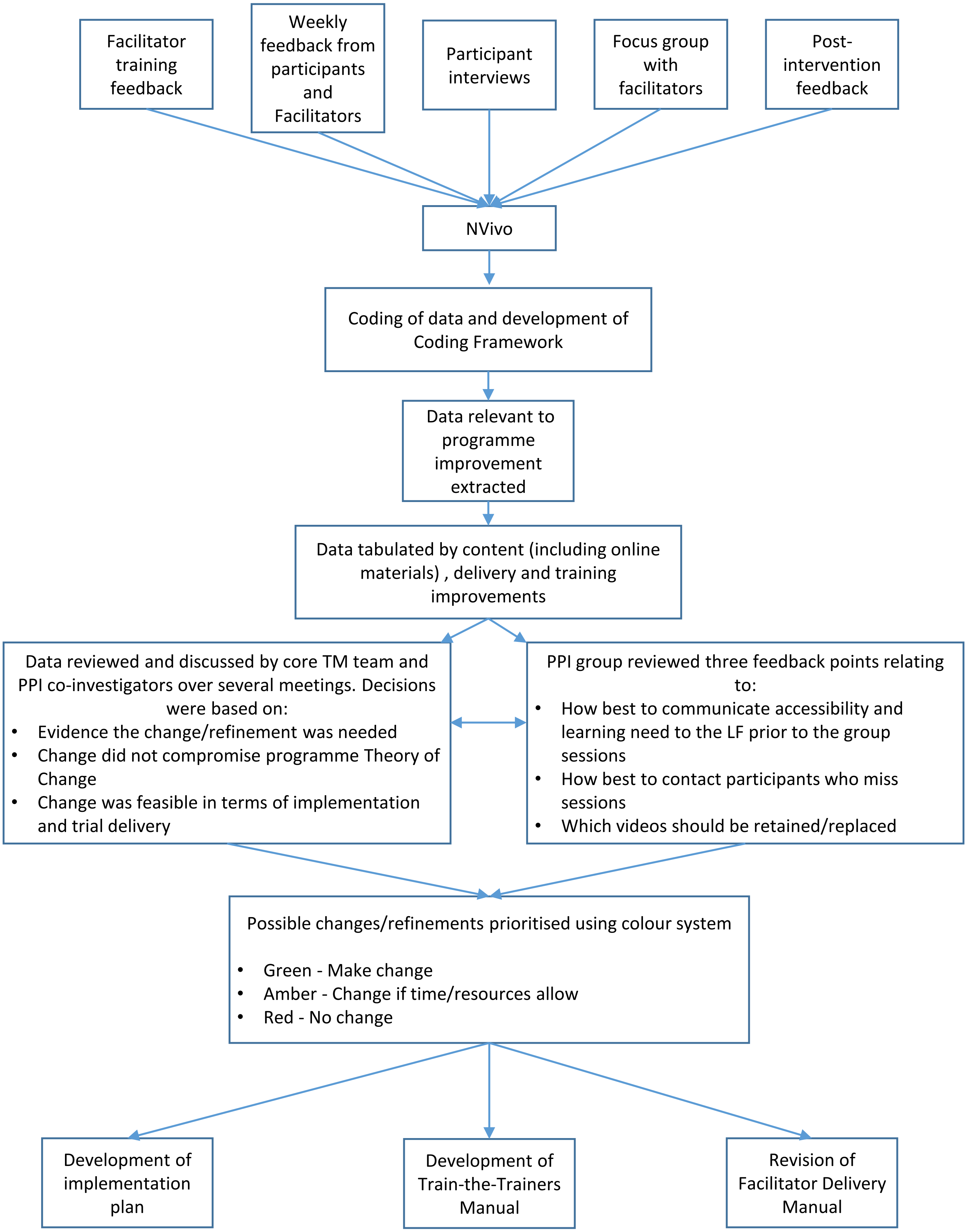

Supplementary Document 1a. Healthy Parent Carers intervention details

The details of the intervention are also reported in the intervention development paper (Borek et al., 2018) and the feasibility study protocol (Bjornstad et al., 2019). The results of the feasibility study are reported separately. The intervention details below are reported using the checklist for reporting of group-based interventions (Borek et al., 2015).

**INTERVENTION DESIGN**	
1. Intervention source or development methods	Developed based on an intervention mapping approach (Bartholomew et al., 2016), in partnership with parent carers. Details are described in Borek et al., 2018.
2. General setting	Community setting (with 6 sites/venues)
3. Venue characteristics	6 sites/venues, with different characteristics (2 special schools, 1 children's hospice, 1 Parent Carer Forum premises, 1 adult learning community venue, and 1 hotel regularly used for parent carer meetings).<br><br>All rooms set up to enable interaction between participants (i.e. facing each other).
4. Total number of group sessions	6 or 12 sessions
5. Length of group sessions	6 daytime sessions of 4 hours (2 modules per session)<br>or 12 evening sessions of 2 hours (1 module per session)
6. Frequency of group sessions	Weekly
7. Duration of the intervention	6 or 12 weeks
**INTERVENTION CONTENT**	
8. Change mechanisms or theories of change	The intervention logic model and intended mechanisms of change are reported in Borek et al., 2018. In brief, the intervention aimed to increase knowledge of health-related behaviours, skills in making health-related changes, improve attitudes towards change, increase self-efficacy, and provide social support to make changes, leading to behaviour change (based on CLANGERS) and psychological change (increased empowerment and resilience), leading to improved health and wellbeing.
9. Change techniques	• Provide/exchange information about behaviour-health link<br>• Provide/exchange instructions on practical strategies<br>• Prompt barrier identification and problem solving<br>• Prompt practice<br>• Prompt specific goal setting (for behaviours)<br>• Prompt use of prompts and rewards<br>• Prompt self-monitoring<br>• Prompt goal/progress review (behaviours)<br>• Provide general support and encouragement<br>• Provide opportunities for social comparisons
10. Session content	12 modules:<br>1. Introduction to health and wellbeing<br>2. Introduction to CLANGERS<br>3. Connect<br>4. Learn<br>5. be Active<br>6. take Notice<br>7. Give<br>8. Eat well

	9. Relax<br>10. Sleep well<br>11. Managing stress<br>12. Keeping healthy
11. Sequencing of sessions	Yes – sessions delivered sequentially (as above)
12. Participants' materials	In the sessions: goal setting and CLANGERS diary sheets given to participants, and other materials as parts of group activities.<br><br>Online Healthy Parent Carers materials (written information for each module, links to videos watched in the sessions)
13. Activities during the sessions	Each session started with a 'warm-up' activity and a review of the past week. This was followed by discussions focused on the topic of each module (e.g. 'what does it mean to you to relax? How do you relax? Why is relaxing important? What might stop you from, or help you, relax?). For each module there was a practical activity to help illustrate the key messages or the CLANGERS (e.g., colouring, 'a day in life of a parent carer', a mindful walk). For details, see Borek et al., 2018.
14. Methods for checking fidelity of delivery	Checklists were completed by the facilitators after each session; a sample of audio-recordings of group sessions were checked using the checklist independently (double-checking) by two researchers. Details are reported separately.
**PARTICIPANTS**	
15. Group composition	Parent carers, all female, except for two groups one of which had one male carer, the other of which had two. Ages ranged from 26-71 years (mean 42.5 years)
16. Methods for group allocation	Participants were recruited locally for each site (then randomised to the group intervention or control).
17. Continuity of participants' group membership	The same participants remained in the same group for the duration of the intervention.
18. Group size	7-10 participants were assigned per group. The attendance varied between sessions and groups (lowest attendance being 2 participants over 6 sessions). Details of attendance are reported separately.
**FACILITATORS**	
19. Number of facilitators	2 facilitators per group: one lead facilitator (LF) and one assistant facilitator (AF).<br><br>Overall 2 LF and 6 AF delivered the programme
20. Continuity of facilitators' group assignment	The same pairs of LF and AF facilitated the same groups.
21. Facilitators' professional background	LF: experienced facilitators of training for parent carers delivered through the Council for Disabled Children (e.g., the Expert Parent Programme).<br><br>AF: three had some experience of facilitating parent carer groups and/or training.
22. Facilitators' personal characteristics	All facilitators were parent carers. Both LF were female and one of the six AFs was male.
23. Facilitators' training in intervention delivery	LF received 4 (2 x 2) days of training in delivering the programme; one block of 2 days of these were delivered

For peer review only

	together for lead and assistant facilitators, LFs and AFs also received one day of refresher training.
24. Facilitators' training in group facilitation	LF were trained and experienced in group facilitation as part of their facilitator/trainer roles for the Council of Disabled Children.<br><br>All facilitators received an overview of facilitating groups and managing group dynamics as part of the training in delivering the intervention.
25. Facilitators' materials	Facilitators delivered the sessions using a manual with instructions outlining all session activities.
26. Intended facilitation style	Participant-centred, interactive, and discussion-based (not didactic).

For peer review only

Supplementary Document 1b. TIDieR checklist for HPC intervention

TIDieR
Template for Intervention Description and Replication

The TIDieR (Template for Intervention Description and Replication) Checklist*:

Information to include when describing an intervention and the location of the information

Item number	Item	Where located ** Primary paper (page or appendix number)	Other † (details)
	**BRIEF NAME**		
**1.**	Provide the name or a phrase that describes the intervention.	__p4________	______________
	**WHY**		
**2.**	Describe any rationale, theory, or goal of the elements essential to the intervention.	__p4________	______________
	**WHAT**		
**3.**	Materials: Describe any physical or informational materials used in the intervention, including those provided to participants or used in intervention delivery or in training of intervention providers. Provide information on where the materials can be accessed (e.g. online appendix, URL).	__p5________	Box 1_______
**4.**	Procedures: Describe each of the procedures, activities, and/or processes used in the intervention, including any enabling or support activities.	__p6________	Supplementary document 1a parts 8-13
	**WHO PROVIDED**		
**5.**	For each category of intervention provider (e.g. psychologist, nursing assistant), describe their expertise, background and any specific training given.	__p5________	Box 2, Supplementary document 1a parts 21-25
	**HOW**		
**6.**	Describe the modes of delivery (e.g. face-to-face or by some other mechanism, such as internet or telephone) of the intervention and whether it was provided individually or in a group.	__p5________	Box 1________

For peer review only

	**WHERE**		
**7.**	Describe the type(s) of location(s) where the intervention occurred, including any necessary infrastructure or relevant features.	___p5________	Supplementary document 1a, parts 2-3
	**WHEN and HOW MUCH**		
**8.**	Describe the number of times the intervention was delivered and over what period of time including the number of sessions, their schedule, and their duration, intensity or dose.	___p5________	Supplementary document 1a, parts 4-7
	**TAILORING**		
**9.**	If the intervention was planned to be personalised, titrated or adapted, then describe what, why, when, and how.	___N/A_______	______________
	**MODIFICATIONS**		
**10.‡**	If the intervention was modified during the course of the study, describe the changes (what, why, when, and how).	___N/A_______	______________
	**HOW WELL**		
**11.**	Planned: If intervention adherence or fidelity was assessed, describe how and by whom, and if any strategies were used to maintain or improve fidelity, describe them.	___p7 _______	Supplementary document 4; Bjornstad et al. (2021)
**12.‡**	Actual: If intervention adherence or fidelity was assessed, describe the extent to which the intervention was delivered as planned.	___p7________	______________

** **Authors** - use N/A if an item is not applicable for the intervention being described. **Reviewers** – use '?' if information about the element is not reported/not sufficiently reported.

† If the information is not provided in the primary paper, give details of where this information is available. This may include locations such as a published protocol or other published papers (provide citation details) or a website (provide the URL).

‡ If completing the TIDieR checklist for a protocol, these items are not relevant to the protocol and cannot be described until the study is complete.

For peer review only

* We strongly recommend using this checklist in conjunction with the TIDieR guide (see *BMJ* 2014;348:g1687) which contains an explanation and elaboration for each item.

* The focus of TIDieR is on reporting details of the intervention elements (and where relevant, comparison elements) of a study. Other elements and methodological features of studies are covered by other reporting statements and checklists and have not been duplicated as part of the TIDieR checklist. When a **randomised trial** is being reported, the TIDieR checklist should be used in conjunction with the CONSORT statement (see www.consort-statement.org) as an extension of **Item 5 of the CONSORT 2010 Statement.** When a **clinical trial protocol** is being reported, the TIDieR checklist should be used in conjunction with the SPIRIT statement as an extension of **Item 11 of the SPIRIT 2013 Statement** (see www.spirit-statement.org). For alternate study designs, TIDieR can be used in conjunction with the appropriate checklist for that study design (see www.equator-network.org).

References:

Bartholomew Eldredge LK, Parcel GS, Kok G, Gottlieb NH, Fernandez ME. *Planning health promotion programs: an intervention mapping approach*. 4th ed. San Francisco: Jossey-Bass; 2016.

Bjornstad G, Wilkinson K, Cuffe-Fuller B, Fitzpatrick K, Borek A, Ukoumunne OC, Hawton A, Tarrant M, Berry V, Lloyd J, McDonald A. Healthy Parent Carers peer-led group-based health promotion intervention for parent carers of disabled children: protocol for a feasibility study using a parallel group randomised controlled trial design. *Pilot and Feasibility Studies*. 2019;5(1):1-3.

Bjornstad G, Cuffe-Fuller B, Ukoumunne OC, et al. Healthy Parent Carers: feasibility randomised controlled trial of a peer-led group-based health promotion intervention for parent carers of disabled children. *Pilot Feasibility Stud*. 2021;7(1):144.

Borek AJ, Abraham C, Smith JR, Greaves CJ, Tarrant M. A checklist to improve reporting of group-based behaviour-change interventions. *BMC Public Health*. 2015;15(1):963.

Borek AJ, McDonald B, Fredlund M, Bjornstad G, Logan S, Morris C. Healthy Parent Carers programme: development and feasibility of a novel group-based health-promotion intervention. *BMC Public Health*. 2018;18(1):270.

For peer review only

Bjornstad *et al. Pilot and Feasibility Studies* (2019) 5:137
https://doi.org/10.1186/s40814-019-0517-3

Pilot and Feasibility Studies

STUDY PROTOCOL Open Access

Healthy Parent Carers peer-led group-based health promotion intervention for parent carers of disabled children: protocol for a feasibility study using a parallel group randomised controlled trial design

Gretchen Bjornstad[1], Kath Wilkinson[1], Beth Cuffe-Fuller[1], Katharine Fitzpatrick[1], Aleksandra Borek[2], Obioha C. Ukoumunne[3], Annie Hawton[4], Mark Tarrant[3], Vashti Berry[3], Jenny Lloyd[3], Annabel McDonald[5], Mary Fredlund[5], Shelley Rhodes[6], Stuart Logan[1] and Christopher Morris[1*]

Abstract

Background: Parent carers of disabled children are at increased risk of mental and physical health problems. They often experience challenges to maintaining good health which have implications for their well-being and their ability to care for their children. In response to these needs, researchers and parent carers developed the Healthy Parent Carers (HPC) programme. It is a peer-led, group-based intervention that promotes behaviours associated with health and well-being. The aims of this trial are to assess the acceptability of the HPC programme and the feasibility of its delivery in the community and to assess the feasibility and acceptability of the design of the definitive trial to evaluate the programme's effectiveness and cost-effectiveness.

Methods: We will establish six research sites and train facilitators to deliver the manualised intervention. Parent carers of children with special educational needs and disabilities will be individually randomised, stratified by group delivery site, to either take part in a group programme and online resources (intervention) or to receive access to the online resources only (control). Measures of mental health; well-being; health-related quality of life; health behaviours; patient activation; protective factors such as resilience, social connections, and practical support; and use of health care, social care, and wider societal resources will be collected before randomisation (baseline), immediately post-intervention, and 6 months later. Recruitment of participants, adherence to the programme, and the dose received will be assessed. Group sessions will be audio-recorded to evaluate the fidelity of delivery and participant engagement. Participants' and facilitators' feedback on the programme content and delivery, their experience, and the acceptability of the outcome measures and trial design will be collected through feedback forms, interviews, and focus groups.

Discussion: This trial will assess whether the programme delivery and evaluative trial design are feasible, to inform whether to progress to a definitive randomised controlled trial to test the effectiveness and cost-effectiveness of the Healthy Parent Carers programme.

(Continued on next page)

* Correspondence: christopher.morris@exeter.ac.uk
[1]Peninsula Childhood Disability Research Unit (PenCRU) and National Institute for Health Research Applied Research Collaboration South West Peninsula (PenARC), University of Exeter Medical School, University of Exeter, St. Luke's Campus, Heavitree Road, Exeter EX1 2LU, UK
Full list of author information is available at the end of the article

© The Author(s). 2019 **Open Access** This article is distributed under the terms of the Creative Commons Attribution 4.0 International License (http://creativecommons.org/licenses/by/4.0/), which permits unrestricted use, distribution, and reproduction in any medium, provided you give appropriate credit to the original author(s) and the source, provide a link to the Creative Commons license, and indicate if changes were made. The Creative Commons Public Domain Dedication waiver (http://creativecommons.org/publicdomain/zero/1.0/) applies to the data made available in this article, unless otherwise stated.

(Continued from previous page)

Trial registration: ISRCTN, ISRCTN151144652, registered on 25 October 2018; ClinicalTrials.gov, NCT03705221, registered on 15 October 2018.

Keywords: Behaviour change, Well-being, Resilience, Peer support, Patient and public involvement, Disabled children, Parents, Carers

Background

There are an estimated 960,000 disabled children in the UK, which is 7.3% of the population of children aged 0–18 years [1]. Parent carers of disabled children commonly report higher levels of stress and depression [2–11] and poorer physical health [3, 4, 7, 8, 12–14] than parents of typically developing children. Population-based studies suggest these health problems persist and may worsen over time [15]. These problems have implications for their ability to care for their children.

Parent carers often find the demands of caregiving have a negative impact on their physical and emotional health. Nevertheless, it is important to acknowledge that not all parents of disabled children report that their child's difficulties negatively affect their psychological or physical health [5], and in fact, some report positive impacts [8, 16]. Indeed, some parent carers may perceive a high burden with looking after a child with a relatively 'mild' condition whereas others, whose child may have more severe disabilities, may not perceive caring as high a burden [4].

Some interventions target external factors, such as navigating healthcare services [17], while others target levels of stress [18, 19] or emotional and social support [20]. A systematic review of psychological therapies for parents of children with chronic illness suggested promising results in terms of improved parent mental health, particularly for problem-solving therapy [21]. No benefits were found for cognitive behavioural therapy or family therapy on parent outcomes; however, the quality of the evidence was low and analyses were limited by lack of data available to the reviewers. A systematic review of mindfulness interventions for parents of children with autism indicated potentially positive effects on parents' stress levels and psychological well-being, with studies reporting good attendance and retention in 8-week programmes [22]. There is growing evidence that groups can facilitate change processes beneficial to health and well-being [23, 24] by enabling the formation of strong psychological connections and/or social identification with other group members which can enhance engagement, and thus possibly increase the interventions' effectiveness [25, 26].

The idea for this research came directly from parent carers who had been involved in a study evaluating peer support for parent carers [20]. They wanted to extend the benefits of emotional support to specific strategies to improve health and well-being. Researchers and parent carers in the Peninsula Childhood Disability Research Unit (PenCRU) Family Faculty co-created the Healthy Parent Carers (HPC) programme [27].

Previously, we tested the principle and acceptability of a 6-week intervention programme with one group of seven parent carers, delivered by the intervention developers. The intervention was developed using Intervention Mapping [28] and extensive stakeholder engagement and is described in detail in a separate paper [29]. Participants had children with various conditions including autism, cerebral palsy, and acquired brain injury. Retention of participants in our preliminary study was high with all staying until the end of the 6-week programme and 2-month follow-up. Participants' and facilitators' feedback were positive, indicating the intervention was feasible to deliver and acceptable to, and valued by, participants. The intervention content and delivery methods were refined following feedback, and the manual was updated.

This feasibility trial will provide information that will be used to determine whether to progress to a definitive trial of the HPC programme, which would have the following objectives:

1) To determine whether the peer-led, group-based HPC programme is more effective at improving health and well-being compared to providing online information only
2) To estimate the costs of delivering the HPC programme, and the cost-effectiveness of the programme, versus the provision of online information
3) To understand how the Healthy Parent Carers intervention is working, for whom, and in what context to inform the implementation of the programme should it be shown to be effective

The current trial aims to assess the acceptability of the HPC programme and the feasibility of its delivery in the community, as well as the feasibility and acceptability of the design of the definitive trial in order to evaluate whether a fully powered randomised controlled trial is warranted and to determine the optimal trial design.

For peer review only

Methods

Objectives

This trial has two overarching aims:

1. To evaluate whether the programme can be delivered in the community by facilitators other than the developers, specifically to:
   (a) Assess the feasibility of establishing venues, and identifying and training group facilitators to be in a position to deliver the intervention
   (b) Assess the fidelity of intervention delivery in terms of format, content, and quality
   (c) Assess the experience and engagement of participants, facilitators, and trainers
   (d) Assess the programme attendance
2. To provide information necessary to design a definitive randomised controlled trial, specifically to:
   (a) Assess the feasibility of recruiting participants in different sites
   (b) Assess the acceptability of randomisation of parent carers
   (c) Assess the attrition and completion and proportion of any missing data in questionnaire measures
   (d) Appraise the performance of candidate health and well-being outcome measures in terms of acceptability to participants, and feasibility and interpretability for researchers
   (e) Estimate the variability (standard deviation) and the level of clustering within programme delivery groups in the intervention arm to help inform the sample size calculation for the definitive trial
   (f) Test the proposed cost-effectiveness framework for a future randomised trial

This will help inform whether to progress to a definitive randomised controlled trial to test the effectiveness and cost-effectiveness of the programme and provide information necessary to design the trial.

Design

A feasibility trial using a parallel group randomised controlled trial design will be carried out in six sites in the southwest of England. Participants will be randomly allocated to receive the group-based programme and access to online programme resources or to a control group receiving access to the online resources only. Data collection will take place at three time points in both trial arms at baseline (prior to randomisation), immediately post-intervention, and 6 months later. As the intervention can be delivered over 6 or 12 weeks, the post-intervention data collection time point will vary relative to randomisation but will be consistent in terms of the amount of time passing after completion of the intervention. Participants in the control arm in each randomised site will complete measures at the same time as participants in the intervention arm for that site. The two arms will therefore be balanced in terms of the timing of outcome measures. The trial design and the flow of participants through the trial are illustrated in the trial flow chart and SPIRIT figure (Fig. 1 and Table 1). The SPIRIT checklist is provided as an additional file (see Additional file 1).

Public involvement

This project has a strong ethos of parent carer and stakeholder engagement from inception. The public involvement in this project will ensure the following: (a) the research is conducted in an acceptable manner, (b) the research outputs are relevant and useful to parents of children with special educational needs and disabilities, and (c) our dissemination materials and methods are appropriate and accessible.

Over 40 parents of children with a range of conditions from the PenCRU Family Faculty public involvement group have participated in a study-specific working group since 2014. Our Stakeholder Advisory Group (SAG) includes representatives from the local authority, public health, parent carer forums, relevant charities, and special schools.

Parent carers have been involved in all stages of developing the intervention and designing the feasibility trial including:

a) Proposing the idea for the project based on their needs and experiences
b) Co-designing and refining the intervention and training content and delivery methods
c) Providing feedback on research methods including the selection of the comparison conditions
d) Advising on the content and form of the Resource Use Questionnaire for use as part of the cost-effectiveness framework
e) Contributing to interpreting and disseminating the findings of the previous study
f) Interviewing and hiring research staff
g) Discussing and advising on the design of the feasibility trial
h) Recommending responses to peer reviews when applying for funding.

Study setting and location

In collaboration with our SAG, we will identify six venues (e.g. schools, community centres, adult and community learning venues) where it is possible to establish and host a group. We will agree days, times, durations

For peer review only

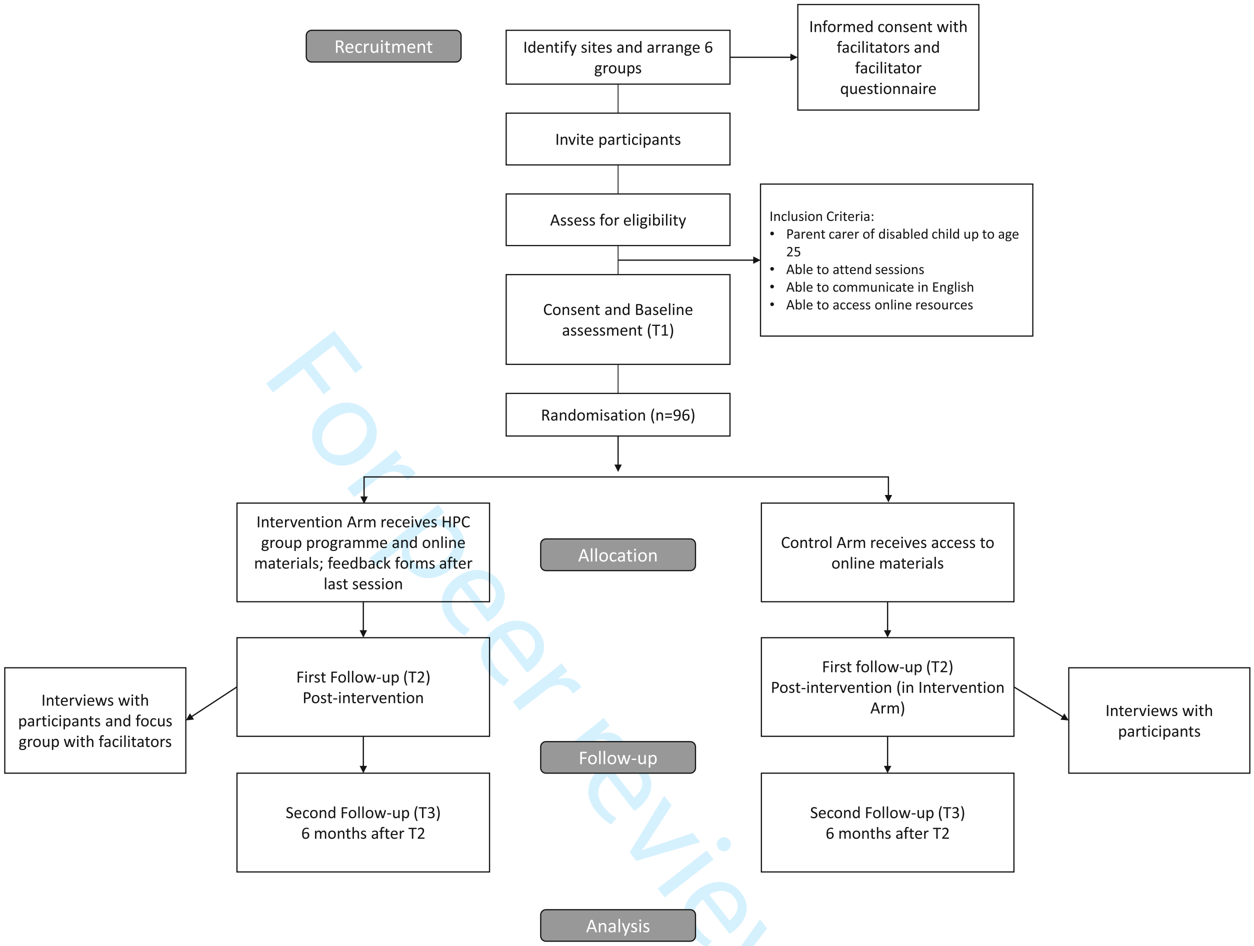

Fig. 1 Trial flow chart

and frequencies of sessions, and local named organisers for each venue.

Sample size

We aim to recruit 96 participants, to be allocated on 1:1 ratio to intervention and control. This is a large enough sample to estimate the percentage providing data at follow-up (assumed to be 80%—76 participants) with a margin of error of 10 percentage points based on the lower bound of the 95% confidence interval. Assuming that 38 participants are followed up in each trial arm, this will be large enough to estimate the standard deviation for continuous outcomes in each arm within 29% of its true value based on the upper bound of the 95% confidence interval. Finally, 76 participants at follow-up *are* large enough to estimate a correlation coefficient of 0.5 between baseline and follow-up scores for a continuous outcome with a margin of error of 0.19 based on the lower bound of the 95% confidence interval. We will randomise a minimum of *8* and *a* maximum of 24 participants at each of the 6 sites. This will mean that 4–12 participants will be allocated to each of the intervention and control trial arms at each site. We expect the ideal group size to be between 6 and 12 people but are allowing for potential attrition and variation in recruitment between sites.

Inclusion criteria

People meeting the inclusion criteria are (1) primary carers of children with additional needs and/or disabilities (participants who self-identify as primary carers are eligible; the child can be up to 25 years old consistent with the current Department of Health and Department of Education Special Educational Needs and Disability (SEND) legislation in England and The Children's Act; no named diagnosis is necessary, and we are not limiting to specific conditions), (2) willing and able to attend the programme group meeting session(s) on arranged dates/times, and (3) able to access online information.

For peer review only - http://bmjopen.bmj.com/site/about/guidelines.xhtml

Table 1 Schedule of enrolment, interventions, and assessments

TIMEPOINT	Enrolment (T1)			Allocation	Post-allocation	Post-intervention (T2)	6 months post-intervention (T3)
**ENROLMENT:**							
Eligibility screen	X						
Informed consent		X					
Allocation				X			
**INTERVENTIONS:**							
Group Healthy Parent Carers programme					—	—	
Online Healthy Parent Carers resources					—	—	
**ASSESSMENTS:**							
Demographics (15 items)			X				
About My Child -19 (19 items)			X				
Warwick-Edinburgh Mental Well-being Scale (WEMWBS) (14 items)			X			X	X
EuroQol-5 Dimensions (EQ-5D-5L) (5 items)			X			X	X
Patient Health Questionnaire-9 (PHQ-9) (9 items)			X			X	X
Health Promoting Activities Scale (HPAS) (8 items)			X			X	X
Patient Activation Measure (PAM) (13 items)			X			X	X
Parents' Assessment of Protective Factors (PAPF) (36 items) "Parent Statements"			X			X	X
ICEpop CAPability measure for Adults (ICECAP-A) (5 items)			X			X	X
Service and Resource Use questionnaire: Healthy Parent Carers (31 items)			X			X	X
Adverse event assessments					X	X	X
Compliance (attendance)					X	X	
Feedback Form (14 items)						X	
Interviews and focus groups						X	

For peer review only

Exclusion criterion

Potential participants who are not able to communicate in English are excluded. This is necessary because the programme has not yet been translated into other languages.

Recruitment

We will advertise the study in several ways. Press releases and interviews in local television and/or radio will be used to publicise the study. Members of our SAG will be asked to advertise the project to their members through their email lists and networks. We will also share study adverts and information via social media and through the PenCRU Family Faculty email list, asking *the* members to share in their networks. We will liaise with Information and Advice Services in each locality, staff in social services, and Special Educational Needs Coordinator *(*SENCOs*)* in schools in each study site to target potentially more isolated parent carers. We will also recruit participants at events for parent carers at the study venues and other venues in the southwest where interested parent carers can discuss the project with the research team. This recruitment strategy uses many different approaches because parent carers do not all access the same services and not all parent carers are connected with their local parent carer forums. We will ask all participants how they heard about the study during screening, and this information will be recorded to assess whether some recruitment methods may be more effective than others. However, we are mindful that while some methods may not result in large numbers of recruits, they may help us to reach parent carers who are more isolated and more in need of support and, as such, will be seen as important methods to take forward in a definitive trial.

Interested parent carers will contact the researchers. There will be *a* telephone or face-to-face screening to check *the* eligibility, understanding of the study, and to answer any questions. A researcher will meet each potential participant individually. Those who want to participate will sign a consent form and complete baseline questionnaires online using an electronic patient-reported outcome (ePRO) system with a researcher on hand for support as necessary [30]. Reasons for not consenting to participate will be recorded if provided by those who decline.

Allocation to trial arms

When recruitment is completed at each site, we will proceed to randomisation. Each of the six programme groups will constitute a study site, with participants who choose that group being randomised to either attend the group or receive the online resources only. A computer-generated randomisation sequence will be used to assign the participants in each site to the intervention and control arms. A block randomisation scheme will be implemented to ensure balance in the number of participants allocated to each trial arm, stratified by group delivery site. The allocation sequence will be concealed from researchers using an online central randomisation service setup and maintained by the Exeter Clinical Trials Unit (UKCRC Registration ID 65). Blinding will not be used in this trial.

All participants will receive an email and letter indicating the result of randomisation. The participants randomly allocated to the intervention arm will be sent details of the group sessions and be contacted by their lead facilitator before the first group session. Participants in both arms will receive a link to the online programme resources and instructions on the web page. We will monitor the number of participants who refuse participation and record their reasoning (if they wish to share it) to gauge the acceptability of our trial design.

Intervention

The group-based programme was developed using Intervention Mapping approach [28]. Full details of the intervention, including its development, logic model, and content (e.g. activities, behaviour change techniques), are available in a previous publication [29]. In brief, the programme aims to expand parent carers' social networks and provide social support from peers with a shared sense of social identity alongside targeted activities to improve parent carers' confidence, motivation, self-efficacy, and empowerment, thus creating the conditions for change necessary for them to feel able to make their own plan to prioritise healthy behaviours for themselves.

The programme content is based around a set of universal and evidence-based actions (called CLANGERS) associated with health and well-being. CLANGERS stands for Connect, Learn, be Active, Notice, Give, Eat well, Relax and Sleep [31]. The 'CLANG' component comprises the 'Five Ways to Wellbeing' based on the evidence from the foresight project on Mental Capital and Wellbeing [32]. Each of these behaviours is potentially more difficult for parent carers.

The programme content is organised into 12 modules lasting 2 h each. The modules can be delivered weekly over 6 sessions (comprising 2 modules per session) or 12 sessions (1 module per session). Our Family Faculty PPI working group suggested that offering either 6 longer sessions in the daytime or 12 shorter evening sessions would be reasonable for most parent carers. One or both options will be offered per area in order to maximise recruitment and to reflect likely real-world delivery in community settings, with 6 groups being delivered in the study in total. If uptake is very low for a particular

For peer review only - http://bmjopen.bmj.com/site/about/guidelines.xhtml

site, the delivery model may be adapted during recruitment to increase numbers.

Facilitators

The lead facilitators of the group-based programme will be experienced facilitators of the 'Expert Parent Programme' courses created by the Council for Disabled Children (CDC) with funding from the Department of Health. The facilitators are parent carers. CDC has a selection process, trains, and provides supervision for their facilitators to ensure that they facilitate the groups effectively. Their nationwide network of over 70 facilitators provides a sustainable model for the implementation of the programme in the future. Facilitators will use the Healthy Parent Carers Facilitator Manual that includes module outlines, content, timings, activities, and resources needed. The Facilitator Manual also includes safeguarding procedures for the facilitator to follow in case of any adverse events such as suicidal ideation or disclosure of safeguarding issues.

There will also be an assistant facilitator in each group to assist the facilitator in sessions. We will recruit a local parent carer for this role using a person specification detailing the required personal qualities and skills and a selection process.

We will provide training for lead and assistant facilitators. The training will take place over 4 days for lead facilitators and 2 days for assistant facilitators; it will be delivered by researchers and the parent carer co-investigators who co-developed the programme and facilitated the group in our previous study. Lead facilitators will receive ongoing supervision and support through the CDC and support from the research team. Conference calls with facilitators, assistants, and researchers will be convened to reflect on delivery of the sessions. These discussions will inform intervention design and training needs and provide a forum to share ideas and ways to address any challenges arising.

The Healthy Parent Carers online resources are part of the intervention. They reflect the content of the group sessions, provide space to write down reflections, and prompts to set specific goals and for self-monitoring of CLANGERS-related behaviours.

If the participants in the group programme miss a session, they will be telephoned by the facilitator, who will summarise the session and encourage the participant to reflect on their week, read the section of the HPC online resources, and set their weekly goals.

Control

Participants in the control arm will receive access to the HPC programme resources online with instructions. Risk of contamination between participants allocated to each arm is low because the intervention is predicated on participants developing a shared social identity as members of the HPC group. Participants in both arms will be asked whether they have had contact with participants in the other arm of the trial as part of a post-intervention feedback form.

Data collection

Study records

We will record the data on the feasibility of recruitment, including how many people respond to the adverts, how they heard about the study, reasons for not taking part for those who decline, and how many are successfully recruited. We will record delivery setting, delivery model, attendance, attrition, and reasons for missed sessions or withdrawal from the study. We will also monitor how long it takes to accrue the target number of participants at each site and at what point in the trial process any participants withdraw.

Sample characteristics

Demographic data will include gender, ethnicity, parent relationship status, number of children, employment status, level of education, income, housing status, and age, gender, and diagnosis (if any) of their disabled child. We will also collect information about their disabled child's functioning and health complexity using the About my Child measure (AMC-19) [33]. We will also use participants' postcodes to link with the Indices of Multiple Deprivation as an indicator of deprivation relative to England and Wales in the area where participants live [34].

Outcome measures

Participants will be asked to complete all measures before randomisation, immediately post-intervention, and 6 months post-intervention, regardless of attendance or engagement with the interventions. Based on the recommendations from our Family Faculty PPI working group, the measures will be available to complete online, using a computer, smartphone, or tablets. The Exeter Clinical Trials Unit will set up an online platform for participants to access and complete the measures. Participants may request to complete the measures on paper if they wish. Any measures completed on paper will be independently double-entered by two researchers. These requests will be monitored to track preferences for online- versus paper-based measures.

Two members of our Family Faculty PPI working group have tested the applicability and time to complete the measures (45 min). A £25 shopping voucher will be posted to participants as acknowledgement for completing measures at each time point.

The measures will comprise:

a) Warwick-Edinburgh Mental Well-being Scale (WEMWBS): The WEMWBS is a 14-item scale used to assess the mental well-being in the general population and in the evaluation of programmes aiming to improve mental well-being [35]. Responses are normally distributed in the general population. WEMWBS has been validated in the UK, Europe, and elsewhere. It has been tested with minority ethnic populations, users of mental health services, and carers. It is sensitive to changes occurring through participation in programmes that promote well-being such as health promotion programmes. A tariff of well-being-adjusted life-year weights is currently being developed for responses on the WEMWBS, which will enable the measure to be used in cost-effectiveness analyses recognised by the National Institute for Health and Care Excellence (NICE) [36].
b) Patient Health Questionnaire-9 (PHQ-9): The PHQ-9 is a 9-item measure that rates the frequency of symptoms and is designed for screening, diagnosing, monitoring, and measuring the severity of depression [37–39]. Categories based on the cutoff scores of 5, 10, 15, and 20 represent none, mild, moderate, moderately severe, and severe depression, respectively. As part of a safeguarding protocol, we will use the Patient Health Questionnaire (PHQ-9) to measure depressive symptoms. The PHQ-9 questionnaire is recommended by NICE to assess depression in adults [40], and its use is highlighted in clinical pathways [41], so the interpretation of scores is widely understood by GPs and primary care staff. There is good evidence, across a range of studies, for the validity, reliability, sensitivity, and specificity of the PHQ-9 for detecting depressive disorders [38, 39]. It can be administered repeatedly to assess change in depression in response to treatment. Question 9 screens for suicidal ideation. If the person scores higher than 0 on question 9, or at any other point discloses suicidal ideation, we will follow the safeguarding protocol.
c) EuroQol 5 Dimensions (EQ-5D-5 L): The EQ-5D-5 L is a measure of health-related quality of life. It consists of five items measuring five dimensions of health-related quality of life (mobility, self-care, usual activities, pain/discomfort, and anxiety/depression) and a vertical visual analogue scale measuring self-rated health [42]. QALY weights can be applied to EQ-5D scores, which can then be used to calculate QALYs, and the cost-per-QALY of the intervention in a future definitive trial. The EQ-5D is NICE's preferred measure for use in health technology cost-effectiveness analyses.
d) Parents' Assessment of Protective Factors (PAPF): The PAPF is a 36-item measure that assesses protective factors identified in the development of the Strengthening Families evidence-based parenting programme [43]. These protective factors are as follows: parental resilience, social connections, concrete support in times of need, and support of children's social and emotional competence. These factors relate to the determinants of change in the logic model for the Healthy Parent Carers Programme.
e) Health Promoting Activities Scale (HPAS): The HPAS is an 8-item measure of a person's estimation of the frequency with which they participate in a range of activities that promote or maintain health and well-being [44, 45]. It was developed for and validated with mothers of children with disabilities.
f) Patient Activation Measure (PAM): The PAM is a 13-item measure that measures the spectrum of skills, knowledge, and confidence in patients and captures the extent to which people feel engaged and confident in managing their own health and care [46, 47]. It has been tested with 100,000 patients with long-term conditions in England to establish the feasibility of using the PAM across the NHS, how activation can inform support for self-management, what support clinicians and commissioners need to use the measure effectively, and whether supporting activation can improve outcomes for patients in the NHS.
g) ICEpop CAPability measure for Adults (ICECAP-A): The ICECAP-A is a 5-item measure of capability, which includes the following aspects of well-being found to be important to adults in the UK: attachment, stability, achievement, enjoyment, and autonomy [48–50]. A set of UK well-being adjusted life-year weights are available for the ICECAP-A, enabling it to be used in economic evaluations.
h) Resource use questionnaire: We developed a study-specific resource use questionnaire in collaboration with parent carers. This includes health, social care, participant, and broader societal resource use, and draws on measures in the Database of Instruments for Resource Use Measurement (DIRUM) [51].

Process evaluation

In line with the MRC guidance on process evaluations, this study will include a process evaluation that is appropriate for the feasibility testing stage of the development-evaluation-implementation process for this intervention [52]. This process evaluation will allow for the exploration of the feasibility of implementation of the intervention by assessing uptake (recruitment) and retention, participant

For peer review only - http://bmjopen.bmj.com/site/about/guidelines.xhtml

engagement, fidelity of delivery (to content and quality), experiences of participants and facilitators, unintended consequences, and contextual factors which may influence experience and delivery.

The following data collection tools will be used to assess fidelity of intervention delivery, participant and trainer characteristics/motivations, trainer knowledge and self-efficacy to deliver the programme, participant engagement with the programme/online materials, and acceptability of the intervention and trial design.

a) Facilitator pre-training questionnaire: We will use a pre-training questionnaire to collect information about facilitators' characteristics, their motivations to take part, relevant background and experience, and expectations of delivering the programme.
b) Facilitator training feedback: Following delivery of the training, we will use a questionnaire to gather the facilitators' feedback about their self-reported knowledge, understanding, skills, and confidence to deliver the intervention and to gather their reflections on the training.
c) Facilitator delivery observations: We will use a checklist to assess lead facilitators' competence to deliver while observing their delivery of the programme content during the facilitator training. The checklist includes key skills and competencies linking to the objectives of the lead facilitator training and will enable trainers and research staff to assess facilitators' readiness to deliver the programme. This will also help to guide and plan additional or future training.
d) Facilitator checklist, records, and support calls: We will use a self-report checklist completed jointly by the facilitators to indicate which content they have covered in each session (adherence), the duration of the sessions (dose), and the participants' engagement. Facilitators will be asked to record attendance at each session. We will also arrange support calls with facilitators to gather more information about how the groups are going and any challenges to delivery.
e) Session recordings: We will audio-record group sessions and will sample two to three recordings from each group to assess fidelity to intervention content, quality of delivery, and participant engagement. A researcher will rate the delivery using the same checklist used by the facilitators after each session. A second researcher will rate one recording per group ($n = 6$, 14%). The two researchers will compare the scoring of the first three groups immediately, and any inconsistencies will be discussed with JL/MT, to ensure there is a clear understanding of the assessment criteria. The scores of the double-coded sessions will be agreed between researchers, and the sessions assessed by the researchers will be compared with the facilitators' scores.
f) Participants' feedback: We will collect feedback from treatment and control arm participants about the programme content and delivery, their experiences, and whether they had contact with participants in the other arm of the trial via feedback forms at the end of each group session for those in the intervention arm and at the end of the programme for those in both trial arms.
g) Participant interviews: We will sample purposively 12 participants (from different groups) in the intervention arm and 6 participants allocated to the control arm across all sites for semi-structured telephone interviews. For the intervention arm, these interviews will explore participants' experiences of, and engagement in, the programme and the group and their views on the group content, activities, and facilitators. All, control and intervention groups, participants will be asked in the interviews about engagement with online resources, perceived impact of the programme and any potential contextual influences, and acceptability of the trial processes and measures. We will also sample up to 4 participants (from different groups) who were allocated to the intervention arm of the trial but did not attend any group sessions to ask them about barriers to attending and whether anything could be done to promote attendance in future groups. All interviews with participants will last approximately 30 min and will be audio-recorded and transcribed verbatim, with names and other personal identifying information changed to protect confidentiality. Interviews will take place as soon as possible after the participants have completed their post-intervention measures and before they complete their 6-month follow-up measures.
h) Focus groups with facilitators: We will invite all lead facilitators and assistant facilitators to a focus group after the end of all groups. The focus group (lasting approximately 2 h) will cover facilitators' experiences of delivering, and engagement with, the programme, views on the programme content, activities and feasibility of programme delivery, facilitator training and skills, group management, and suggestions for improvements. The focus group will be audio-recorded and transcribed verbatim (with any potentially identifiable information anonymised).

For peer review only

Cost-effectiveness framework

We will develop and test a framework for assessing the cost-effectiveness of the intervention in a future randomised trial. We will:

a) Establish methods for estimating intervention resource use and costs (e.g. training of facilitators, facilitators' time, venue hire), in collaboration with the programme facilitators and site representatives
b) Develop a resource use questionnaire in collaboration with parent carers, drawing on measures in the Database of Instruments for Resource Use Measurement (DIRUM) repository [51]
c) Assess the acceptability to parent carers of the EQ-5D-5 L, the ICECAP-A, and the WEMWBS, judged by missing data and measurement properties [53].

Data analysis

Statistical/quantitative analysis

We will report the number of eligible people who self-refer and the percentage (with 95% confidence intervals (CIs)) of these that are randomised in the trial. These findings will also be reported separately for each type of delivery setting and delivery model to assess whether particular delivery settings or models are more popular and therefore would lead to higher recruitment rates in the subsequent definitive trial. We will also report numbers and percentages of people who heard about the study via different sources, organised into categories.

We will also report the percentage (with 95% confidence intervals) of participants who complete each assessment at each time point as an assessment of the acceptability of the measures and of the feasibility of collecting sufficient data in a definitive trial. We will summarise the characteristics of recruited participants using demographic data to allow for assessment of the representativeness of the sample relative to figures available from the Office for National Statistics on the population in the southwest of England. We will also report the baseline comparability of the trial arms with respect to demographics and outcome measures.

For the intervention arm, we will report the number and percentage of participants that attend each group session with 95% confidence intervals. The percentage of participants that are lost to follow-up at each follow-up point will be reported for each trial arm. Select characteristics (parent carer gender and age, child gender and age, Index of Multiple Deprivation quintile, study site (centre), and baseline scores on outcomes) will be compared between those who are and are not lost to follow-up within each trial arm using descriptive summaries, but no formal statistical tests. Acceptability, judged by missing data of EQ-5D-5 L and ICECAP-A, will help to plan methods for estimation of cost-effectiveness in the future trial.

Means, standard deviations, and the correlation between baseline and follow-up scores on continuous outcomes will be reported to inform the sample size calculation for the subsequent definitive trial.

Level of clustering within groups in the intervention arm will be quantified using the intra-cluster (intra-group) correlation coefficient to inform the sample size calculation for the definitive trial; however, we recognise the relatively small sample size for this purpose, and it will be used alongside information about levels of clustering in published studies of trials of similar group-based interventions in similar settings.

We will compare the outcomes at follow-up between the two trial arms based on the intention-to-treat principle with participants analysed according to the trial arm they were randomised to. Missing data will not be imputed. We will report only confidence intervals for the intervention effect and no p values, in line with the extension to the CONSORT statement for reporting randomised pilot and feasibility studies [54].

Analysis of process data

Descriptive statistics will be reported for the quantitative data collected in delivery observation checklists, facilitator checklists, checklists used to assess intervention session recordings, and participant feedback forms.

Qualitative data collected from feedback forms, interviews, and focus groups will be analysed thematically to provide insights into participants' experiences of the programme, intervention acceptability, and suggestions for improvement, and to enhance understanding of the impact of the intervention and the mechanisms of change in relation to the programme logic model [54]. Data will be analysed using inductive thematic analysis [55] separately for each data source (i.e. feedback comments, interviews, and focus groups), following the same approach. Some issues will emerge as more salient than others and the interpretation of findings will be influenced by the original research objectives as well as the themes emerging directly from the data.

NVivo software (version 12 Pro for Windows, QSR International) will be used to organise and analyse the qualitative data. Initially, two researchers will independently read and code line-by-line a sample of the data and discuss their coding to develop and agree on the coding framework. New codes may be added as the coding proceeds, and the codes and coded data will be reviewed. Codes will be defined, compared to each other, and organised into categories and themes. Attention will be paid to negative, or 'deviant', cases to inform developing themes and interpretation. Short summaries of each interview will be also written to explore how individual

For peer review only

experiences and views of the intervention may differ between participants. The analysis and interpretation of the data will be regularly discussed with the research team. A detailed record will be kept of the analysis process, including definitions of the themes and concepts and their application.

Discussion

We will interpret the findings of this feasibility trial and report the implications for progression to a definitive randomised controlled trial of the HPC programme. This will include any necessary amendments to the intervention content and delivery, as well as the development of a train-the-trainer manual to be used in training future facilitators of the programme. The following indicators of feasibility will be used to determine whether a definitive randomised controlled trial is feasible with the current trial design and procedures:

a) Recruit a minimum of 48 participants, which is the minimum number that will enable all six sites to be randomised and the intervention to be tested
b) Deliver 6 groups in total for the intervention arm, assessed by establishing 6 venues, and identifying and training facilitators, and groups completing the programme curriculum
c) At least 80% of participants completing measures at 6-month follow-up or a clear plan to achieve this in the trial

If any of these indicators are not met, the research team will consider whether a definitive trial may not be feasible or whether changes to the design or procedures and further feasibility testing are needed. The need to translate programme materials into other languages will also be taken into account for a subsequent trial.

A complete and transparent report of the trial will be produced with reference to recommendations of the CONSORT 2010 statement: extension to randomised pilot and feasibility trials, including a CONSORT participant flow chart [54]. The report will be written for publication in a peer-reviewed, open access, academic journal with authorship eligibility determined by following the International Committee of Medical Journal Editors recommendations [56]. A plain language summary of the findings will also be co-produced with members of our Family Faculty public involvement group and sent to trial participants and organisations that help to recruit participants and host the groups. We will consult our Family Faculty and Stakeholder Advisory Group for advice on ways to disseminate the findings.

NHS England's Commitment to Carers states 'Helping carers to provide better care and to stay well themselves will contribute to better lives for those needing care and more effective use of NHS resources' [57]. However, there is currently a paucity of interventions that promote health for parent carers. This feasibility trial and a subsequent definitive trial may have important implications for a public health strategy for parent carers of children with disabilities in the UK. It will also inform research and public health policy internationally, as the higher risk of psychological and physical health problems in parent carers is not limited to the UK.

Project timetable and milestones

The main milestones are as follows. Ethical approval for the study was received on 20 August 2018. The trial was registered on 25 October 2018 (ISRCTN 15144652). Recruitment of participants began on 29 October 2018. The analysis of data on fidelity and process evaluation will be conducted following data collection (summer to autumn 2019). The analyses of outcome measures will be conducted in February 2020. The expected date of completion is June 2020.

Supplementary information

Supplementary information accompanies this paper at https://doi.org/10.1186/s40814-019-0517-3.

Additional file 1. SPIRIT 2013 Checklist: Recommended items to address in a clinical trial protocol and related documents*.

Abbreviations

AMC-19: About my Child measure; CDC: Council for Disabled Children; CLANGERS: Connect, Learn, be Active, Notice, Give, Eat well, Relax, and Sleep; CONSORT: Consolidated Standards of Reporting Trials; DIRUM: Database of Instruments for Resource Use Management; EQ-5D-5 L: EuroQol 5 Dimensions; HPAS: Health Promoting Activities Scale; HPC: Healthy Parent Carers; ICECAP-A: ICEpop CAPability measure for Adults; NIHR: National Institute for Health Research; PAM: Patient Activation Measure; PAPF: Parents' Assessment of Protective Factors; PenCLAHRC: The National Institute for Health Research (NIHR) Collaboration for Leadership in Applied Health Research and Care (CLAHRC) South West Peninsula; PenCRU: Peninsula Cerebra Research Unit; PHQ-9: Patient Health Questionnaire-9; PPI: Patient and public involvement; SAG: Stakeholder Advisory Group; SEND: Special educational needs and disability; WEMWBS: Warwick-Edinburgh Mental Well-being Scale

Acknowledgements

We thank members of the PenCRU Family Faculty and the Stakeholders Advisory Group for their involvement in the development of the intervention and the design of this study.

Study Sponsor

University of Exeter, Research Ethics and Governance Office, University of Exeter, Lafrowda House, St Germans Road, Exeter, Devon, EX4 6TL

Authors' contributions

All authors contributed. CM led the development and preliminary evaluation of the programme and leads the study and public involvement work. GB and CM drafted the initial study design. AB, AM, MF, and CM designed the original programme. OU designed the quantitative analysis plan. AH designed the methods to develop and test a cost-effectiveness framework. AB, CM, VB, JL, MT, KW, and BCF designed the fidelity and process evaluation. KF facilitated public involvement. SR coordinated the CTU involvement in the design and conduct of the project. SL facilitated infrastructure support through National Institute for Health Research Applied Research

For peer review only - http://bmjopen.bmj.com/site/about/guidelines.xhtml

Collaboration South West Peninsula. All authors contributed to drafting and refining the study protocol and approved the final manuscript. The study Sponsor is the University of Exeter.

Funding

This paper presents independent research funded by the National Institute for Health Research (NIHR) under its Research for Patient Benefit (RfPB) Programme (Grant Reference Number PB-PG-0317-20044). We also acknowledge support from the National Institute for Health Research Applied Research Collaboration South West Peninsula. The views and opinions expressed in this paper are those of the authors and not necessarily those of the National Institute for Health Research or the Department of Health and Social Care. Delivery of the Healthy Parent Carers programme is funded by Public Health England, Kernow Clinical Commissioning Group, Northern, Eastern and Western Devon Clinical Commissioning Group, and a grant from the National Lottery Community Fund through the Reaching Communities programme. Peer review of the study design was provided during the NIHR funding application process. The funders have had no involvement in the writing of the manuscript and will not have any role during its execution, analyses, interpretation of the data, writing of the report, or the decision to submit the report for publication.

Availability of data and materials

Not applicable

Ethics approval and consent to participate

Ethics approval for the study was received on 20 August 2018 from the University of Exeter Medical School Research Ethics Committee in the College of Medicine and Health (UEMS REC 18/06/174); recruitment is currently ongoing.

Consent for publication

Not applicable

Competing interests

The authors declare that they have no competing interests.

Author details

[1]Peninsula Childhood Disability Research Unit (PenCRU) and National Institute for Health Research Applied Research Collaboration South West Peninsula (PenARC), University of Exeter Medical School, University of Exeter, St. Luke's Campus, Heavitree Road, Exeter EX1 2LU, UK. [2]Nuffield Department of Primary Care Health Sciences, Medical Sciences Division, Radcliffe Observatory Quarter, University of Oxford, Woodstock Road, Oxford OX2 6GG, UK. [3]National Institute for Health Research Applied Research Collaboration South West Peninsula (PenARC), University of Exeter Medical School, University of Exeter, St. Luke's Campus, Heavitree Road, Exeter EX1 2LU, UK. [4]Health Economics Group, University of Exeter Medical School, University of Exeter, St. Luke's Campus, Heavitree Road, Exeter EX1 2LU, UK. [5]PenCRU Family Faculty, University of Exeter Medical School, University of Exeter, St. Luke's Campus, Heavitree Road, Exeter EX1 2LU, UK. [6]Exeter Clinical Trials Unit, University of Exeter Medical School, University of Exeter, St. Luke's Campus, Heavitree Road, Exeter EX1 2LU, UK.

Received: 2 April 2019 Accepted: 15 October 2019
Published online: 23 November 2019

References

1. Blackburn CM, Spencer NJ, Read JM. Prevalence of childhood disability and the characteristics and circumstances of disabled children in the UK: secondary analysis of the Family Resources Survey. BMC Pediatr. 2010;10(21): 1471–2431.
2. Barlow J, Cullen-Powell L, Cheshire A. Psychological well-being among mothers of children with cerebral palsy. Early Child Dev Care. 2006;176(3–4):421–8.
3. Brehaut JC, Kohen DE, Raina P, Walter SD, Russell DJ, Swinton M, O'Donnell M, Rosenbaum P. The health of primary caregivers of children with cerebral palsy: how does it compare with that of other Canadian caregivers? Pediatrics. 2004;114(2):e182–91.
4. Brehaut JC, Kohen DE, Garner RE, Miller AR, Lach LM, Klassen AF, Rosenbaum PL. Health among caregivers of children with health problems: findings from a Canadian population-based study. Am J Public Health. 2009; 99(7):1254–62.
5. Emerson E. Mothers of children and adolescents with intellectual disability: social and economic situation, mental health status, and the self-assessed social and psychological impact of the child's difficulties. J Intellect Disabil Res. 2003;47(4–5):385–99.
6. Gallagher S, Phillips AC, Oliver C, Carroll D. Predictors of psychological morbidity in parents of children with intellectual disabilities. J Pediatr Psychol. 2008;33(10):1129–36.
7. Lach LM, Kohen DE, Garner RE, Brehaut JC, Miller AR, Klassen AF, Rosenbaum PL. The health and psychosocial functioning of caregivers of children with neurodevelopmental disorders. Disabil Rehabil. 2009;31(9):741–52.
8. Murphy NA, Christian B, Caplin DA, Young PC. The health of caregivers for children with disabilities: caregiver perspectives. Child Care Health Dev. 2007;33(2):180–7.
9. Oelofsen N, Richardson P. Sense of coherence and parenting stress in mothers and fathers of preschool children with developmental disability. J Intellect Dev Disabil. 2006;31(1):1–12.
10. Olsson MB, Hwang C. Depression in mothers and fathers of children with intellectual disability. J Intellect Disabil Res. 2001;45(6):535–43.
11. Singer GH, Floyd F. Meta-analysis of comparative studies of depression in mothers of children with and without developmental disabilities. Am J Ment Retard. 2006;111(3):155–69.
12. Tong HC, Haig AJ, Nelson VS, Yamakawa KS-J, Kandala G, Shin KY. Low back pain in adult female caregivers of children with physical disabilities. Arch Pediatr Adolesc Med. 2003;157(11):1128–33.
13. Lee M, Park C, Matthews AK, Hsieh K. Differences in physical health, and health behaviors between family caregivers of children with and without disabilities. Disabil Health J. 2017;10:565–70.
14. Arim RG, Miller AR, Kohen DE, Guèvremont A, Lach LM, Brehaut JC. Changes in the health of mothers of children with neurodevelopmental disabilities: an administrative data study. Res Dev Disabil. 2019;86:76–86.
15. Brehaut JC, Garner RE, Miller AR, Lach LM, Klassen AF, Rosenbaum PL, Kohen DE. Changes over time in the health of caregivers of children with health problems: growth-curve findings from a 10-year Canadian population-based study. Am J Public Health. 2011;101(12):2308–16.
16. Resch JA, Mireles G, Benz MR, Grenwelge C, Peterson R, Zhang D. Giving parents a voice: a qualitative study of the challenges experienced by parents of children with disabilities. Rehabil Psychol. 2010;55(2):139–50.
17. Council for Disabled Children. Expert Parent Programme. https://councilfordisabledchildren.org.uk/our-work/whole-child/practice/expert-parent-programme. Accessed 21 Jan 2019.
18. Singer GH, Ethridge BL, Aldana SI. Primary and secondary effects of parenting and stress management interventions for parents of children with developmental disabilities: a meta-analysis. Dev Disabil Res Rev. 2007;13(4):357–69.
19. Dykens EM, Fisher MH, Taylor JL, Lambert W, Miodrag N. Reducing distress in mothers of children with autism and other disabilities: a randomized trial. Pediatrics. 2014;134(2):e454–63.
20. Shilling V, Morris C, Thompson-Coon J, Ukoumunne O, Rogers M, Logan S. Peer support for parents of children with chronic disabling conditions: a systematic review of quantitative and qualitative studies. Dev Med Child Neurol. 2013;55(7):602–9.
21. Law E, Fisher E, Eccleston C, Palermo TM. Psychological interventions for parents of children and adolescents with chronic illness. Cochrane Database of Systematic Reviews 2019, Issue 3. Art. No.: CD009660. https://doi.org/10.1002/14651858.CD009660.pub4.
22. Cachia RL, Anderson A, Moore DW. Mindfulness, stress and well-being in parents of children with autism spectrum disorder: a systematic review. J Child Fam Stud. 2016;25(1):1–14.
23. Jetten J, Haslam C, Alexander SH. The social cure: identity, health and well-being: Psychology press; 2012.
24. Borek A, Abraham C, Greaves C, Gillison F, Tarrant M, Morgan-Trimmer S, McCabe R, Smith J: Identifying change processes in group-based health behaviour-change interventions: Development of the Mechanisms of Action in Group-based Interventions (MAGI) framework. Health Psychol Rev. 2019: 1–21. https://doi.org/10.1080/17437199.2019.1625282.
25. Tarrant M, Khan SS, Farrow CV, Shah P, Daly M, Kos K. Patient experiences of a bariatric group programme for managing obesity: a qualitative interview study. Br J Health Psychol. 2017;22(1):77–93.

For peer review only

26. Haslam C, Cruwys T, Haslam SA, Dingle G, Chang MX-L. Groups 4 Health: evidence that a social-identity intervention that builds and strengthens social group membership improves mental health. J Affect Disord. 2016;194:188–95.
27. Peninsula Childhood Disability Research Unit. Our Family Faculty. http://www.pencru.org/getinvolved/ourfamilyfaculty/. Accessed 29 Jan 2019.
28. Bartholomew LK, Parcel GS, Kok G, Gottlieb NH, Fernandez ME. Planning health promotion programs: an intervention mapping approach. 4th ed. San Francisco: Jossey-Bass; 2016.
29. Borek AJ, McDonald B, Fredlund M, Bjornstad G, Logan S, Morris C. Healthy Parent Carers programme: development and feasibility of a novel group-based health-promotion intervention. BMC Public Health. 2018;18(1):270.
30. Muehlhausen W, Doll H, Quadri N, Fordham B, O'Donohoe P, Dogar N, Wild DJ. Equivalence of electronic and paper administration of patient-reported outcome measures: a systematic review and meta-analysis of studies conducted between 2007 and 2013. Health Qual Life Outcomes. 2015;13(1):167.
31. Hammond P. Staying alive: how to get the best out of the NHS - advice from a doctor. London: Quercus; 2015.
32. Aked J, Marks N, Cordon C, Thompson S. Five ways to wellbeing: a report presented to the foresight project on communicating the evidence base for improving people's well-being. London: Nef; 2009.
33. Williams U, Rosenbaum P, Gorter JW, McCauley D, Gulko R. Psychometric properties and parental reported utility of the 19-item 'About My Child' (AMC-19) measure. BMC Pediatr. 2018;18(1):174.
34. Department for Communities and Local Government. English indices of deprivation 2015. https://www.gov.uk/government/statistics/english-indices-of-deprivation-2015. Accessed 12 May 2017.
35. Tennant R, Hiller L, Fishwick R, Platt S, Joseph S, Weich S, Parkinson J, Secker J, Stewart-Brown S. The Warwick-Edinburgh mental well-being scale (WEMWBS): development and UK validation. Health Qual Life Outcomes. 2007;5(1):63.
36. National Institute for Health and Care Excellence. The social care guidance manual. London: NICE; 2016.
37. Kroenke K, Spitzer RL. The PHQ-9: a new depression diagnostic and severity measure. Psychiatr Ann. 2002;32(9):509–15.
38. Kroenke K, Spitzer RL, Williams JB, Löwe B. The patient health questionnaire somatic, anxiety, and depressive symptom scales: a systematic review. Gen Hosp Psychiatry. 2010;32(4):345–59.
39. Wittkampf KA, Naeije L, Schene AH, Huyser J, van Weert HC. Diagnostic accuracy of the mood module of the Patient Health Questionnaire: a systematic review. Gen Hosp Psychiatry. 2007;29(5):388–95.
40. National Collaborating Centre for Mental Health, Royal College of Psychiatrists. Common mental health disorders: identification and pathways to care: RCPsych Publications; 2011.
41. National Institute for Health and Care Excellence. Depression in adults: recognition and management. https://www.nice.org.uk/guidance/cg90. Accessed 21 Jan 2019.
42. The EuroQol Group. EuroQol-a new facility for the measurement of health-related quality of life. Health Policy. 1990;16(3):199–208.
43. Kiplinger VL, Browne CH. Parents' assessment of protective factors: user's guide and technical report. Washington: Center for the Study of Social Policy; 2014.
44. Bourke-Taylor H, Law M, Howie L, Pallant JF. Initial development of the Health Promoting Activities Scale to measure the leisure participation of mothers of children with disabilities. Am J Occup Ther. 2012;66(1):e1–e10.
45. Muskett R, Bourke-Taylor H, Hewitt A. Intrarater reliability and other psychometrics of the Health Promoting Activities Scale (HPAS). Am J Occup Ther. 2017;71(4):7104190010p7104190011–8.
46. Hibbard JH, Mahoney ER, Stockard J, Tusler M. Development and testing of a short form of the patient activation measure. Health Serv Res. 2005; 40(6p1):1918–30.
47. Hibbard JH, Stockard J, Mahoney ER, Tusler M. Development of the Patient Activation Measure (PAM): conceptualizing and measuring activation in patients and consumers. Health Serv Res. 2004;39(4p1):1005–26.
48. Al-Janabi H, Flynn TN, Coast J. Development of a self-report measure of capability wellbeing for adults: the ICECAP-A. Qual Life Res. 2012;21(1):167–76.
49. Al-Janabi H, Peters TJ, Brazier J, Bryan S, Flynn TN, Clemens S, Moody A, Coast J. An investigation of the construct validity of the ICECAP-A capability measure. Qual Life Res. 2013;22(7):1831–40.
50. Mitchell PM, Al-Janabi H, Byford S, Kuyken W, Richardson J, Iezzi A, Coast J. Assessing the validity of the ICECAP-A capability measure for adults with depression. BMC Psychiatry. 2017;17(1):46.
51. Ridyard CH, Hughes DA, Team D. Development of a database of instruments for resource-use measurement: purpose, feasibility, and design. Value Health. 2012;15(5):650–5.
52. Moore GF, Audrey S, Barker M, Bond L, Bonell C, Hardeman W, Moore L, O'Cathain A, Tinati T, Wight D. Process evaluation of complex interventions: Medical Research Council guidance. BMJ. 2015;350:h1258.
53. Ridyard CH, Hughes DA. Taxonomy for methods of resource use measurement. Health Econ. 2015;24(3):372–8.
54. Eldridge SM, Chan CL, Campbell MJ, Bond CM, Hopewell S, Thabane L, Lancaster GA. CONSORT 2010 statement: extension to randomised pilot and feasibility trials. Pilot Feasibility Stud. 2016;2(1):64.
55. Braun V, Clarke V. Using thematic analysis in psychology. Qual Res Psychol. 2006;3(2):77–101.
56. International Committee of Medical Journal Editors. Defining the role of authors and contributors. http://www.icmje.org/recommendations/browse/roles-and-responsibilities/defining-the-role-of-authors-and-contributors.html. Accessed 18 Mar 2019.
57. NHS England: NHS England's commitment to carers; 2014.

Publisher's Note

Springer Nature remains neutral with regard to jurisdictional claims in published maps and institutional affiliations.

Ready to submit your research? Choose BMC and benefit from:

- fast, convenient online submission
- thorough peer review by experienced researchers in your field
- rapid publication on acceptance
- support for research data, including large and complex data types
- gold Open Access which fosters wider collaboration and increased citations
- maximum visibility for your research: over 100M website views per year

At BMC, research is always in progress.

Learn more biomedcentral.com/submissions

BMC

For peer review only

Facilitator training feedback form

During this phase of the research we are still developing and finalising the facilitator training. Your feedback will help us to ensure that any future training delivered is as comprehensive and useful as possible.

Your feedback is anonymous and you will not be identified in any research reports. This information will only be viewed by the research team.

1. Please rate, on a scale of 1-5, your knowledge and understanding, and skills and confidence in the following areas (with 1 being very little and 5 being a lot).

**Knowledge and understanding of:**	**1**	**2**	**3**	**4**	**5**
The intervention's theoretical framework (CLANGERS); the session activities and associated learning outcomes.					
Group facilitation techniques and how to create a positive group atmosphere.					
Particular challenges faced by parent carers and how this may impact on health and wellbeing.					
The role of the HPC Lead/Assistant Facilitator.					
The study design.					
Safeguarding procedures.					

**Skills and confidence**	**1**	**2**	**3**	**4**	**5**
Ability to confidently present programme information, lead activities and facilitate discussions.					
Ability to create a positive group atmosphere and inspire collective desire to make lifestyle changes to improve health and wellbeing.					
Ability to manage time effectively.					
Ability to manage difficult/sensitive issues that may arise.					

For peer review only

2. The time allotted for the training was sufficient** (**Please tick one)

Strong Agree	Agree	Neutral	Disagree	Strongly Disagree

3. Are there any aspects of the training that could be improved (e.g. content, time provided to cover the learning objectives and/or delivery)?

4. Any other comments?

Many thanks for taking the time to give your feedback.

For peer review only

Healthy
PARENT CARERS

CHECKLIST FOR MODULE 1 DELIVERY

Instructions to the Facilitators and Assistant Facilitators:
Please complete this checklist at the end of each session to provide us with feedback on what was covered and how the session went. This feedback will help us assess if the sessions can be delivered as planned and identify potential challenges and improvements.

Date & time of the session: ____

Session number: ________________________

Venue: ____

Facilitators' names: ____

Session duration (as delivered): _______________________

Number of participants attending: _____________________

**Activities**	**Delivered** (Please tick if delivered)	**Comment** (If partly or not delivered, please explain why)
1. Introductions / icebreaker activity		
2. Hopes and fears		
3. Ground rules		
4. What are health and wellbeing?		
5. What influences health and wellbeing?		
6. The resilience game		
7. Conclusion		
Time for informal interaction (e.g. tea break)		

For peer review only - http://bmjopen.bmj.com/site/about/guidelines.xhtml

Reflections on the session

Please indicate which picture best describes your perception of how well connected, in general, the attendees of today's session appeared to be (tick the box on the right of the picture).

For example, the first picture would suggest that there was very little connection between individuals – that they did not come together as a group at all. Whereas the final picture would suggest that individuals were very closely connected – they very much came together as a group.

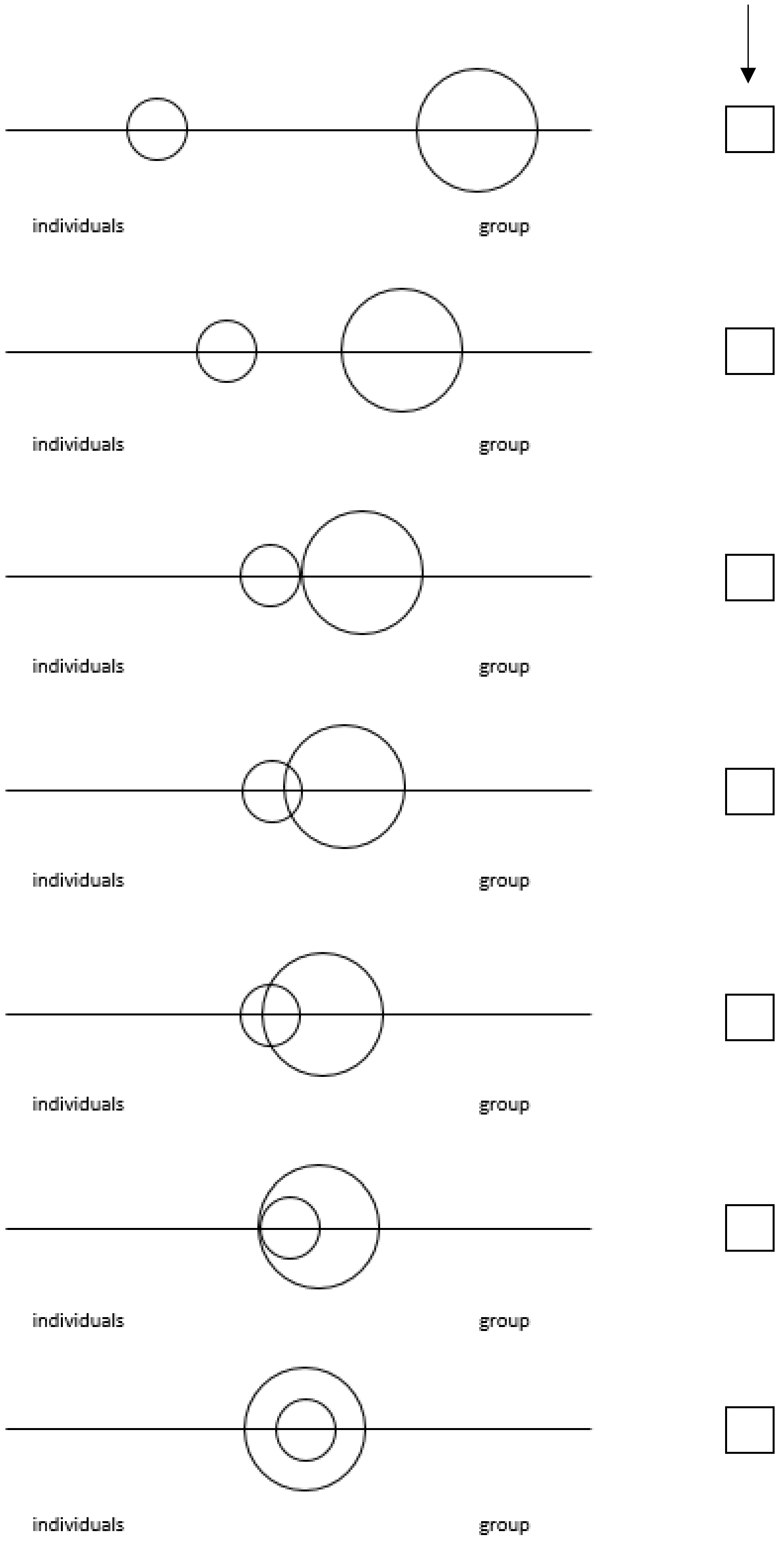

<u>Reflections on the session cont.</u>

<u>Please circle one</u>

In your opinion, how well did today's session go?

Not well at all				Very well
1	2	3	4	5

How engaged were the participants in today's session?

Not well at all				Very well
1	2	3	4	5

Do you have any comments or reflections?

(For example, related to how the group worked together, participants' engagement, session content or activities)

For peer review only

For peer review only - http://bmjopen.bmj.com/site/about/guidelines.xhtml

Draft topic guide for a focus group with HPC Facilitators

Topics and example questions for the focus group with HPC Facilitators and Assistant Facilitators:

- Motivation for / expectations of delivering the group programme, e.g.:
  - Why did you decide to get involved in this study?
  - What did you expect from taking part in the study / delivering the programme?
- Views about training / preparation to deliver the programme, e.g.:
  - How did you find the training to deliver the programme? (Prompt about whether it was sufficient, anything that would be helpful)
  - How prepared did you feel to deliver the programme? (Prompt about their background and previous experience, or any new skills developed by delivering HPC)
- Experience of delivering the programme / group facilitation, e.g.:
  - Having delivered the programme now, what do you think about it?
  - Generally, how did you find delivering the programme? (Prompt about following the Facilitator Manual, any adaptations made, challenges, how feasible it was to deliver)
  - How engaged was the group / the participants?
  - How did you find facilitating / managing the group? (Prompt about any challenges and how they managed those, how engaged they were in delivering it)
- What worked best in the programme? Why? (Prompt about activities, content, group)
- What didn't work well? What were the challenges or difficulties? Why?
- Do you have any suggestions for improvements? (e.g. related to the programme delivery, content, Facilitator Manual, training)
- Is there anything else that we haven't talked about and that you'd like to discuss?

For peer review only

Healthy PARENT CARERS

HEALTHY PARENT CARERS
WEEKLY PARTICIPANT FEEDBACK FORM FOR GROUP INTERVENTION

GROUP: ____

SESSION NUMBER: ____________________________________

We would like to know what you think about this session and would be grateful for your feedback. The feedback is anonymous and it will help us refine and improve the programme in future.

Please circle one

1. Overall, how satisfied are you with today's session? (please circle one)

Not satisfied at all				Very satisfied
1	2	3	4	5

2. Do you feel that you were able to take an active part in the session? (please circle one)

Not at all				Very much
1	2	3	4	5

3. Please tell us about the best things in today's session (e.g. most enjoyed or most helpful):

4. Please tell us about the things that could be improved in today's session:

For peer review only

5. Which picture best describes how well connected, in general, you felt to the rest of the group today?

For example, the first picture would suggest that you felt very little connection to the rest of the group, whereas the final picture would suggest that you felt very connected to the group.

Please tick the box on the right hand side of the picture that best describes how you felt.

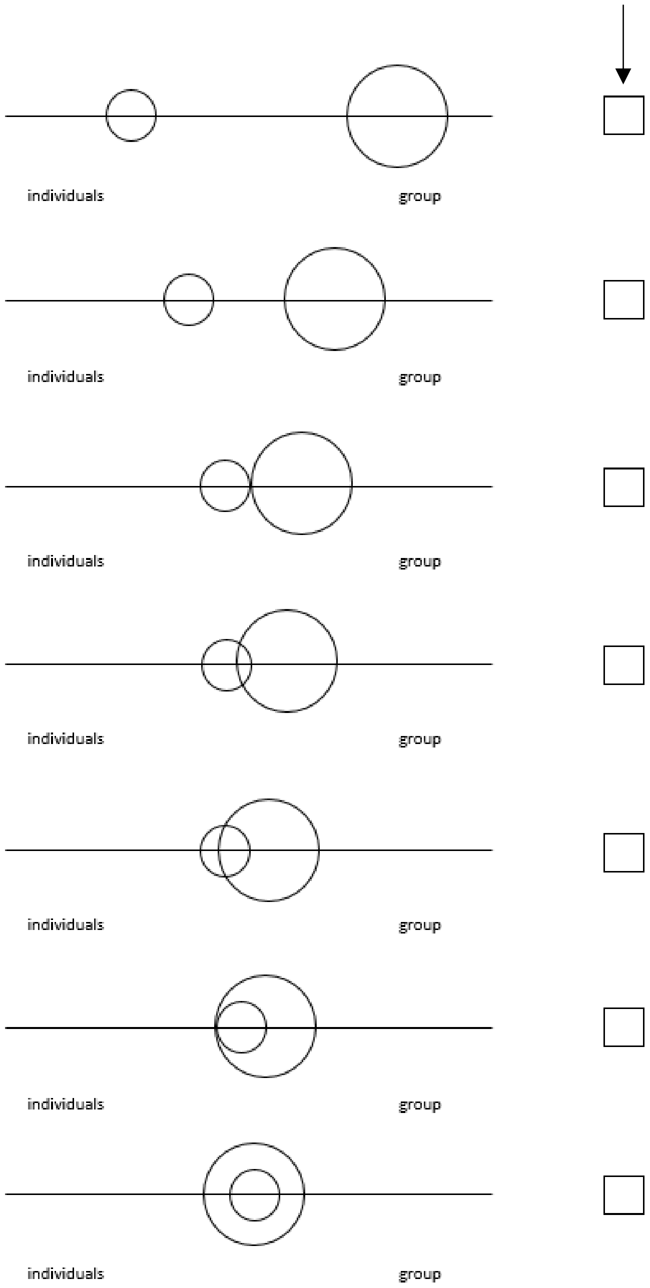

6. How much did you identify with other members of this group? (please circle one)

Not at all				Very much
1	2	3	4	5

HEALTHY PARENT CARERS
Feedback FORM

We would like to know what you think about the Healthy Parent Carers programme and the research study and would be grateful for your feedback. The feedback is anonymous and it will help us refine and improve the programme and our research in future.

1. Overall, how satisfied are you with taking part in the Healthy Parent Carers programme?

Not satisfied at all ... Very satisfied
1 2 3 4 5

2. How useful has the programme been in helping you improve your health and wellbeing?

Not at all ... Very much
1 2 3 4 5

Was there anything in particular that helped you?

3. Have you made any changes as a result of taking part in the programme? No / Yes

If yes, what?

If no, why?

4. Approximately how many of the online materials have you accessed?

None	25%	50%	More than 50%

5. Did you attend group sessions for the programme? Yes / No

If yes, reveal questions 6-10, then continue from question 12.

If no, reveal question 11, then continue from question 12.

6. How would you rate the length of the individual sessions?

Too short	About right	Too short

7. Did you miss any of the group sessions? Yes / No

If yes, why?

8. Overall, how much did you feel included and part of the group?

Not at all				Very much
1	2	3	4	5

9. How satisfied were you with the way the sessions were facilitated and delivered?

Not satisfied at all				Very satisfied
1	2	3	4	5

10. Did you discuss the programme with anyone who had access to the online materials only (i.e. they did not attend the group sessions)? Yes / No

11. Did you discuss the programme with anyone who attended the group sessions? Yes / No

12. Do you have any suggestions about how the programme or materials could be improved?

13. Would you recommend this programme to other parent carers?

Not at all				Very much
1	2	3	4	5

14. Do you have any other comments, reflections or suggestions?

For peer review only

Appendix 2: Data collection materials

Follow-up questionnaire
Version 1, 11 June 2019

HEALTHY PARENT CARERS
Follow-up questionnaire

1. Have you continued to access the online resources in the past 6 months?

Yes / No

2. Approximately how many of the online materials have you accessed in total?

None	25%	50%	More than 50%

3. Did you attend group sessions for the programme? Yes / No

If yes, reveal question 4.

If no, end survey.

4. Since the Healthy Parent Carers group ended, how many members of your group have you been in touch with (including members that you knew prior to starting)?

(number up to 10)

5. Out of these, how many were people that you did not know before the programme?

(number up to 10)

6. Since the group ended, have you been in touch with any members the group that you didn't know before in any of the following ways?

(select all that apply)

Phone

Text

Email

WhatsApp

Other Social media (e.g. Facebook, Twitter)

Meeting in person

Other (please specify – please do not include anyone's name) (free text box needed if ticked)

For peer review only - http://bmjopen.bmj.com/site/about/guidelines.xhtml

Appendix 2: Data collection materials

Follow-up questionnaire
Version 1, 11 June 2019

I have not been in touch with any of the group members that I didn't know before

7. Approximately how long ago was the last time you had contact with any members of the group that you didn't know before it started (please do not include anyone's name)?

(free text)

8. Can you please describe how often you have been in touch with any members of the group that you didn't know before (please do not include anyone's name)?

(free text)

9. How many of these new contacts would you now say are your friends?

(number up to 10)

For peer review only - http://bmjopen.bmj.com/site/about/guidelines.xhtml

Healthy
PARENT CAREERS

Draft interview topic guide: HPC Participants (intervention arm)

- Introduce myself (PenCRU childhood disability research unit)
- The aim of the interview is to get their feedback on the HPC programme and research study
- Confirm how much time interviewee has for interview. Confirm that meant to take about 30 mins but can vary depending on length of discussion – let me know if you have somewhere you need to be and we can manage accordingly
- Remind participant that taking part is voluntary – if I ask about something you don't want to talk about, please just say you'd rather not go in to that
- Remind participants that the interview will be recorded and transcribed and analysed to help us understand how the programme and study worked. Interview transcripts will be anonymous.
- Check participant is comfortable (has drink of water etc.) and has given consent to continue

Record before/after the interview:

- Participant ID:
- Participant's group ID & facilitators' IDs:
- Gender:
- Age:

Topics and example questions for participants in the intervention arm:

- Motivation to participate & expectations, e.g.:
    - Why did you decide to take part in the study / programme?
    - Was there anything that helped you to decide to take part?
    - What did you expect from taking part in the study?
- Understanding of, and views on, the study design, e.g.:
    - How sufficient did you find the information about the study & the programme and what they involve?
    - What did you think when you found out that you were assigned to the group programme, rather than only being given access to online materials?
    - Would you change any aspect of how it was explained to you, or what information was available, about the programme or the research? (probe suggestions from them)
- Experience of the group programme (activities, group):
    - What do you think about the content of the programme? (prompt about relevance "relevant to you as a parent carer", helpfulness of the content e.g. CLANGERS)
- Views on length & frequency of sessions; any missed sessions & reasons for missing sessions
    - What do you think about the length and frequency of the sessions? Did you need to miss any sessions? (prompt about reasons for missing sessions)
- Views on & engagement with the group:
    - Tell me about your group. Did you feel included and a part of it?
- Views about facilitators and delivery style, e.g.:

For peer review only

- What do you think about the facilitators and the way they delivered the programme? How well did they do in delivering the programme and facilitating the group? (prompt about facilitator engagement)

- Usage of, and views on, the online programme materials
  - Did you access the programme materials online? (prompt about how they used them/how often, or about reasons for not using them) If yes, what prompted you to look at them?
- Impact of the programme & any changes made, e.g.:
  - How helpful did you find the programme? Did you make any changes in result of the programme? (prompt for examples) Was there anything that got in the way of you being able to make those changes? Was there anything that supported you to make those changes?
- Contextual influences, e.g.:
  - Did anything affect how much you got out of participating in the programme? Were there any barriers to you engaging with or benefiting from the programme?
- Best & worst things, suggestions for improvements, e.g.:
  - What did you like most / found most helpful (if anything) about the programme?
  - What did you not like / did not find helpful (if anything)?
  - Do you have any suggestions for how the programme could be improved? (prompts: delivery, content, group, timing / length of programme)
- Acceptability of data collection and measures, e.g.:
  - How did you find having to fill in all the questionnaires at the beginning and at the end of the study? (prompt about time, clarity, relevance)
  - The sessions were audio recorded. How would you have felt if the sessions had been video recorded instead?
- Is there anything else that we haven't talked about and that you'd like to mention?

For peer review only

Healthy PARENT CARERS

Draft interview topic guide: HPC Participants (control arm)

- Introduce myself (PenCRU childhood disability research unit)
- The aim of the interview is to get their feedback on the HPC programme and research study
- Confirm how much time interviewee has for interview. Confirm that meant to take about 30 mins but can vary depending on length of discussion – let me know if you have somewhere you need to be and we can manage accordingly
- Remind participant that taking part is voluntary – if I ask about something you don't want to talk about, please just say you'd rather not go in to that
- Remind participants that the interview will be recorded and transcribed and analysed to help us understand how the programme and study worked. Interview transcripts will be anonymous.
- Check participant is comfortable (has drink of water etc.) and has given consent to continue

Record before/after the interview:

- Participant ID:
- Gender:
- Age:

Topics and example questions for participants in the control arm:

- Motivation / expectations to participate, e.g.:
    - Why did you decide to take part in the study / programme?
    - Was there anything that helped you to decide to take part?
    - What did you expect from taking part in the study?
- Understanding of, and views on, the randomised controlled design and control group, e.g.:
    - How sufficient did you find the information about the study and the programme, and what they involve?
    - What did you think when you found out that you were assigned to the group with access to online materials only and not the group programme?
    - Would you change any aspect of how it was explained to you, or what information was available, about the programme or the research?
- Contamination with the intervention arm, e.g.:
    - Do you know anyone who also took part in the study and attended a group? (If yes, prompt about how much they discussed what happened in the group and how much that might have influenced their view on the programme materials or changes made).
- Usage of, and views on, the online programme materials, e.g.:
    - Did you access the programme materials online? (prompt about how they used them/how often, or about reasons for not using them)
    - What do you think about them?
- Impact of the programme, any changes made, e.g.:

For peer review only

- How helpful did you find the materials? Did you make any changes in result? (examples) Was there anything that got in the way of you being able to make those changes? Was there anything that supported you to make those changes?
- Views on the data collection and measures
    - How did you find having to fill in all the questionnaires at the beginning and at the end of the study? (prompt about time, clarity, relevance)
- Best & worst things, suggestions for improvements, e.g.:
    - What did you like most / found most helpful (if anything) about the programme?
    - What did you not like / did not find helpful (if anything)?
    - Do you have any suggestions for how the materials or the research elements could be improved?

Is there anything else that we haven't talked about and that you'd like to mention?

For peer review only

Supplementary Document 11. Additional quotes and comments

1. Facilitators' experiences

1.1. Motivations and expectations

New experiences, new skills & being involved in the research project. (Facilitator pre-training questionnaire)

Recognised the need for healthy wellbeing of parent carers through myself and friends. Facilitate a small support group locally. Ready for a new challenge. (Facilitator pre-training questionnaire)

To apply my training delivery skills and experience to a programme which is unique in its focus on parent carer health, something myself as a parent carer feel I could directly benefit from. (Facilitator pre-training questionnaire)

CLANGERS has changed my life and I feel it is really important to help others by spreading the message. It's also a research project and that excites me as well! (Facilitator pre-training questionnaire)

I always believed in this course because it had such a positive impact on me and I just wanted to give back to others. (AF, focus group)

When we did the [training], I do remember thinking some of this is really resonating with me and my own challenges and thinking actually, I can see where this is going, and it has been really, really good. (LF, focus group)

1.2. Training, preparation and support

It became clear to me that there was actually quite a distinctive style that was expected as well… we would all have liked some more concrete direction in terms of what to do and how. (AF, focus group)

It's something actually a little bit more fundamental about that. It's about why are you writing this stuff down? Because there's nothing in the trainer notes that explained to the trainer what the purpose of having this stuff written down is. I always find it difficult delivering training that's been written by somebody else… I had to keep saying in my head, 'do it as it's written, don't go off and do your own thing'. (LF, focus group)

I would [practise] 'Connect' because it would give you the opportunity to identify some of the issues that are going to come up, because 'connect' can be really difficult for parent carers. So you could use it as, well this is an example of somebody who struggles, how would you do it in this situation, how would you cope with this? So it can be quite an emotional one and it's also the first one that we do in the programme… The group were also very nervous and quiet in that one, so that makes it harder. (AF, focus group)

LF: …trainers probably need to undergo the training in the first instance because I think you need to experience the programme, both to have a better understanding but you would get a lot of ideas and facilitation through that.

For peer review only

AF: I felt I was at an advantage because I had done it… because I knew the course I was able to listen and scribe, knew what was coming up because I'd done it before, but I was also able to input in my way and from my experiences… which I felt helped. So it wasn't new information to me, I'd already gone through that quite hard process of thinking and I had already adjusted and adapted and was aware of it and everything else. And you could see it on the faces of the participants that actually it was hard at times and I had been there, so I understood. (Focus group)

More time for assistants to get a clearer understanding of breath of exercises / module content. (Assistant training, feedback form)

It's all very hurried. More time practicing our role. (Refresher training, feedback form)

1.3. Programme content and group delivery

It's tricky to manage but more difficult with some groups than others, definitely… but I think for the most part parents actually said it was really nice to focus on themselves. (LF, focus group)

LF: I would be very tempted to remove SMART from there and just replace it with achievable.
AF: The most important thing about CLANGERS is that you take ownership of it and you become aware that that's what you're doing. So it's important to have those goals but it's also important to recognise when you're not achieving those goals, where did it fall down. (…) so actually you might not have achieved that goal but you've actually done bits of CLANGER here instead. So it's just raising that awareness and your own understanding and building up that way. (Focus group)

The fact that we had such small groups was really disappointing for me… I think it changed the nature of the course, being such small groups, it was far more intense, I believe, for the training team, than it would have been if we'd had lots of participants there who were able to share their views and other things. So yes, altogether a highly enjoyable and positive experience but a few things that, perhaps, my expectations were slightly different. (LF, focus group)

I think the key of the leaders being parents of children with disabilities just really, really helps everybody to bond and understand. For me, I'm like wow, you've been through it, you're this much further on the same journey that I'm on and look at you; you're out here and you're doing this and you're working and, oh, your life is okay… That's reassuring. (AF, focus group)

All participants were very engaged. Unfortunately, one has a lot of problems currently so found it difficult to discuss resilience without being extremely emotional. Other participants very supportive. (Facilitators session checklist)

Group extremely connected. Tearful at the end of evening. Have made arrangements for continued contact as a group. Great sharing + reflection of course. (Facilitator checklist at the end of the final session)

2. Participants' experiences and views

2.1. Motivation to participate and expectations

For peer review only

Wanting to improve health and wellbeing:

I feel that being a parent-carer is really draining on your mental health and also I wanted to try and get healthy anyway, especially with it being January. Yes, I just thought it would be a good thing to try out and see if it does help me. (…) a bit of knowledge maybe about the way I feel and maybe a bit less guilt, the fact that I feel that I need to take time out for myself. (P13, control)

I was aware that my own health, fitness, mental health. All of those things really have taken quite a hit over the years, so it was a bit of a chance to actually focus on that for probably the first time in a long time really. So that was my main motivation. (…) It just seemed to fall into my lap at the right moment, so I, yes, sort of grabbed the opportunity with both hands really. (P3, intervention)

We decided to take part in the study because we wanted to be proactive as we are on most things. We knew that we needed to help ourselves as carers because we've become carers abruptly and inadvertently. (…) We've been using all the materials together [with his wife]. And we wanted to take part because we aren't going to sit on our arses and wait for something to happen because what we've learnt is the public sector will do nothing for you unless you poke it very hard. (P14, control)

I just thought it could be useful because a lot of the time I find as a parent carer all the focus is on the children which is obviously the main thing but the parents need to be healthy and happy to give the children the best. (P8, intervention)

Expecting advice, resources and signposting:

I was hoping just to find some resources to help me turn the attention off my daughter and more onto myself, just learn how to look after myself in amongst the very challenging times that we have looking after my daughter. (P15, control)

It was sort of different to what I expected because, like I say, I was expecting more sort of direct, "Right, this is who you can go to," rather than sort of what seemed like giving people information that they probably already knew. (P7, intervention)

Good timing of the programme:

If you wanted me to do the course at the beginning of last year I wouldn't have been able to do it because my daughter was very ill, but now she's in a good place at the moment so I could do it. (…) I've got to that point in my life that I have to say right, [my daughter] will always come first, she has to, and my husband and the rest of the children and grandchildren. But sometimes the work you do has to change, it has to go in a different direction (…) I've just always got on with it, but I think now I've got to that point that I think right, things have to change. (P11, intervention)

I just thought that it just fitted with the times in my life that I wanted to try and get a bit healthier and a bit less stressed. (P13, control)

Probably the fact that it was on Monday night. I've got very few timeslots that are regularly available and that one happens to be free, so. I think that probably was the biggest thing that made me go, yes let's just do it. (P2, intervention)

For peer review only

Wanting to contribute to research:

I thought it would be really helpful for me and I just felt it is quite important research, because I can't say I've looked up if there is research in this area, but I don't think there is a lot of research and I don't think unless you are in the situation, no one really gets the impact on your life and your health and everything else, it just takes over. So it just felt important for me to do for myself and also part of a bigger picture really because I think they are many many people in this situation and I don't think there's much light shed on it. I think it's getting better. But it just felt like an important thing to be part of really. (P3, intervention)

Well I thought it would help me with day-to-day stuff, but it's also an area that I have looked for help with in the past and found that there isn't any specifically, mental and physical health specific to parent carers, so I wanted to contribute to it because I want it to work [laughs], I want it to be there for when people need it. (P6, intervention)

Preference for group programme:

I was just excited to be involved in something and potentially going to be around other people… (…) I was hoping to meet other people who are in a similar situation to me and I think that you can learn a lot that way and also it's a support, meeting other people. Particularly for me, I'm very isolated and I'm assuming that there are other parent carers that are the same, that you can become very isolated and also quite often friends and family don't really understand, they say they do but you know from their actions and the things that they say sometimes that they don't really understand. So you can feel quite lonely. So having the opportunity to know that I'm going to be with other people who are in a similar situation, I was really pleased about. (P4, intervention)

I just thought that was going to be a lot more likely to be, well, something I would see through because there's a kind of commitment to turning up each week and I'm dreadful for just dropping out of things and going, oh whatever, I'm too busy… (P2, intervention)

I was a bit gutted really because I know that I always struggle with things on computers. It's not because I struggle with computers, but I always put it off to the next day and the next day, whereas if it was a group, I would just go to it as if it was an appointment. (P13, control)

I would have liked to have been part of a group to be able to discuss it with as a parent going to do similar things. (…) If there was to be a Healthy Parent workshop here in [town], I would have done it, I would have been able to find a way and I think I probably would have got more out of it from just sharing experiences and getting to know other people just for networking and support for each other. (P15, control)

Preference for, or acceptance of, online-only programme:

I wouldn't have been able to attend the groups anyway. I'm working 12-hour days, so it would have been a real rush. If you'd assigned me to the group, I probably would have asked to either drop out or go online anyway. So it worked out well in the end. (P14, control)

I was disappointed [when found out about being assigned to the group programme] because I was quite looking forward to doing the online material where I didn't have to go out, see people and talk to people, but again that is just my personal preference. It is perfect because it pushed me out of my comfort zone. I was quite apprehensive on the first day. (P9, intervention)

For peer review only

Feeling nervous before the first session:

It took a lot of self-will to take myself to the first session. It was one of those where when it came to it I was really excited and getting ready for it, then when it actually came to it I suddenly was trying to find excuses. (…) It was the sheer fact that just as it was about to start, everything around me became 10 times busier. (…) I think that was my main excuse for why I was allowing apprehension to take over. I think it is always that self-doubt factor of, is it going to be any use to me? Am I going to have to talk to loads of people and I don't really want to? It was a combination of those factors I think. (P9, intervention)

2.2. Experiences of the programme and its impact (and mechanisms)

Positive experiences:

It was fantastic. I think it was really well done. (…) I think it's changed my life. I really do. I haven't stopped talking about it since. Yes. It just makes complete sense. (…) I think the best thing was going to the group, meeting everybody in the group, just meeting like-minded people, having a break from life and being in a different place for a while, reflecting on aspects of my life that I didn't really think about or had put away, and I learnt more about myself. (P5, intervention)

I thought it was brilliant. It really has opened my eyes on an awful lot of things and I have changed an awful lot in my life. (P10, intervention)

The impact of a course like that, if that keeps me going so that I can look after my daughter, if it helps me figure out, work out our stresses in our family, keeps my relationship together, that has not just impact for our family obviously but wider in terms of taxpayers' money. So for me, I think it's really important. (P4, intervention)

I suppose they made you feel uncomfortable, which once you have got over the feeling uncomfortable about the fact that you have analysed something and thought oh I don't really like that and you can kind of box it but you think well actually no I need to address that. But that's just more about taking you out of your comfort zone really. But that's not really a bad thing, that's just an uncomfortable thing. (P1, intervention)

Lots of things! Meeting people who understand and 'get it ' reduced my isolation. Having parent carers as facilitators helped a lot. Having 12 structured sessions made me commit to it and focus on my own health. Having structured fun informative sessions helped a lot - I definitely went through a process of change. The use of humour was really important to me - and learning to be more resilient. (End-of-programme feedback)

Learning how useful and how important it is to keep myself healthy, not only for myself but for my child family and friends. Learning how to reflect and realising the amount I already do for myself and others that I am now proud of achieving a good sense of self-worth. Using Smart goals. (End-of-programme feedback)

I just think it was excellent and totally life changing!! Every parent carer in the UK and worldwide should have access to this course!! Thank you so so so much!! (End-of-programme feedback)

For peer review only

The group:

Being part of a group feeling like my challenges were understood and acknowledged. We laughed a lot cried sometimes and developed a lovely supportive friendship. I felt that the course was a really good balance of talking to off-load whilst also being 'solution focussed'. This was very empowering. Whilst my situation remains very challenging, I feel I have the skills and knowledge to help myself whilst also now having a wonderful new group of supportive friends. I am so grateful for this experience. Thank you. (End-of-programme feedback)

Our group worked really well actually and it really evolved, which was lovely because there were people who were thinking they weren't going to make it through to the end and they did. (…) We had quite an empathetic group and there was sort of, now that I'm thinking about it, possibly an unwritten code. (…) There was a lot of respect in the group. Obviously, there's some people whose kids have mental health issues and having major issues. There was a lot of well let's just sit and make sure we listen. There was a lot of support and everybody injected into the study, nobody sort of sat back, or if they were uncomfortable they said they were. As a group, it did, it evolved and it moulded and it's a really nice group. (P1, intervention)

I found it all helpful. I think the… and again, because it was a group, and that's why I would say I'm really glad that I was on the group side of it, the interaction and people's knowledge and people's just experiences and you kind of… but that sort of camaraderie type of feeling really, that's probably what I got more… probably, and then, yes, you put that down and the connecting that we did really. There's lots and lots of connecting really and… Yes, there was lots and lots of all of it actually, thinking about it, but just people putting through ideas of, "Well I do this, I don't know if that would work for you". There was a lot of that and it was, yes, that's probably what I got mostly from it. Yes. (P1, intervention)

It was a really nice group of people, everyone was very… I'd say everyone was very open and honest, people were able to talk about quite difficult circumstances and situations and it felt like a supportive environment and people were able to get upset and feel supported I think and also we did have a really good laugh, which was brilliant. I think we were really lucky. We are planning to keep in touch. Typical carers, no one can find the time when we are all available. It just sums it up really, doesn't it? But it was such a positive experience. After the first night I came out on a bit of a high because it's just always so… because friends and family and they care, but people just don't get it in the same way as when you are with a group of people who just know how bleak it can be, yes. (P3, intervention)

What I loved about the course was that it was very solution focused and I think quite often you can go to groups and things that it's just about talking, and as helpful as that is, you still come away with it going, well I'm not really sure… It was nice, but I don't really know how to make things better still, so to go to a group that's like you have the opportunity to talk and to gain help from other people and be listened to, great, but let's put that to one side, what are the barriers and how can we overcome them and let's work together to help each other overcome our barriers to having a more healthy and greater wellbeing in our lives. So I think if I'd have known that beforehand, I would have been even more excited. So maybe to have known that that was what it was trying to achieve. (P4, intervention)

For peer review only

I liked being around other people who were in a similar position to me. I liked the fact that we could listen to each other and offer support and help and useful advice. There was a lot of that that went on between us. Thinking creatively about how… from everybody's own experiences, what knowledge do I have that can help you? So I liked the supportiveness of the group. It wasn't all serious either, we laughed a lot, and that was lovely to have those moments where we shared silly jokes. It really was a case of we laughed together and we cried together, do you know what I mean, and we helped each other. So it was a really lovely group to be in and I really liked that. (P4, intervention)

We all got to share our stories, we all got to know each other, we all gave each other presents at the end and had a little party. It was really good. I think we all really became different people throughout the course. (…) I think the best thing was going to the group, meeting everybody in the group, just meeting like-minded people, having a break from life and being in a different place for a while, reflecting on aspects of my life that I didn't really think about or had put away, and I learnt more about myself. (P5, intervention)

When somebody was missing it kind of really felt like somebody was missing because we all had very different perspectives and different situations and it was really interesting. The biggest part of the thing for me was not what came out of the structure but out of the conversations that we had between us. Everybody interacted equally with everybody else across the six weeks, so there wasn't like two people who got on really well and had lots more in common and went off and talked between themselves, it was like completely among the group and it wasn't… In some weeks one of us might be quiet for our own reasons and that was fine. Everybody was very… I don't know, sort of accepting of other people's contribution, whether they were contributing a lot or not very much that week. (P6, intervention)

Everybody was listening and it was nice… near the end of the… the latter sessions, we were very much… if you understand what I mean, as a group, we were able to have little jokes together and bring up things that we discussed before. It was very nice. It was very much a camaraderie if you know what I mean. It was really nice and everybody was nice to everybody. There was not any anger or frustration. You could see people were struggling with certain things. We were able to advise them 'Actually, you are better than what you think' so it was really nice. (P12, intervention)

Group size:
It was smaller than I expected. I was expecting a group twice the size. And in some ways that might have been better because it might have been easier to find maybe subgroups within that that I could get… because there were definitely individuals in that group I could definitely get along with better but instead because it was very small it felt quite kind of intense which then meant that, yes, each person ends up trying to make it large and that… yes, and in a way that almost makes it harder to share. Yes, I don't know. I think I probably would have preferred a slightly larger group actually, but not massive. (P2, intervention)

There was only three of us, which was kind of a shame there wasn't more but, at the same time, we couldn't have got as much out of it as we did because we all got to share our stories, we all got to know each other, we all gave each other presents at the end and had a little party. (P5, intervention)

I think if the group was a little bit bigger, maybe not massively bigger but maybe, say, nine to ten people, I think you would get more input and there would be more discussion because even though

For peer review only

we were able to discuss things, I think a bit more of an open, broader amount of different people with different lives would make it a lot more interesting. (P12, intervention)

Peer facilitators:

We were sometimes a bit naughty on time when we talked too much, but I think that just happens when you are in that group. But yes, we managed to cover everything. There was ample time. They made sure everyone was included and had a say at every step really. I don't think there was anyone who didn't feel they could contribute or felt left out. (P3, intervention)

[Facilitators were] Brilliant, brilliant. And they were bouncing off each other. It was not just coming from one person. Because sometimes, you know when they do these things and you are only getting it from one perspective and [name] was able to challenge that perspective and then we were all able to challenge it and it was nice to be able to share rather than actually going, 'We are learning off a book that they were using to get their information' but actually, we were having to break it up even more on the sheets in front of us and where they were writing it all down, we were able to take notes and everything, so it was really good. (P12, intervention)

They really kept things going. They both had quite different approaches which I think complemented each other. Yes, it just seemed to work really well as a complete group including the facilitators really. (P3, intervention)

I think it makes a massive difference that they were both parent carers because they obviously have that innate understanding. I think it would have been different if they hadn't been, if the facilitators hadn't experienced some of those issues, I think it would have been very different. So I think that was a massive bonus. Because it's almost like a short hand, isn't it, there's things you don't even have to explain or go into because you just know that there's an understanding there already. So I think that was really helpful. (P3, intervention)

I think that's really helpful and important because it's not… Both of them are parent carers themselves and have been through very difficult situations and were open about sharing some of their challenges and their experiences and I think that that helps you then feel that it's okay to express what you are going through and that it's understood, because I think in those situations where it's being delivered by somebody who is reading the material without having had any of those challenges themselves, makes it less believable, less… like going to a dieting class that's being led by someone who has never had any problems, challenges with their weight, do you know what I mean? (…) It makes it less believable and somebody preaching to you but coming from a place that they have no experience of but they are preaching to you about how you can make changes in your life. And I think for me then I go I'm not sure if I can… It makes me not want to… It's less genuine, I guess. So for me having people facilitate the programme who have both had those challenges and, as I said, were quite open about their own challenges, and each week when we were going through the different clangers or whatever, they were honest at times, saying, "Well I've struggled this week to do it," and so it makes you feel it's okay that I'm struggling with this as well. And we all kind of worked together to help each other and encourage each other and that was a really lovely thing. Really lovely thing. (P4, intervention)

The facilitators of the programme were excellent in that they helped to create an environment where, as I said, it felt safe to talk about these things, that you were understood, that they cared about what you were going through and that very clearly came across from both of the facilitators, that they genuinely cared about what we were all going through and our challenges and also that they were honest about their own challenges. (P4, intervention)

The facilitators were very experienced (…) [they] were really knowledgeable, made us feel very relaxed, and I think that was a great part of it as well. (P11, intervention)

[Facilitator] was great, really good and whist she was delivering a programme she was absolutely excellent at tuning into individual people's situations and responses to things without singling them out. I could see she was being very empathetic and helping different people to get through different bits of it individually but as a group, if that makes sense, which I think was a real skill. She wasn't just delivering a programme, she was also doing it as individuals and again I think being a very small group really helped that. [Facilitator 2], yes, she was lovely. I mean, she was very good, kind. (P6, intervention)

They were lovely. Both of them were fantastic. I cried at times and they were caring, they were welcoming when you got there, they understood and remembered things. You felt like you got to know them and they got to know you and they'd talk about their experiences as well. I couldn't fault them at all, either of them, they were both fantastic. (P8, intervention)

This was doable because the facilitators weren't just 'professionals' preaching at us. (End-of-programme feedback)

The 2 tutors delivering the course were absolutely amazing! Being parent carers themselves really made them part of the group and not just 'teachers'. They were both friendly, welcoming, caring and informative. They made the course something to look forward to each week. (End-of-programme feedback)

Having the course delivered by another parent carer helped you to feel more at ease with disclosing information about your life and to someone who could totally relate and understand you circumstances. (End-of-programme feedback)

Content and activities:
The content of the course was good, it was really helpful and I know that it's going to help me going forward and I know that I am going to keep referring back to that to help me. (P4, intervention)

I think on every feedback form after every week it was like what was good about it and I was like discussion and sharing [laughs] which didn't come directly from the content of the programme but it made that happen. So that for me was the biggest thing because those are the things that stick, someone else's experience or if somebody else is maybe struggling with something, you'd support each other and you would kind of remember those things, you remember the personal stuff, human interaction that you have with people, whichever way, more than stuff you have been told. So I thought that was great. (…) It just seemed like a very natural way with that structure and the structure almost kind of… kind of like disappeared a bit into the background. I mean, sometimes to keep things moving, [facilitator] would have to bring us back to it and move on and stop one thing and start another thing, but it was almost like that became like a supporting thing, so the structure supported what happened in the session, it didn't drive what happened in the session, although it gave it structure but the session didn't hang on the content. (P6, intervention)

I think it would be relevant to everybody actually. I think everyone could probably do with doing that course. I think as parent carers we have additional challenges, obviously certainly lots of the stress and the anxiety associated with your children who are struggling, but I thought that the content

For peer review only

really helped you to focus on how do I keep myself healthy and look after my wellbeing, because if I fall down then I can't support my children. And I think that's really really important because I think so often families don't get support until it's too late. (P4, intervention)

I think it's relevant to all human beings [laughs], but I think putting it in the context of being a parent carer, yes I did find it very helpful and yes they were all the things. I don't think there was anything in there that was kind of superfluous and I don't think there was anything in there that was, I don't know. Yes, just when you blend them, when you blend the CLANGERS, they cover everything in life really. So parent carers, I didn't feel there was anything there, they were all things that were accessible. So there weren't things in there like for a lot of parent carers would feel like a non-starter, overwhelming, unachievable. They were all sort of day-to-day things that we all need to look at. Yes, I think they absolutely covered the typical things. Not to say that other people don't struggle with them but that parent carers particularly are more likely to struggle with more than the average person. (P6, intervention)

Learning about the CLANGERS and realistic ways to apply them - small steps. Having a laugh. (Weekly feedback, module 2)

Everything today I could relate to, enjoyed the videos, very informative and has made me look at how I can change to help improve my life. (Weekly feedback, module 3)

Looking at achievability of fitting in more exercise / active stuff. Looking for opportunities rather than barriers. (Weekly feedback, module 5)

Really enjoyed doing a 'top tips' exercise. It was a nice reminder of the CLANGERS. Revisiting the 'wheel' to see how far I have come. Very positive for me. (Weekly feedback, module 12)

Having 12 structured sessions made me commit to it and focus on my own health. Having structured, fun, informative, sessions helped a lot - I definitely went through a process of change. (End-of-the-programme feedback)

Loved the CLANGERS definitely made me think about the areas of my own wellbeing I need to work on. (End-of-the-programme feedback)

Reading the information made me think more about things I can do to help myself feel better. I even completed a cross stitch for the learning something new part. The CLANGERS acted as prompts to get me thinking about things I needed to do to give myself some time for myself. (End-of-the-programme feedback)

Being able to reflect on what's working and what isn't by going through the resources and questionnaire in a structured way. (End-of-the-programme feedback)

Being reminded and prompted:

I just am aware of… perhaps more aware of what's wrong, but I don't know that I've made, yes, any great changes to anything. I mean, I went away and said I'd do various things, and some of them I think were okay. I suppose it gives me more of a picture of what doing well might look like. (…) I'd have made them before if I could. I wasn't unaware… (P2, intervention)

It gave me a big kick up the bum to do something, I think that's been helpful. (P13, control)

For peer review only

A lot of it is very common sense stuff… you don't think about or focus on things enough. The CLANGERS, I've discussed that with quite a few people, because it's so simple, isn't it, but it really did help me to focus on yes just all of those things really. (…) It's like a revelation without it being a revelation, if you know what I mean, because you know all that stuff, but you don't take the time to think about it and it was just very much about focusing on us and improving things for ourselves before we can do it for everyone else really. (P3, intervention)

It was all about trying to make sure we reminded ourselves what we already knew but take the time to try and make sure we can sustain ourselves because sometimes you just get really tired, physically and emotionally. (P14, control)

It's not like a six-week programme where you go in not knowing something at the beginning and you come out knowing something at the end or being able to do something at the end that you didn't already know in your heart. It's a six-week programme that concentrates on reminding you of things that you can do and having had that reminder in a concentrated way it's with me all the time. (P6, intervention)

The CLANGERS acted as prompts to get me thinking about things I needed to do to give myself some time for myself. Being able to reflect on what's working and what isn't by going through the resources and questionnaire in a structured way. (End-of-programme feedback)

Reflecting and setting goals:
I think acceptance is a big thing, so accepting what you can't change. And we talked a lot about that on the course, looking at what are our challenges. (…) So it's kind of separating what you can and can't change I think helps you make steps forward, so going, okay, well this is the way it is, I can't do anything about that, so I'm just going to accept it, and now okay what can I change, what do I have control over and then breaking that down again into what are the specific barriers to me making those changes and working on those. (P4, intervention)

I think what causes the stress and anxiety and things like that for me and kind of the downward spiral that you can get into is that feeling that you don't have control, that everything is happening to you and you can't take control of it (…) So you can end up feeling like you don't have control over anything in your life anymore and that's when things start to spiral. So to stop for a minute and go, okay, well let's just think about my health and wellbeing and to break it down in a way that you can go, okay, this is what I know I need to do to be healthy and for my wellbeing for me to carry on doing this, but often everything can feel so overwhelming that you don't know where to start. So to be able to break it down like that and then go, right, I know I need to do this for my wellbeing, what are my barriers, what's stopping me from doing it, and then to analyse that and go, okay, well how can I realistically overcome that to make a positive change gives you… makes you feel like you have more control again. It brings back some control into being able to help yourself, which I think is really important, is having the tools and the skills to be able to help yourself, which is what I felt that course gave me. (P4, intervention)

It taught me how to break down those things into manageable chunks and to know that even a small change is good, that it doesn't… because I think so often we get caught up in the fact that it's all or nothing, right? You have to make that change and you have to do it all the time otherwise it's not worth it, and I think it's recognising that even small changes can make a big difference and being

For peer review only

able to work out how to make those changes, as I said, by breaking it down and looking at what my barriers are. (P4, intervention)

The message was you don't have to make massive changes to improve things. So I'm just trying to, for me and the kids really, tweak things a little bit to try and improve things a bit so it doesn't feel like an onerous task and I don't have to go to a class, I can sort of fit in activity as and when. That's quite reassuring really. So it's about making little changes that you can achieve rather than setting massive goals that you are just going to fail and then feel like a failure. (P3, intervention)

When you do the diary, ticking those and understanding what each planner meant it was suddenly occurring to me that I didn't eat very well, I didn't relax, I didn't sleep, so I needed to focus more on that side of my life. That really helped – the tick chart and setting goals. (…) What might stop you was literally time, so I know I needed to make adjustments. (P10, intervention)

Focusing on oneself:
You know all that stuff, but you don't take the time to think about it and it was just very much about focusing on us and improving things for ourselves before we can do it for everyone else really. (…) it has made me focus much more on my health and wellbeing and thinking I've just got to give myself that time. There's loads of things that I need to do, but at the minute I just need to get better. (P3, intervention

To be honest, it is all basic stuff, isn't it? It is just bringing it to the forefront, that actually, you are a worthy person and it is important that you do something for you, as well as your family and everybody else. (P18, control)

It is about making the carer feel that they are just as important as the caree. Because if we do not focus on ourselves, which is something we learnt, then you cannot work well for the person that you are caring for. (P12, intervention)

I think because of the way it was designed as well, we found out more about the people ourselves. We were ourselves in that. Even though we were carers and we were there because we were carers, we were talking about how we were. (P1, intervention)

Less positive experiences, limited impact:
The thing is, I found it helpful and I know I will continue to find it helpful. I can't say anything has changed as a direct result. Well, maybe some things have changed as a direct result of the programme but because I had an injury and because I'm just tired all the time, it's sort of like well that hasn't really changed [laughs] and it's not going to change. I think it gave me more hope that I will use… It's not like a six-week programme where you go in not knowing something at the beginning and you come out knowing something at the end or being able to do something at the end that you didn't already know in your heart. It's a six-week programme that concentrates on reminding you of things that you can do and having had that reminder in a concentrated way it's with me all the time. So as an example, I'm in a fairly poor state at the moment, not in life, nothing terrible but I'm really tired and struggling with this damn finger and feeling a bit like I'll never get things back to normal, but I know I will and I know what things I'm going to do to do that and the CLANGERS thing just… yes, it just stuck with me and I'm not speaking for other people but I could say that it did for the group, the other participants. It didn't make a massive short-term difference in my life. It did make a massive difference in my

For peer review only

thinking and my awareness and hope that [laughs]… I think it's hope. It gives you hope that you can get back on track no matter what, I'd say. (P6, intervention)

It was interesting, it showed me lots of aspects that I could look at. It wasn't just one thing to change, there were lots of things and one of the videos stuck in my mind. I keep thinking of that, but I haven't actually motivated myself to do anything about that just half an hour of your day. There's like 24 hours in the day and it's just like all we are asking for is half an hour to actually go and do some exercise. So that was the most beneficial that you could do to try and lift your wellbeing and your health and everything, I thought yeah, yeah. I'd got it in my head, but I still hadn't done anything which is a bit… I feel a bit annoyed with myself that I haven't. I thought that in my head all the time why don't I just do that half hour? (P16, control)

I think I probably would have got more if there had been more time for chatting because that was pretty much banned and the time was very limited because of the research element of it, whereas I think in a group like that people would chat normally, so probably the loo break was the best bit in terms of chatting, which sounds weird but it's a bit unnatural to ban chatting. (…) Some of the topics were much heavier than others and bigger than others and that wasn't reflected in the time allocation, so some of it felt like a bit of time filling and all the kind of introductory and end sessions felt a bit time filling when the actual topics really could have done with a lot more depth in some cases. (P2, intervention)

Right at the start was when we did the connecting. Well, it was a big one… I know CLANGERS starts with a C so you start with connect, but connecting is actually a really big one and actually can cause… I know we were talking about connecting with people outside, you are walking the dog, you chat with people you see and say hi, but you can actually come away feeling more lonely than you did when you went out and connected with them. (P1, intervention)

The one I disliked most of all was… It might have been relax. It was a lot to do with managing stress and it was all related to work type stress and very unrelated to the experiences that you have as a parent carer. Yes. Mmm. Once upon a time I had a job and that just pissed me off even more because actually I miss having a job enormously and it's just like [sighs] right, yes, great, yes, people get stressed at work, mmm, that would be nice [laughs]. Just a bit sort of… that one missed the mark quite badly for me but not necessarily for everybody else because it was hitting a sore spot really. (P2, intervention)

A lot of the videos were based on how to eat properly and how the brain works when you have exercise and I think that most people sort of understand these things and how beneficial they are but not everyone has access to them and not everyone can achieve what is put across in the videos and I was sort of more expecting help on… The thing is I think what you can't get away with being a parent carer is it's supposed to be for you as a parent carer but everything that you think about and you do, you think about your children, so you can't really get away from that aspect of it. So maybe if it was done in a way that it was for a parent carer and their children and what they can achieve then it would be different. (P7, intervention)

I must admit when I was watching the 'How to make a box' video, I was thinking, 'This is quite random.' For me, that was right up my street because I love making things like that, but I was thinking, 'If this was my husband watching it, he would probably be watching it thinking "Why am I watching this video?"' (P18, control)

For peer review only

2.3. Views on online materials

Mixed views:

I probably accessed about, well, 7 or 8 of them. Nearly all of them. I don't think I looked at the last one. But it just reinforced what we did in… I didn't do them… Of course, they only came up after, didn't they? I was going to say I didn't do them before because I didn't come up before, that's why. Yes, so I only accessed them after and then I think there was one occasion where the video didn't work or something… there was some reason why we couldn't see it and then I accessed it online. So it was great to have that as a backup if other things didn't quite work out in the session and to reinforce. (P1, intervention)

There's almost always something that I need to get done and if I've got access to a computer I'd rather be doing something more pleasant or relaxing or social than going through an online course on something that isn't central to my interests I guess. So yes, I think that goes back to why I was pleased that I got the real life group because it's like yes I actually will turn up and listen to that, whereas I'm not sure I will actually get round to doing an online course, I'd probably just go yes I just dropped out. (P2, intervention)

Initially I was curious and then I think it dwindled off because I felt like the course was covering everything so thoroughly that would it add anything to what I had already had? So I think that's probably why I didn't bother so much was because, yes, I felt that everything had been covered well during the session. (P4, intervention)

I missed most of [one] session. But then I went online and did it from online. Yes. It was really easy to get into and everything was on there, but I didn't take it in as well as I did on all the other days. It was the Connect and Learn were the two CLANGERS I wasn't there for. All of the other ones I think I got more out of than that one because even though I wrote stuff down from the internet onto the pages she gave me, it didn't sink in as well discussing it. (P5, intervention)

The week that I missed, I went through and used the online materials for that. So yes, a little bit, but I didn't do it systematically alongside the group each week… Once I'm out of the room, group, I've got a hundred million other things that take priority over that. (P6, intervention)

I thought they were really good, the little videos and things like that, I thought it was really helpful information and I think had I have been accessing it every week I would have found it helpful. I had a quick flick through the weeks to just have a quick nosey what it was about at the start. But yes, I do think it was helpful information, but I just didn't follow it through. (P13, control)

I really appreciated the reminders and I really appreciated the way it was laid out in saying what commitments are you going to make today to yourself, to anybody to do these things? But some of the online stuff was very good. (…) Some of the other stuff was just lowbrow and moved on, to be honest. It didn't keep my attention. Making a box, well, get real, come on. (…) So yes, there's some very good video content and yes it fitted together quite well, it's just that there was some demeaning stuff that I didn't do anything with, I just sort of saw it and thought that's not for me, moved it to one side and moved on. (P14, control)

What was there was absolutely fantastic and I think the videos were a good way of doing it because I, for one, am better at learning through watching the videos and being able to go back to them rather than just having loads and loads of information to read because obviously, you do get jaded and you do get tired, so… yeah, I thought it was done really well. (P18, control)

For peer review only

I found some of them were a bit patronising, a bit sort of childish [laughs] and very Americanised. There were like a few which I found more helpful or were quite pleasant to do, but some of the videos I thought were a bit, yeah, a little bit childish. (P17, control)

Lack of groups as impeding the learning:

I always put it off to the next day and the next day, whereas if it was a group I would just go to it as if it was an appointment, and I forget about things all the time otherwise. (P13, control)

I think although it was very useful to have it online, I did like that, but I wish there had been an option to maybe meet up once or twice in the group. I'm the sort of person that when I'm (recording inaudible 0:15:05.2) myself I do like getting out and about and meeting other people who have got similar things and I think it would have been… If you'd have had this group locally, I would have loved to have gone every week and I think that if I'd have gone every week I probably would have finished it no matter what and I think that personally I would have maybe got even more out of it by engaging with other people. (…) Maybe just gaining new friendships, to be able to have that contact with people after the group had finished. Yes, just sharing experiences, sharing tips. Lots of people have different ways of dealing with things and you get ideas from other people or you find out about things to support your children or your family or yourself that maybe you have not heard about. (…) I think it's really useful to be able to have that physical contact with other people really. (…) if you are in a group and you went back the next week and you said, "How did you get on?" it would just encourage you to discuss what you'd done that week and, as I said, maybe learn from other people and just taking it forward, being able to just be perhaps more motivated, I guess. If you are in a group and you see lots of people benefitting it just encourages you. (P15, control)

I was a bit disappointed because I'm in [town] and I thought oh I could do this every week, it will get me out of the house, because that would have given me the incentive to go somewhere and get it and it would have been a walk and some exercise to get there. I was thinking all that in my head, but actually even getting there would be part of it, part of the looking after myself. So I thought online it wasn't going to be as useful. (…) it took a discipline to sit down and actually make that time to look at the second, go back to it. (…) I did forget about it because you've got lots of things to do, whether you had a little reminder or something where you could check in or just say what you… you know what I mean? How did you find doing that part of it or maybe a bit of homework or something [laughs]. You could say, "Oh I didn't manage to do it," but it might be that incentive just to do something, whereas if no one is checking in on you just to say, "How did you find that?" or "Did you do anything?" then you are more likely to not and to put it lower down your list, aren't you? So if I had someone who I thought they are going to ask me what I've done, even like an email, they are going to ask me what I've done, I'll feel like I've got to do it, to try it, yes, but because there was nobody doing that, I didn't push myself. (P16, control)

I found doing the online course quite isolating. Personally, I would have… I think it would have made more changes in a quicker timespan if I had been in the group one. For me, it is not so much… like I said earlier, it is having that group session, it is being with people who are going through very similar things and I think if you have got the pressure, for want of a better word, of a group session, you are more likely to make those changes. Do you know where I am coming from? It is very difficult to put into words. But for me, I have done it online and I have ticked the box, if that makes sense. I have ticked the box. Yeah, I have done it, but it is… I think it is going to be more of a… it is going to be more difficult for me to be able to actually put those things into practice having done it online than if I was going to a course and they said, 'Right. Okay, what have you done for yourself this week?' (…) I think

For peer review only

that whilst it is brilliant to have had access to the course online, I do think it is something that possibly needs to be more personable, more face-to-face rather than a computer screen. Because that does isolate you in yourself. I am taking myself away from my husband and my family to go up and do it, which is great because it is time for me. (…) I do contradict myself, I know, because in one sense, it is easier to do it online because of the timings involved and things like that with work and all the balls that you are juggling, but on the other side, it is harder. It has been harder to do. (…) There was one video that was really quite long and I will not lie, I cannot even remember what it was about now, but it was probably the day I watched it, I had a really difficult emotional day and when I was watching this really long video, I think it was about eleven minutes, and I did find that I was very tired and I did find myself… my mind wandered because you are looking at a computer screen and you are thinking around you, 'I have got to do the washing. I have got to do this.' Because I was in my own environment, I found myself thinking about everything else that I should be doing, whereas if I was in… if I had gone to the group and they were watching that video, you have got nothing else to do, you are switched off. You are switched on to be watching that video, whereas for me, I was there thinking, 'Yeah. Okay, I have got to do this, so I will sit down and do this… and then my mind… like I say, I was… at one point, I was so tired that I was thinking, 'Oh gosh, I have got to go and make lunch, something for [name] and I to have for lunch tomorrow because I have got work, I have got issues at work.' (P18, control)

I found being in the online group really hard as well I could never find time to look at the resources. If I'd have been in the face to face group I'd have had allocated time to get out and access the sessions. (End-of-the-programme feedback)

I wasn't able to attend the groups so looked at the online resources and I don't tend to follow up on things from online. (End-of-the-programme feedback)

2.4. Factors influencing ability to engage with and benefit from the programme

Commitments and inadvertent events:

Just giving myself time to do it really. I didn't prioritise it highly enough; I was just putting other things before it. I knew it was all at the back of my mind and I put a note up for me to look at it, but because there was nobody telling me, I did prioritise other things really. (P16, control)

There were certain things that came up in my life that I hadn't planned, an interview one day and stuff, that it was kind of a big commitment for that sort of six weeks to be able to sort of… if something came up and I was like, oh no, really, and I didn't want to let people down obviously, but there was a few of us that had to take days off here, there and everywhere and maybe it was a bit too long. In hindsight, it probably would have been easier to have done the online stuff. (P7, intervention)

I've had this chest infection, which I missed the last session unfortunately because I was really quite ill and I didn't want to pass it on to other people who are looking after vulnerable people particularly. (P3, intervention)

I think the people who are kind of at the not coping end of the spectrum are likely to not make a lot of the sessions because you have to drop everything all the time. (P2, intervention)

For peer review only

I put it in a sheet, put it in Excel, added a few columns, put in words and scoring and, yes, I'm quite happy for you to see it. It just explains on some days you are impacted by inadvertent events and there is no relaxation, there is five hours' sleep. That's just the way it is. (P14, control)

At the end of a very long day when you are exhausted, you have been to work, you have had a meltdown from an unstable children, you are then getting them to bed, cooking them tea, bla bla bla and then you sit down, you can read something online, but it does not necessarily go in, so it was good to print some bits off, so that I have got that for future. (P18, control)

External factors:
When you have got too much in the way of other things that you are not getting what you need, you are not going to be able to look at it. So yes, I don't know that it felt massively relevant to me because I have got bigger fish to fry. (…) I have some really quite big social care needs and health care needs of my own that are not being met and so it is much more difficult to sit there going, "Ah well, have I eaten properly today or have I…?" That kind of stuff isn't the kind of… But those things don't exist so it's better than nothing. (…) But I think it's working really well for some people. Yes, I can see that other members of the group were getting masses more out of it than I was. So I think if it's the right thing for you then it's clearly way more beneficial. (P2, intervention)

To be honest, the timing of this course could not have been worse [laughter] because my colleague has just been diagnosed with lymphoma, so he has been taken away and he is undergoing really aggressive chemo at the moment, so that means we are a man short in the office and I am thinking, 'Oh my goodness, I have got all this to contend with at the minute,' but that is a parameter that you cannot… as a group, that is not your fault. That is just one of those things. That is not something that you can control. (…) Ironically, I do not know if you have got access to my before and my after, but I actually think my after ones are possibly the answers that are not quite so good in terms of my mental health than the beginning ones but like I say, that is circumstances out of your control because it is nothing to do with reflecting on the course; it is just purely that we have had so much negativity and bad things happening in the last six weeks, that that is possibly reflected in that, but like I say, there is not an awful lot you can do because that is outside of your control. (P18, control)

Unfortunately I have experienced many personal stressful situations since starting the programme which has not helped me to focus on it as much as I had hoped if things had been stable. (End-of-programme feedback)

If there is no respite available and you'll see very little difference as you can't implement what you learn - care is 24-7 in some cases. (End-of-programme feedback)

My son was poorly so I was only able to access so much. (End-of-programme feedback)

For peer review only

Standards for Reporting Qualitative Research (SRQR)*

http://www.equator-network.org/reporting-guidelines/srqr/

Title and abstract

	Page/line no(s).
**Title** - Concise description of the nature and topic of the study Identifying the study as qualitative or indicating the approach (e.g., ethnography, grounded theory) or data collection methods (e.g., interview, focus group) is recommended	P1, line 1
**Abstract** - Summary of key elements of the study using the abstract format of the intended publication; typically includes background, purpose, methods, results, and conclusions	P3, line 1-25

Introduction

**Problem formulation** - Description and significance of the problem/phenomenon studied; review of relevant theory and empirical work; problem statement	P4, line 11-25
**Purpose or research question** - Purpose of the study and specific objectives or questions	P6, line 1-3

Methods

**Qualitative approach and research paradigm** - Qualitative approach (e.g., ethnography, grounded theory, case study, phenomenology, narrative research) and guiding theory if appropriate; identifying the research paradigm (e.g., postpositivist, constructivist/ interpretivist) is also recommended; rationale**	P7, line 1-15
**Researcher characteristics and reflexivity** - Researchers' characteristics that may influence the research, including personal attributes, qualifications/experience, relationship with participants, assumptions, and/or presuppositions; potential or actual interaction between researchers' characteristics and the research questions, approach, methods, results, and/or transferability	P6, line 24-28
**Context** - Setting/site and salient contextual factors; rationale**	Box 3, p21
**Sampling strategy** - How and why research participants, documents, or events were selected; criteria for deciding when no further sampling was necessary (e.g., sampling saturation); rationale**	Box 3, p21
**Ethical issues pertaining to human subjects** - Documentation of approval by an appropriate ethics review board and participant consent, or explanation for lack thereof; other confidentiality and data security issues	P16, line 11-14
**Data collection methods** - Types of data collected; details of data collection procedures including (as appropriate) start and stop dates of data collection and analysis, iterative process, triangulation of sources/methods, and modification of procedures in response to evolving study findings; rationale**	P6, line 24 – p7 line 21; Box 3, P21

For peer review only - http://bmjopen.bmj.com/site/about/guidelines.xhtml

**Data collection instruments and technologies** - Description of instruments (e.g., interview guides, questionnaires) and devices (e.g., audio recorders) used for data collection; if/how the instrument(s) changed over the course of the study	P6, line 25; Box 3, p21
**Units of study** - Number and relevant characteristics of participants, documents, or events included in the study; level of participation (could be reported in results)	P8, line 2
**Data processing** - Methods for processing data prior to and during analysis, including transcription, data entry, data management and security, verification of data integrity, data coding, and anonymization/de-identification of excerpts	P6, line 25
**Data analysis** - Process by which inferences, themes, etc., were identified and developed, including the researchers involved in data analysis; usually references a specific paradigm or approach; rationale**	P7, line 1
**Techniques to enhance trustworthiness** - Techniques to enhance trustworthiness and credibility of data analysis (e.g., member checking, audit trail, triangulation); rationale**	P7, line 11-16

Results/findings

**Synthesis and interpretation** - Main findings (e.g., interpretations, inferences, and themes); might include development of a theory or model, or integration with prior research or theory	P8, line 2 to p12 line 11
**Links to empirical data** - Evidence (e.g., quotes, field notes, text excerpts, photographs) to substantiate analytic findings	Box 4, 5 & supp doc 11

Discussion

**Integration with prior work, implications, transferability, and contribution(s) to the field** - Short summary of main findings; explanation of how findings and conclusions connect to, support, elaborate on, or challenge conclusions of earlier scholarship; discussion of scope of application/generalizability; identification of unique contribution(s) to scholarship in a discipline or field	P13, line 30
**Limitations** - Trustworthiness and limitations of findings	P4, line 1

Other

**Conflicts of interest** - Potential sources of influence or perceived influence on study conduct and conclusions; how these were managed	P16, line 16
**Funding** - Sources of funding and other support; role of funders in data collection, interpretation, and reporting	P15, line 29

*The authors created the SRQR by searching the literature to identify guidelines, reporting standards, and critical appraisal criteria for qualitative research; reviewing the reference lists of retrieved sources; and contacting experts to gain feedback. The SRQR aims to improve the transparency of all aspects of qualitative research by providing clear standards for reporting qualitative research.

For peer review only

For peer review only - http://bmjopen.bmj.com/site/about/guidelines.xhtml

**The rationale should briefly discuss the justification for choosing that theory, approach, method, or technique rather than other options available, the assumptions and limitations implicit in those choices, and how those choices influence study conclusions and transferability. As appropriate, the rationale for several items might be discussed together.

Reference:

O'Brien BC, Harris IB, Beckman TJ, Reed DA, Cook DA. **Standards for reporting qualitative research: a synthesis of recommendations.** *Academic Medicine*, Vol. 89, No. 9 / Sept 2014 DOI: 10.1097/ACM.0000000000000388

For peer review only
